# The burden of ischemic stroke in Eastern Europe from 1990 to 2021

**DOI:** 10.1186/s12883-025-04081-z

**Published:** 2025-02-22

**Authors:** Jingyao Xu, Shuai Hou, Zimeng Chen, Yong Liu, Xia Deng, Chunping Wang, Shijie Liu, Yanqiang Wang

**Affiliations:** 1 Emergency Department, The Affiliated Hospital of Shandong Second Medical University, Weifang, Shandong China; 2 Department II of Neurology, The Affiliated Hospital of Shandong Second Medical University, Weifang, Shandong China; 3 School of Public Health, Shandong Second Medical University, Weifang, Shandong China; 4 Emergency Department, Yantai Laiyang Central Hospital, Laiyang, Shandong China

**Keywords:** Disease burden of ischemic stroke, Eastern Europe, Disability-adjusted life year, Incidence, Mortality

## Abstract

**Background and purpose:**

Ischemic stroke is a significant public health concern, particularly in Eastern Europe, where the burden remains high. This study aims to evaluate the trends and burden of ischemic stroke in Eastern Europe from 1990 to 2021, providing insights into epidemiological changes and informing public health strategies.

**Methods:**

We used data from the Global Burden of Disease 2021 database to comprehensively assess regional and national ischemic stroke indicators in Eastern Europe. These indicators included the number of cases, incidence, number of deaths, mortality, disability-adjusted life years (DALYs), DALY rates, and estimated annual percentage change (EAPC). Joinpoint analysis was employed to examine sex-specific time trends in the burden of ischemic stroke across Eastern European countries. These estimates incorporated the Socio-Demographic Index (SDI).

**Results:**

In 2021, Eastern Europe reported 490,197 cases of ischemic stroke, with an age-standardized incidence rate (ASIR) of 142.57 (95% UI: 122.12 to 164.67), exceeding the global level. The region recorded 329,291 deaths, with an age-standardized mortality rate (ASMR) of 90.99 (95% UI: 82.79 to 98.48), significantly higher than the global rate. Disability-adjusted life years (DALYs) totaled 5,713,718, with an age-standardized DALYs rate (ASDR) of 1601.20 (95% UI: 1483.51 to 1723.12). Our joinpoint regression analysis indicates that the disease burden remains notably high in Eastern Europe, despite an overall declining trend from 1990 to 2021 in ASIR and ASMR across most countries, with estimated annual percentage changes (EAPC) of -1.13 (95% CI: -1.24 to -1.02) and − 2.78 (95% CI: -3.24 to -2.32), respectively. Lithuania reported the highest incidence rate, while the Russian Federation had the highest mortality and DALY rate. Conversely, Estonia showed significant improvements in stroke indicators. Key risk factors included low temperature and smoking, contributing notably to environmental and behavioral risks.

**Conclusion:**

Ischemic stroke continues to be a significant global health issue. Our temporal trends study results indicate that the disease burden remains notably high in Eastern Europe, particularly in Lithuania and the Russian Federation.

**Supplementary Information:**

The online version contains supplementary material available at 10.1186/s12883-025-04081-z.

## Introduction

Ischemic stroke remains a leading cause of morbidity and mortality worldwide, posing a significant public health challenge [1, 2]. Among all strokes, ischemic stroke is the predominant type, accounting for 65.3% (95% UI: 62.4 to 67.7) of all incident strokes in 2021. It resulted in the highest rates of both incidence (92.4 per 100,000, 95% UI: 79.8 to 105.8) and fatal cases (44.2 per 100,000, 95% UI: 39.5 to 47.8) compared to other stroke subtypes such as intracerebral hemorrhage and subarachnoid hemorrhage. Notably, ischemic stroke also significantly contributes to long-term disability, with an estimated 70.4 million disability-adjusted life years (DALYs) globally in 2021. Men experienced considerably higher DALY rates (975 per 100,000, 95% UI: 886 to 1070) than women (720 per 100,000, 95% UI: 643 to 792), reflecting a substantial sex-specific burden. Over the past three decades, the percentage change in DALYs for men (69.1%, 95% UI: 49.7 to 89.9) has been slightly higher than that for women (37.3%, 95% UI: 26.9 to 48.7). However, age-specific trends in DALY rates reveal a contrasting dynamic. DALY rates for ischemic stroke among individuals aged ≥ 70 years have shown a significant decline of 34.0% (95% UI: -38.6 to -29.7). In contrast, for individuals under 70 years of age, the decline has been minimal, with a reduction of only 3.0% (95% UI: -11.6 to 6.0) [3].

These epidemiological trends emphasize the urgent need for targeted interventions and effective public health strategies, particularly in view of the high morbidity and mortality associated with ischemic stroke in Eastern Europe, which is exacerbated by socio-economic and health-care disparities [4].

Previous studies have extensively documented the global burden of ischemic stroke, highlighting variations in incidence and outcomes across different regions [5, 6]. Research indicates that the incidence of ischemic stroke in Eastern Europe has consistently been higher compared to other parts of the world. This is attributed to factors such as the high prevalence of risk factors (e.g., smoking, and unhealthy diet), inadequate healthcare infrastructure, and socio-economic challenges [7, 8].

Despite a substantial body of research, significant gaps remain in understanding the long-term trends and sex-specific differences in the burden of ischemic stroke in Eastern Europe. Most previous studies have focused on global or macro-regional analyses of disease burden, with data updated only until GBD 2019. To our knowledge, there are no detailed reports specifically on the burden of ischemic stroke in individual Eastern European countries using the GBD2021 database. Additionally, many studies using secondary analysis of GBD data interpret trends primarily through the estimated annual percentage change (EAPC), which, while effective for analyzing long-term trends in age-standardized rates, has limitations in capturing recent changes in disease burden [9, 10]. Furthermore, the impact of environmental and behavioral factors on the burden of ischemic stroke in this region is not well understood, necessitating further investigation.

This study aims to fill these gaps by providing a detailed analysis of the trends and burden of ischemic stroke in Eastern Europe from 1990 to 2021. Utilizing data from the Global Burden of Disease 2021 database, we assess various ischemic stroke indicators, including the number of cases, incidence, mortality, disability-adjusted life years (DALYs), DALY rates, and estimated annual percentage change (EAPC). Additionally, we incorporate joinpoint regression models to examine sex-specific time trends in recent years across different Eastern European countries. By addressing the limitations of previous research and employing comprehensive statistical analyses, this study aims to enhance our understanding of the burden and trends of ischemic stroke in Eastern Europe, ultimately aiding in the development of effective public health interventions and policies.

## Method

### Data acquisition

The Global Burden of Disease (GBD) 2021 database (https://vizhub.healthdata.org/gbd-results/), developed by the Institute for Health Metrics and Evaluation (IHME) at the University of Washington with support from the Bill & Melinda Gates Foundation, provides comprehensive and up-to-date information on the distribution and burden of diseases and injuries globally, stratified by age, sex, location, and sociodemographic groups [11, 12]. In this study, ICD-10 (International Classification of Diseases, 10th Revision) codes were utilised for cause of death for ischemic strokes.For fatal cases, IS was classified using the following ICD-10 codes: G45-G46.8, I63-I63.9, I65-I66.9, I67.2-I67.3, I67.5-I67.6, I69.316. For non-fatal cases, the relevant codes included: I63-I63.9, I65-I66.9, I67.2-I67.3, I67.5-I67.6, I69.3 [3]. We first extracted data on the incidence, mortality, and Disability-Adjusted Life Years (DALYs) for ischemic stroke from the GBD 2021 database for the global population, different SDI stratified regions, and specifically for Eastern Europe. To determine the scope of Eastern European countries, we searched the IHME database using “Eastern Europe” as the search term, identifying seven countries in the region: Belarus, Estonia, Latvia, Lithuania, Republic of Moldova, Russian Federation, and Ukraine. Subsequently, we further refined this data to include incidence, mortality, and DALYs indicators stratified by sex and age groups within Eastern European countries.Lastly, we evaluated the burden of ischemic stroke attributable to environmental and behavioral risk factors, including environmental/occupational risks such as particulate matter pollution, ambient particulate matter pollution, household air pollution from solid fuels, and low temperatures, and behavioral risks including smoking, secondhand smoke, diet high in sodium, diet low in vegetables and fruits, diet high in processed meat, diet high in sugar-sweetened beverages, and diet low in polyunsaturated fatty acids [13].

### Socio-demographic index

SDI is a composite measure that reflects the development level of a country or region, ranging from 0 to 1, with higher values indicating higher levels of socioeconomic development [14]. For this study, the GBD database categorized countries and regions into five SDI levels: low, low-middle, middle, high-middle, and high [15]. According to 2021 GBD classifications, Estonia, Latvia, and Lithuania were assigned to the High SDI category, while Belarus, Republic of Moldova, Russian Federation, and Ukraine were categorized as High-middle SDI regions (Supplementary Table 1).

### Statistical analysis

Ischemic strokes incidence, mortality, and DALYs estimates are presented in absolute numbers and as age-standardised rates(ASR) per 100 000 population (with 95% UIs) and are stratified by age, sex, Eastern Europe and SDI.Age-standardized rates were calculated using the global age-standardized population developed for the GBD study.

UIs represent the uncertainty inherent in data sources and are derived from 1,000 draws sampled from the posterior distribution of each estimate. The upper and lower bounds correspond to the 975th and 25th values of these draws, respectively [16]. To evaluate the trends in disease burden from 1990 to 2021, we calculated the annual percentage change (EAPC) based on age-standardized rates for each year [17]. EAPC assumes a linear relationship between ASR and time, modeled as y = α + βx+ϵ, where y represents log 10(ASR), x represents the calendar year, and β represents the regression coefficient. EAPC is calculated using the formula EAPC = 100 × (10^β − 1). Unlike other estimates that use a 95% uncertainty interval (UI), EAPC is accompanied by a 95% confidence interval (95% CI). If both the EAPC and the lower limit of its 95% CI are greater than zero, the ASR is considered to show an upward trend, and vice versa [18]. To address the limitations of EAPC in capturing local temporal changes, we used the joinpoint regression model for further trend analysis, applying methods consistent with our previous research. Data analysis and visualization were performed using Joinpoint software [10, 19].Using a log-linear model with a significance level of α = 0.05, the joinpoint regression analysis was performed. The default modeling approach employed was the grid search method (GSM), supplemented by the Monte Carlo permutation method for model selection. The joinpoint model calculates the annual percentage change (APC) over the study period, along with its 95% confidence interval (CI). A significant APC deviation from zero indicates a trend, which can be classified as increasing (worsening) or decreasing (improving). Conversely, if the APC does not significantly differ from zero, the trend is considered stable or unchanged [10]. When assessing the impact of risk factors, we included the population attributable fraction (PAF) to estimate the proportion of ischemic stroke attributable to specific risk factors [20].The PAF quantifies the proportion of events attributable to specific risk factors, providing an estimate of the preventable disease burden if these factors were eliminated [21]. All statistical analyses were considered significant at *p* < 0.05, and tests were two-tailed [10]. All data analyses were conducted using R Studio software (version 4.4.0). The tidyverse R package was used for data cleaning and calculations.(https://www.tidyverse.org/).

## Results

### Temporal trends in ischemic stroke burden in Eastern Europe: incidence, mortality, and DALYs (1990–2021)

**Fig. 1:**
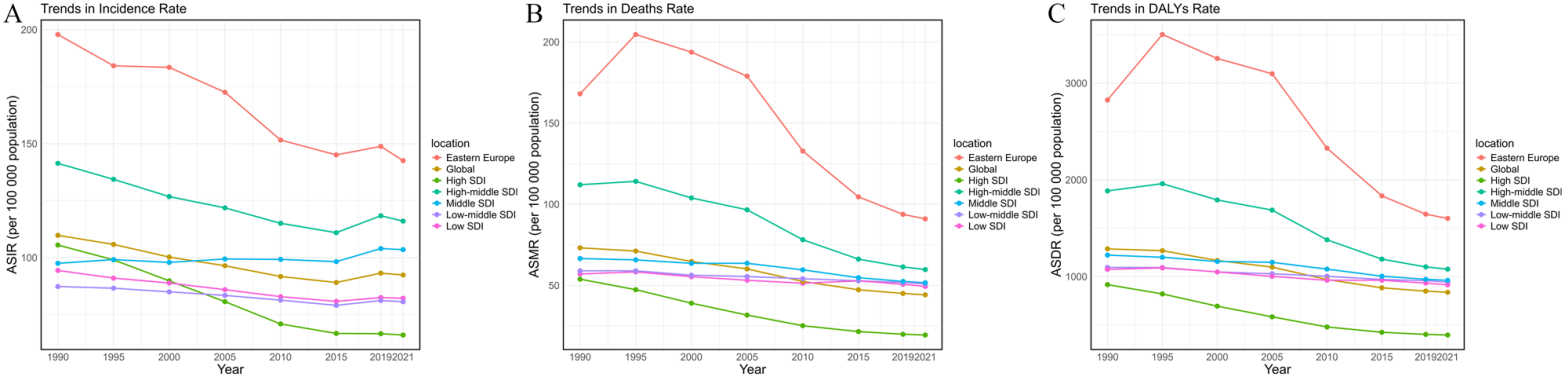
Age-standardized incidence, mortality and Disability-adjusted life years rates of ischemic stroke in Eastern Europe, globally, and across Socio-Demographic Index regions (1990–2021): **A**. Trends in Incidence Rate; **B**. Trends in Death Rate; **C**. Trends in DALYs Rate. DALYs, Disability-adjusted life years; SDI, Socio-Demographic Index

In 2021, Eastern Europe reported 490,197 (95% UI: 415,356 to 571,451) cases of ischemic stroke, with an age-standardized incidence rate of 142.57 (95% UI: 122.12 to 164.67). This incidence rate is notably higher than the global rate of 92.39 (95% UI: 79.84 to 105.82) and exceeds rates in all SDI regions: High SDI at 66.05 (95% UI: 58.24 to 74.72), High-middle SDI at 116.04 (95% UI: 99.23 to 133.86), Middle SDI at 103.51 (95% UI: 88.01 to 119.98), Low-middle SDI at 80.66 (95% UI: 69.98 to 91.67), and Low SDI at 82.17 (95% UI: 71.04 to 93.10) (Fig. 1, and Table 1).

**Table 1:** Global, regional, and national trends in ischemic stroke incidence from 1990 to 2021: detailed comparisons of case counts, age-standardized incidence rates, percentage changes, and estimated annual percentage change

Location	1990		2021		1990–2021	
	Incident cases (95% UI)	ASIR (95% UI)	Incident cases (95% UI)	ASIR (95% UI)	Cases change (%,95% UI)	EAPC (95% CI)
**Global**	4,151,978 (3536772 to 4868150)	109.79 (93.56 to 127.62)	7,804,449 (6719760 to 8943692)	92.39 (79.84 to 105.82)	87.97 (81.15 to 94.54)	-0.67 (-0.76 to -0.58)
**Regions**						
High SDI	1,153,374 (992806 to 1333283)	105.54 (91.16 to 121.17)	1,351,913 (1179761 to 1535710)	66.05 (58.24 to 74.72)	17.21 (12.48 to 22.76)	-1.75 (-1.86 to -1.64)
High-middle SDI	1,306,277 (1096265 to 1547833)	141.37 (119.89 to 164.14)	2,243,340 (1899825 to 2614463)	116.04 (99.23 to 133.86)	71.74 (64.76 to 79.11)	-0.74 (-0.85 to -0.63)
Middle SDI	953,541 (800912 to 1134827)	97.51 (81.45 to 115.65)	2,648,525 (2240569 to 3082587)	103.51 (88.01 to 119.98)	177.76 (162.95 to 193.09)	0.12 (0.07 to 0.16)
Low-middle SDI	516,851 (439586 to 603885)	87.34 (74.13 to 102.39)	1,120,427 (971630 to 1273722)	80.66 (69.98 to 91.67)	116.78 (107.33 to 126.26)	-0.34 (-0.38 to -0.3)
Low SDI	216,493 (183974 to 252019)	94.39 (80.38 to 110.81)	433,298 (374565 to 494218)	82.17 (71.04 to 93.10)	100.14 (91.68 to 108.34)	-0.51 (-0.56 to -0.46)
Eastern Europe	515,846 (425258 to 623601)	197.9 (164.89 to 232.39)	490,197 (415356 to 571451)	142.57 (122.12 to 164.67)	-4.97 (-10.96 to 0.77)	-1.13 (-1.24 to -1.02)
**Country**						
Belarus	23,266 (20087 to 26740)	183.47 (159.18 to 208.59)	22,187 (19277 to 25305)	140.59 (122.40 to 159.36)	-4.64 (-13.60 to 3.93)	-0.9 (-1.08 to -0.73)
Estonia	3383 (3056 to 3760)	170.01 (154.20 to 187.83)	1888 (1640 to 2159)	71.54 (62.07 to 81.77)	-44.19 (-50.12 to -37.78)	-3.2 (-3.42 to -2.97)
Latvia	7223 (6351 to 8312)	205.64 (181.20 to 235.84)	5679 (5047 to 6349)	137.46 (122.06 to 153.26)	-21.37 (-28.17 to -14.22)	-1.24 (-1.29 to -1.19)
Lithuania	9460 (8283 to 10533)	210.95 (184.72 to 235.11)	8869 (7639 to 10148)	151.81 (132.64 to 172.16)	-6.25 (-16.12 to 3.07)	-0.94 (-1.24 to -0.64)
Republic of Moldova	5155 (4481 to 5912)	128.90 (113.56 to 146.78)	6405 (5552 to 7300)	109.67 (95.42 to 124.36)	24.25 (12.57 to 38.57)	-0.29 (-0.38 to -0.2)
Russian Federation	325,936 (266420 to 396726)	196.72 (163.22 to 232.25)	337,709 (285475 to 395210)	144.18 (122.84 to 167.40)	3.61 (-3.59 to 10.77)	-1.01 (-1.15 to -0.88)
Ukraine	141,423 (115123 to 172949)	209.54 (172.59 to 249.65)	107,459 (89951 to 126619)	143.47 (121.28 to 166.97)	-24.02 (-30.11 to -18.14)	-1.51 (-1.63 to -1.39)

ASIR, age-standardized incidence rate; EAPC, estimated annual percentage change; SDI, Socio-Demographic Index. 95% UI: 95% uncertainty interval. 95% CI: 95% confidence interval

Eastern Europe also recorded 329,291 (95% UI: 299,911 to 356,035) deaths due to ischemic stroke, resulting in an age-standardized mortality rate of 90.99 (95% UI: 82.79 to 98.48). This rate is higher than the global mortality rate of 44.18 (95% UI: 39.29 to 47.81) and exceeds rates in all SDI regions: High SDI at 19.42 (95% UI: 16.54 to 21.03), High-middle SDI at 59.75 (95% UI: 52.99 to 65.45), Middle SDI at 51.64 (95% UI: 45.40 to 57.09), Low-middle SDI at 50.90 (95% UI: 45.37 to 57.00), and Low SDI at 49.38 (95% UI: 42.13 to 60.35) (Fig. 1, and Supplementary Table 2).

The DALYs for ischemic stroke in Eastern Europe totaled 5,713,718 (95% UI: 5,294,961 to 6,142,848), with an age-standardized DALYs rate of 1601.2 (95% UI: 1483.51 to 1723.12). This rate is higher than the global average rate of 837.36 (95% UI: 763.73 to 904.98) and surpasses rates in all SDI regions: High SDI at 395.56 (95% UI: 352.17 to 434.79), High-middle SDI at 1076.54 (95% UI: 973.38 to 1176.25), Middle SDI at 960.71 (95% UI: 863.98 to 1047.82), Low-middle SDI at 942.27 (95% UI: 846.11 to 1065.86), and Low SDI at 914.28 (95% UI: 789.76 to 1116.62) (Fig. 1, and Supplementary Table 3).

From 1990 to 2021, the number of ischemic stroke incidences in Eastern Europe decreased with an estimated annual percentage change (EAPC) of -1.13 (95% CI: -1.24 to -1.02). This decline is second only to that in High SDI regions, which experienced a decrease of -1.75 (95% CI: -1.86 to -1.64). The number of deaths decreased by -18.75% (95% UI: -24.17% to -13.28%), representing the highest decline among all regions, with an EAPC of -2.78 (95% CI: -3.24 to -2.32). This decline is second only to that in High SDI regions, which saw a decrease of -3.58 (95% CI: -3.71 to -3.44). The DALY rate decreased by -23.02% (95% UI: -27.81% to -17.93%), also the highest decline among all regions, with an EAPC of -2.61 (95% CI: -3.06 to -2.16), second only to the decline in High SDI regions, which experienced a decrease of -2.98 (95% CI: -3.11 to -2.85) (Table 1, Supplementary Tables 2–3).

### Country-specific variations in ischemic stroke burden across Eastern Europe

**Fig. 2:**
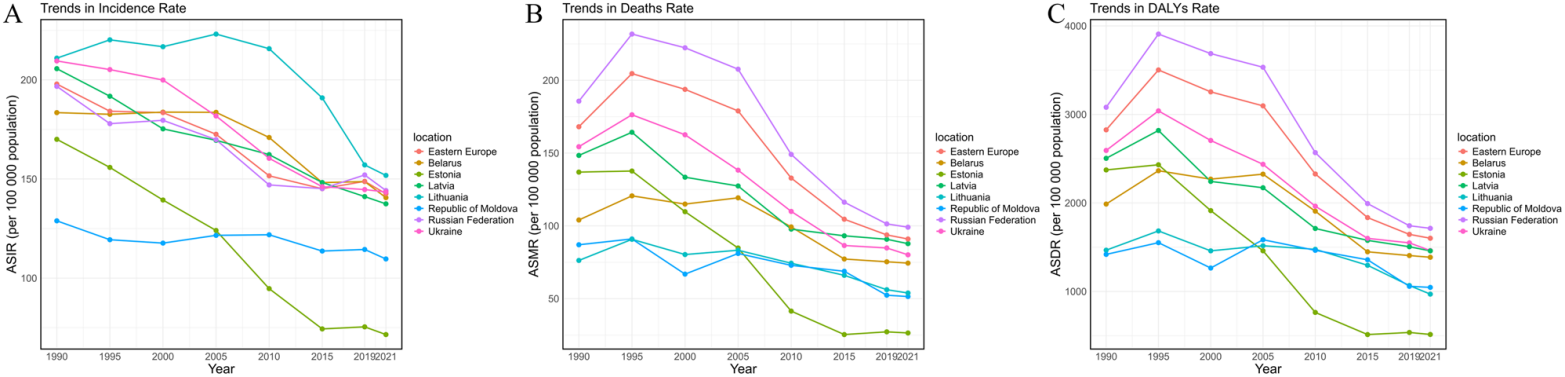
Age-standardized incidence, mortality and Disability-adjusted life years rates of ischemic stroke in Eastern European Countries (1990–2021): **A**. Trends in Incidence rate; **B**. Trends in Death rate; **C**. Trends in DALYs Rate. DALYs, Disability-adjusted life years

In 2021, Lithuania reported the highest age-standardized incidence rate of ischemic stroke at 151.81 (95% UI: 132.64 to 172.16). The Russian Federation had the highest age-standardized mortality rate at 99.09 (95% UI: 89.81 to 106.59), as well as the highest age-standardized DALYs rate at 1713.22 (95% UI: 1588.50 to 1846.08) (Fig. 2, and Supplementary Tables 4–6).

Between 1990 and 2021, the Republic of Moldova exhibited the most significant increases in incidence rate, mortality rate, and DALY rate, with changes of 24.25% (95% UI: 12.57–38.57%), 13.96% (95% UI: 1.17–31.13%), and 19.89% (95% UI: 6.63–37.40%), respectively. Despite a general downward trend in incidence, mortality, and DALY rates across Eastern European countries, the Republic of Moldova experienced the slowest declines in incidence rate and DALY rate, with EAPCs of -0.29 (95% CI: -0.38 to -0.2) and − 0.75 (95% CI: -1.17 to -0.34), respectively. Lithuania showed the slowest decline in mortality rate, with an EAPC of -1.39 (95% CI: -1.7 to -1.07). Conversely, Estonia achieved the most pronounced reductions, with case changes of -44.19% (95% CI: -50.12% to -37.78%) in incidence, -67.23% (95% CI: -71.21% to -63.40%) in mortality, and − 68.97% (95% CI: -72.53% to -65.82%) in DALYs. Similarly, Estonia’s EAPCs were the steepest in the region, at -3.2 (95% CI: -3.42 to -2.97) for incidence, -6.83 (95% CI: -7.44 to -6.22) for mortality, and − 6.3 (95% CI: -6.84 to -5.77) for DALYs, underscoring its exceptional progress in reducing the burden of ischemic stroke (Table 1, Supplementary Tables 2–3).

In 2021, the number of ischemic stroke cases, deaths, and DALYs among women in Eastern European countries exceeded those among men. However, in the majority of countries, the age-standardized incidence rate (ASIR), age-standardized mortality rate (ASMR), and age-standardized DALYs rate (ASDR) were lower in women than in men. For both sexes, the highest incidence, mortality, and DALY rates were observed in the 75 + age group. The majority of cases, deaths, and DALYs were concentrated in individuals aged 50 and above, with male cases tending to be younger than female cases (Supplementary Tables 4–6).

The joinpoint regression model analyzed trends in the age-standardized mortality rate (ASMR) and disability-adjusted life years rate (ASDR) of ischemic stroke in Eastern Europe from 1990 to 2021. The analysis showed a significant declining trend in both ASMR and ASDR across Eastern European countries during 2013–2021, indicating sustained improvements in these metrics in recent years. The incidence rate demonstrated a decreasing trend in both men and women across the region. Ukraine had the slowest decline in incidence rate from 2014 to 2021, with an APC of -0.21 (95% CI: -0.28 to -0.14) for men and − 0.45 (95% CI: -0.50 to -0.41) for women (Supplementary Figs. 1–3 and Supplementary Tables 7–9).

### Contribution of risk factors to ischemic stroke burden: focus on environmental and behavioral factors

In 2021, the population attributable fraction (PAF) for environmental risks in Eastern Europe was 19.81% (95% UI: 15.13–24.53%), with an age-standardized mortality rate (ASMR) of 18.03 (95% UI: 13.65 to 22.65). Among the four environmental risk factors analyzed—particulate matter pollution, ambient particulate matter pollution, household air pollution from solid fuels, and low temperature—low temperature demonstrated the highest population attributable fraction (PAF) of 9.56% (95% UI: 7.88–11.10%) and the highest age-standardized mortality rate (ASMR) of 8.70 (95% UI: 6.91 to 10.27). Among the countries, Latvia had the highest PAF at 10.68% (95% UI: 9.05–11.91%), while the Russian Federation recorded the highest ASMR at 9.38 (95% UI: 7.45 to 11.37). (Supplementary Figs. 4,5 and Supplementary Table 10)

For behavioral risks, the PAF in Eastern Europe was 25.11% (95% UI: 12.84–37.43%) in 2021, with an ASMR of 22.85 (95% UI: 12.17 to 34.26). Smoking was identified as the most significant behavioral risk, with a PAF of 7.49% (95% UI: 6.20–9.03%) and an ASMR of 6.81 (95% UI: 5.57 to 8.21). Belarus exhibited the highest PAF for smoking at 9.91% (95% UI: 8.24–11.83%), while the Russian Federation again had the highest ASMR at 7.40 (95% UI: 6.08 to 8.89) (Supplementary Figs. 4,6 and Supplementary Table 10).

## Discussion

Our study highlights the substantial burden of ischemic stroke in Eastern Europe, demonstrating that age-standardized incidence, mortality, and DALYs rates are significantly higher than the global average and other Socio-Demographic Index (SDI) regions. In 2021, Eastern Europe reported 490,197 cases of ischemic stroke, with an age-standardized incidence rate of 142.57 per 100,000, exceeding the global incidence rate. Lithuania had the highest incidence rate, while the Russian Federation had the highest mortality and DALY rates. Despite a general decline in stroke indicators since 1990, disparities persist among countries, with the Republic of Moldova showing a notable increase in incidence.

Our findings align with the 2019 GBD study, which also reported higher incidence, mortality, and DALY rates for stroke in Eastern Europe compared to global and other regional rates, indicating a persistently high stroke burden in this region. However, our study provides updated and detailed country-specific insights, emphasizing the unique challenges faced by Lithuania, the Russian Federation, and the Republic of Moldova.

The trends observed in this study highlight the complex interplay of demographic, socio-economic, and systemic factors driving the stroke burden in Eastern Europe. Specifically, population aging is a particularly critical factor contributing to the high burden of ischemic stroke. The aging process is strongly associated with increased prevalence of stroke risk factors, such as hypertension, atrial fibrillation, diabetes, and atherosclerosis [22]. As highlighted in the study on global aging trends, the rising proportion of older adults worldwide has led to an increased burden of non-communicable diseases, including stroke [23]. In Eastern Europe, aging populations amplify these challenges, as evidenced by the disproportionately high stroke incidence, mortality, and DALY rates among individuals aged 75 and above for both sexes. This supports the findings of Scott et al., who reported lower stroke incidence rates in younger individuals and higher rates in older age groups [24]. These trends are compounded by disparities in healthcare access and preventive measures, further exacerbating the burden of stroke in vulnerable populations [25].

Focusing on country-specific trends, the Republic of Moldova represents a striking exception to the general downward trends in ischemic stroke incidence, mortality, and DALY rates observed across Eastern Europe. Moldova’s increases in these indicators may be attributed to the combined impact of modifiable risk factors, such as abdominal obesity, dyslipidemia, hyperhomocysteinemia, and arterial hypertension, alongside socio-economic challenges like limited healthcare access and inadequate preventive measures [26]. These factors highlight the urgent need for targeted public health interventions and primary prevention strategies to mitigate the stroke burden in Moldova and other similarly affected regions.

Beyond demographic factors, regional trends also point to broader systemic factors influencing the ischemic stroke burden. For example, the observed increase in stroke incidence and mortality during the mid-1990s coincides with the socio-economic upheavals following the collapse of the Soviet Union. This period was marked by economic instability, rising psychosocial stress, and sharp reductions in healthcare funding, all of which adversely impacted public health outcomes [27].

Furthermore, Ischemic stroke is a multifactorial disease influenced by a variety of genetic, metabolic, and environmental factors [28]. In the Middle East and North Africa (MENA) region, high systolic blood pressure (53.5%), high body mass index (39.4%), and ambient particulate air pollution (27.1%) were identified as the top contributors to stroke burden [29]. Similarly, our study highlights the critical impact of environmental factors in Eastern Europe, where low temperature emerged as the leading environmental risk factor, contributing to 9.56% of ischemic stroke deaths in 2021. However, the unique socio-economic and environmental contexts of Eastern Europe, including its colder climate and differing pollution profiles, necessitate tailored interventions to address these region-specific challenges effectively.

In contrast to the concerning trends in some countries, positive examples of stroke burden reduction can be found. Conversely, Estonia serves as a positive example of how systemic reforms can effectively reduce stroke burden. Following its accession to the European Union, Estonia implemented comprehensive public health measures, improved healthcare access, and enhanced stroke care infrastructure. These reforms have led to substantial declines in stroke incidence, mortality, and DALY rates, offering valuable lessons for neighboring countries [30].

The impact of the COVID-19 pandemic on ischemic stroke burden trends warrants specific attention. The COVID-19 pandemic, which emerged in late 2019 and persisted through 2021, likely influenced ischemic stroke burden trends in Eastern Europe. While overall stroke incidence and mortality declined in most countries, pandemic-related disruptions may have contributed to observed anomalies during this period. These disruptions included delays in stroke diagnosis and treatment due to healthcare systems’ reallocation of resources to manage COVID-19 cases and patient hesitancy to seek medical care amid lockdowns and fear of infection [31]. Studies indicate that hospital admissions for stroke declined during the pandemic, with mild cases underrepresented and severe cases disproportionately prevalent.Moreover, severe COVID-19 infections are associated with a pro-thrombotic state, increasing the risk of ischemic stroke [31].This association may have counterbalanced potential reductions in stroke incidence caused by pandemic-related behavioral changes, though this remains to be confirmed by future research. Further research is needed to analyze the direct and indirect impacts of the COVID-19 pandemic on stroke burden in Eastern Europe and to assess its long-term consequences.

Examining sex-specific trends, in 2021, age-standardized incidence (ASIR), mortality (ASMR), and DALYs rates (ASDR) for ischemic stroke were lower in women than in men across Eastern European countries. This finding is consistent with the study by Norman et al., which reported higher ischemic stroke incidence rates in men compared to women [32]. This sex difference is often attributed to higher rates of hypertension, smoking, and alcohol consumption among men. Bonkhoff et al. suggested that women with acute ischemic stroke have better hospital recovery outcomes, lower in-hospital mortality, and a higher likelihood of achieving favorable functional outcomes compared to men [33].

Effective prevention and management strategies are crucial to alleviate the stroke burden [34].These strategies include reducing smoking rates, improving dietary habits, and addressing environmental risk factors. Long-term management of stroke should focus on controlling common risk factors among stroke patients, such as hypertension, diabetes, and dyslipidemia [35–37]. Implementing public health policies tailored to specific regions and improving access to healthcare services will help achieve these goals.

This study has several strengths that contribute to its significance. First, the use of the Global Burden of Disease (GBD) 2021 database provides a comprehensive and standardized framework for assessing the burden of ischemic stroke across Eastern Europe. This enables robust comparisons across regions, sexes, and age groups, and allows for the identification of temporal trends over a 30-year period. Second, the inclusion of detailed analyses on environmental and behavioral risk factors, such as low temperature and smoking, provides actionable insights for tailoring public health interventions. Lastly, employing joinpoint regression modeling and estimating annual percentage changes (EAPC) adds statistical rigor, ensuring a nuanced understanding of trends over time.

However, several limitations should be noted when interpreting the findings. First, the study did not analyze the burden of ischemic stroke subtypes, which may have varying risk factors, management strategies, and outcomes. This limitation restricts the granularity of the conclusions and highlights the need for future research to explore these subtypes. Second, while this study assessed major environmental and behavioral risk factors, it did not account for alcohol consumption — a significant contributor to stroke risk in Eastern Europe. The exclusion of alcohol consumption limits the study’s comprehensiveness in evaluating preventable risk factors. Third, the reliance on secondary data from the GBD database introduces potential biases related to the accuracy and completeness of data collection, particularly in regions with underdeveloped healthcare reporting systems. This may lead to an underestimation of the true burden of ischemic stroke, especially in low- and middle-income countries within the region. Lastly, the modeling techniques employed in GBD analyses, while robust, rely on assumptions that may introduce uncertainties in the estimates. These limitations underscore the need for caution in interpreting the findings and highlight areas for future investigation.Addressing these limitations in subsequent research will further refine our understanding of ischemic stroke epidemiology and support the development of more targeted and effective public health strategies.

In conclusion, our study underscores the substantial burden of ischemic stroke in Eastern Europe, characterized by higher age-standardized incidence, mortality, and DALYs rates compared to global averages and other Socio-Demographic Index (SDI) regions. While notable progress has been made in reducing the ischemic stroke burden in many countries, significant disparities persist across nations, sexes, and age groups. Men consistently exhibited higher age-standardized rates than women, and individuals aged 75 and above bore the highest burden of ischemic stroke.The sustained improvements in age-standardized mortality and DALYs rates from 2013 to 2021 highlight the potential of targeted health interventions. However, persistent challenges, including modifiable risk factors, healthcare access inequalities, and systemic factors such as socio-economic transitions and environmental risks, underscore the need for region-specific prevention and management strategies.Future research should prioritize understanding the direct and indirect impacts of systemic, environmental, and behavioral factors on stroke burden while developing tailored interventions to address these region-specific challenges. Strengthened public health policies and equitable healthcare resource allocation are critical to reducing the burden of ischemic stroke and improving outcomes across Eastern Europe.

## Electronic supplementary material

Below is the link to the electronic supplementary material.

Supplementary Material 1

Supplementary Material 2

## Data Availability

The data that support the findings of this study are openly available in The Global Burden of Disease (GBD) 2021 database (https://vizhub.healthdata.org/gbd-results/).

## References

[1] Budincevic H, Tiu C, Bereczki D, Korv J, Tsiskaridze A, Niederkorn K, et al. Management of ischemic stroke in Central and Eastern Europe. Int J Stroke. 2015;10(Suppl A):125–7. 10.1111/ijs.12575. 26179030 10.1111/ijs.12575

[2] Hartley A, Marshall DC, Salciccioli JD, Sikkel MB, Maruthappu M, Shalhoub J. Trends in Mortality from Ischemic Heart Disease and Cerebrovascular Disease in Europe: 1980 to 2009. Circulation. 2016;133 20:1916–26. 10.1161/CIRCULATIONAHA.115.018931. 27006480 10.1161/CIRCULATIONAHA.115.018931

[3] Collaborators GBDSRF. Global, regional, and national burden of stroke and its risk factors, 1990–2021: a systematic analysis for the global burden of Disease Study 2021. Lancet Neurol. 2024;23 10:973–1003. 10.1016/S1474-4422(24)00369-7.39304265 10.1016/S1474-4422(24)00369-7

[4] Wafa HA, Wolfe CDA, Emmett E, Roth GA, Johnson CO, Wang Y. Burden of stroke in Europe: thirty-year projections of incidence, prevalence, deaths, and disability-adjusted life years. Stroke. 2020;51 8:2418–27. 10.1161/STROKEAHA.120.029606. 32646325 10.1161/STROKEAHA.120.029606 PMC7382540

[5] Ding Q, Liu S, Yao Y, Liu H, Cai T, Han L. Global, Regional, and National Burden of ischemic stroke, 1990–2019. Neurology. 2022;98(3):e279–90. 10.1212/WNL.0000000000013115. 34911748 10.1212/WNL.0000000000013115

[6] Krishnamurthi RV, Ikeda T, Feigin VL. Global, Regional and Country-Specific Burden of Ischaemic Stroke, Intracerebral Haemorrhage and Subarachnoid Haemorrhage: a systematic analysis of the global burden of Disease Study 2017. Neuroepidemiology. 2020;54 2:171–9. 10.1159/000506396. 32079017 10.1159/000506396

[7] Andersen KK, Steding-Jessen M, Dalton SO, Olsen TS. Socioeconomic position and incidence of ischemic stroke in Denmark 2003–2012. A nationwide hospital-based study. J Am Heart Assoc. 2014;3(4). 10.1161/JAHA.113.000762. 10.1161/JAHA.113.000762 PMC4310360 25030354

[8] Fan J, Li X, Yu X, Liu Z, Jiang Y, Fang Y, et al. Global Burden, risk factor analysis, and Prediction Study of ischemic stroke, 1990–2030. Neurology. 2023;101(2):e137–50. 10.1212/WNL.0000000000207387. 37197995 10.1212/WNL.0000000000207387 PMC10351546

[9] Zhang K, Kan C, Han F, Zhang J, Ding C, Guo Z, et al. Global, Regional, and National Epidemiology of Diabetes in Children from 1990 to 2019. JAMA Pediatr. 2023;177 8:837–46. 10.1001/jamapediatrics.2023.2029. 37399036 10.1001/jamapediatrics.2023.2029 PMC10318549

[10] Hou S, Pang M, Zhang Y, Xia Y, Wang Y, Wang G. Assessing tobacco-related ischemic stroke in Pakistan (1990–2019): insights from the global burden of Disease Study. Tob Induc Dis. 2024;22. 10.18332/tid/185566. 10.18332/tid/185566 PMC10973799 38550907

[11] Diseases GBD, Injuries C. Global incidence, prevalence, years lived with disability (YLDs), disability-adjusted life-years (DALYs), and healthy life expectancy (HALE) for 371 diseases and injuries in 204 countries and territories and 811 subnational locations, 1990–2021: a systematic analysis for the global burden of Disease Study 2021. Lancet. 2024;403 10440:2133–61. 10.1016/S0140-6736(24)00757-8. 38642570 10.1016/S0140-6736(24)00757-8 PMC11122111

[12] Collaborators GBDCD. Global burden of 288 causes of death and life expectancy decomposition in 204 countries and territories and 811 subnational locations, 1990–2021: a systematic analysis for the global burden of Disease Study 2021. Lancet. 2024;403 10440:2100–32. 10.1016/S0140-6736(24)00367-2. 38582094 10.1016/S0140-6736(24)00367-2 PMC11126520

[13] Collaborators GBDRF. Global burden and strength of evidence for 88 risk factors in 204 countries and 811 subnational locations, 1990–2021: a systematic analysis for the global burden of Disease Study 2021. Lancet. 2024;403 10440:2162–203. 10.1016/S0140-6736(24)00933-4. 38762324 10.1016/S0140-6736(24)00933-4 PMC11120204

[14] Diseases GBD, Injuries C. Global burden of 369 diseases and injuries in 204 countries and territories, 1990–2019: a systematic analysis for the global burden of Disease Study 2019. Lancet. 2020;396 10258:1204–22. 10.1016/S0140-6736(20)30925-9. 33069326 10.1016/S0140-6736(20)30925-9 PMC7567026

[15] Global Burden of Disease, Cancer C, Kocarnik JM, Compton K, Dean FE, Fu W, Gaw BL, et al. Cancer Incidence, Mortality, Years of Life Lost, Years lived with disability, and disability-adjusted life years for 29 Cancer groups from 2010 to 2019: a systematic analysis for the global burden of Disease Study 2019. JAMA Oncol. 2022;8(3):420–44. 10.1001/jamaoncol.2021.6987. 34967848 10.1001/jamaoncol.2021.6987 PMC8719276

[16] Deuschl G, Beghi E, Fazekas F, Varga T, Christoforidi KA, Sipido E, et al. The burden of neurological diseases in Europe: an analysis for the global burden of Disease Study 2017. Lancet Public Health. 2020;5 10:e551–67. 10.1016/S2468-2667(20)30190-0. 33007212 10.1016/S2468-2667(20)30190-0

[17] Ou Z, Pan J, Tang S, Duan D, Yu D, Nong H, et al. Global trends in the incidence, prevalence, and years lived with disability of Parkinson’s Disease in 204 Countries/Territories from 1990 to 2019. Front Public Health. 2021;9:776847. 10.3389/fpubh.2021.776847. 34950630 10.3389/fpubh.2021.776847 PMC8688697

[18] Dong Y, Peng R, Kang H, Song K, Guo Q, Zhao H, et al. Global incidence, prevalence, and disability of vertebral fractures: a systematic analysis of the global burden of disease study 2019. Spine J. 2022;22 5:857–68. 10.1016/j.spinee.2021.12.007. 34906740 10.1016/j.spinee.2021.12.007

[19] Kim HJ, Fay MP, Feuer EJ, Midthune DN. Permutation tests for joinpoint regression with applications to cancer rates. Stat Med. 2000;19 3:335– 51; doi: 10.1002/(sici)1097-0258(20000215)19:3-335::aid-sim336-3.0.co;2-z. 10.1002/(sici)1097-0258(20000215)19:3<335::aid-sim336>3.0.co;2-z 10649300

[20] Hankey GJ. Population Impact of potentially modifiable risk factors for stroke. Stroke. 2020;51 3:719–28. 10.1161/STROKEAHA.119.024154. 32078497 10.1161/STROKEAHA.119.024154

[21] Zou J, Sun T, Song X, Liu YM, Lei F, Chen MM, et al. Distributions and trends of the global burden of COPD attributable to risk factors by SDI, age, and sex from 1990 to 2019: a systematic analysis of GBD 2019 data. Respir Res. 2022;23(1:90). 10.1186/s12931-022-02011-y. 10.1186/s12931-022-02011-y PMC8996417 35410227

[22] Satue E, Vila-Corcoles A, Ochoa-Gondar O, de Diego C, Forcadell MJ, Rodriguez-Blanco T, et al. Incidence and risk conditions of ischemic stroke in older adults. Acta Neurol Scand. 2016;134 4:250–7. 10.1111/ane.12535. 26592375 10.1111/ane.12535

[23] Safiri S, Motlagh Asghari K, Sullman MJM. The Global Burden of diseases and injuries among older adults. Int J Aging. 2023;1 1:e16–e. 10.34172/ija.2023.e16.

[24] Scott CA, Li L, Rothwell PM. Diverging temporal trends in Stroke incidence in younger vs older people: a systematic review and Meta-analysis. JAMA Neurol. 2022;79 10:1036–48. 10.1001/jamaneurol.2022.1520. 35943738 10.1001/jamaneurol.2022.1520 PMC9364236

[25] Andrade CAS, Mahrouseh N, Gabrani J, Charalampous P, Cuschieri S, Grad DA, et al. Inequalities in the burden of non-communicable diseases across European countries: a systematic analysis of the global burden of Disease 2019 study. Int J Equity Health. 2023;22(1:140). 10.1186/s12939-023-01958-8. 10.1186/s12939-023-01958-8 PMC10375608 37507733

[26] Efremova D, Ciolac D, Zota E, Glavan D, Ciobanu N, Aulitzky W, et al. Dissecting the spectrum of stroke risk factors in an apparently healthy Population: paving the Roadmap to Primary Stroke Prevention. J Cardiovasc Dev Dis. 2023;10(2). 10.3390/jcdd10020035. 10.3390/jcdd10020035 PMC9965290 36826531

[27] Petrukhin IS, Lunina EY. Cardiovascular Disease Risk factors and mortality in Russia: challenges and barriers. Public Health Rev. 2011;33 2:436–49. 10.1007/BF03391645.

[28] Chauhan G, Debette S. Genetic risk factors for ischemic and Hemorrhagic Stroke. Curr Cardiol Rep. 2016;18 12:124. 10.1007/s11886-016-0804-z. 27796860 10.1007/s11886-016-0804-z PMC5086478

[29] Jaberinezhad M, Farhoudi M, Nejadghaderi SA, Alizadeh M, Sullman MJM, Carson-Chahhoud K, et al. The burden of stroke and its attributable risk factors in the Middle East and North Africa region, 1990–2019. Sci Rep. 2022;12(1:2700). 10.1038/s41598-022-06418-x. 10.1038/s41598-022-06418-x PMC8854638 35177688

[30] Korv J, Antsov K, Gross-Paju K, Kalju I, Kreis A, Liigant A, et al. Developments in quality of stroke care in Estonia. Eur Stroke J. 2023;8(1 Suppl):35–43. 10.1177/23969873221110745. 36793745 10.1177/23969873221110745 PMC9923126

[31] Nannoni S, de Groot R, Bell S, Markus HS. Stroke in COVID-19: a systematic review and meta-analysis. Int J Stroke. 2021;16(2):137–49. 10.1177/1747493020972922. 33103610 10.1177/1747493020972922 PMC7859578

[32] Norman K, Eriksson M, von Euler M. Sex differences in ischemic stroke within the younger Age Group: a Register-based study. Front Neurol. 2022;13:793181. 10.3389/fneur.2022.793181. 35237226 10.3389/fneur.2022.793181 PMC8882967

[33] Bonkhoff AK, Karch A, Weber R, Wellmann J, Berger K. Female stroke: sex differences in Acute Treatment and early outcomes of Acute ischemic stroke. Stroke. 2021;52(2):406–15. 10.1161/STROKEAHA.120.032850. 33493053 10.1161/STROKEAHA.120.032850

[34] Towfighi A, Stroke, Prevention. Semin Neurol. 2017;37 3:235–6. 10.1055/s-0037-1603947. 10.1055/s-0037-1603947 28759905

[35] Wajngarten M, Silva GS. Hypertension and stroke: update on treatment. Eur Cardiol. 2019;14 2:111–5. 10.15420/ecr.2019.11.1. 31360232 10.15420/ecr.2019.11.1 PMC6659031

[36] Mosenzon O, Cheng AY, Rabinstein AA, Sacco S. Diabetes and stroke: what are the connections? J Stroke. 2023;25 1:26–38. 10.5853/jos.2022.02306. 36592968 10.5853/jos.2022.02306 PMC9911852

[37] Alloubani A, Nimer R, Samara R. Relationship between Hyperlipidemia, Cardiovascular Disease and Stroke: a systematic review. Curr Cardiol Rev. 2021;17 6:e051121189015. 10.2174/1573403X16999201210200342. 33305711 10.2174/1573403X16999201210200342 PMC8950504

