# Supplementary material for: The burden of ischemic stroke in Eastern Europe from 1990 to 2021
Source: BMC Neurol. 2025 Feb 22;25:74. doi: 10.1186/s12883-025-04081-z (PMC11846382; doi:10.1186/s12883-025-04081-z)
Supplement: Supplementary file 1 — Supplementary Material 1 [file 12883_2025_4081_MOESM1_ESM.pdf]

Supplementary Figures:

Supplementary Figure 1. Annual percentage change of age-standardized incidence rates of ischemic stroke in Eastern European Countries from 1990 to 2021.

Supplementary Figure 2. Annual percentage change of age-standardized mortality rates of ischemic stroke in Eastern European Countries from 1990 to 2021.

Supplementary Figure 3. Annual percentage change of age-standardized DALYs rates of ischemic stroke in Eastern European Countries from 1990 to 2021.

Supplementary Figure 4. Dual X-axis plot illustrating the burden of ischemic stroke death attributable to various risk factors (environmental/occupational risks and behavioral risks) in Eastern European Countries.

Supplementary Figure 5. Dual X-axis plot illustrating the burden of ischemic stroke death attributable to four environmental/occupational risk factors in Eastern European Countries.

Supplementary Figure 6. Dual X-axis plot illustrating the burden of ischemic stroke death attributable to eight behavioral risk factors in Eastern European Countries.

A

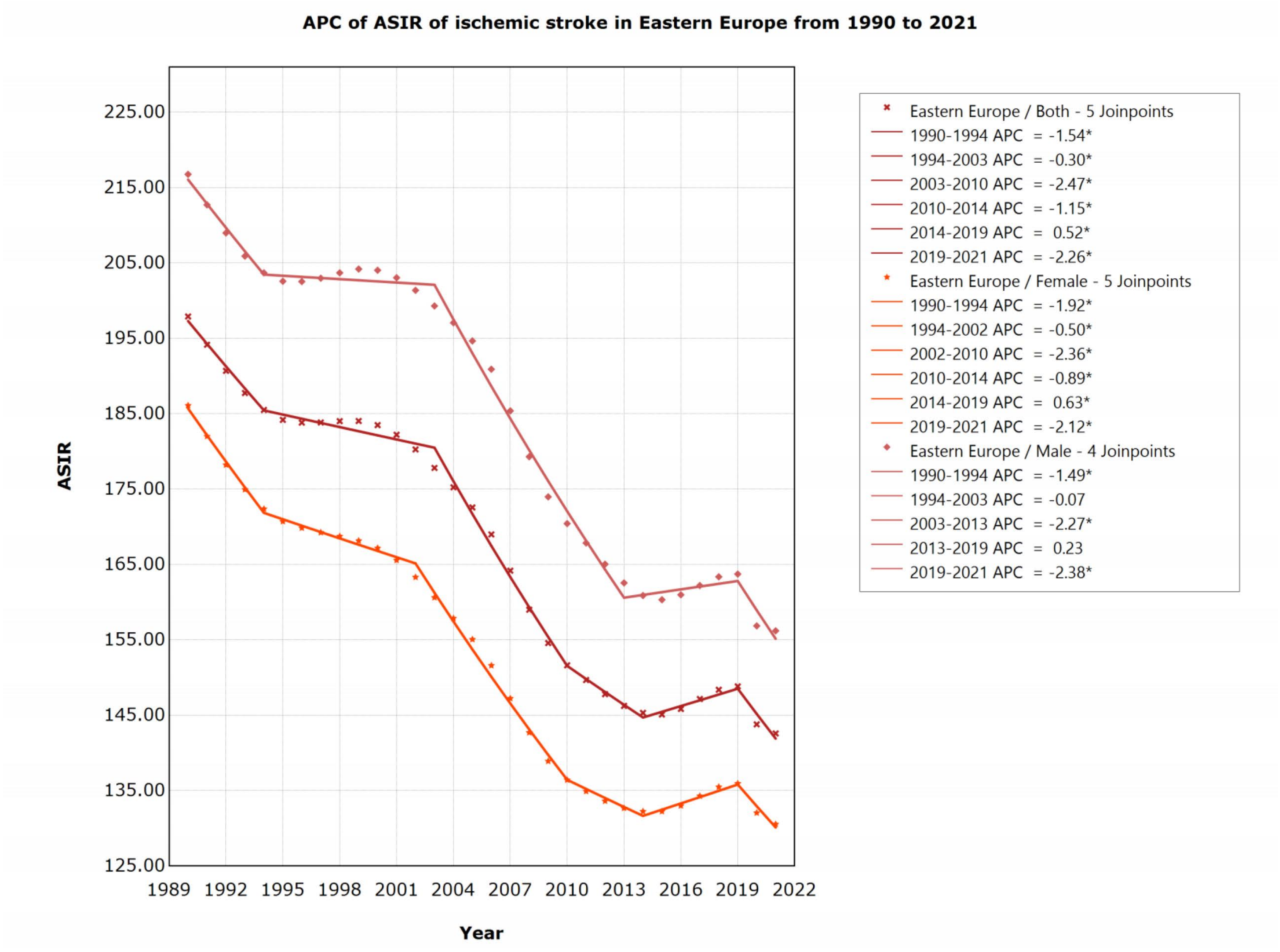

APC of ASIR of ischemic stroke in Eastern Europe from 1990 to 2021
ASIR
225.00
215.00
205.00
195.00
185.00
175.00
165.00
155.00
145.00
135.00
125.00
1989 1992 1995 1998 2001 2004 2007 2010 2013 2016 2019 2022
Year
Eastern Europe / Both - 5 Joinpoints
1990-1994 APC = -1.54*
1994-2003 APC = -0.30*
2003-2010 APC = -2.47*
2010-2014 APC = -1.15*
2014-2019 APC = 0.52*
2019-2021 APC = -2.26*
Eastern Europe / Female - 5 Joinpoints
1990-1994 APC = -1.92*
1994-2002 APC = -0.50*
2002-2010 APC = -2.36*
2010-2014 APC = -0.89*
2014-2019 APC = 0.63*
2019-2021 APC = -2.12*
Eastern Europe / Male - 4 Joinpoints
1990-1994 APC = -1.49*
1994-2003 APC = -0.07
2003-2013 APC = -2.27*
2013-2019 APC = 0.23
2019-2021 APC = -2.38*

B

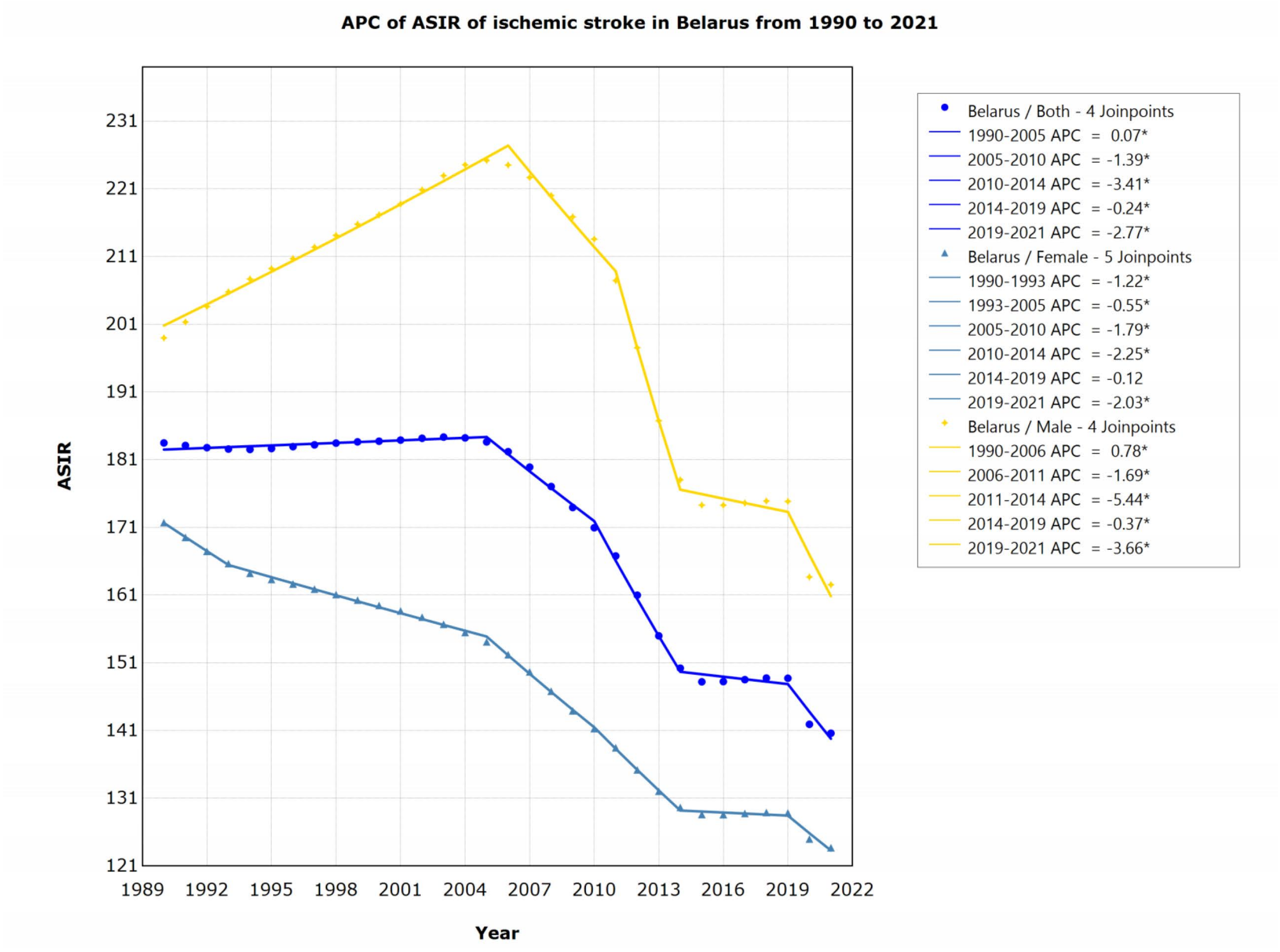
APC of ASIR of ischemic stroke in Belarus from 1990 to 2021
ASIR
Year
231
221
211
201
191
181
171
161
151
141
131
121
1989
1992
1995
1998
2001
2004
2007
2010
2013
2016
2019
2022
Belarus / Both - 4 Joinpoints
1990-2005 APC = 0.07*
2005-2010 APC = -1.39*
2010-2014 APC = -3.41*
2014-2019 APC = -0.24*
2019-2021 APC = -2.77*
Belarus / Female - 5 Joinpoints
1990-1993 APC = -1.22*
1993-2005 APC = -0.55*
2005-2010 APC = -1.79*
2010-2014 APC = -2.25*
2014-2019 APC = -0.12
2019-2021 APC = -2.03*
Belarus / Male - 4 Joinpoints
1990-2006 APC = 0.78*
2006-2011 APC = -1.69*
2011-2014 APC = -5.44*
2014-2019 APC = -0.37*
2019-2021 APC = -3.66*

C

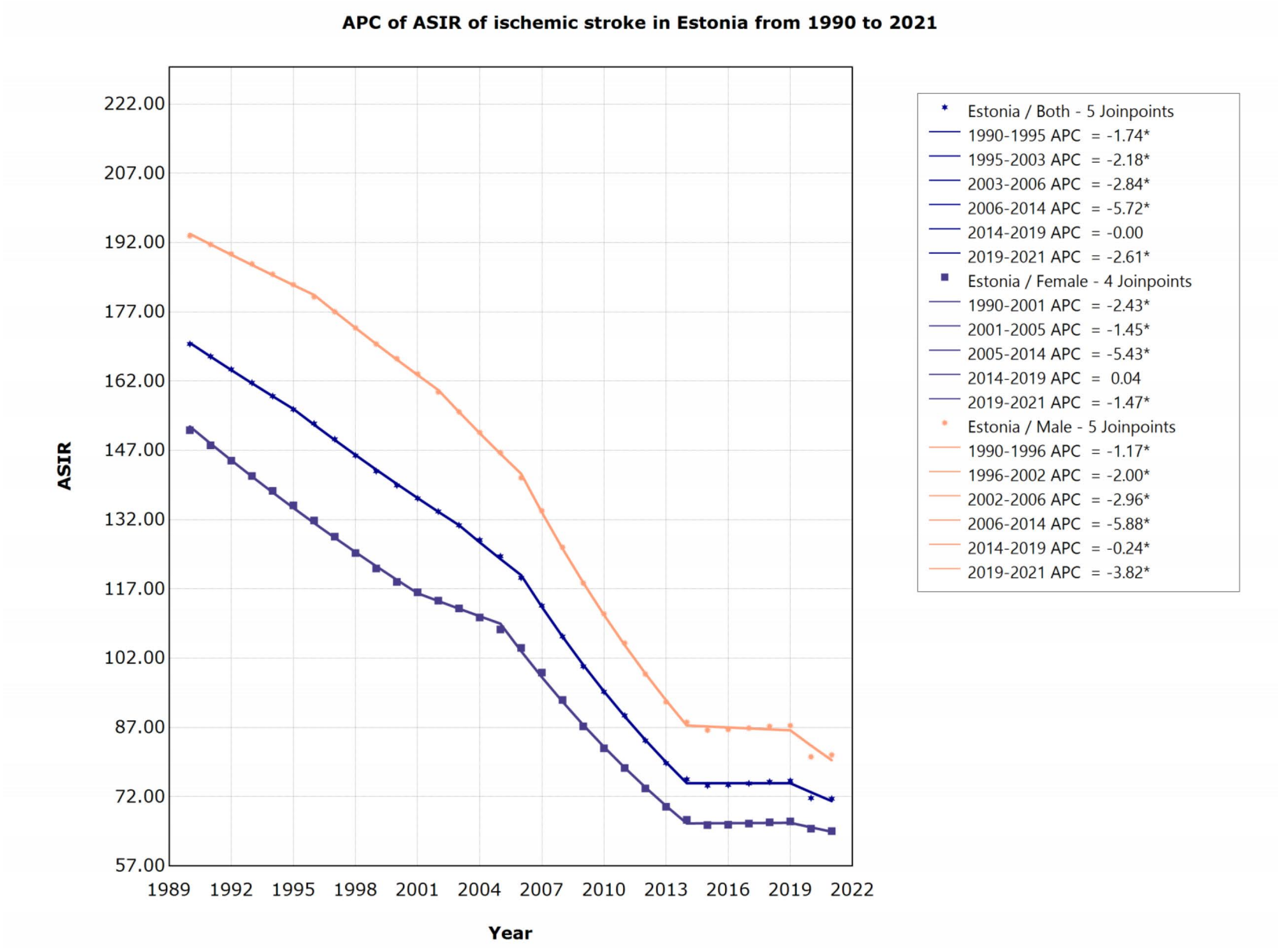

APC of ASIR of ischemic stroke in Estonia from 1990 to 2021
ASIR
222.00
207.00
192.00
177.00
162.00
147.00
132.00
117.00
102.00
87.00
72.00
57.00
1989
1992
1995
1998
2001
2004
2007
2010
2013
2016
2019
2022
Year
Estonia / Both - 5 Joinpoints
1990-1995 APC = -1.74*
1995-2003 APC = -2.18*
2003-2006 APC = -2.84*
2006-2014 APC = -5.72*
2014-2019 APC = -0.00
2019-2021 APC = -2.61*
Estonia / Female - 4 Joinpoints
1990-2001 APC = -2.43*
2001-2005 APC = -1.45*
2005-2014 APC = -5.43*
2014-2019 APC = 0.04
2019-2021 APC = -1.47*
Estonia / Male - 5 Joinpoints
1990-1996 APC = -1.17*
1996-2002 APC = -2.00*
2002-2006 APC = -2.96*
2006-2014 APC = -5.88*
2014-2019 APC = -0.24*
2019-2021 APC = -3.82*

D

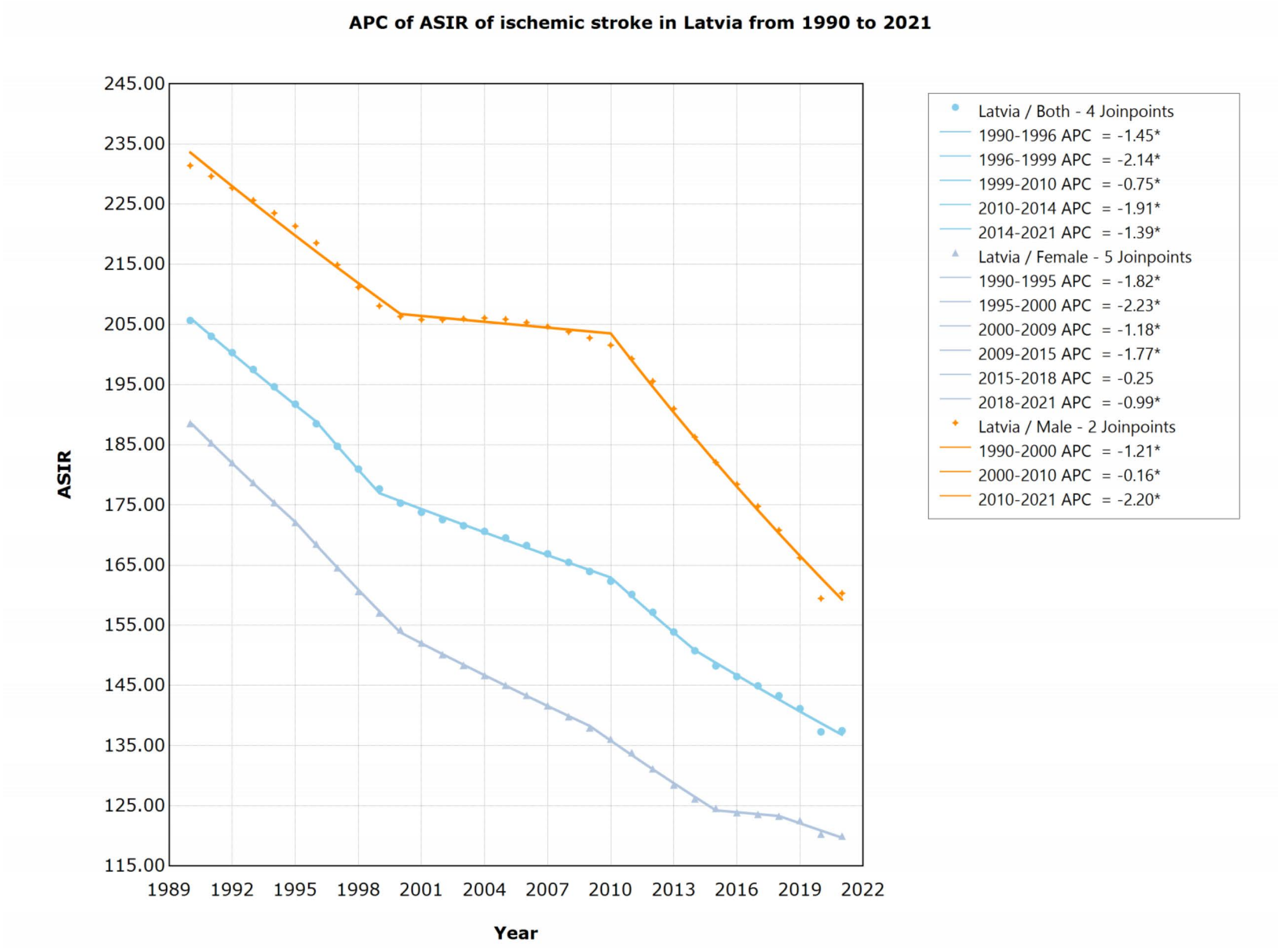
APC of ASIR of ischemic stroke in Latvia from 1990 to 2021
ASIR
Year
245.00
235.00
225.00
215.00
205.00
195.00
185.00
175.00
165.00
155.00
145.00
135.00
125.00
115.00
1989
1992
1995
1998
2001
2004
2007
2010
2013
2016
2019
2022
Latvia / Both - 4 Joinpoints
1990-1996 APC = -1.45*
1996-1999 APC = -2.14*
1999-2010 APC = -0.75*
2010-2014 APC = -1.91*
2014-2021 APC = -1.39*
Latvia / Female - 5 Joinpoints
1990-1995 APC = -1.82*
1995-2000 APC = -2.23*
2000-2009 APC = -1.18*
2009-2015 APC = -1.77*
2015-2018 APC = -0.25
2018-2021 APC = -0.99*
Latvia / Male - 2 Joinpoints
1990-2000 APC = -1.21*
2000-2010 APC = -0.16*
2010-2021 APC = -2.20*

E

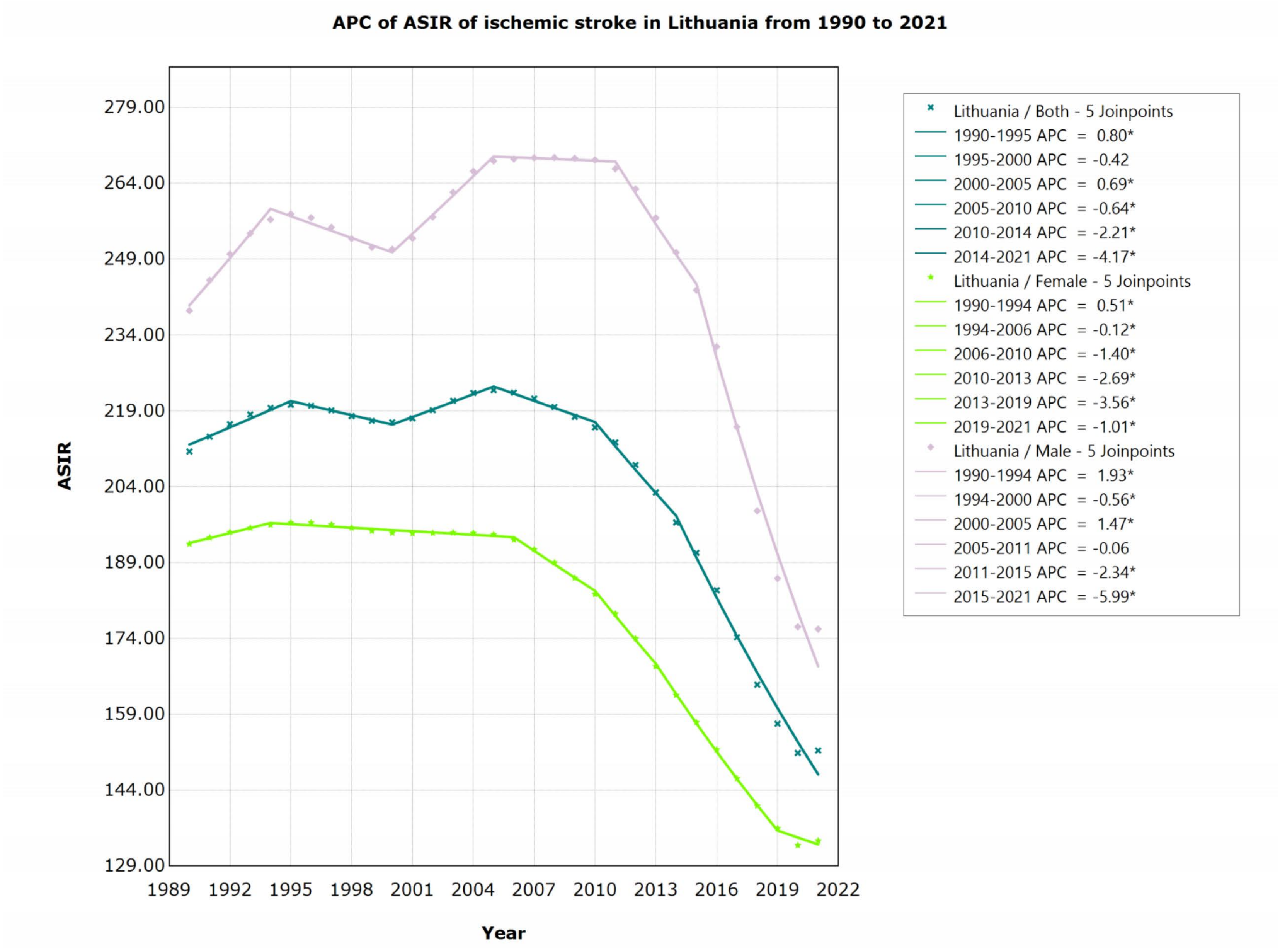
APC of ASIR of ischemic stroke in Lithuania from 1990 to 2021
279.00
264.00
249.00
234.00
219.00
204.00
189.00
174.00
159.00
144.00
129.00
ASIR
1989
1992
1995
1998
2001
2004
2007
2010
2013
2016
2019
2022
Year
Lithuania / Both - 5 Joinpoints
1990-1995 APC = 0.80*
1995-2000 APC = -0.42
2000-2005 APC = 0.69*
2005-2010 APC = -0.64*
2010-2014 APC = -2.21*
2014-2021 APC = -4.17*
Lithuania / Female - 5 Joinpoints
1990-1994 APC = 0.51*
1994-2006 APC = -0.12*
2006-2010 APC = -1.40*
2010-2013 APC = -2.69*
2013-2019 APC = -3.56*
2019-2021 APC = -1.01*
Lithuania / Male - 5 Joinpoints
1990-1994 APC = 1.93*
1994-2000 APC = -0.56*
2000-2005 APC = 1.47*
2005-2011 APC = -0.06
2011-2015 APC = -2.34*
2015-2021 APC = -5.99*

F

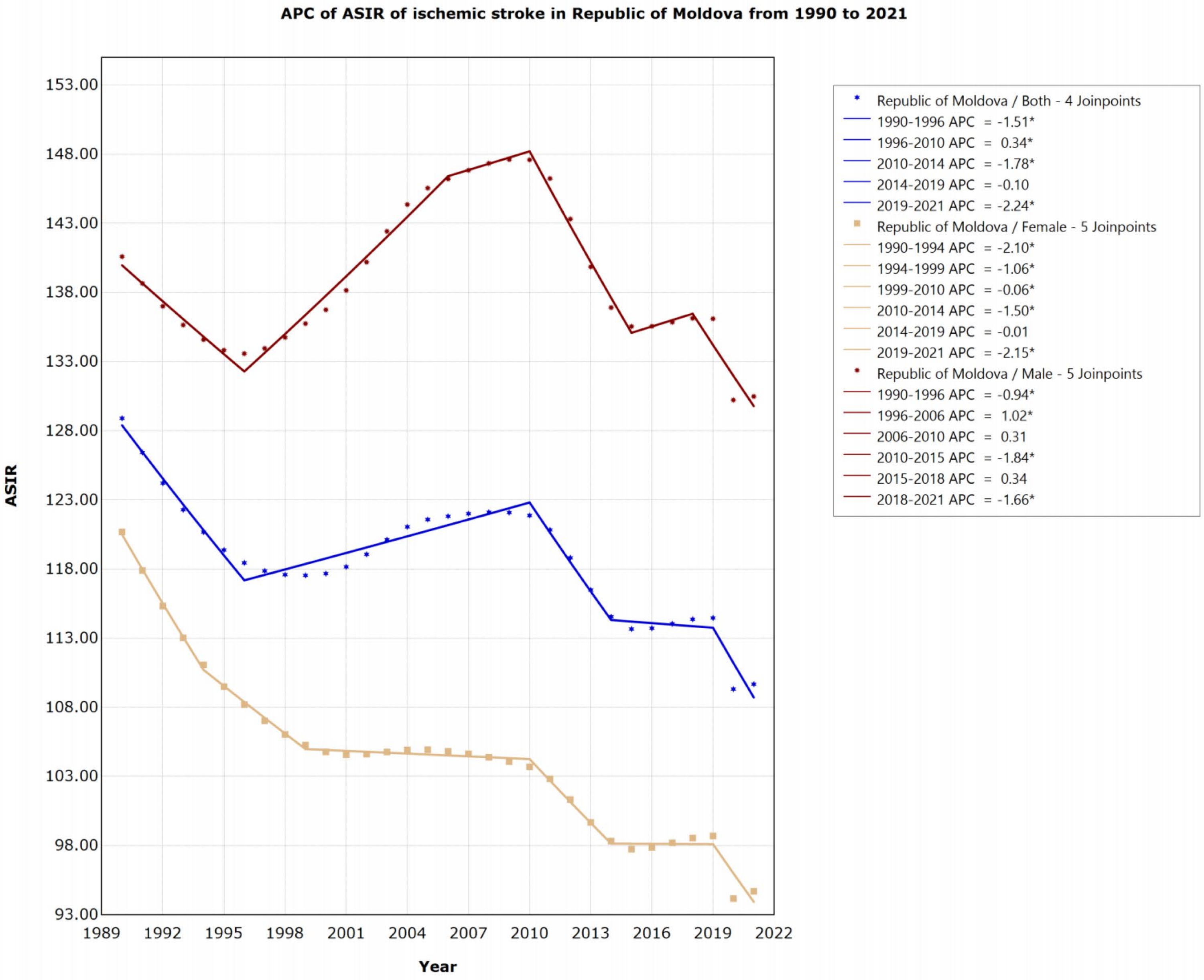
APC of ASIR of ischemic stroke in Republic of Moldova from 1990 to 2021
ASIR
Year
153.00
148.00
143.00
138.00
133.00
128.00
123.00
118.00
113.00
108.00
103.00
98.00
93.00
1989
1992
1995
1998
2001
2004
2007
2010
2013
2016
2019
2022
Republic of Moldova / Both - 4 Joinpoints
1990-1996 APC = -1.51*
1996-2010 APC = 0.34*
2010-2014 APC = -1.78*
2014-2019 APC = -0.10
2019-2021 APC = -2.24*
Republic of Moldova / Female - 5 Joinpoints
1990-1994 APC = -2.10*
1994-1999 APC = -1.06*
1999-2010 APC = -0.06*
2010-2014 APC = -1.50*
2014-2019 APC = -0.01
2019-2021 APC = -2.15*
Republic of Moldova / Male - 5 Joinpoints
1990-1996 APC = -0.94*
1996-2006 APC = 1.02*
2006-2010 APC = 0.31
2010-2015 APC = -1.84*
2015-2018 APC = 0.34
2018-2021 APC = -1.66*

G

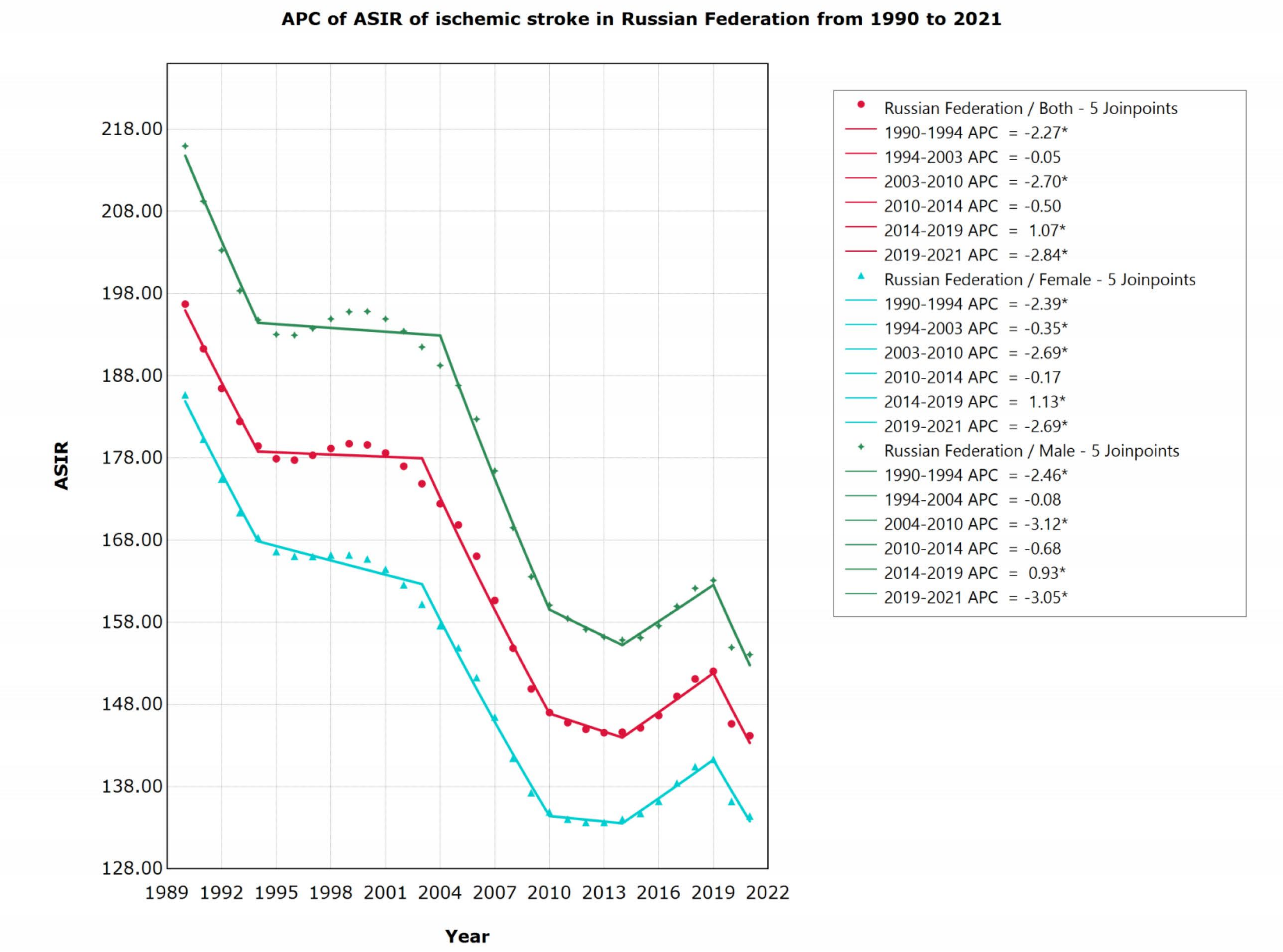
APC of ASIR of ischemic stroke in Russian Federation from 1990 to 2021
ASIR
218.00
208.00
198.00
188.00
178.00
168.00
158.00
148.00
138.00
128.00
1989 1992 1995 1998 2001 2004 2007 2010 2013 2016 2019 2022
Year
Russian Federation / Both - 5 Joinpoints
1990-1994 APC = -2.27*
1994-2003 APC = -0.05
2003-2010 APC = -2.70*
2010-2014 APC = -0.50
2014-2019 APC = 1.07*
2019-2021 APC = -2.84*
Russian Federation / Female - 5 Joinpoints
1990-1994 APC = -2.39*
1994-2003 APC = -0.35*
2003-2010 APC = -2.69*
2010-2014 APC = -0.17
2014-2019 APC = 1.13*
2019-2021 APC = -2.69*
Russian Federation / Male - 5 Joinpoints
1990-1994 APC = -2.46*
1994-2004 APC = -0.08
2004-2010 APC = -3.12*
2010-2014 APC = -0.68
2014-2019 APC = 0.93*
2019-2021 APC = -3.05*

H

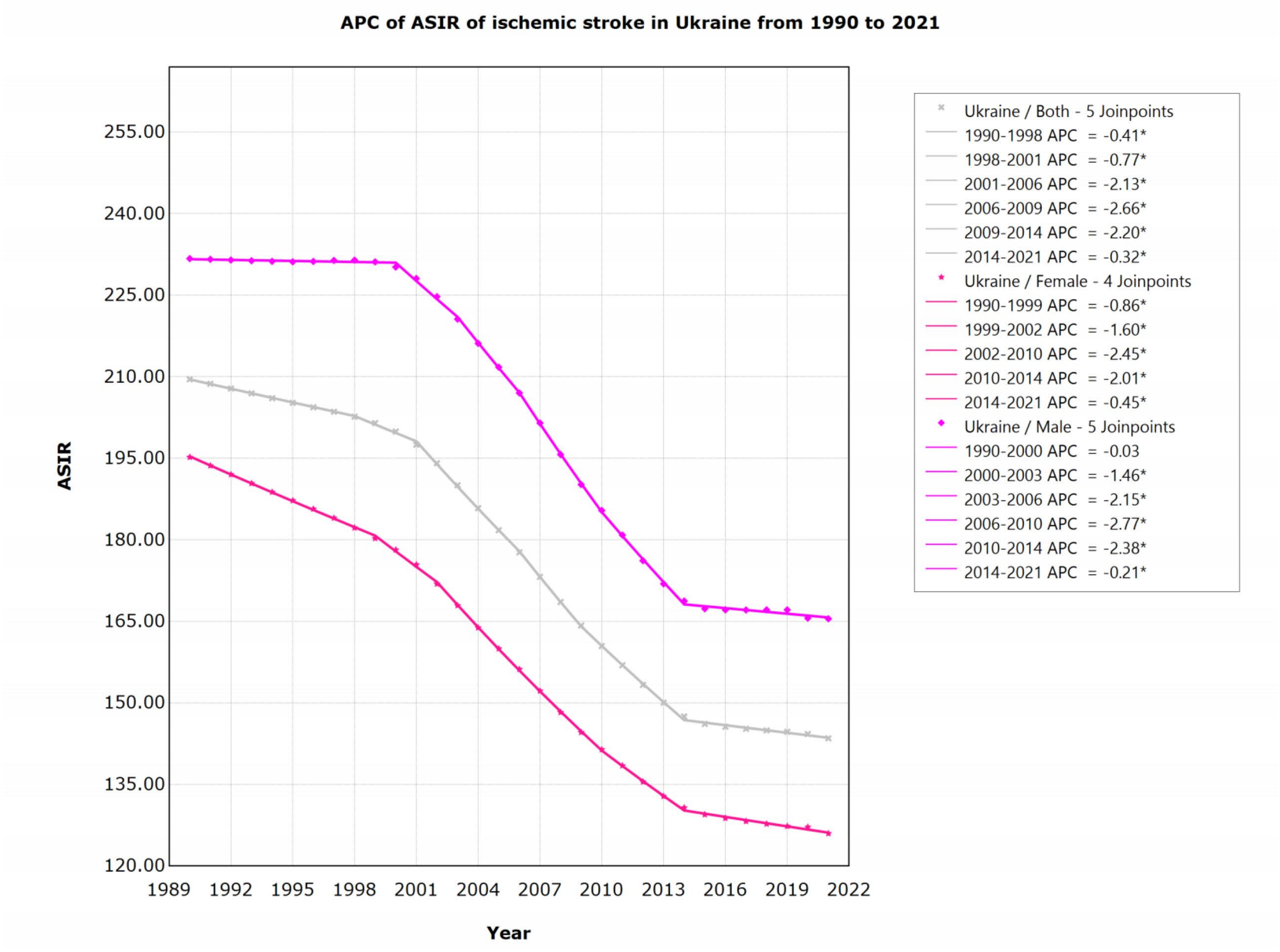

APC of ASIR of ischemic stroke in Ukraine from 1990 to 2021
ASIR
255.00
240.00
225.00
210.00
195.00
180.00
165.00
150.00
135.00
120.00
1989
1992
1995
1998
2001
2004
2007
2010
2013
2016
2019
2022
Year
Ukraine / Both - 5 Joinpoints
1990-1998 APC = -0.41*
1998-2001 APC = -0.77*
2001-2006 APC = -2.13*
2006-2009 APC = -2.66*
2009-2014 APC = -2.20*
2014-2021 APC = -0.32*
Ukraine / Female - 4 Joinpoints
1990-1999 APC = -0.86*
1999-2002 APC = -1.60*
2002-2010 APC = -2.45*
2010-2014 APC = -2.01*
2014-2021 APC = -0.45*
Ukraine / Male - 5 Joinpoints
1990-2000 APC = -0.03
2000-2003 APC = -1.46*
2003-2006 APC = -2.15*
2006-2010 APC = -2.77*
2010-2014 APC = -2.38*
2014-2021 APC = -0.21*

Supplementary Figure 1. Annual percentage change of age-standardized incidence rates of ischemic stroke in Eastern European Countries from 1990 to 2021: A. APC of ASIR of ischemic stroke in Eastern Europe from 1990 to 2021; B. APC of ASIR of ischemic stroke in Belarus from 1990 to 2021; C. APC of ASIR of ischemic stroke in Estonia from 1990 to 2021; D. APC of ASIR of ischemic stroke in Latvia from 1990 to 2021; E. APC of ASIR of ischemic stroke in Lithuania from 1990 to 2021; F. APC of ASIR of ischemic stroke in Republic of Moldova from 1990 to 2021; G. APC of ASIR of ischemic stroke in Russian Federation from 1990 to 2021; H. APC of ASIR of ischemic stroke in Ukraine from 1990 to 2021. APC, Annual percentage change; ASIR, age-standardized incidence rate.

A

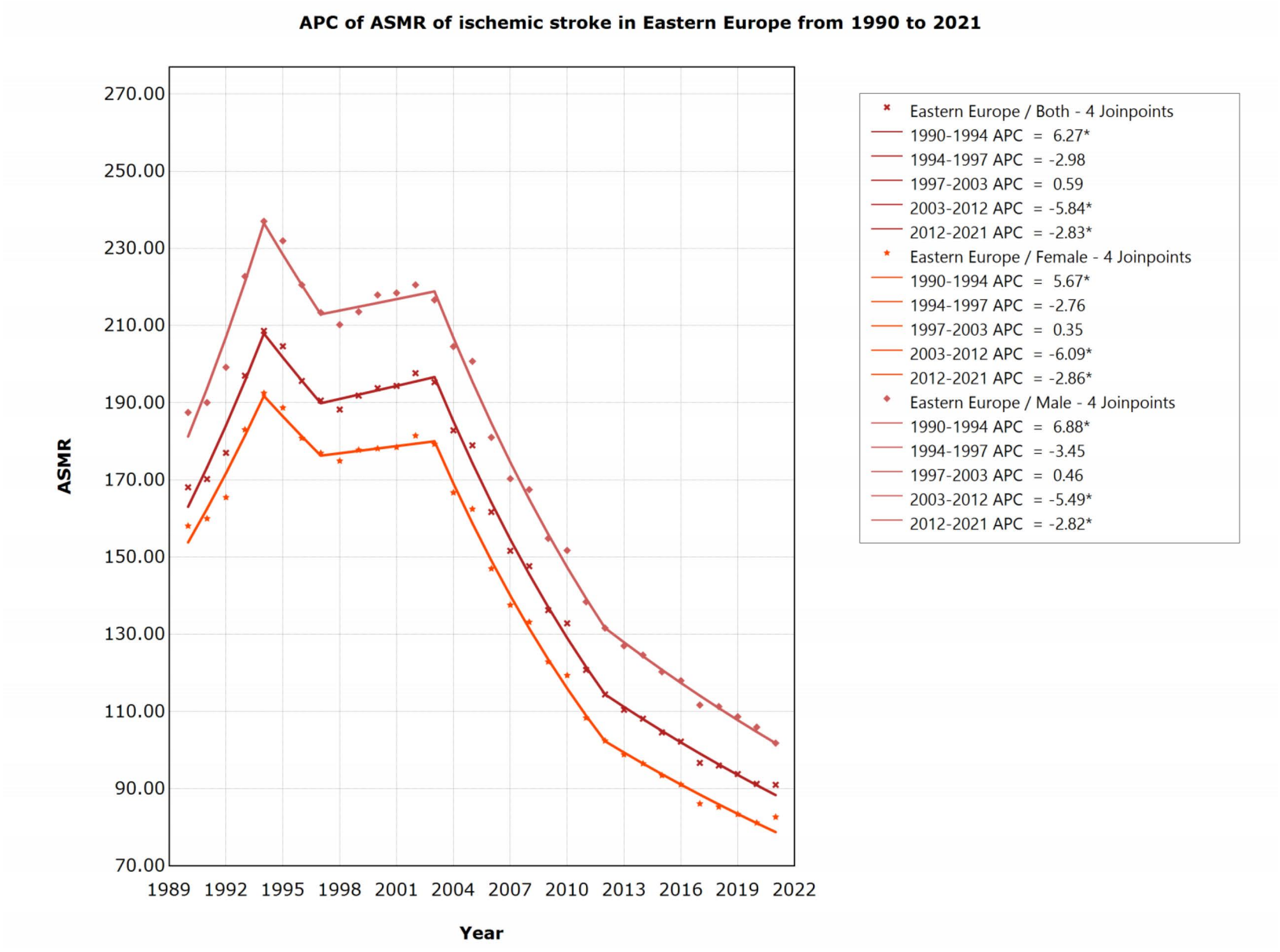
APC of ASMR of ischemic stroke in Eastern Europe from 1990 to 2021
ASMR
270.00
250.00
230.00
210.00
190.00
170.00
150.00
130.00
110.00
90.00
70.00
1989
1992
1995
1998
2001
2004
2007
2010
2013
2016
2019
2022
Year
Eastern Europe / Both - 4 Joinpoints
1990-1994 APC = 6.27*
1994-1997 APC = -2.98
1997-2003 APC = 0.59
2003-2012 APC = -5.84*
2012-2021 APC = -2.83*
Eastern Europe / Female - 4 Joinpoints
1990-1994 APC = 5.67*
1994-1997 APC = -2.76
1997-2003 APC = 0.35
2003-2012 APC = -6.09*
2012-2021 APC = -2.86*
Eastern Europe / Male - 4 Joinpoints
1990-1994 APC = 6.88*
1994-1997 APC = -3.45
1997-2003 APC = 0.46
2003-2012 APC = -5.49*
2012-2021 APC = -2.82*

B

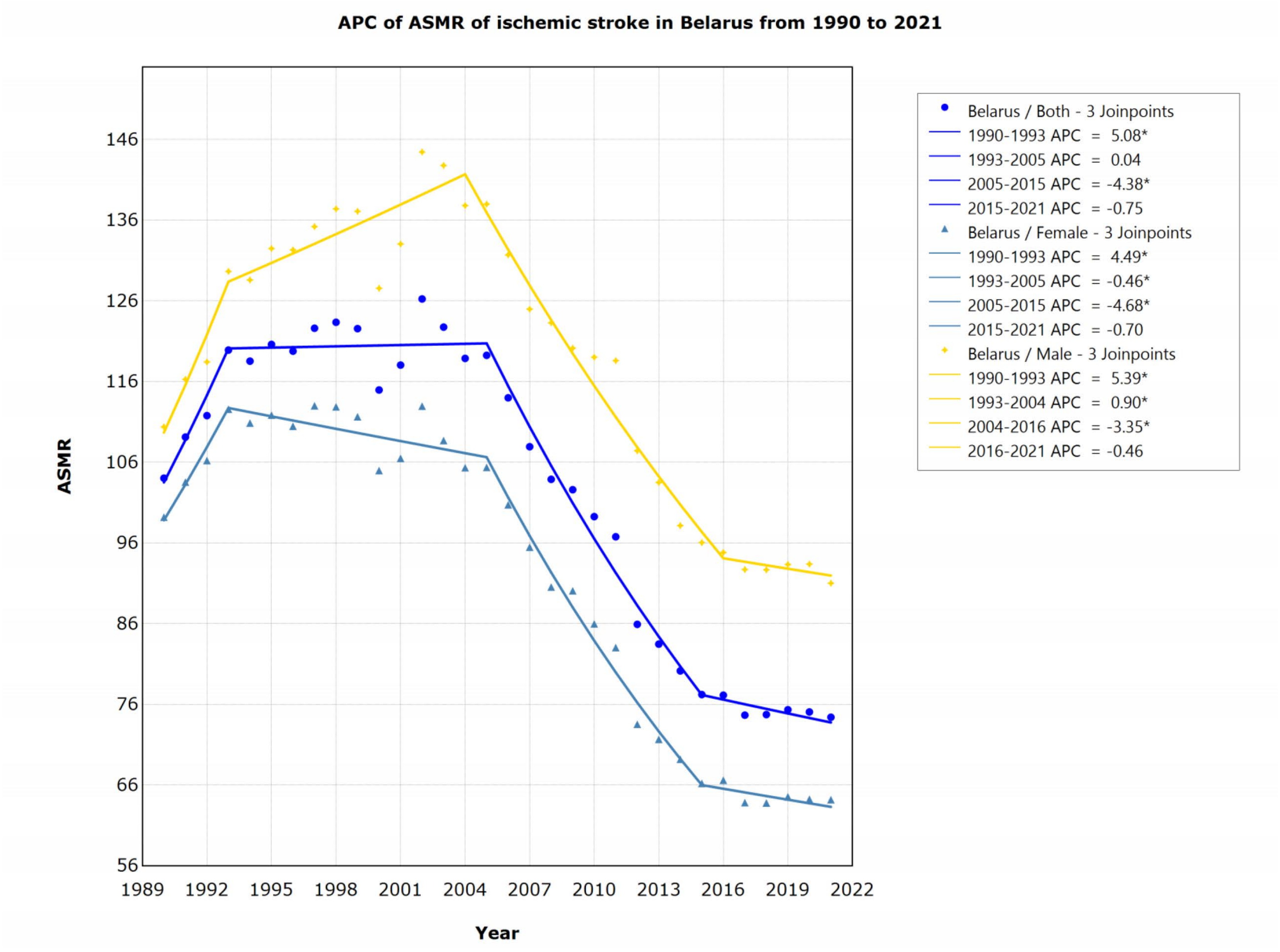
APC of ASMR of ischemic stroke in Belarus from 1990 to 2021
ASMR
146
136
126
116
106
96
86
76
66
56
1989
1992
1995
1998
2001
2004
2007
2010
2013
2016
2019
2022
Year
Belarus / Both - 3 Joinpoints
1990-1993 APC = 5.08*
1993-2005 APC = 0.04
2005-2015 APC = -4.38*
2015-2021 APC = -0.75
Belarus / Female - 3 Joinpoints
1990-1993 APC = 4.49*
1993-2005 APC = -0.46*
2005-2015 APC = -4.68*
2015-2021 APC = -0.70
Belarus / Male - 3 Joinpoints
1990-1993 APC = 5.39*
1993-2004 APC = 0.90*
2004-2016 APC = -3.35*
2016-2021 APC = -0.46

C

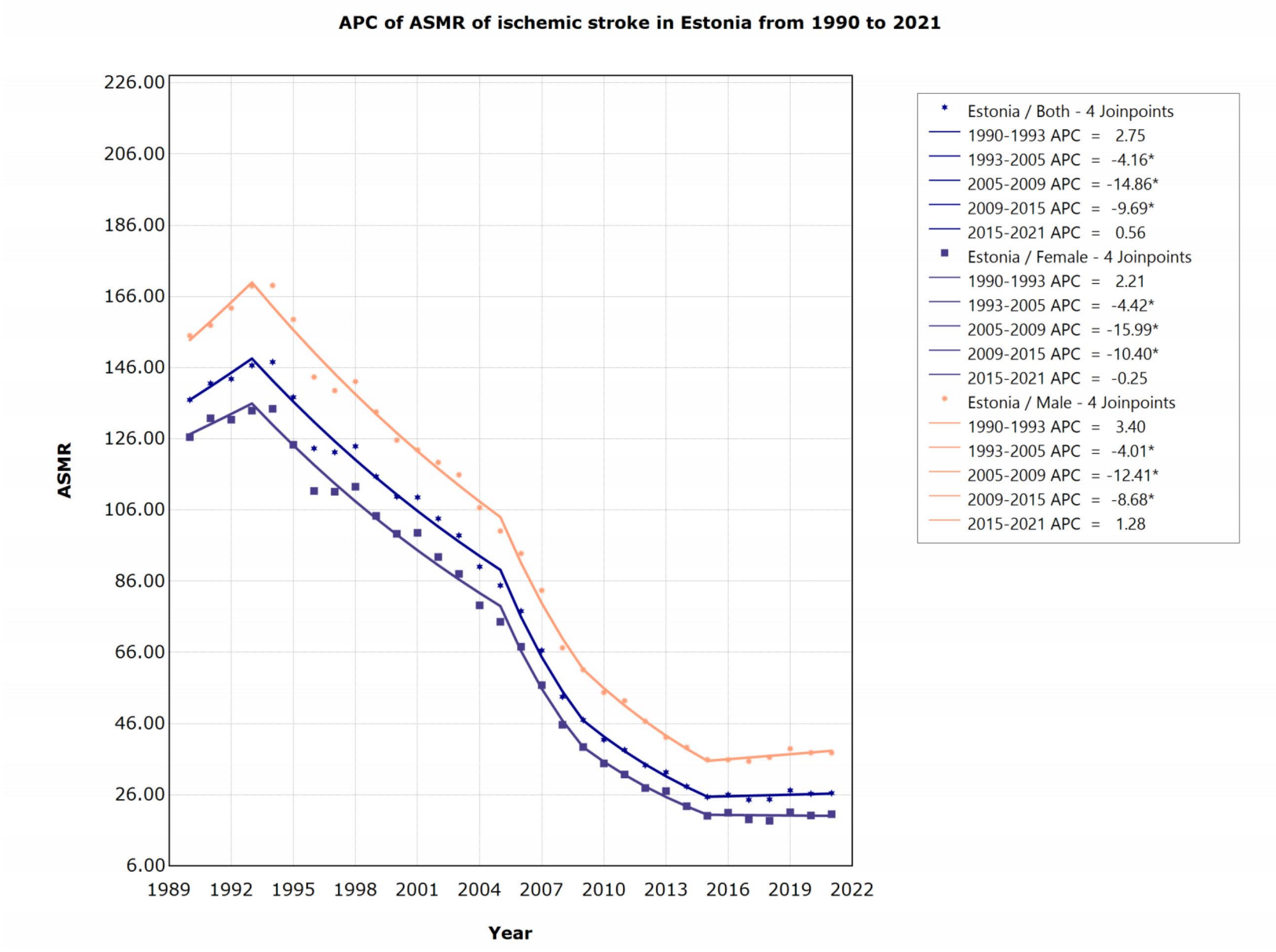

APC of ASMR of ischemic stroke in Estonia from 1990 to 2021
ASMR
Year
226.00
206.00
186.00
166.00
146.00
126.00
106.00
86.00
66.00
46.00
26.00
6.00
1989
1992
1995
1998
2001
2004
2007
2010
2013
2016
2019
2022
Estonia / Both - 4 Joinpoints
1990-1993 APC = 2.75
1993-2005 APC = -4.16*
2005-2009 APC = -14.86*
2009-2015 APC = -9.69*
2015-2021 APC = 0.56
Estonia / Female - 4 Joinpoints
1990-1993 APC = 2.21
1993-2005 APC = -4.42*
2005-2009 APC = -15.99*
2009-2015 APC = -10.40*
2015-2021 APC = -0.25
Estonia / Male - 4 Joinpoints
1990-1993 APC = 3.40
1993-2005 APC = -4.01*
2005-2009 APC = -12.41*
2009-2015 APC = -8.68*
2015-2021 APC = 1.28

D

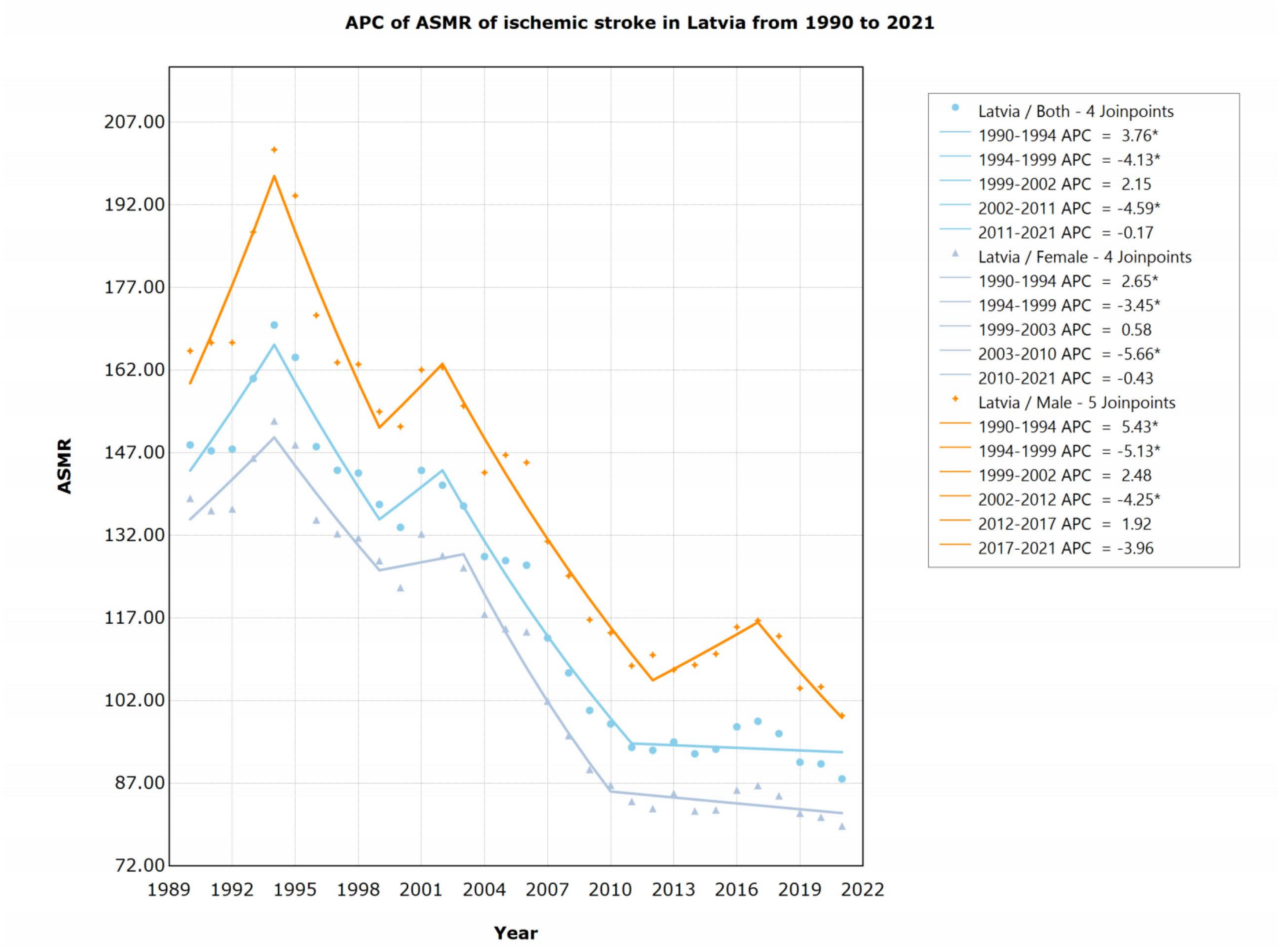
APC of ASMR of ischemic stroke in Latvia from 1990 to 2021
ASMR
Year
207.00
192.00
177.00
162.00
147.00
132.00
117.00
102.00
87.00
72.00
1989
1992
1995
1998
2001
2004
2007
2010
2013
2016
2019
2022
Latvia / Both - 4 Joinpoints
1990-1994 APC = 3.76*
1994-1999 APC = -4.13*
1999-2002 APC = 2.15
2002-2011 APC = -4.59*
2011-2021 APC = -0.17
Latvia / Female - 4 Joinpoints
1990-1994 APC = 2.65*
1994-1999 APC = -3.45*
1999-2003 APC = 0.58
2003-2010 APC = -5.66*
2010-2021 APC = -0.43
Latvia / Male - 5 Joinpoints
1990-1994 APC = 5.43*
1994-1999 APC = -5.13*
1999-2002 APC = 2.48
2002-2012 APC = -4.25*
2012-2017 APC = 1.92
2017-2021 APC = -3.96

E

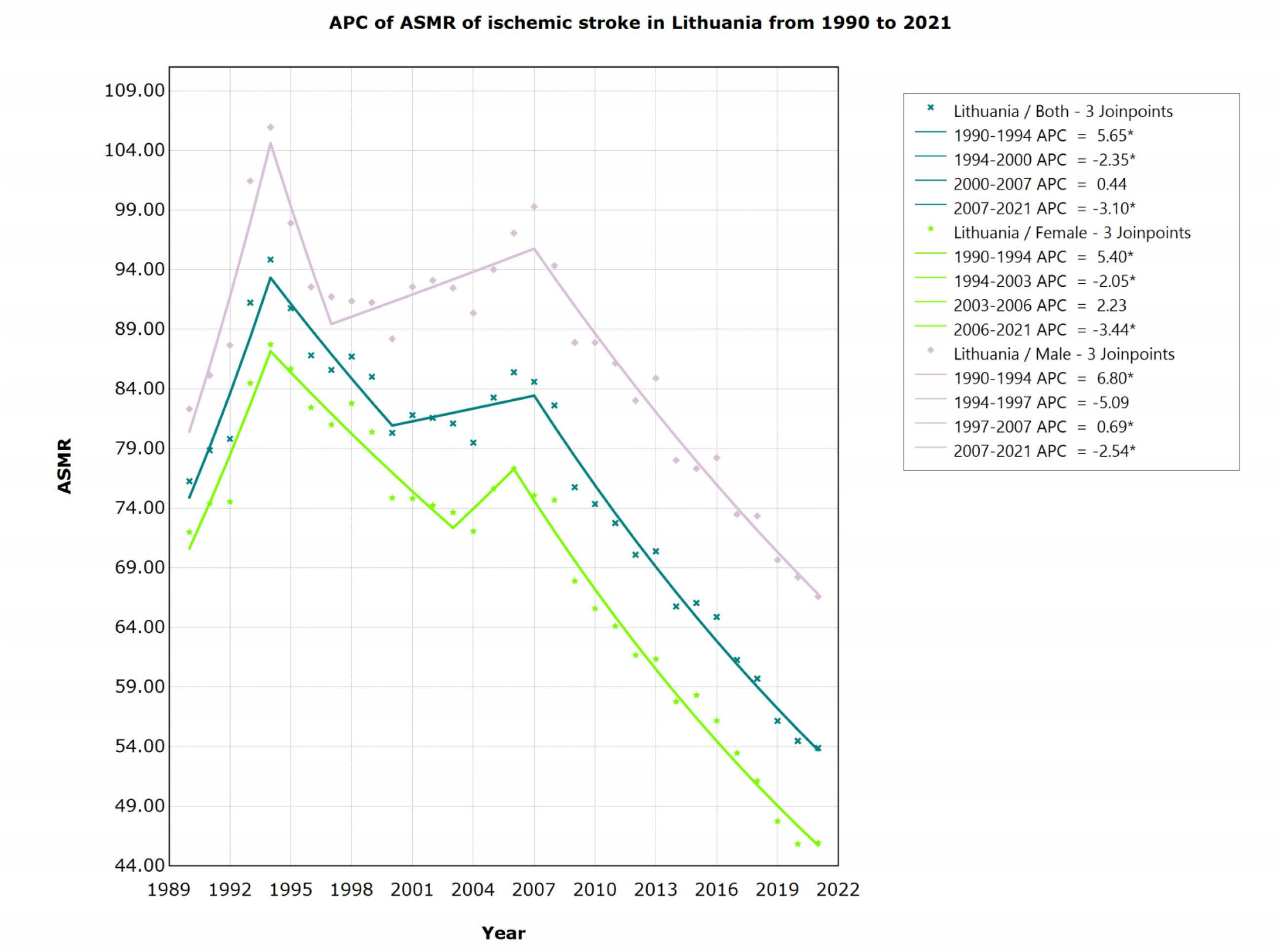
APC of ASMR of ischemic stroke in Lithuania from 1990 to 2021
ASMR
Year
109.00
104.00
99.00
94.00
89.00
84.00
79.00
74.00
69.00
64.00
59.00
54.00
49.00
44.00
1989
1992
1995
1998
2001
2004
2007
2010
2013
2016
2019
2022
Lithuania / Both - 3 Joinpoints
1990-1994 APC = 5.65*
1994-2000 APC = -2.35*
2000-2007 APC = 0.44
2007-2021 APC = -3.10*
Lithuania / Female - 3 Joinpoints
1990-1994 APC = 5.40*
1994-2003 APC = -2.05*
2003-2006 APC = 2.23
2006-2021 APC = -3.44*
Lithuania / Male - 3 Joinpoints
1990-1994 APC = 6.80*
1994-1997 APC = -5.09
1997-2007 APC = 0.69*
2007-2021 APC = -2.54*

F

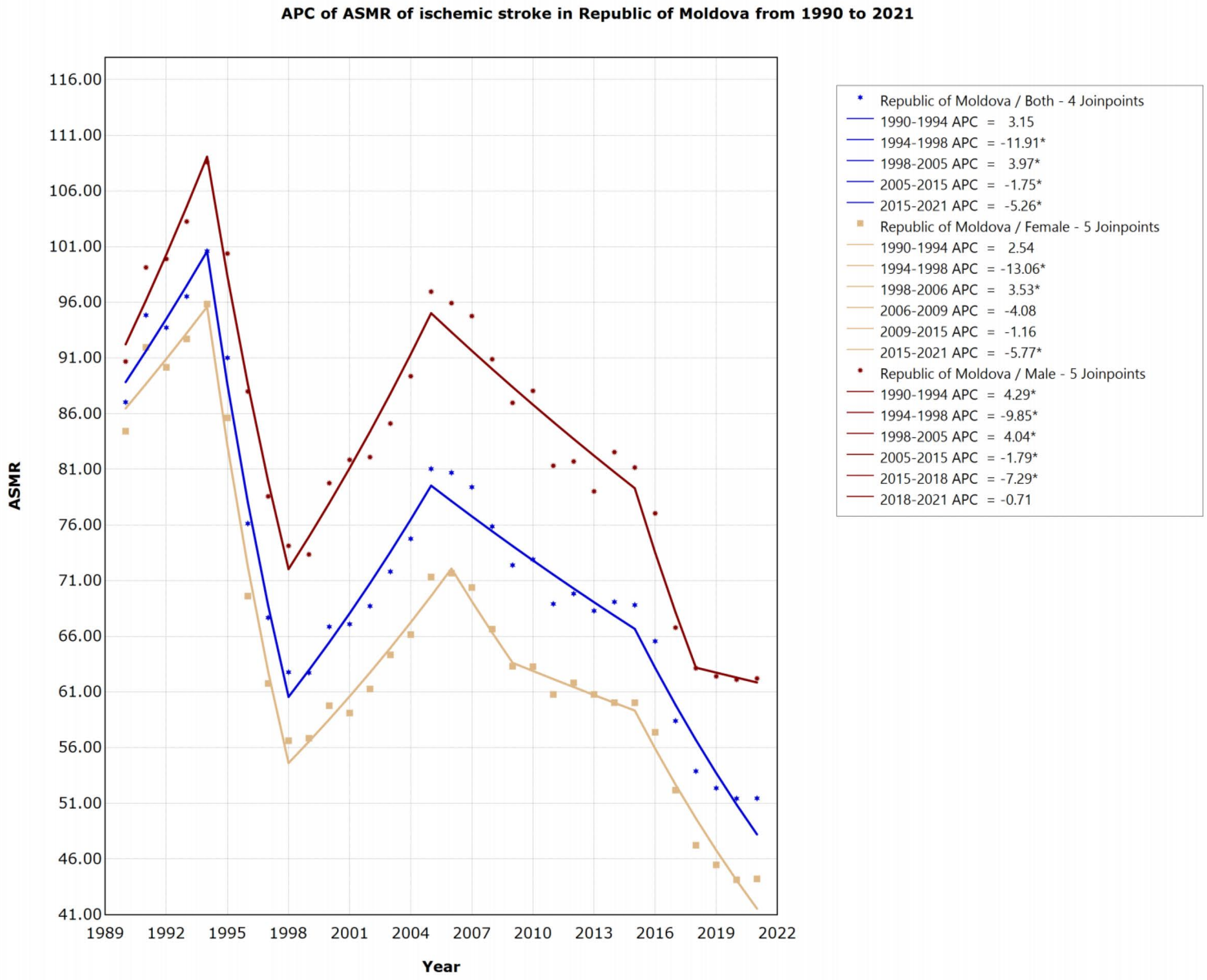

APC of ASMR of ischemic stroke in Republic of Moldova from 1990 to 2021
ASMR
Year
116.00
111.00
106.00
101.00
96.00
91.00
86.00
81.00
76.00
71.00
66.00
61.00
56.00
51.00
46.00
41.00
1989
1992
1995
1998
2001
2004
2007
2010
2013
2016
2019
2022
Republic of Moldova / Both - 4 Joinpoints
1990-1994 APC = 3.15
1994-1998 APC = -11.91*
1998-2005 APC = 3.97*
2005-2015 APC = -1.75*
2015-2021 APC = -5.26*
Republic of Moldova / Female - 5 Joinpoints
1990-1994 APC = 2.54
1994-1998 APC = -13.06*
1998-2006 APC = 3.53*
2006-2009 APC = -4.08
2009-2015 APC = -1.16
2015-2021 APC = -5.77*
Republic of Moldova / Male - 5 Joinpoints
1990-1994 APC = 4.29*
1994-1998 APC = -9.85*
1998-2005 APC = 4.04*
2005-2015 APC = -1.79*
2015-2018 APC = -7.29*
2018-2021 APC = -0.71

G

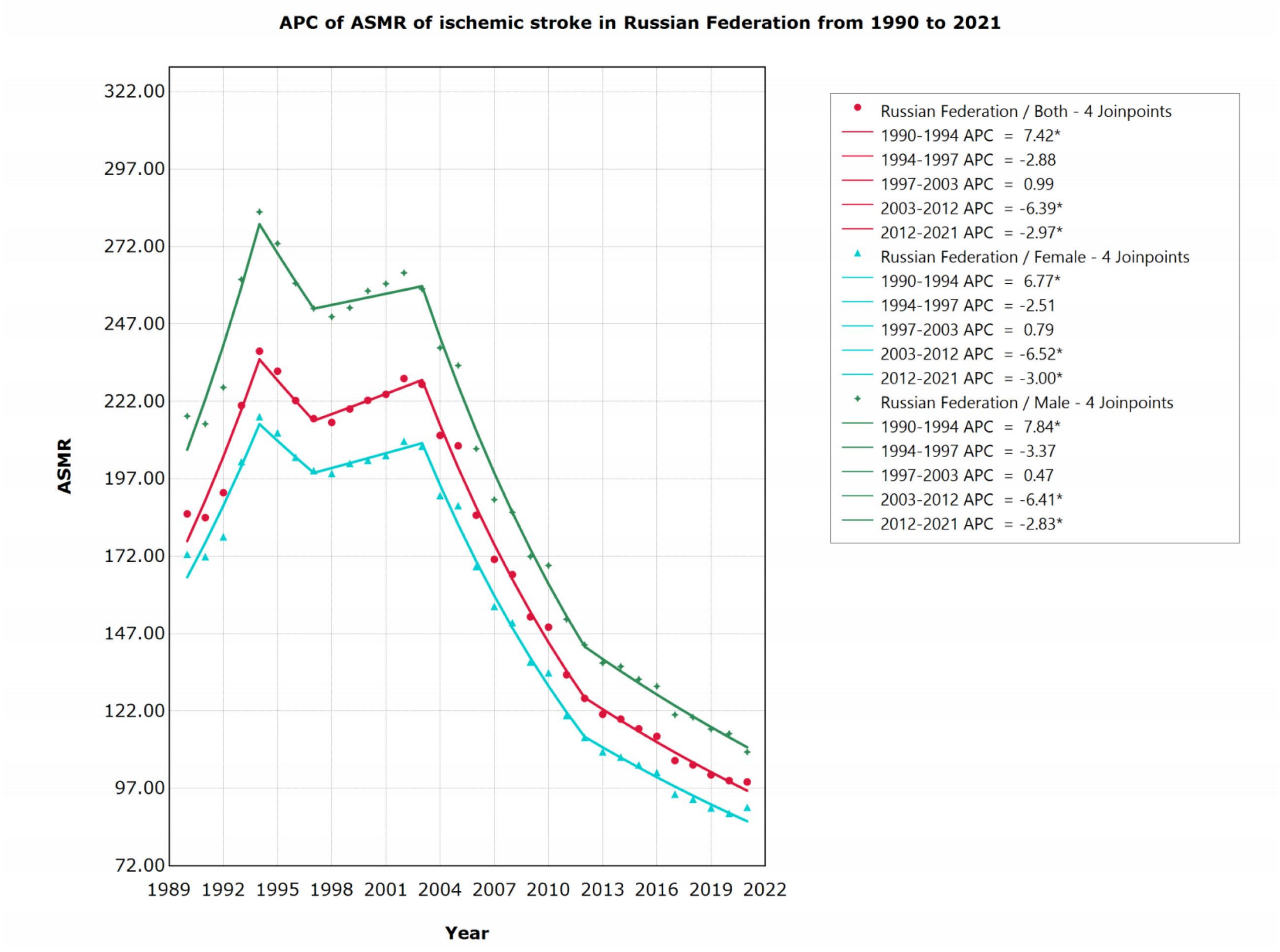

APC of ASMR of ischemic stroke in Russian Federation from 1990 to 2021
ASMR
322.00
297.00
272.00
247.00
222.00
197.00
172.00
147.00
122.00
97.00
72.00
1989 1992 1995 1998 2001 2004 2007 2010 2013 2016 2019 2022
Year
Russian Federation / Both - 4 Joinpoints
1990-1994 APC = 7.42*
1994-1997 APC = -2.88
1997-2003 APC = 0.99
2003-2012 APC = -6.39*
2012-2021 APC = -2.97*
Russian Federation / Female - 4 Joinpoints
1990-1994 APC = 6.77*
1994-1997 APC = -2.51
1997-2003 APC = 0.79
2003-2012 APC = -6.52*
2012-2021 APC = -3.00*
Russian Federation / Male - 4 Joinpoints
1990-1994 APC = 7.84*
1994-1997 APC = -3.37
1997-2003 APC = 0.47
2003-2012 APC = -6.41*
2012-2021 APC = -2.83*

H

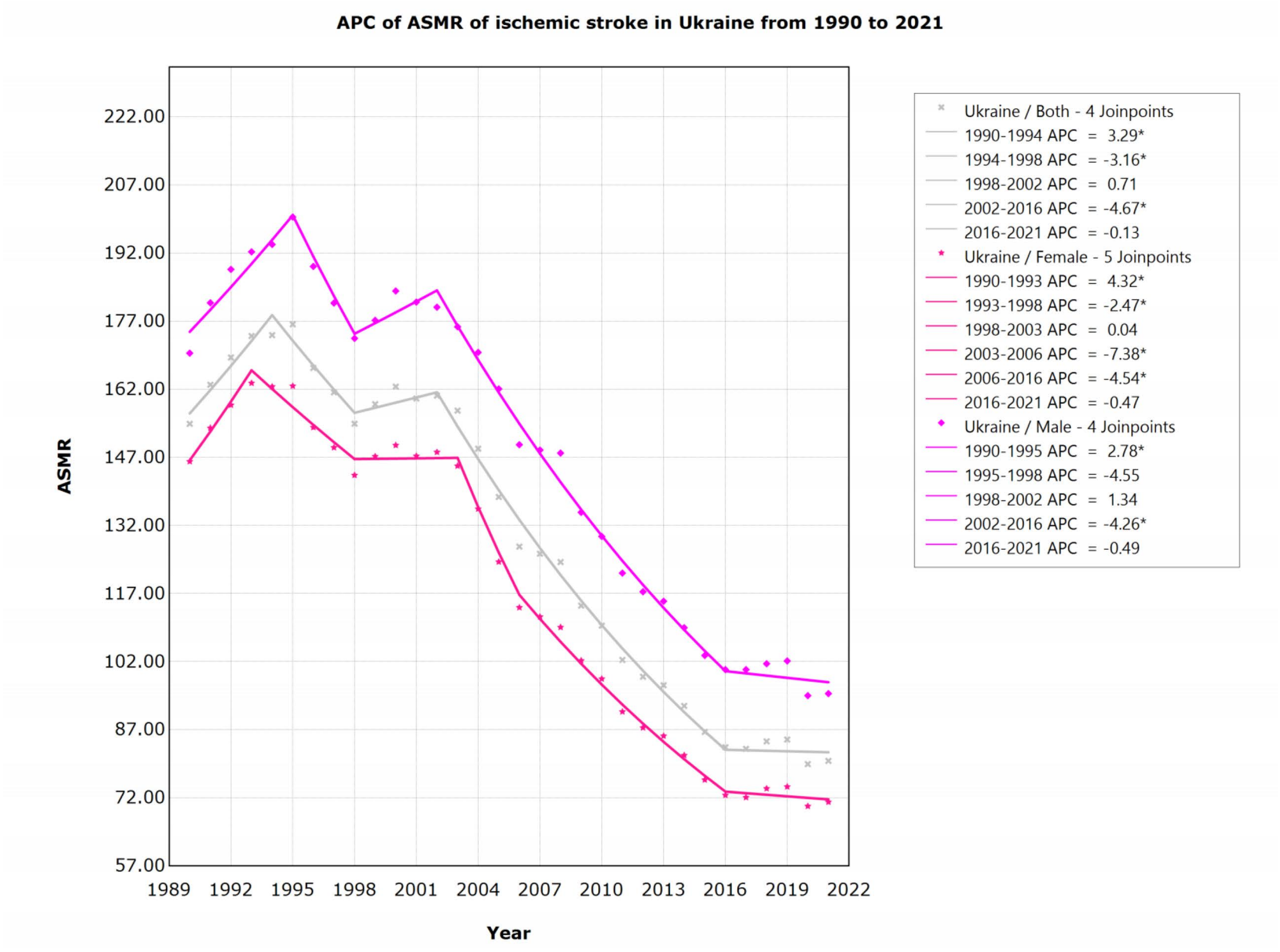

APC of ASMR of ischemic stroke in Ukraine from 1990 to 2021
ASMR
222.00
207.00
192.00
177.00
162.00
147.00
132.00
117.00
102.00
87.00
72.00
57.00
1989
1992
1995
1998
2001
2004
2007
2010
2013
2016
2019
2022
Year
Ukraine / Both - 4 Joinpoints
1990-1994 APC = 3.29*
1994-1998 APC = -3.16*
1998-2002 APC = 0.71
2002-2016 APC = -4.67*
2016-2021 APC = -0.13
Ukraine / Female - 5 Joinpoints
1990-1993 APC = 4.32*
1993-1998 APC = -2.47*
1998-2003 APC = 0.04
2003-2006 APC = -7.38*
2006-2016 APC = -4.54*
2016-2021 APC = -0.47
Ukraine / Male - 4 Joinpoints
1990-1995 APC = 2.78*
1995-1998 APC = -4.55
1998-2002 APC = 1.34
2002-2016 APC = -4.26*
2016-2021 APC = -0.49

Supplementary Figure 2. Annual percentage change of age-standardized mortality rates of ischemic stroke in Eastern European Countries from 1990 to 2021: A. APC of ASMR of ischemic stroke in Eastern Europe from 1990 to 2021; B. APC of ASMR of ischemic stroke in Belarus from 1990 to 2021; C. APC of ASMR of ischemic stroke in Estonia from 1990 to 2021; D. APC of ASMR of ischemic stroke in Latvia from 1990 to 2021; E. APC of ASMR of ischemic stroke in Lithuania from 1990 to 2021; F. APC of ASMR of ischemic stroke in Republic of Moldova from 1990 to 2021; G. APC of ASMR of ischemic stroke in Russian Federation from 1990 to 2021; H. APC of ASMR of ischemic stroke in Ukraine from 1990 to 2021. APC, Annual percentage change; ASMR, age-standardized mortality rate.

A

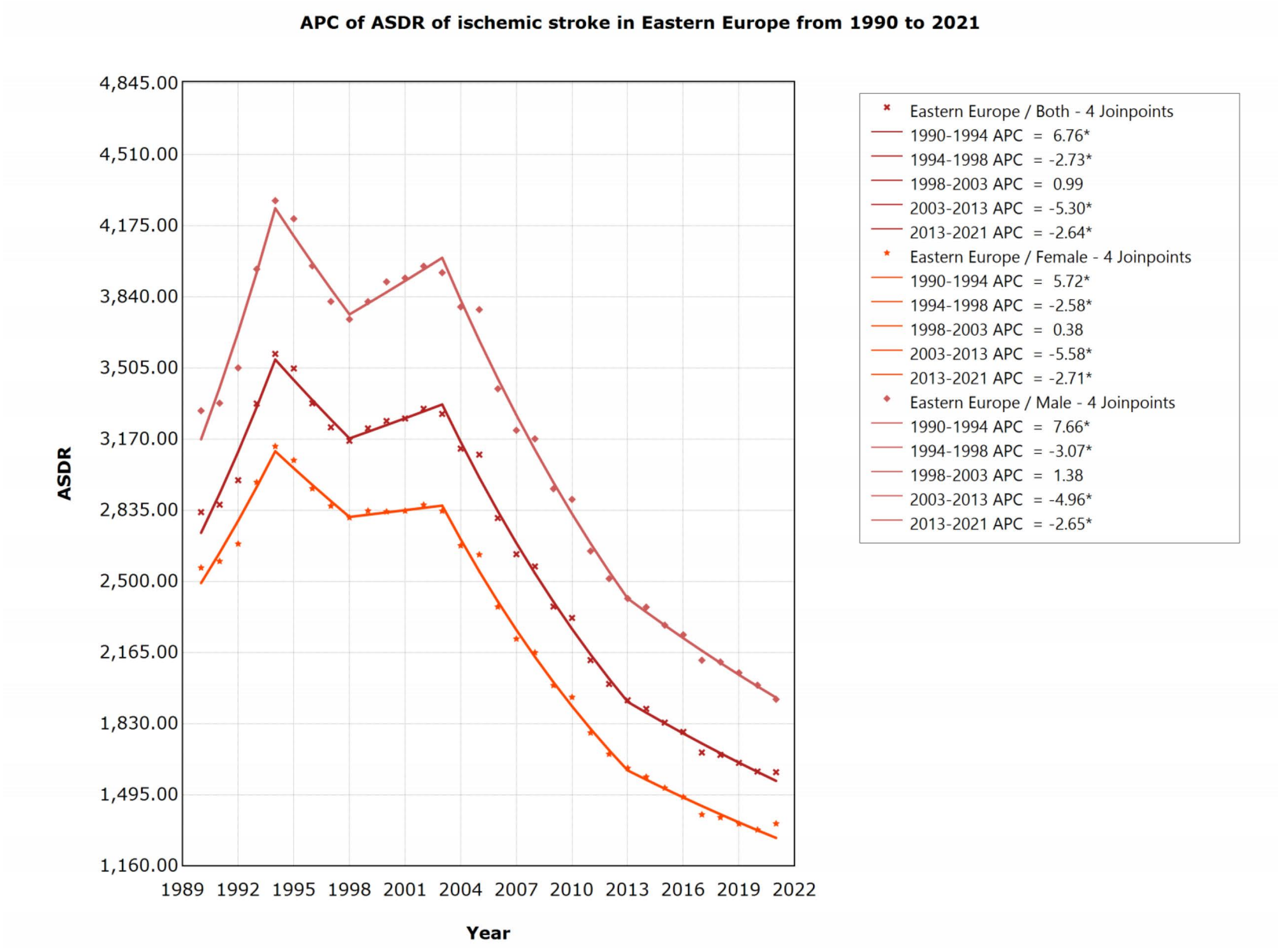
APC of ASDR of ischemic stroke in Eastern Europe from 1990 to 2021
ASDR
4,845.00
4,510.00
4,175.00
3,840.00
3,505.00
3,170.00
2,835.00
2,500.00
2,165.00
1,830.00
1,495.00
1,160.00
1989 1992 1995 1998 2001 2004 2007 2010 2013 2016 2019 2022
Year
Eastern Europe / Both - 4 Joinpoints
1990-1994 APC = 6.76*
1994-1998 APC = -2.73*
1998-2003 APC = 0.99
2003-2013 APC = -5.30*
2013-2021 APC = -2.64*
Eastern Europe / Female - 4 Joinpoints
1990-1994 APC = 5.72*
1994-1998 APC = -2.58*
1998-2003 APC = 0.38
2003-2013 APC = -5.58*
2013-2021 APC = -2.71*
Eastern Europe / Male - 4 Joinpoints
1990-1994 APC = 7.66*
1994-1998 APC = -3.07*
1998-2003 APC = 1.38
2003-2013 APC = -4.96*
2013-2021 APC = -2.65*

B

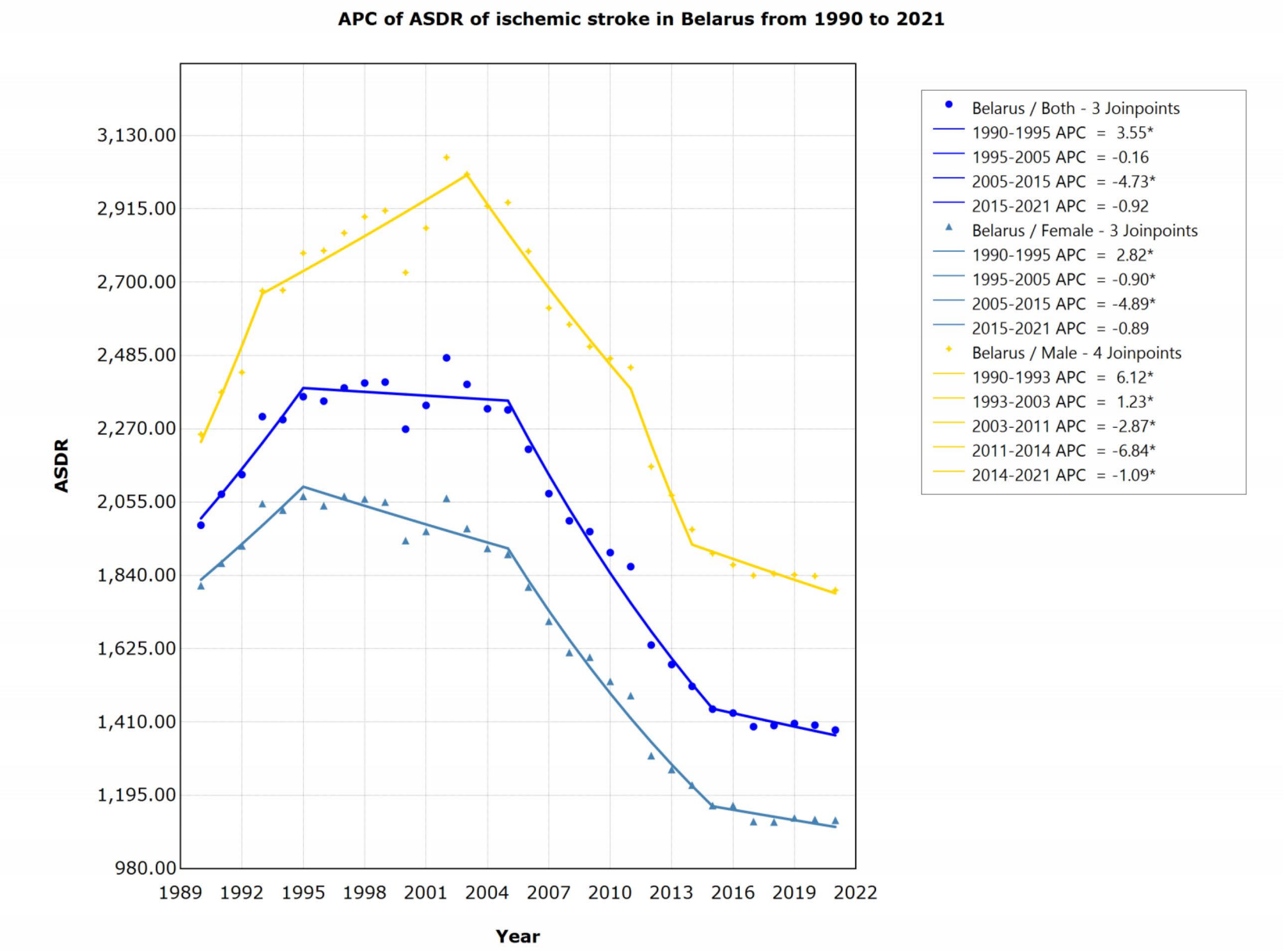
APC of ASDR of ischemic stroke in Belarus from 1990 to 2021
ASDR
3,130.00
2,915.00
2,700.00
2,485.00
2,270.00
2,055.00
1,840.00
1,625.00
1,410.00
1,195.00
980.00
1989
1992
1995
1998
2001
2004
2007
2010
2013
2016
2019
2022
Year
Belarus / Both - 3 Joinpoints
1990-1995 APC = 3.55*
1995-2005 APC = -0.16
2005-2015 APC = -4.73*
2015-2021 APC = -0.92
Belarus / Female - 3 Joinpoints
1990-1995 APC = 2.82*
1995-2005 APC = -0.90*
2005-2015 APC = -4.89*
2015-2021 APC = -0.89
Belarus / Male - 4 Joinpoints
1990-1993 APC = 6.12*
1993-2003 APC = 1.23*
2003-2011 APC = -2.87*
2011-2014 APC = -6.84*
2014-2021 APC = -1.09*

C

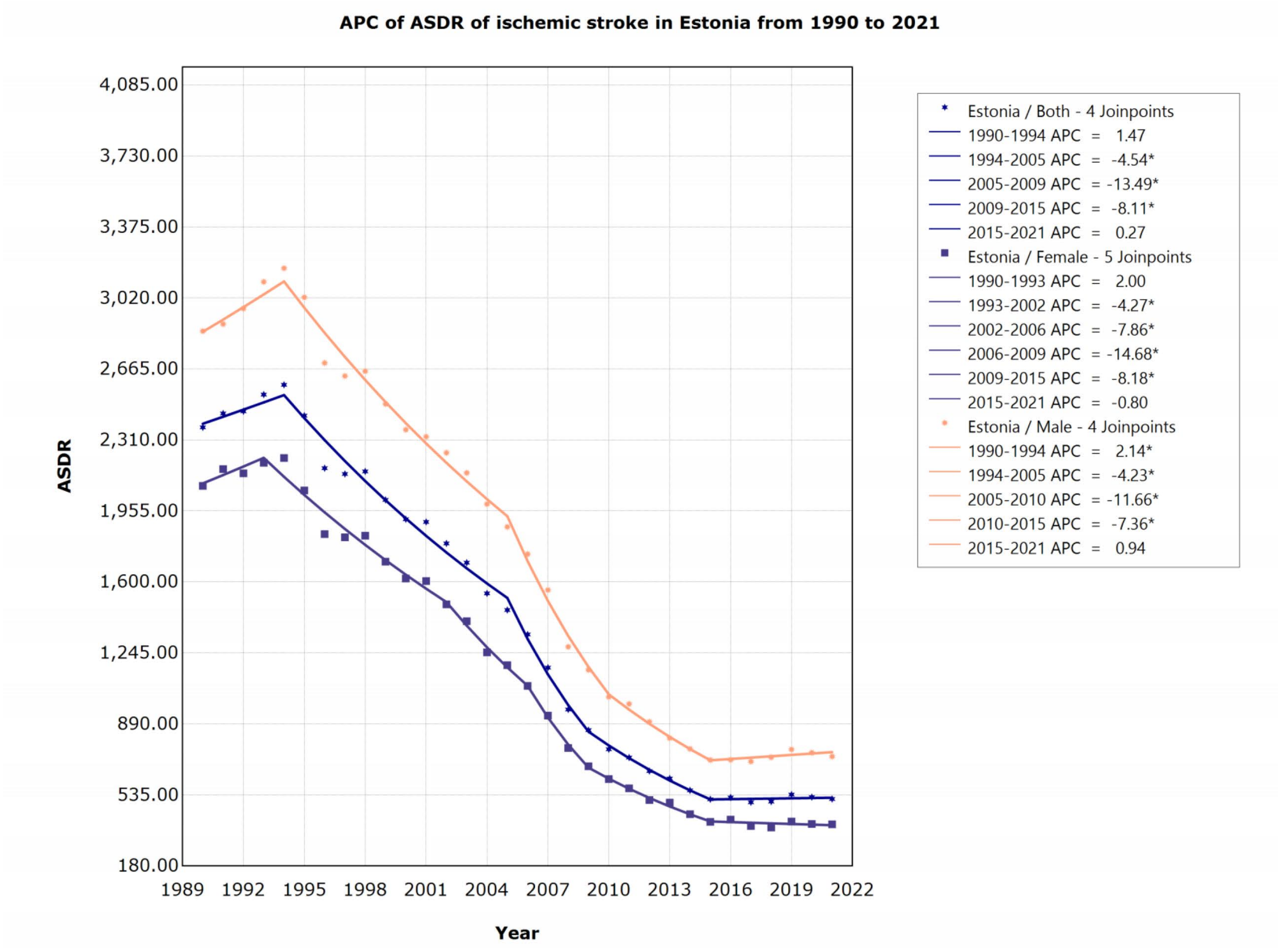

APC of ASDR of ischemic stroke in Estonia from 1990 to 2021
ASDR
4,085.00
3,730.00
3,375.00
3,020.00
2,665.00
2,310.00
1,955.00
1,600.00
1,245.00
890.00
535.00
180.00
1989
1992
1995
1998
2001
2004
2007
2010
2013
2016
2019
2022
Year
Estonia / Both - 4 Joinpoints
1990-1994 APC = 1.47
1994-2005 APC = -4.54*
2005-2009 APC = -13.49*
2009-2015 APC = -8.11*
2015-2021 APC = 0.27
Estonia / Female - 5 Joinpoints
1990-1993 APC = 2.00
1993-2002 APC = -4.27*
2002-2006 APC = -7.86*
2006-2009 APC = -14.68*
2009-2015 APC = -8.18*
2015-2021 APC = -0.80
Estonia / Male - 4 Joinpoints
1990-1994 APC = 2.14*
1994-2005 APC = -4.23*
2005-2010 APC = -11.66*
2010-2015 APC = -7.36*
2015-2021 APC = 0.94

D

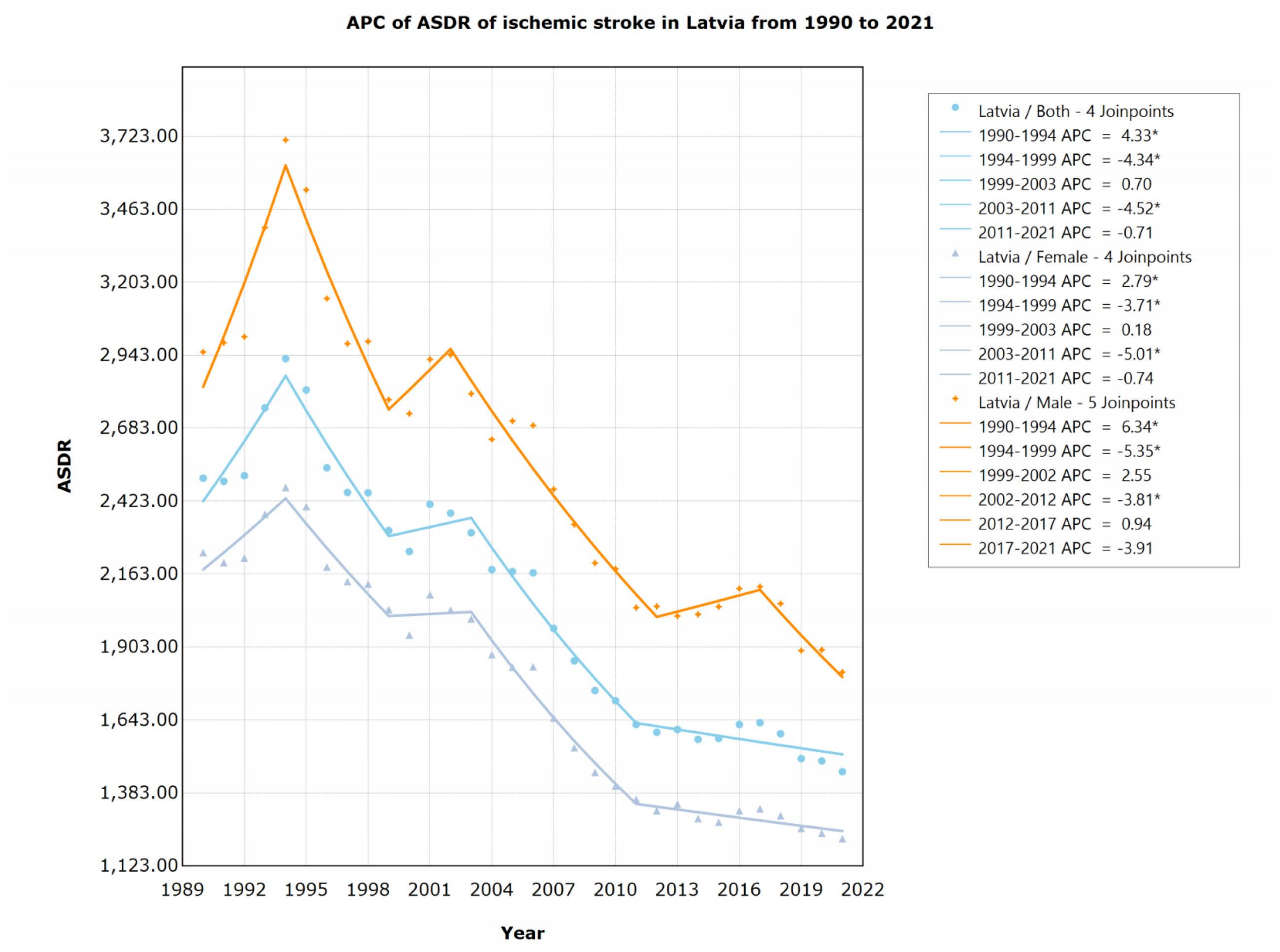
APC of ASDR of ischemic stroke in Latvia from 1990 to 2021
ASDR
Year
3,723.00
3,463.00
3,203.00
2,943.00
2,683.00
2,423.00
2,163.00
1,903.00
1,643.00
1,383.00
1,123.00
1989
1992
1995
1998
2001
2004
2007
2010
2013
2016
2019
2022
Latvia / Both - 4 Joinpoints
1990-1994 APC = 4.33*
1994-1999 APC = -4.34*
1999-2003 APC = 0.70
2003-2011 APC = -4.52*
2011-2021 APC = -0.71
Latvia / Female - 4 Joinpoints
1990-1994 APC = 2.79*
1994-1999 APC = -3.71*
1999-2003 APC = 0.18
2003-2011 APC = -5.01*
2011-2021 APC = -0.74
Latvia / Male - 5 Joinpoints
1990-1994 APC = 6.34*
1994-1999 APC = -5.35*
1999-2002 APC = 2.55
2002-2012 APC = -3.81*
2012-2017 APC = 0.94
2017-2021 APC = -3.91

E

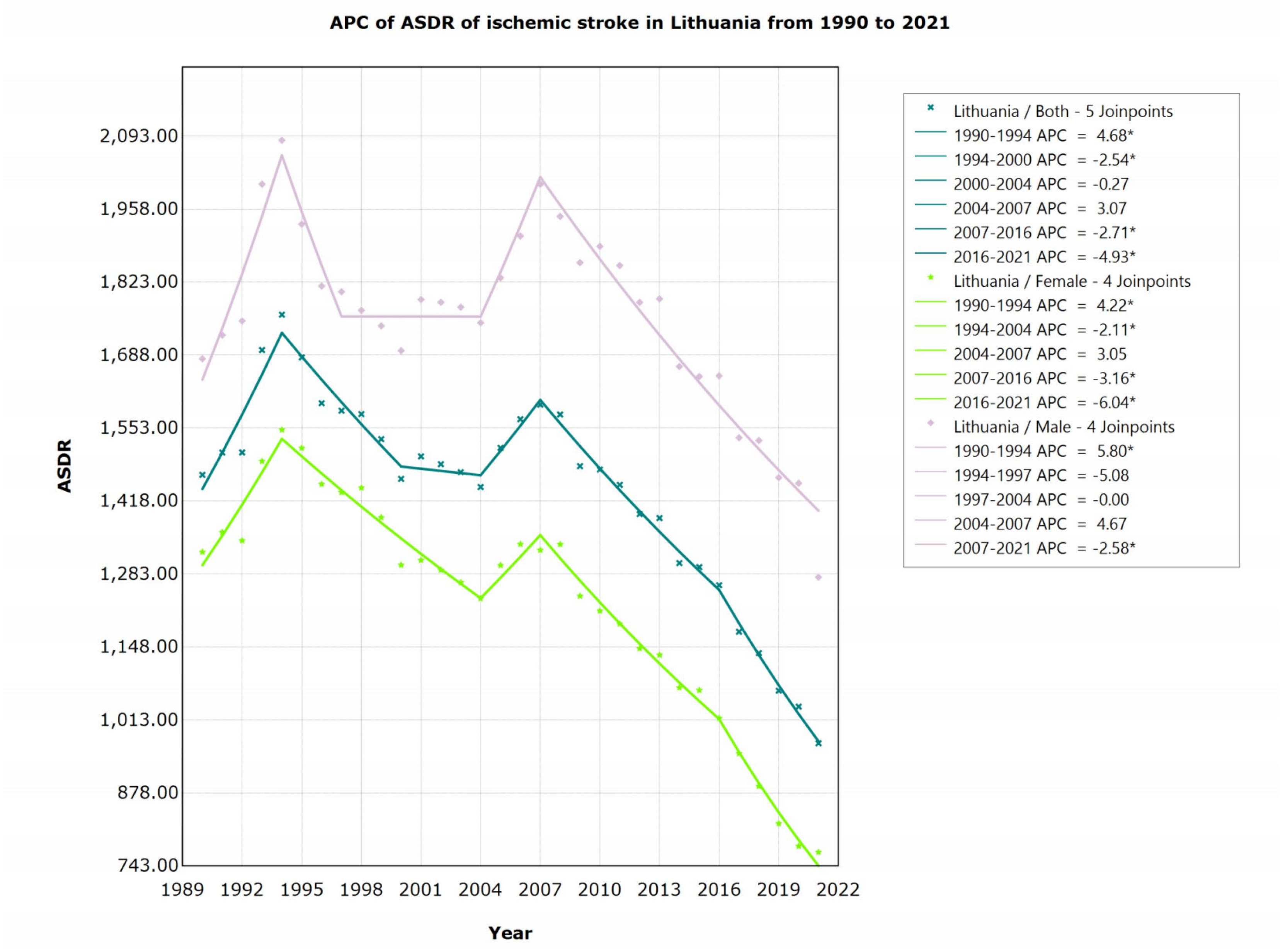

APC of ASDR of ischemic stroke in Lithuania from 1990 to 2021
ASDR
2,093.00
1,958.00
1,823.00
1,688.00
1,553.00
1,418.00
1,283.00
1,148.00
1,013.00
878.00
743.00
1989 1992 1995 1998 2001 2004 2007 2010 2013 2016 2019 2022
Year
Lithuania / Both - 5 Joinpoints
1990-1994 APC = 4.68*
1994-2000 APC = -2.54*
2000-2004 APC = -0.27
2004-2007 APC = 3.07
2007-2016 APC = -2.71*
2016-2021 APC = -4.93*
Lithuania / Female - 4 Joinpoints
1990-1994 APC = 4.22*
1994-2004 APC = -2.11*
2004-2007 APC = 3.05
2007-2016 APC = -3.16*
2016-2021 APC = -6.04*
Lithuania / Male - 4 Joinpoints
1990-1994 APC = 5.80*
1994-1997 APC = -5.08
1997-2004 APC = -0.00
2004-2007 APC = 4.67
2007-2021 APC = -2.58*

F

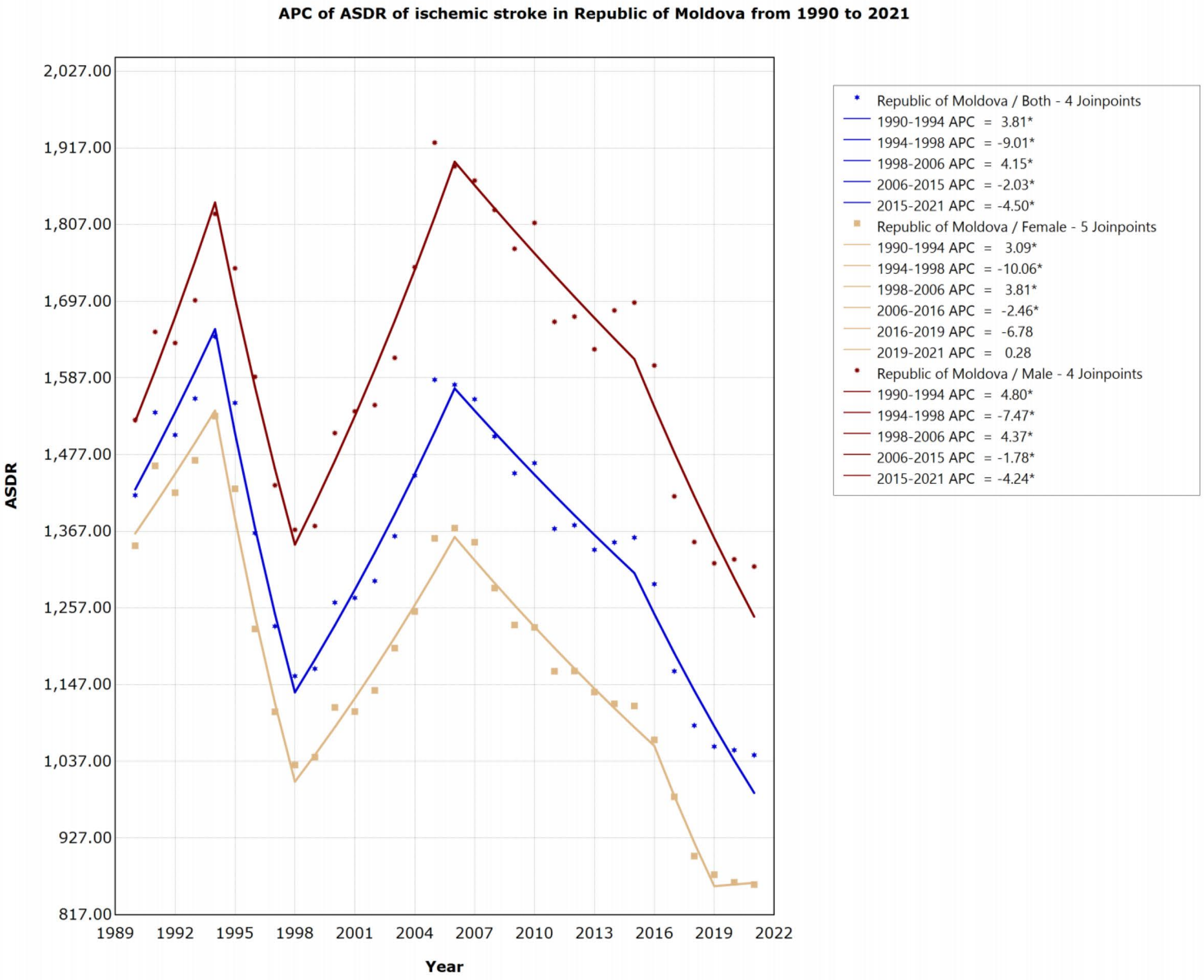
APC of ASDR of ischemic stroke in Republic of Moldova from 1990 to 2021
ASDR
Year
2,027.00
1,917.00
1,807.00
1,697.00
1,587.00
1,477.00
1,367.00
1,257.00
1,147.00
1,037.00
927.00
817.00
1989
1992
1995
1998
2001
2004
2007
2010
2013
2016
2019
2022
Republic of Moldova / Both - 4 Joinpoints
1990-1994 APC = 3.81*
1994-1998 APC = -9.01*
1998-2006 APC = 4.15*
2006-2015 APC = -2.03*
2015-2021 APC = -4.50*
Republic of Moldova / Female - 5 Joinpoints
1990-1994 APC = 3.09*
1994-1998 APC = -10.06*
1998-2006 APC = 3.81*
2006-2016 APC = -2.46*
2016-2019 APC = -6.78
2019-2021 APC = 0.28
Republic of Moldova / Male - 4 Joinpoints
1990-1994 APC = 4.80*
1994-1998 APC = -7.47*
1998-2006 APC = 4.37*
2006-2015 APC = -1.78*
2015-2021 APC = -4.24*

G

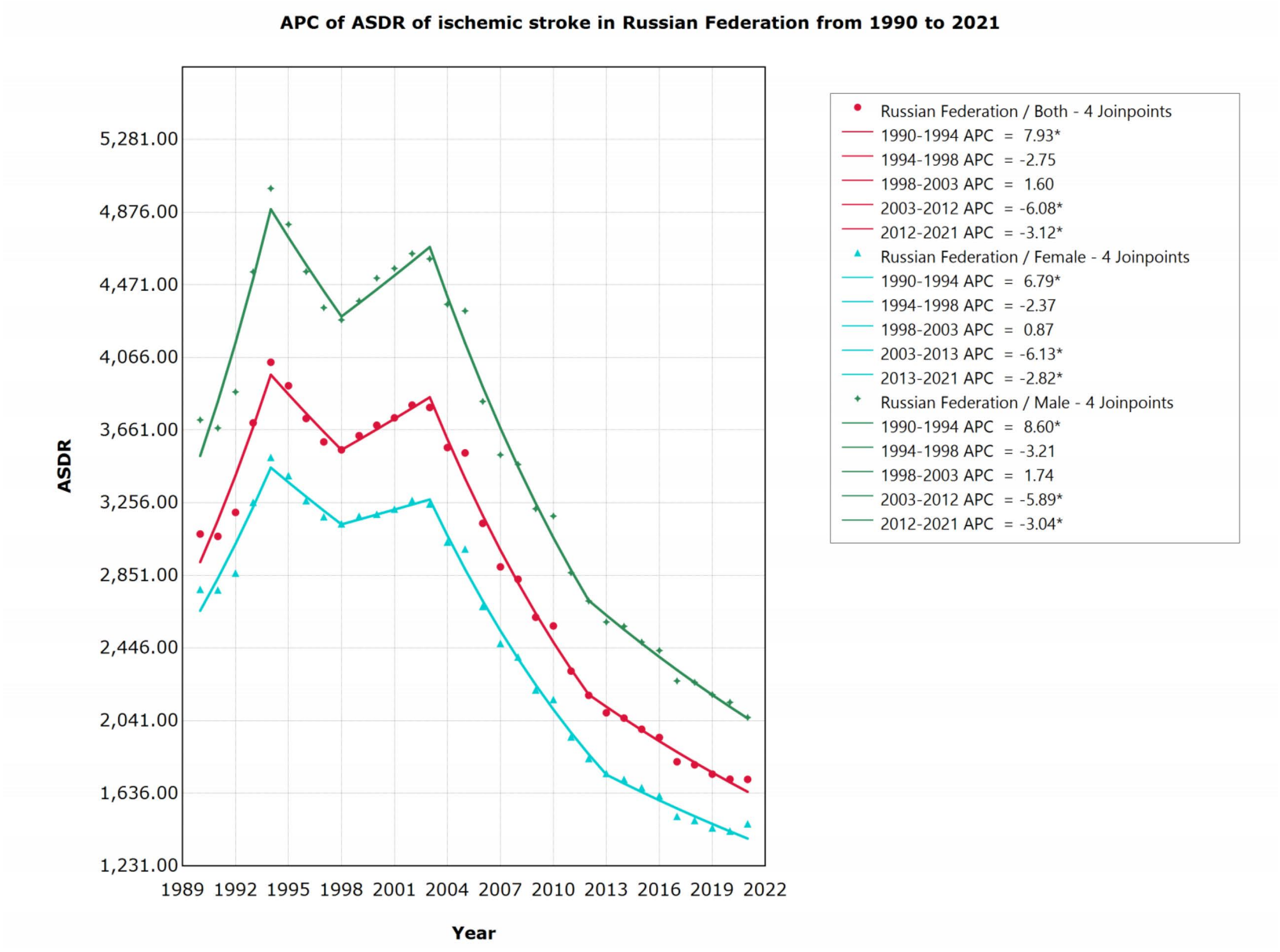

APC of ASDR of ischemic stroke in Russian Federation from 1990 to 2021
5,281.00
4,876.00
4,471.00
4,066.00
3,661.00
3,256.00
2,851.00
2,446.00
2,041.00
1,636.00
1,231.00
ASDR
1989 1992 1995 1998 2001 2004 2007 2010 2013 2016 2019 2022
Year
Russian Federation / Both - 4 Joinpoints
1990-1994 APC = 7.93*
1994-1998 APC = -2.75
1998-2003 APC = 1.60
2003-2012 APC = -6.08*
2012-2021 APC = -3.12*
Russian Federation / Female - 4 Joinpoints
1990-1994 APC = 6.79*
1994-1998 APC = -2.37
1998-2003 APC = 0.87
2003-2013 APC = -6.13*
2013-2021 APC = -2.82*
Russian Federation / Male - 4 Joinpoints
1990-1994 APC = 8.60*
1994-1998 APC = -3.21
1998-2003 APC = 1.74
2003-2012 APC = -5.89*
2012-2021 APC = -3.04*

H

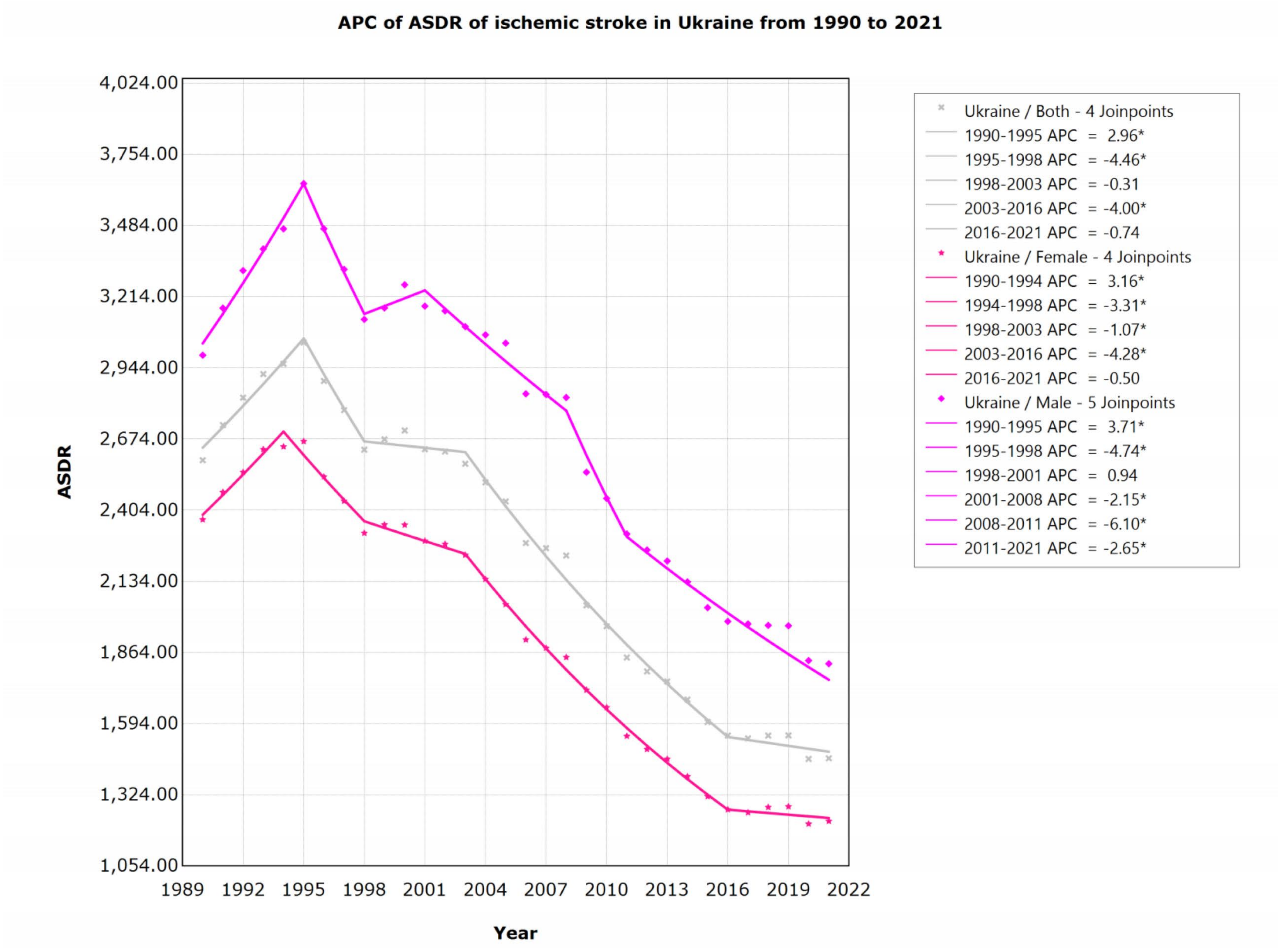
APC of ASDR of ischemic stroke in Ukraine from 1990 to 2021
4,024.00
3,754.00
3,484.00
3,214.00
2,944.00
2,674.00
2,404.00
2,134.00
1,864.00
1,594.00
1,324.00
1,054.00
ASDR
1989 1992 1995 1998 2001 2004 2007 2010 2013 2016 2019 2022
Year
Ukraine / Both - 4 Joinpoints
1990-1995 APC = 2.96*
1995-1998 APC = -4.46*
1998-2003 APC = -0.31
2003-2016 APC = -4.00*
2016-2021 APC = -0.74
Ukraine / Female - 4 Joinpoints
1990-1994 APC = 3.16*
1994-1998 APC = -3.31*
1998-2003 APC = -1.07*
2003-2016 APC = -4.28*
2016-2021 APC = -0.50
Ukraine / Male - 5 Joinpoints
1990-1995 APC = 3.71*
1995-1998 APC = -4.74*
1998-2001 APC = 0.94
2001-2008 APC = -2.15*
2008-2011 APC = -6.10*
2011-2021 APC = -2.65*

Supplementary Figure 3. Annual percentage change of age-standardized DALYs rates of ischemic stroke in Eastern European Countries from 1990 to 2021: A. APC of ASDR of ischemic stroke in Eastern Europe from 1990 to 2021; B. APC of ASDR of ischemic stroke in Belarus from 1990 to 2021; C. APC of ASDR of ischemic stroke in Estonia from 1990 to 2021; D. APC of ASDR of ischemic stroke in Latvia from 1990 to 2021; E. APC of ASDR of ischemic stroke in Lithuania from 1990 to 2021; F. APC of ASDR of ischemic stroke in Republic of Moldova from 1990 to 2021; G. APC of ASDR of ischemic stroke in Russian Federation from 1990 to 2021; H. APC of ASDR of ischemic stroke in Ukraine from 1990 to 2021. APC, Annual percentage change; ASDR, age-standardized DALYs rate.

A

Dual X-axis plot illustrating the burden of ischemic stroke death attributable to environmental and behavioral risk factors in Eastern Europe

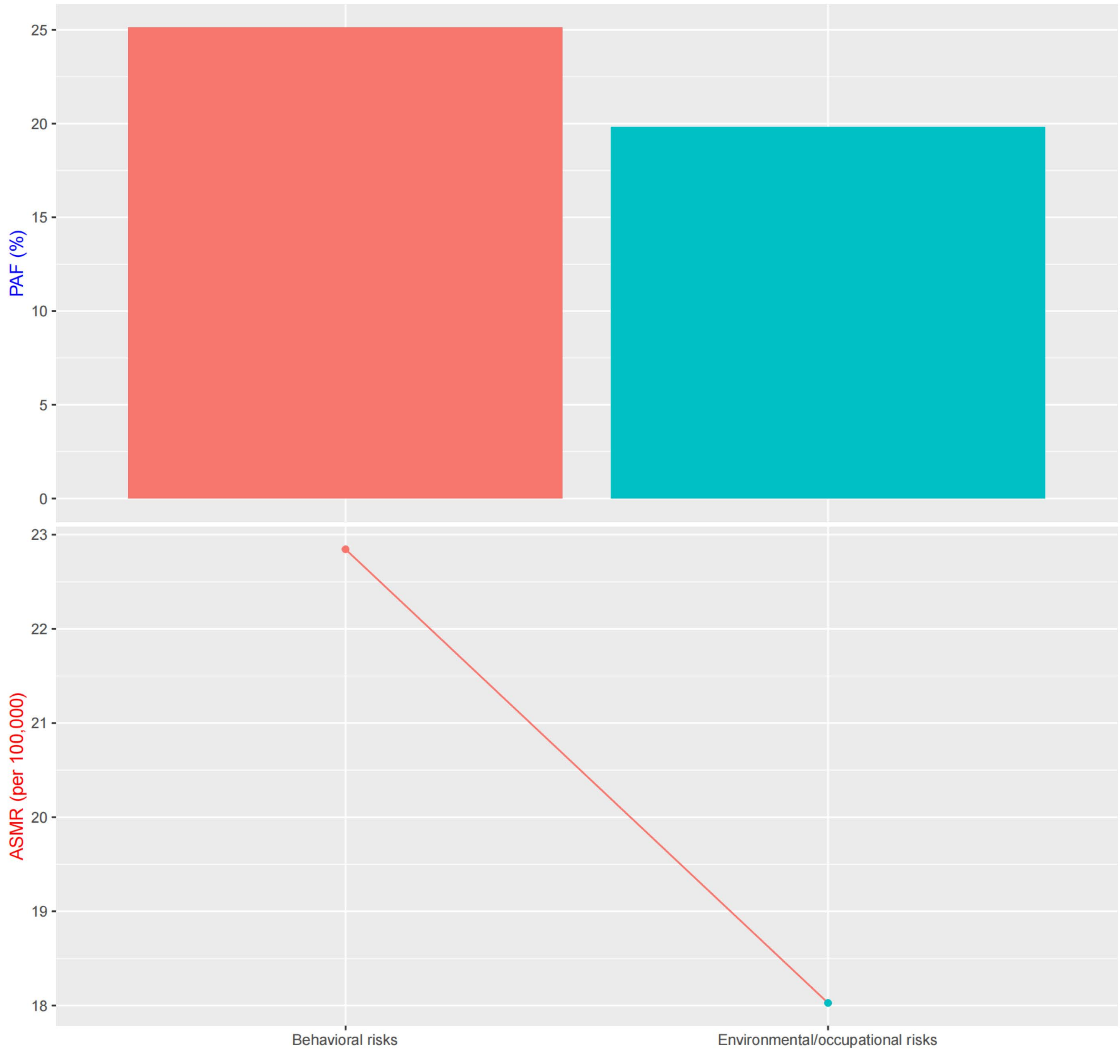

B

Dual X-axis plot illustrating the burden of ischemic stroke death attributable to environmental and behavioral risk factors in Belarus

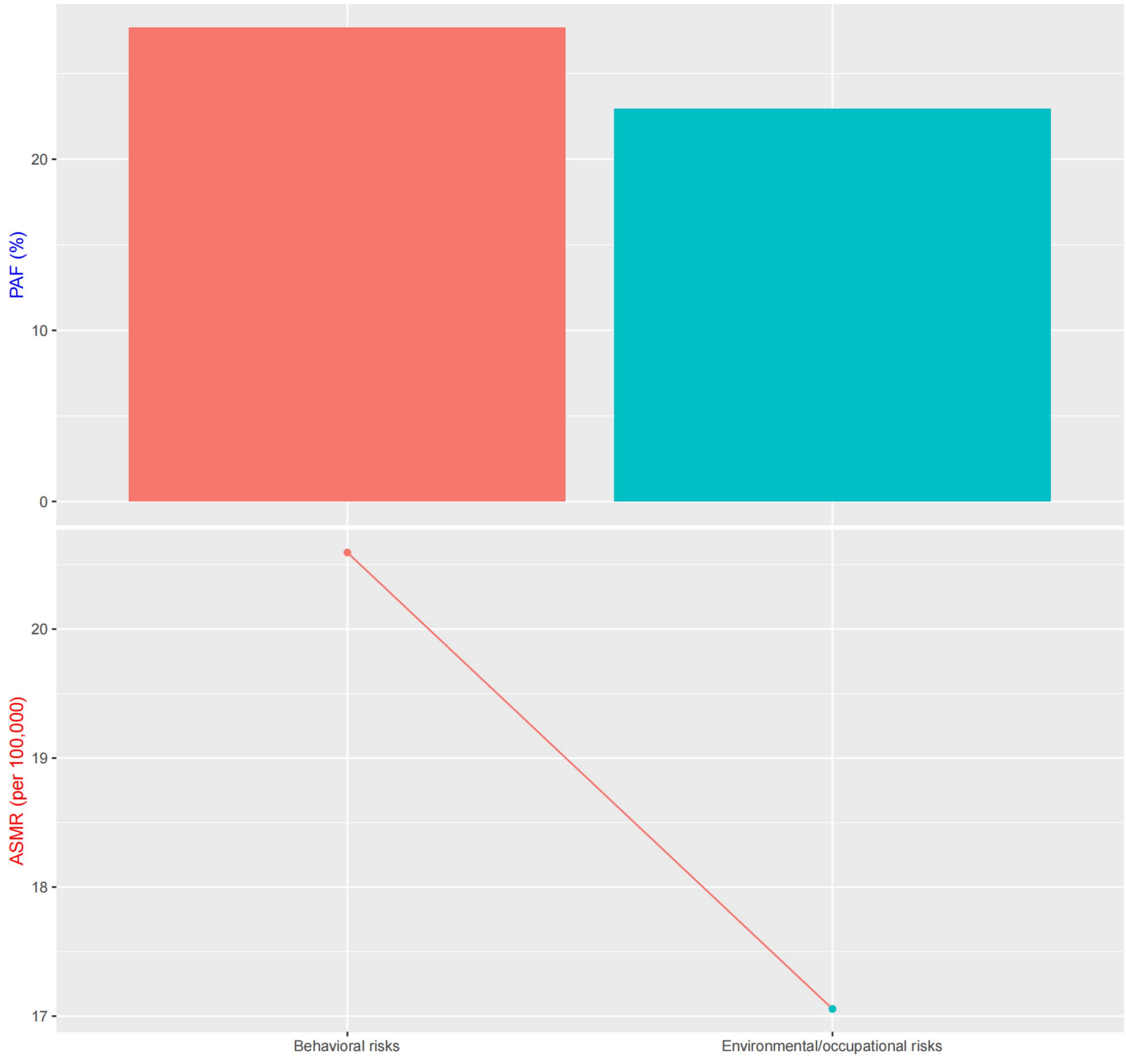

C

Dual X-axis plot illustrating the burden of ischemic stroke death attributable to environmental and behavioral risk factors in Estonia

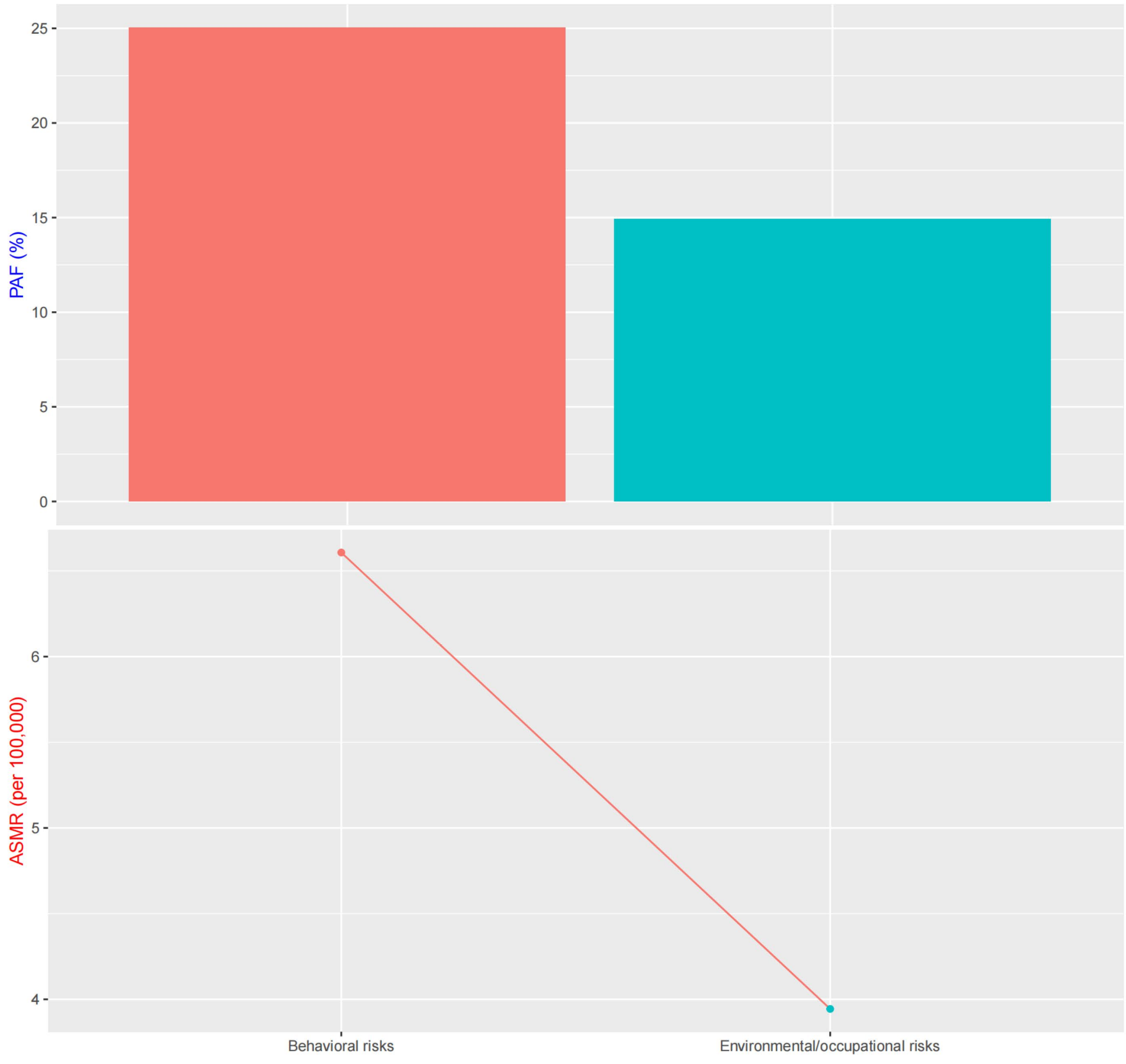

D

Dual X-axis plot illustrating the burden of ischemic stroke death attributable to environmental and behavioral risk factors in Latvia

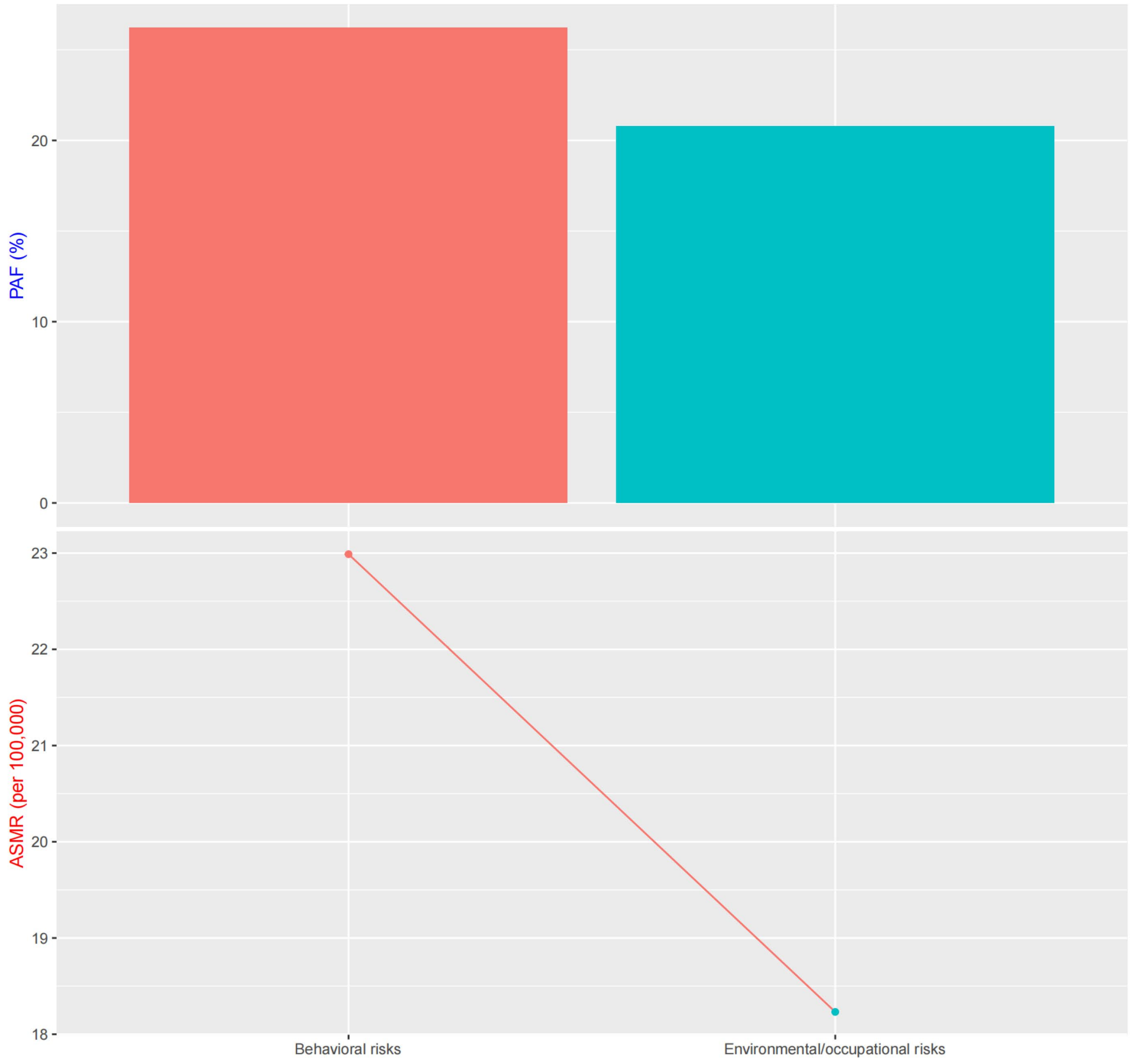

E

Dual X-axis plot illustrating the burden of ischemic stroke death attributable to environmental and behavioral risk factors in Lithuania

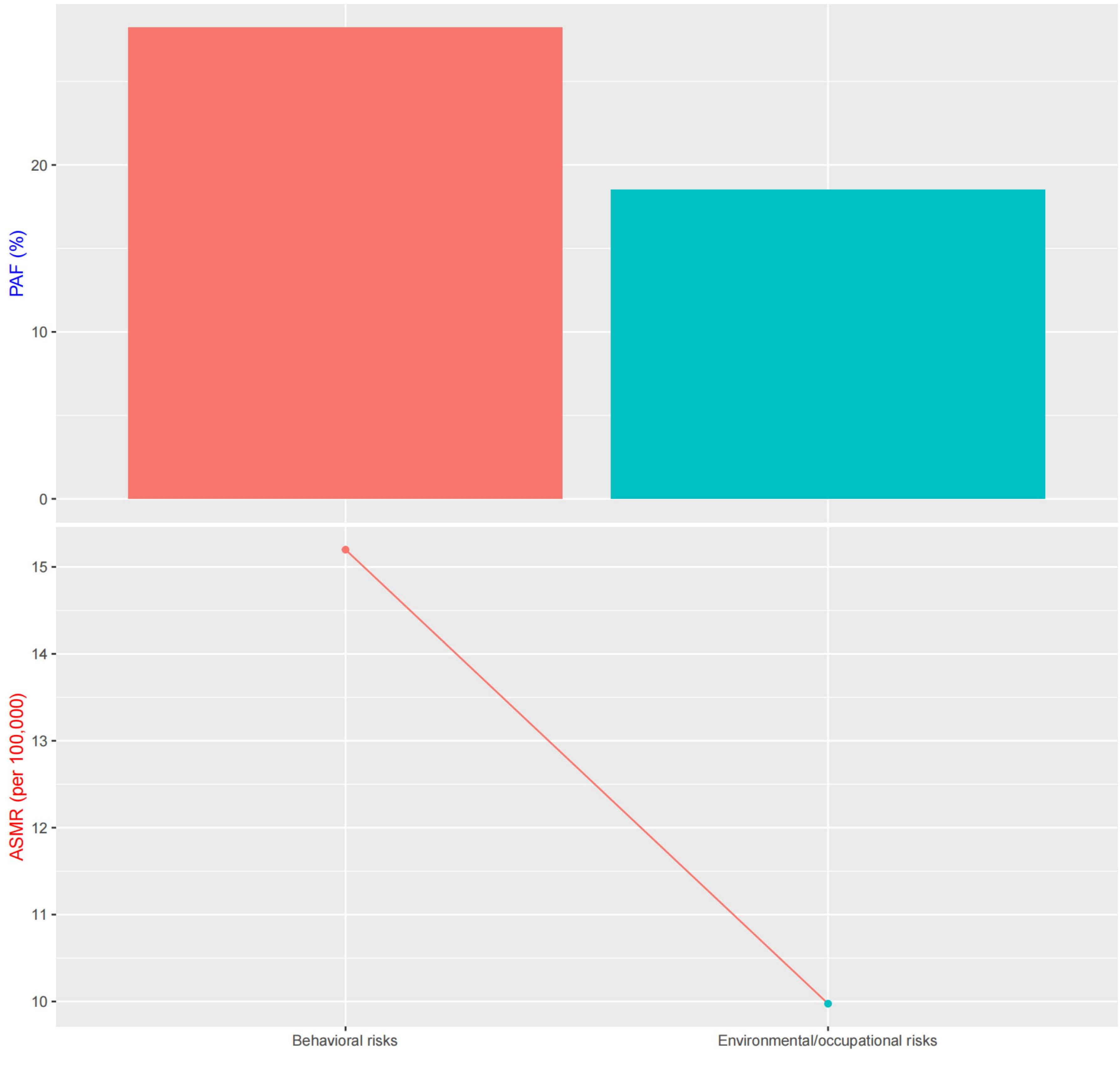

F

Dual X-axis plot illustrating the burden of ischemic stroke death attributable to environmental and behavioral risk factors in Republic of Moldova

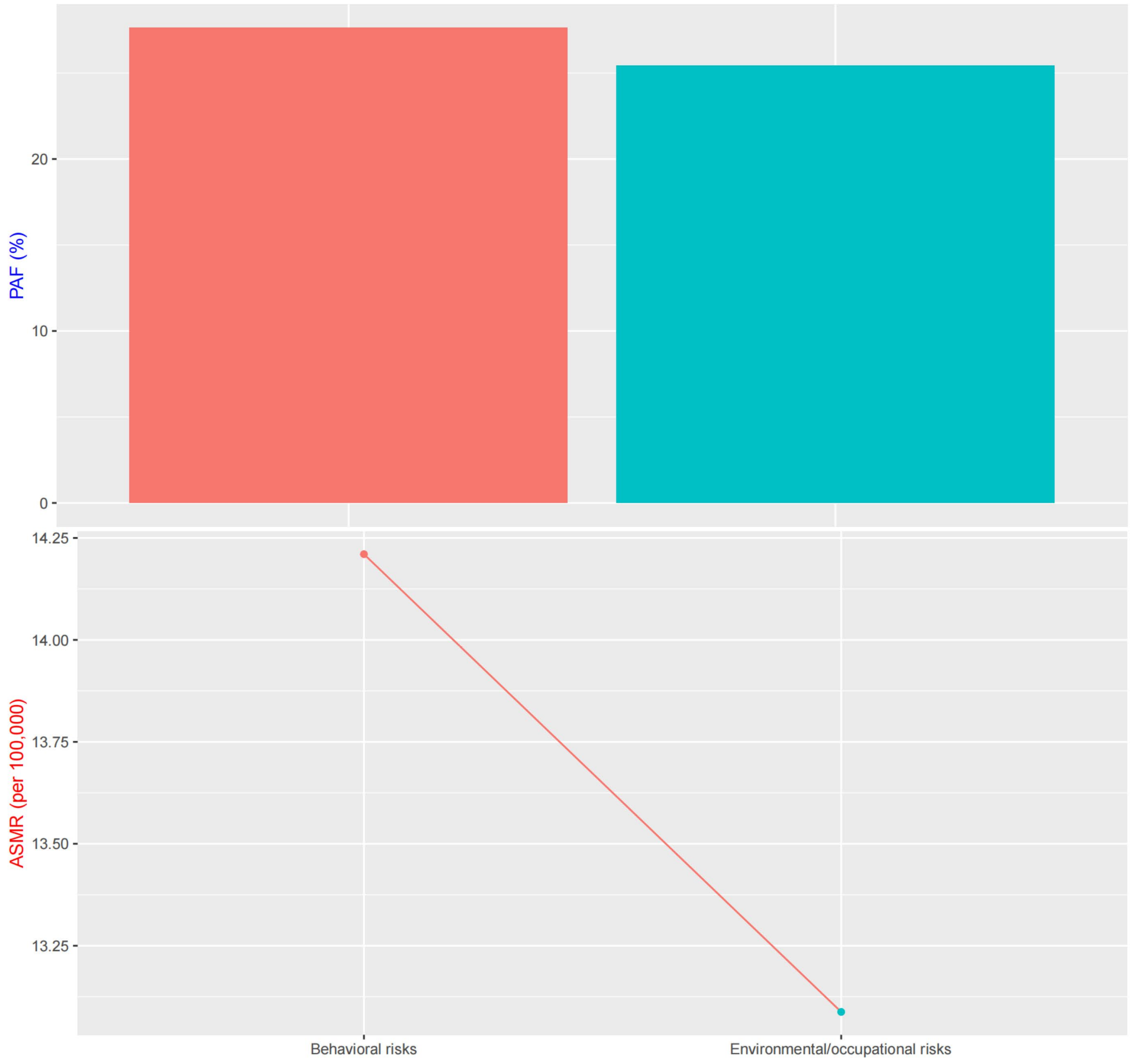

G

Dual X-axis plot illustrating the burden of ischemic stroke death attributable to environmental and behavioral risk factors in Russian Federation

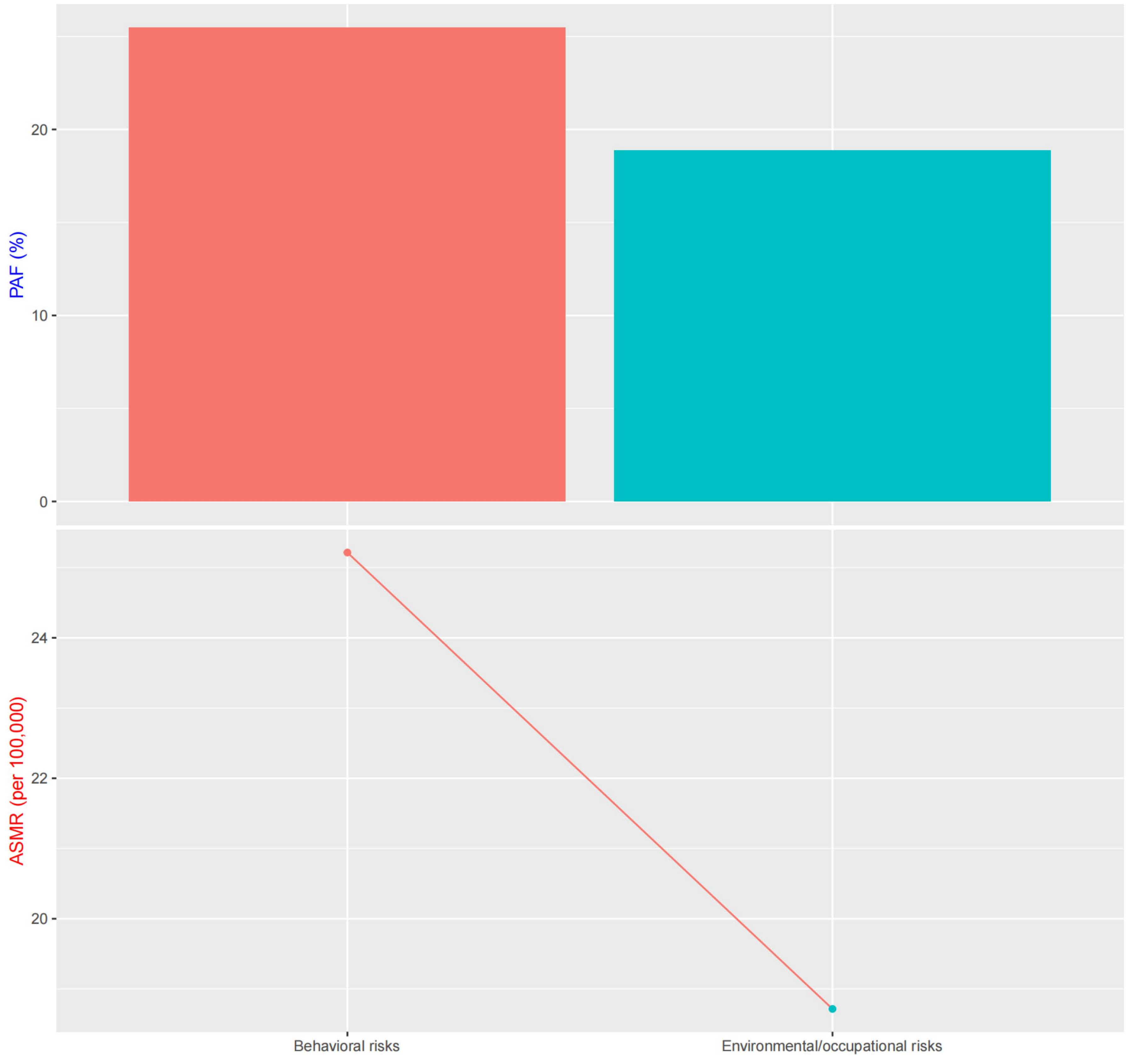

H

Dual X-axis plot illustrating the burden of ischemic stroke death attributable to environmental and behavioral risk factors in Ukraine

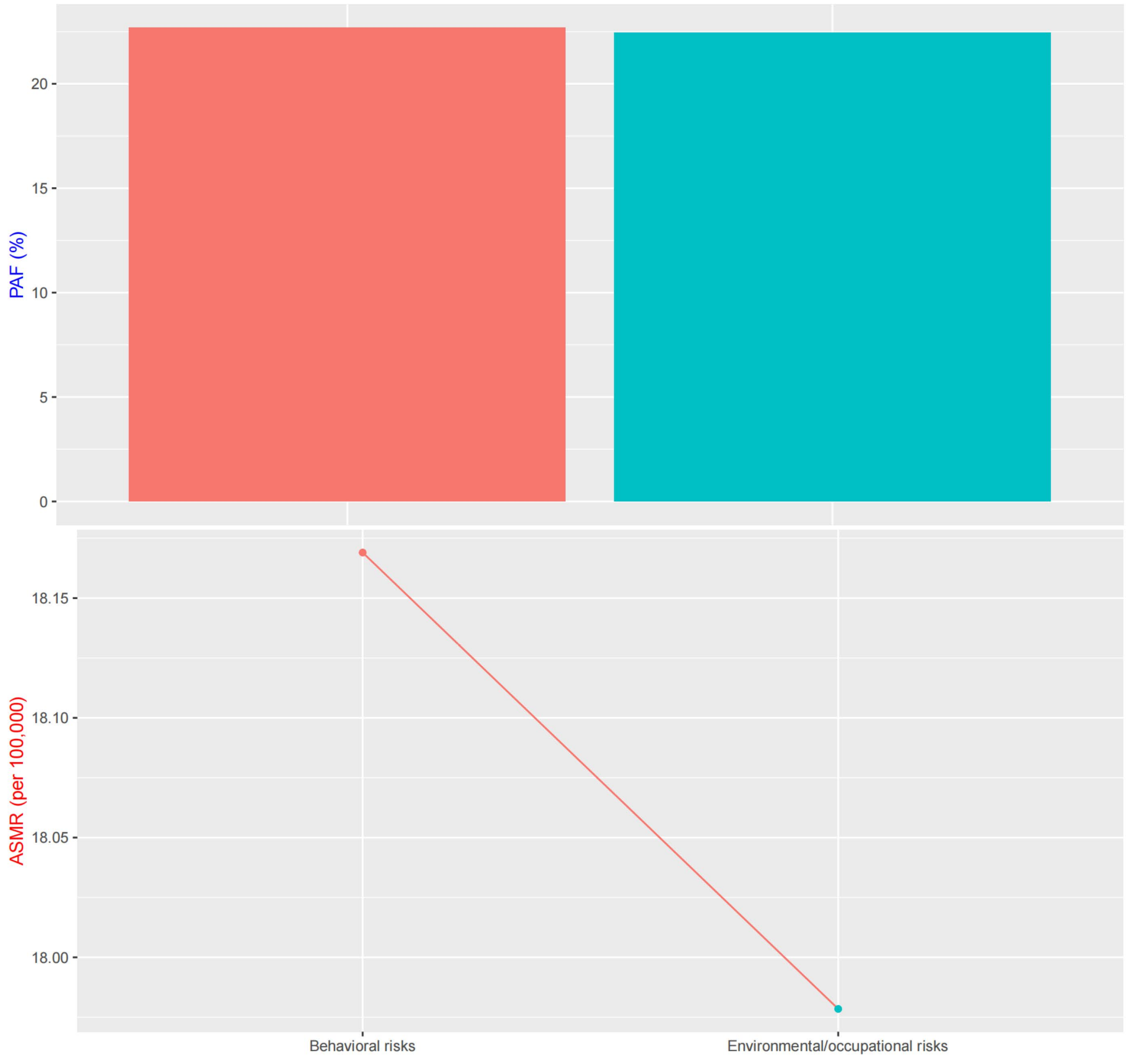

Supplementary Figure 4. Dual X-axis plot illustrating the burden of ischemic stroke death attributable to various risk factors (environmental/occupational risks and behavioral risks) in Eastern European Countries: A. Dual X-axis plot illustrating the burden of ischemic stroke death attributable to environmental and behavioral risk factors in Eastern Europe; B. Dual X-axis plot illustrating the burden of ischemic stroke death attributable to environmental and behavioral risk factors in Belarus; C. Dual X-axis plot illustrating the burden of ischemic stroke death attributable to environmental and behavioral risk factors in Estonia; D. Dual X-axis plot illustrating the burden of ischemic stroke death attributable to environmental and behavioral risk factors in Latvia; E. Dual X-axis plot illustrating the burden of ischemic stroke death attributable to environmental and behavioral risk factors in Lithuania; F. Dual X-axis plot illustrating the burden of ischemic stroke death attributable to environmental and behavioral risk factors in Republic of Moldova; G. Dual X-axis plot illustrating the burden of ischemic stroke death attributable to environmental and behavioral risk factors in Russian Federation; H. Dual X-axis plot illustrating the burden of ischemic stroke death attributable to environmental and behavioral risk factors in Ukraine.

A

Dual X-axis plot illustrating the burden of ischemic stroke death attributable to four environmental/occupational risk factors in Eastern Europe

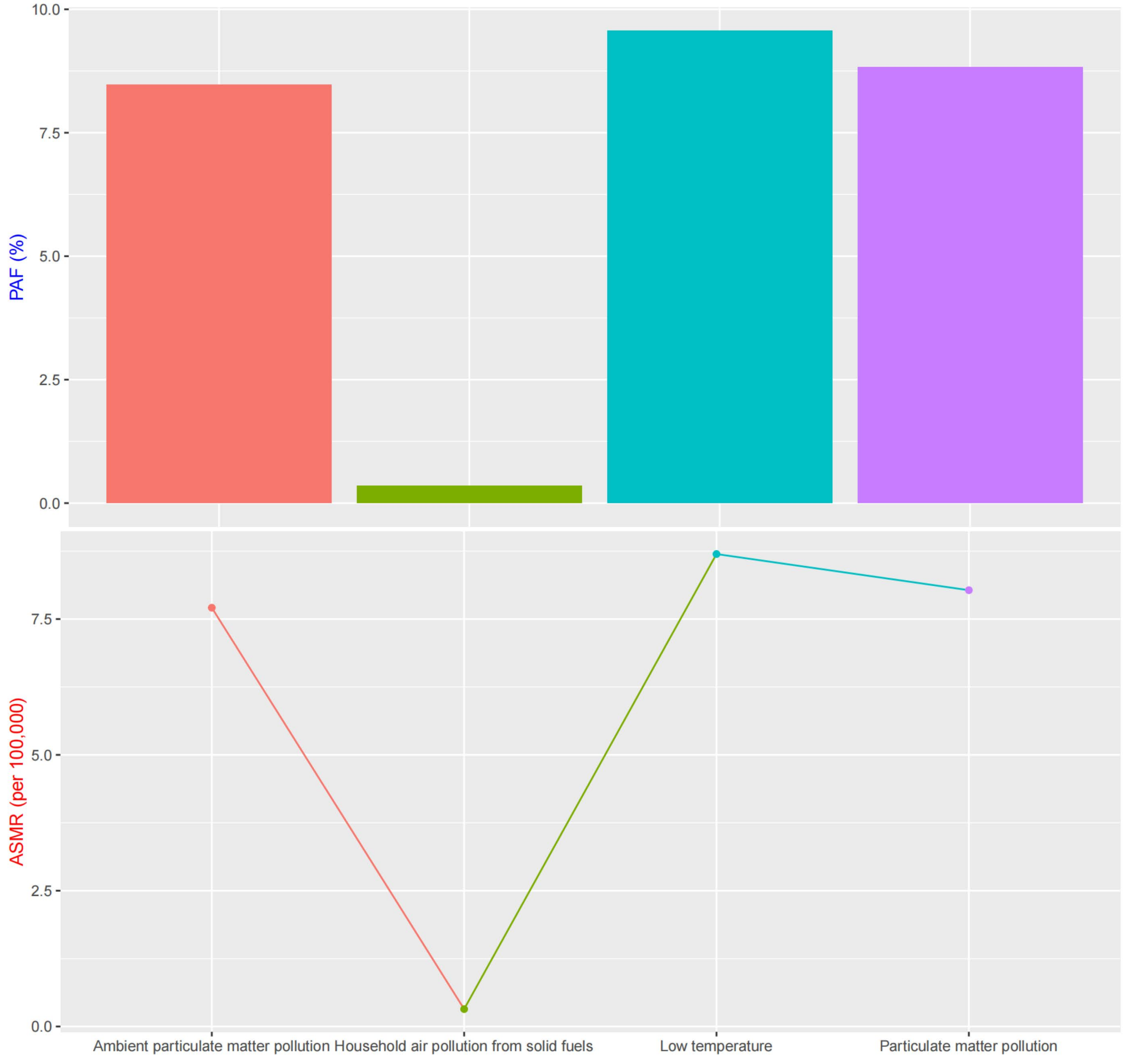

B

Dual X-axis plot illustrating the burden of ischemic stroke death attributable to four environmental/occupational risk factors in Belarus

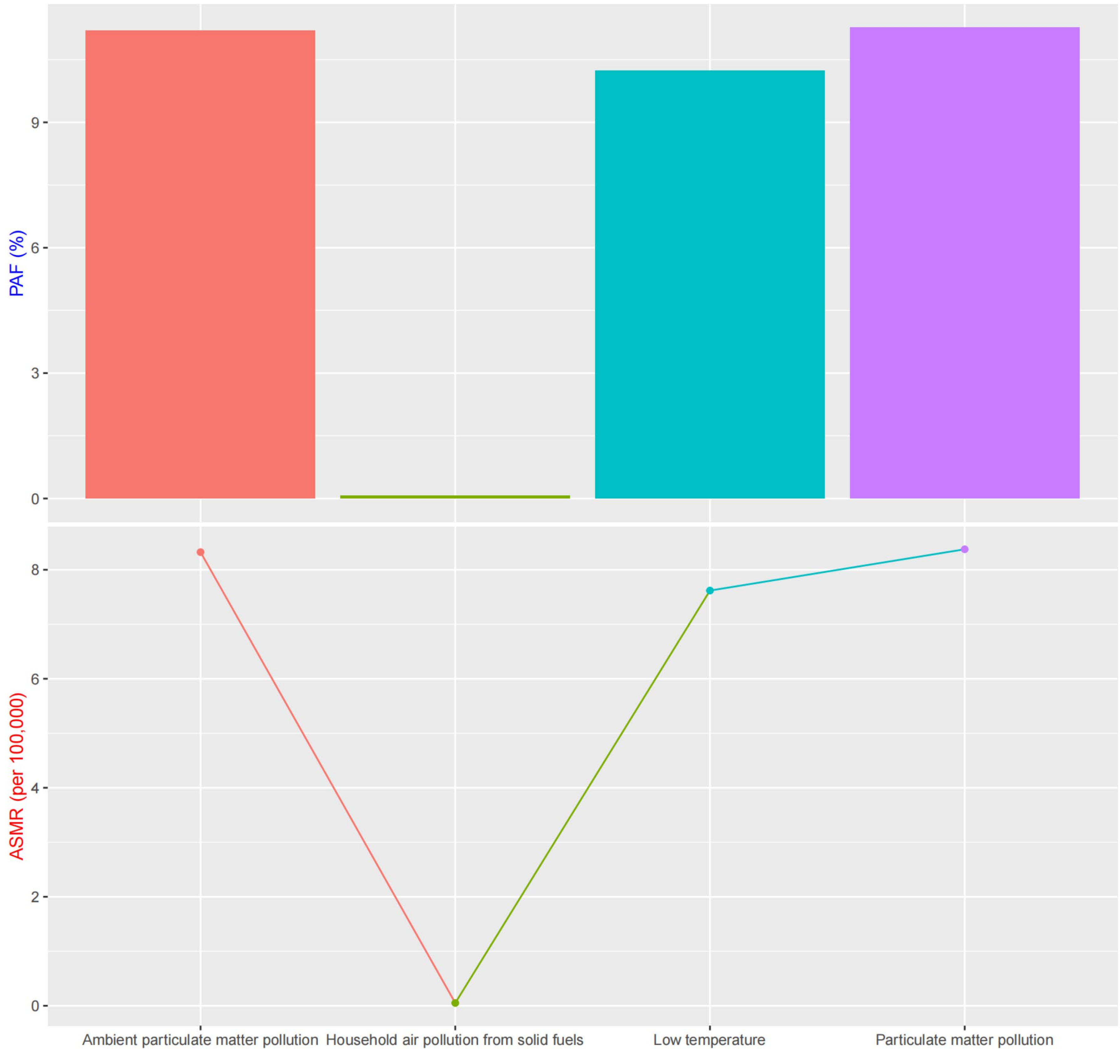

C

Dual X-axis plot illustrating the burden of ischemic stroke death attributable to four environmental/occupational risk factors in Estonia

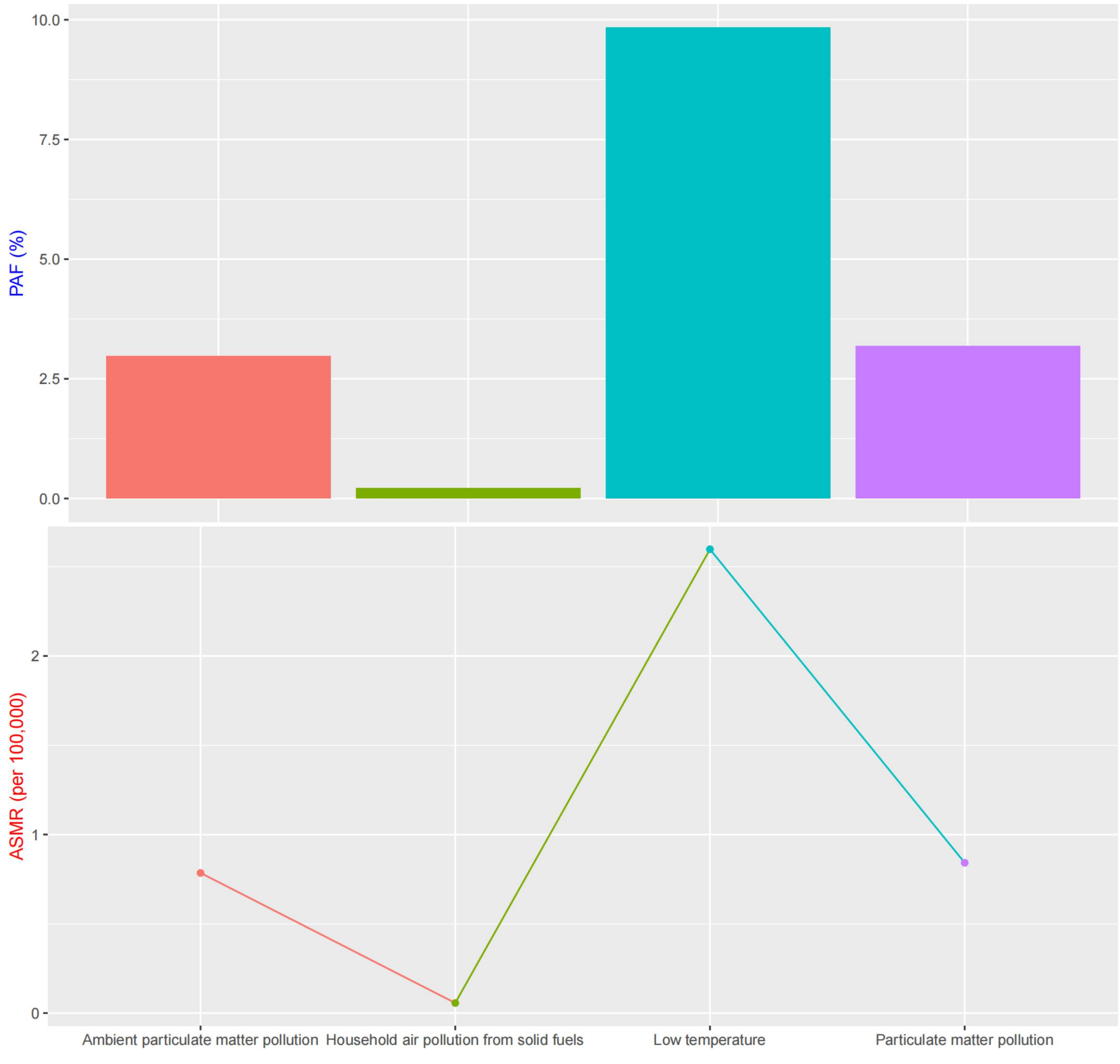

D

Dual X-axis plot illustrating the burden of ischemic stroke death attributable to four environmental/occupational risk factors in Latvia

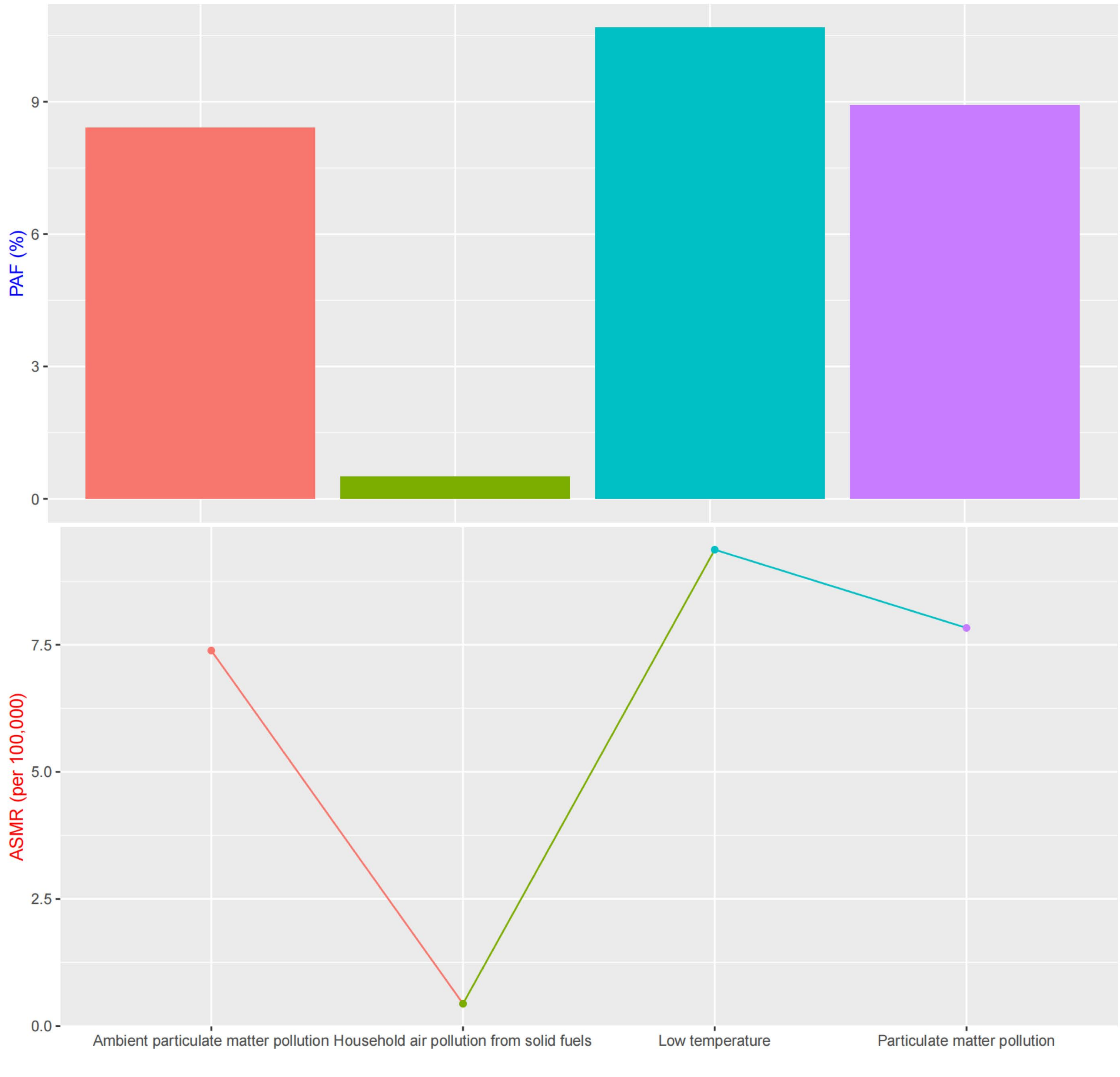

E

Dual X-axis plot illustrating the burden of ischemic stroke death attributable to four environmental/occupational risk factors in Lithuania

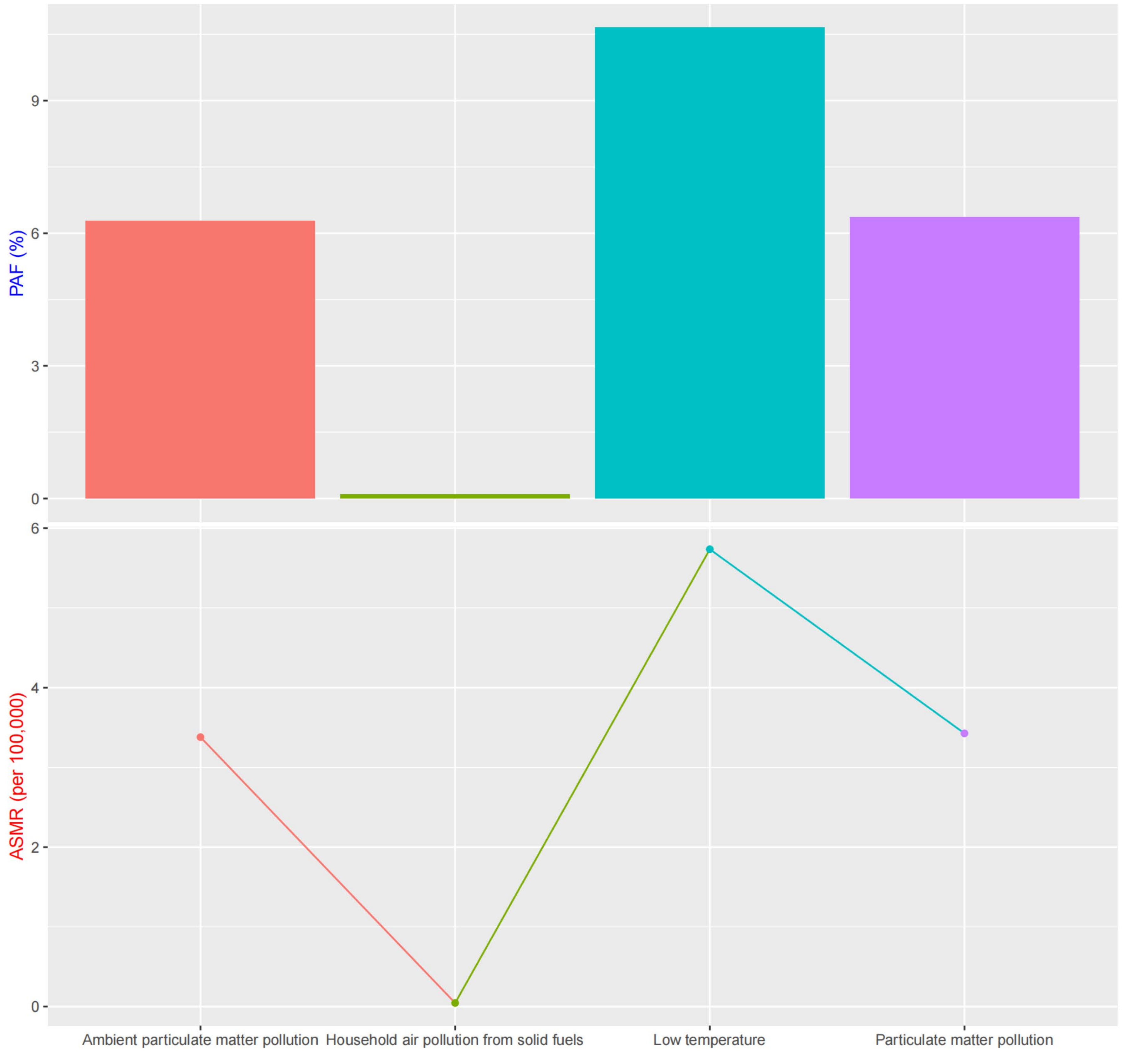

F

Dual X-axis plot illustrating the burden of ischemic stroke death attributable to four environmental/occupational risk factors in Republic of Moldova

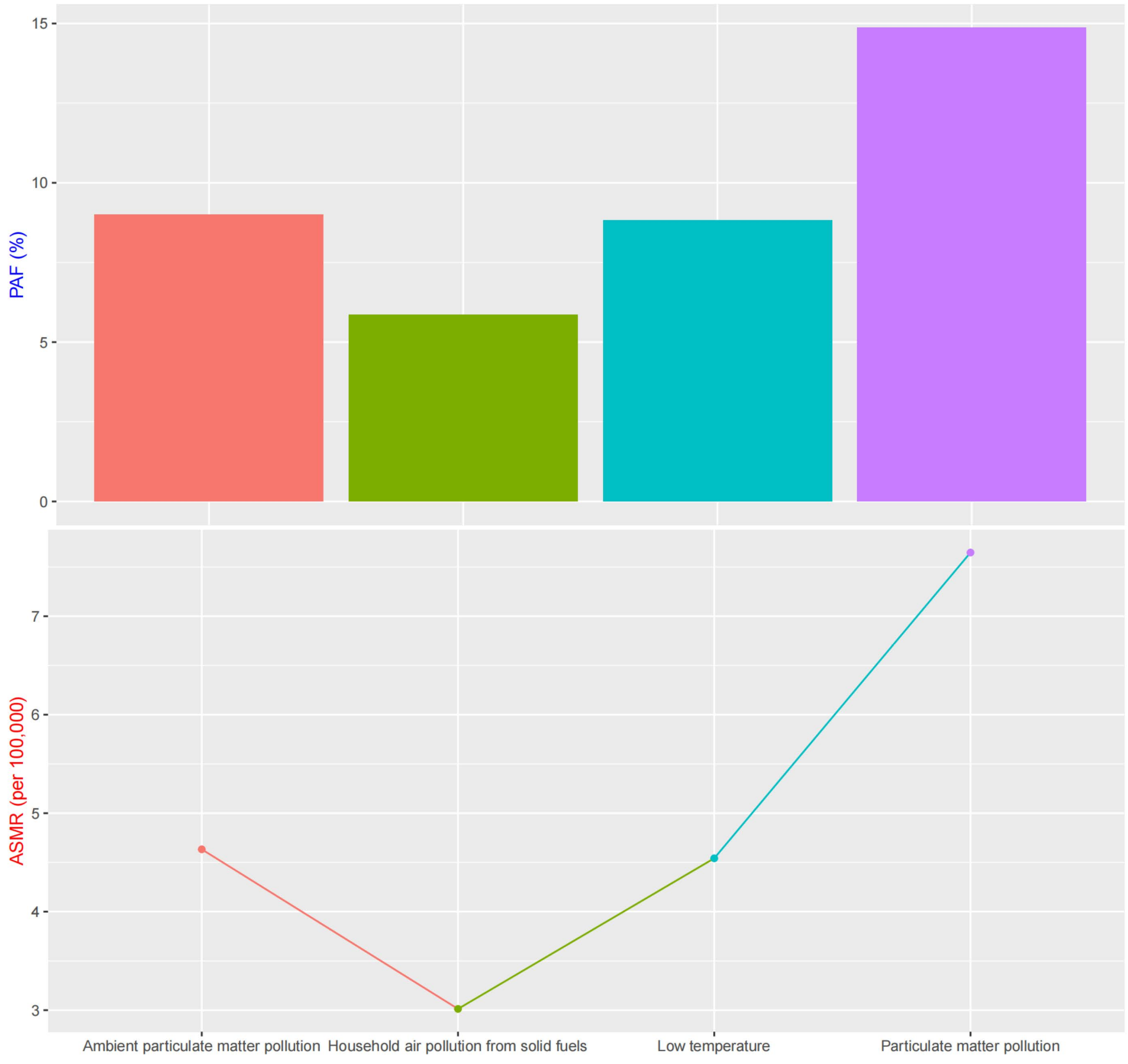

G

Dual X-axis plot illustrating the burden of ischemic stroke death attributable to four environmental/occupational risk factors in Russian Federation

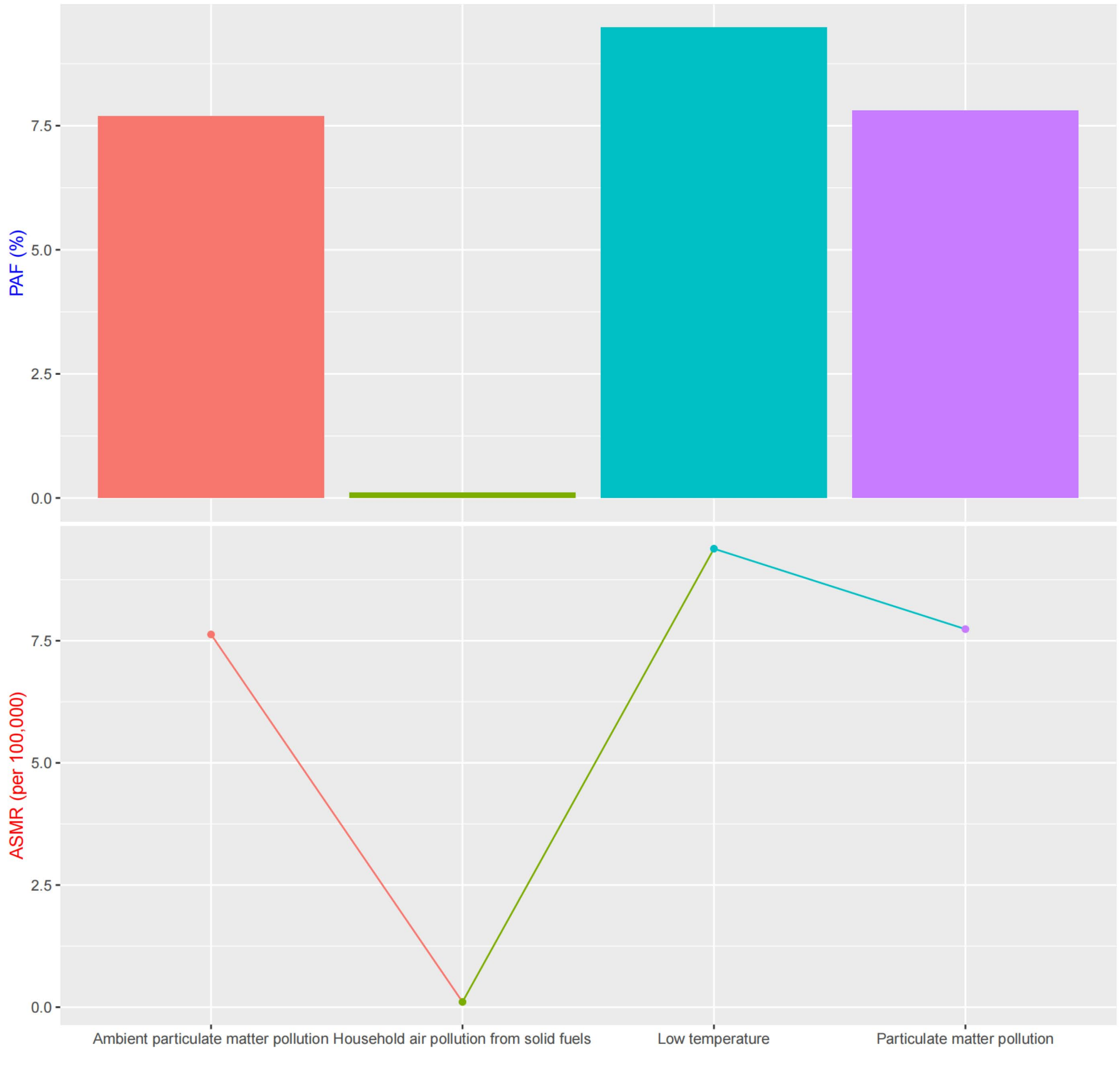

H

Dual X-axis plot illustrating the burden of ischemic stroke death attributable to four environmental/occupational risk factors in Ukraine

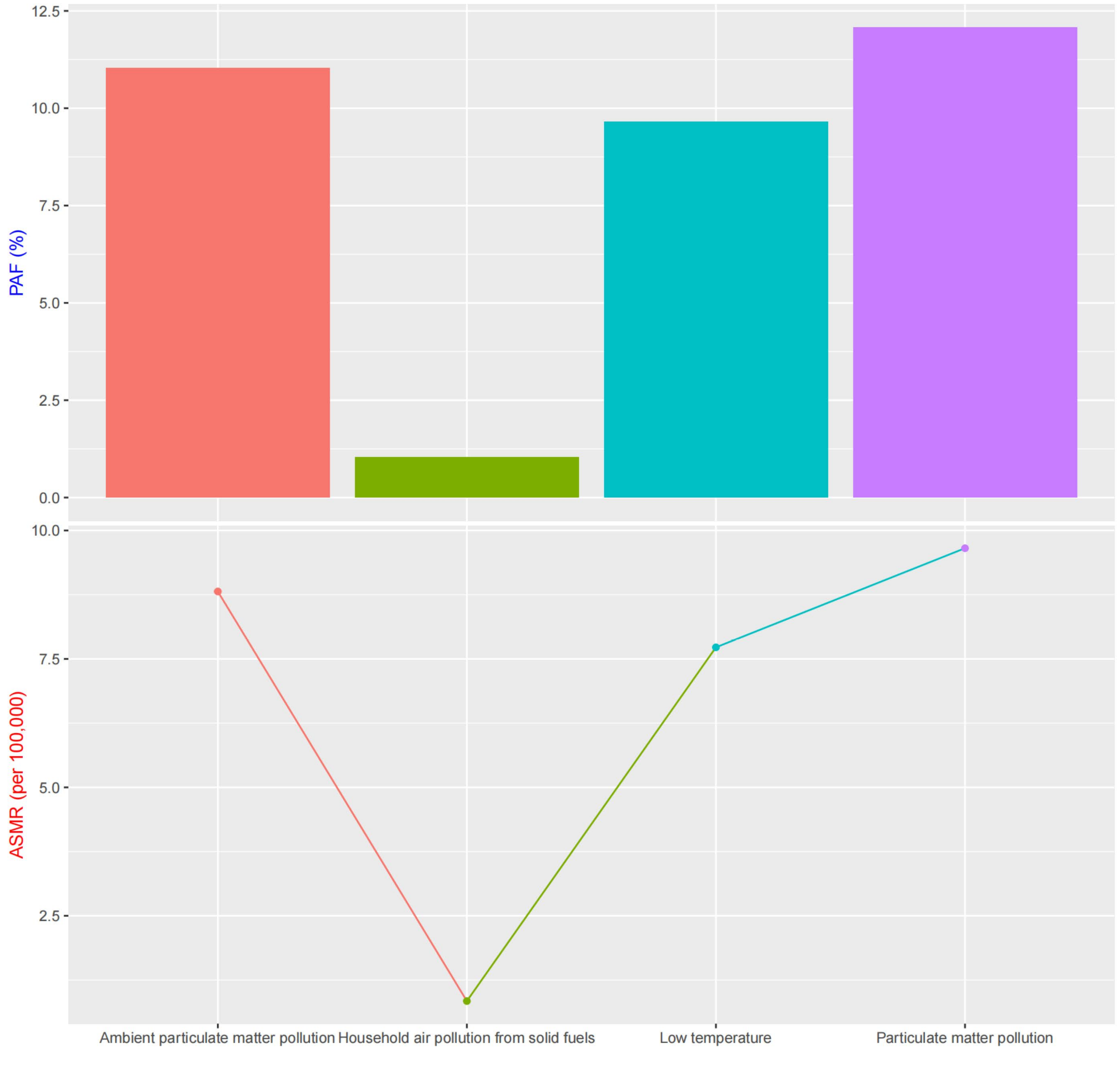

Supplementary Figure 5. Dual X-axis plot illustrating the burden of ischemic stroke death attributable to four environmental/occupational risk factors in Eastern European Countries: A. Dual X-axis plot illustrating the burden of ischemic stroke death attributable to four environmental/occupational risk factors in Eastern Europe; B. Dual X-axis plot illustrating the burden of ischemic stroke death attributable to four environmental/occupational risk factors in Belarus; C. Dual X-axis plot illustrating the burden of ischemic stroke death attributable to four environmental/occupational risk factors in Estonia; D. Dual X-axis plot illustrating the burden of ischemic stroke death attributable to four environmental/occupational risk factors in Latvia; E. Dual X-axis plot illustrating the burden of ischemic stroke death attributable to four environmental/occupational risk factors in Lithuania; F. Dual X-axis plot illustrating the burden of ischemic stroke death attributable to four environmental/occupational risk factors in Republic of Moldova; G. Dual X-axis plot illustrating the burden of ischemic stroke death attributable to four environmental/occupational risk factors in Russian Federation; H. Dual X-axis plot illustrating the burden of ischemic stroke death attributable to four environmental/occupational risk factors in Ukraine. Four environmental/occupational risk factors: particulate matter pollution, ambient particulate matter pollution, household air pollution from solid fuels and low temperatures.

A

Dual X-axis plot illustrating the burden of ischemic stroke death attributable to eight behavioral risk factors in Eastern Europe

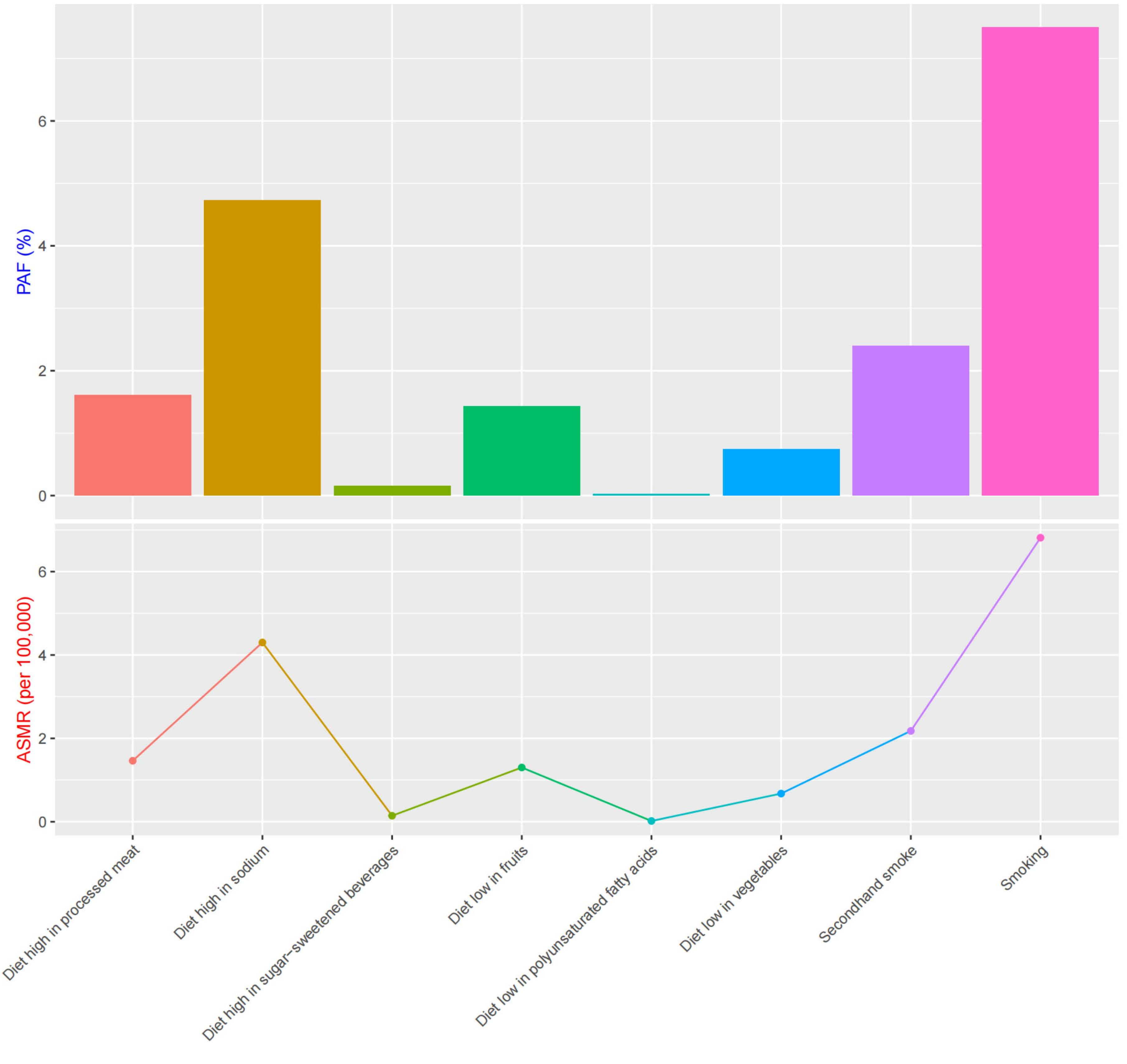

B

Dual X-axis plot illustrating the burden of ischemic stroke death attributable to eight behavioral risk factors in Belarus

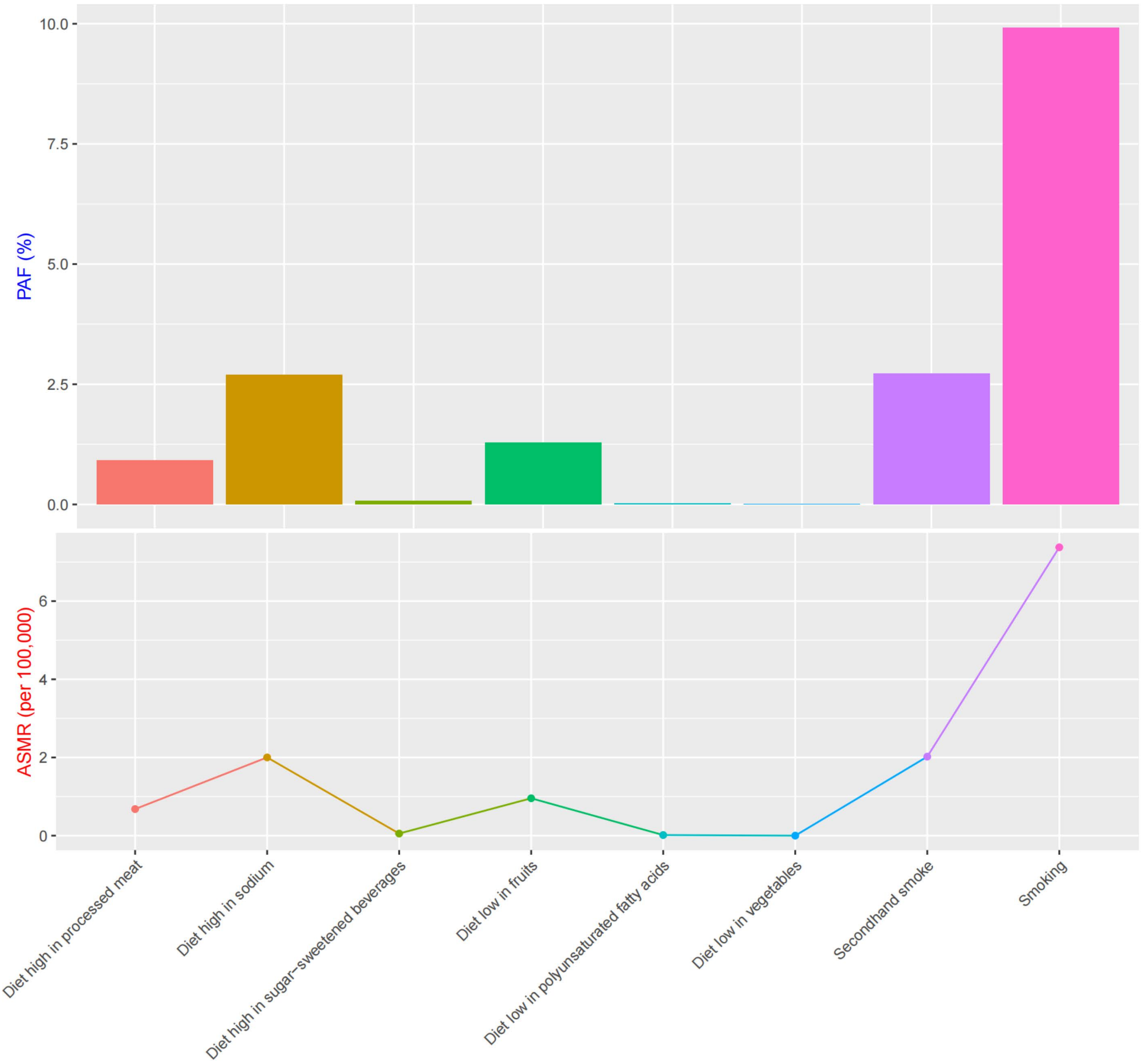

C

Dual X-axis plot illustrating the burden of ischemic stroke death attributable to eight behavioral risk factors in Estonia

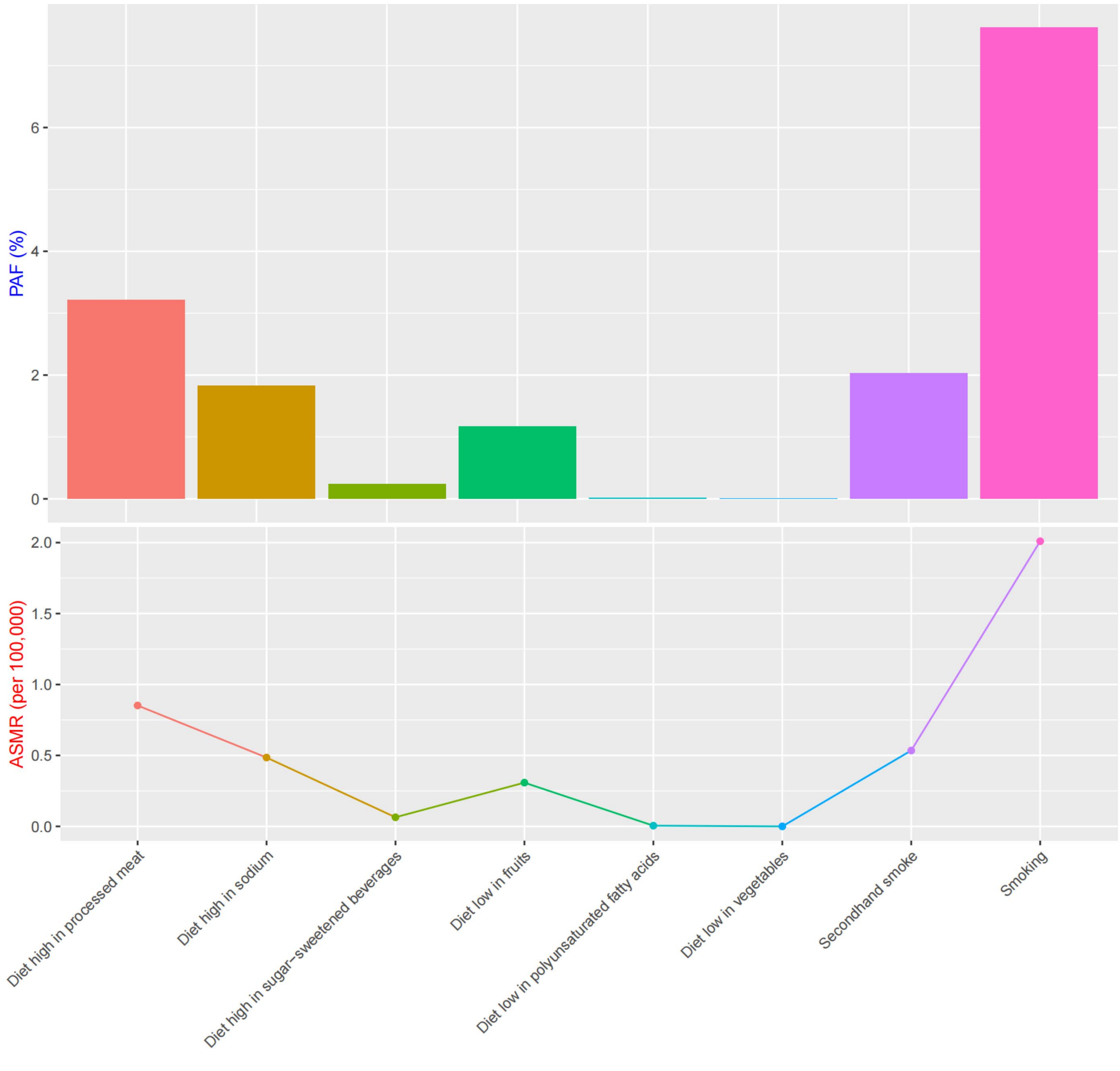

D

Dual X-axis plot illustrating the burden of ischemic stroke death attributable to eight behavioral risk factors in Latvia

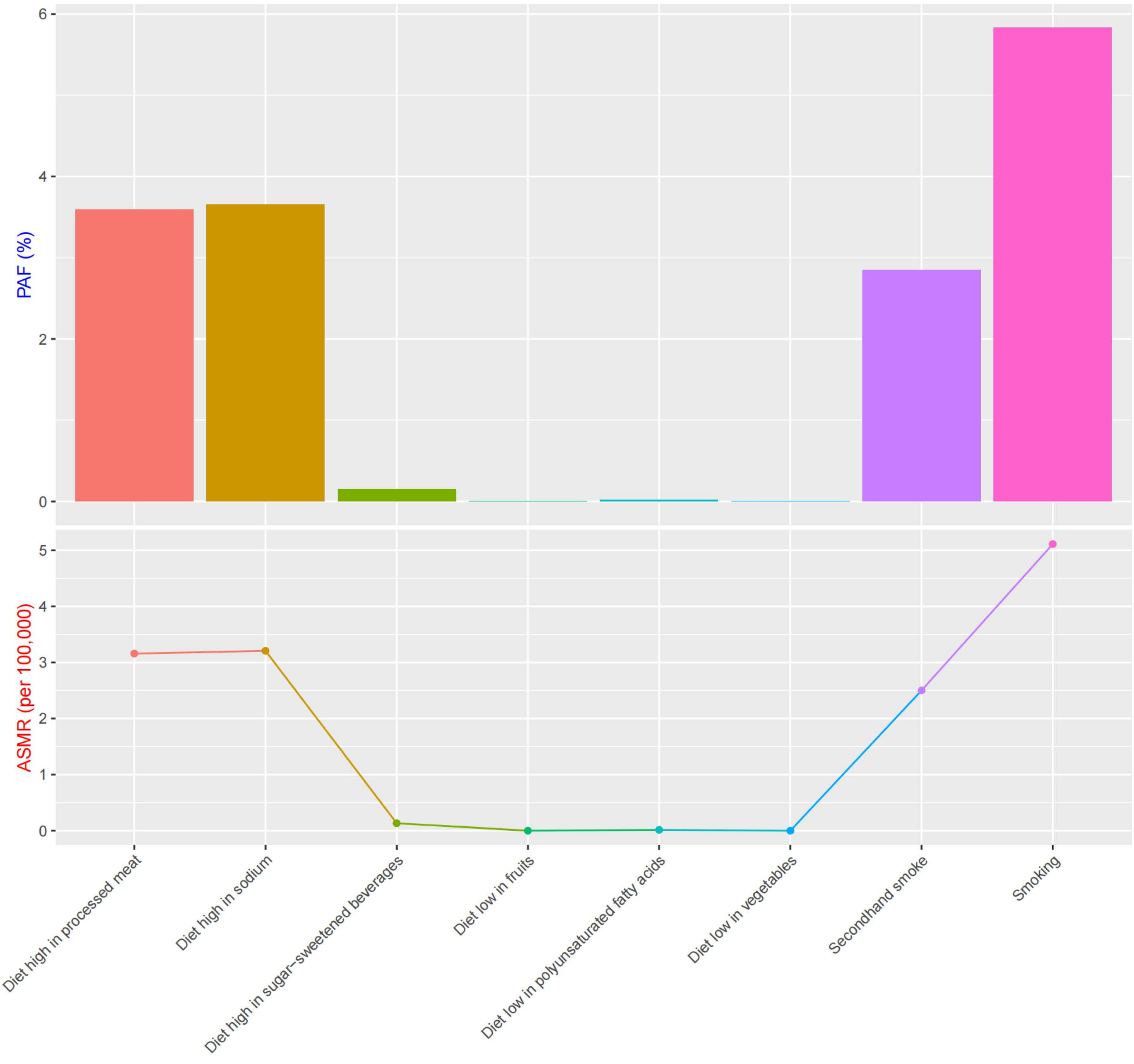

E

Dual X-axis plot illustrating the burden of ischemic stroke death attributable to eight behavioral risk factors in Lithuania

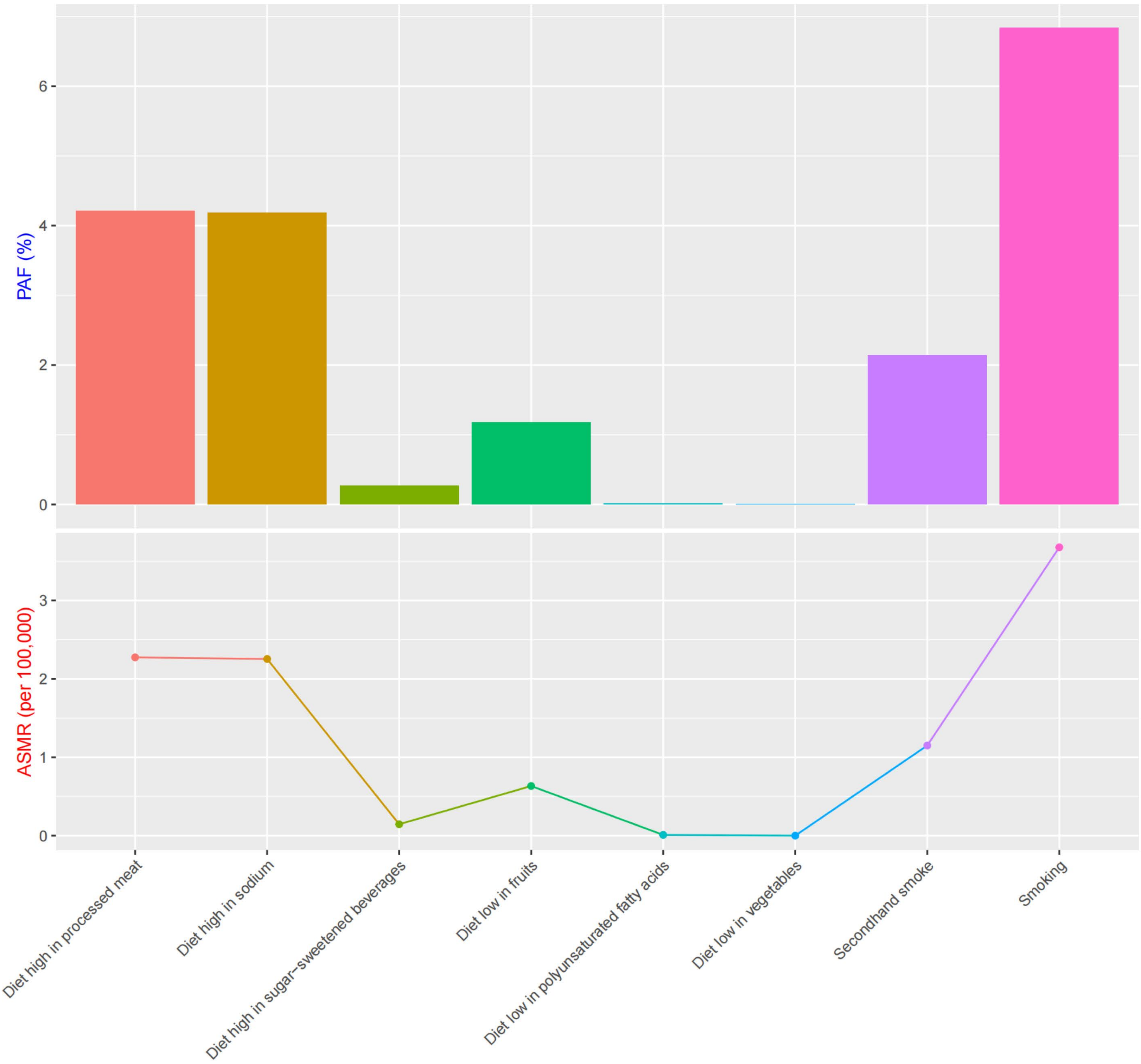

F

Dual X-axis plot illustrating the burden of ischemic stroke death attributable to eight behavioral risk factors in Republic of Moldova

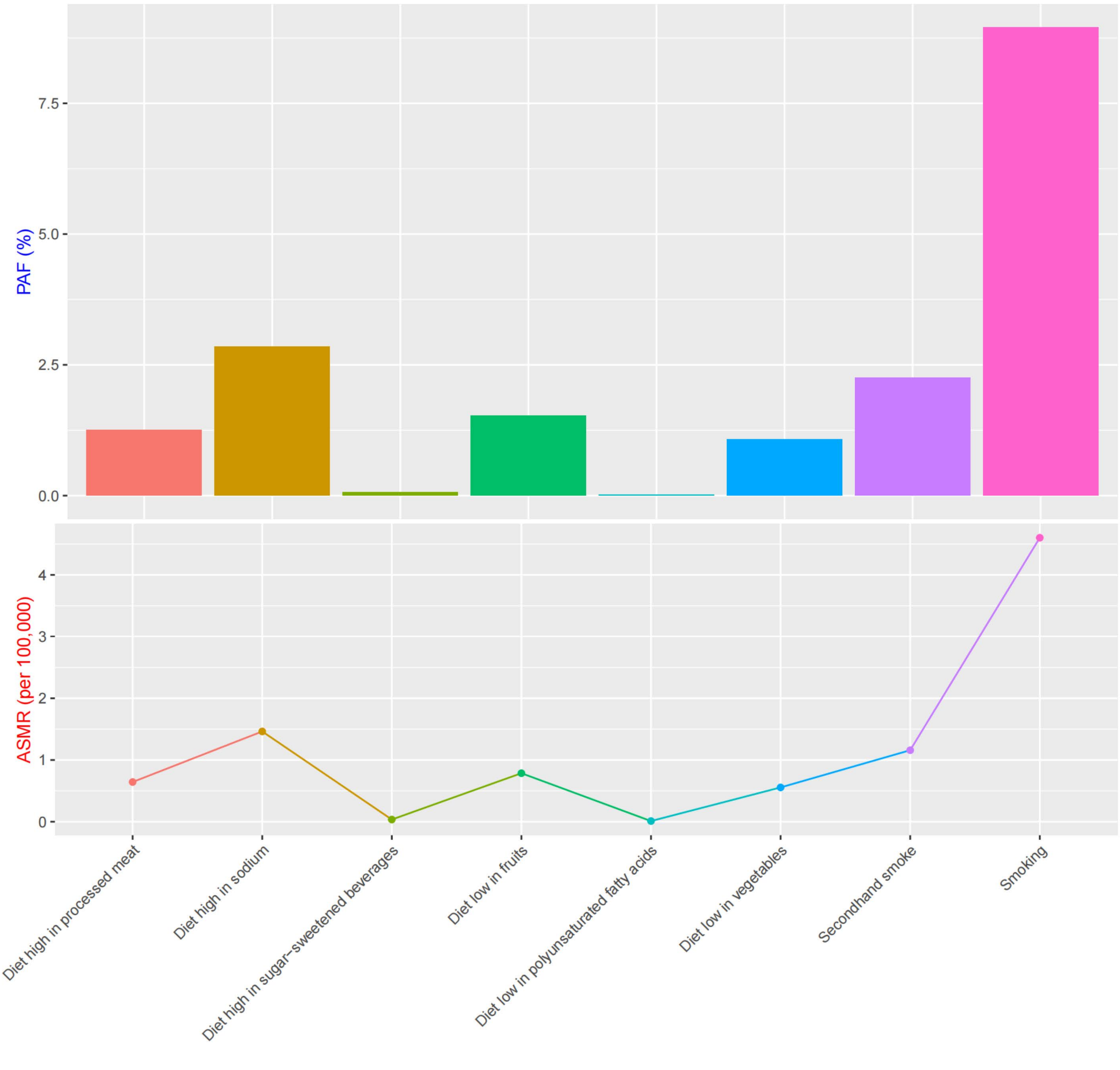

G

Dual X-axis plot illustrating the burden of ischemic stroke death attributable to eight behavioral risk factors in Russian Federation

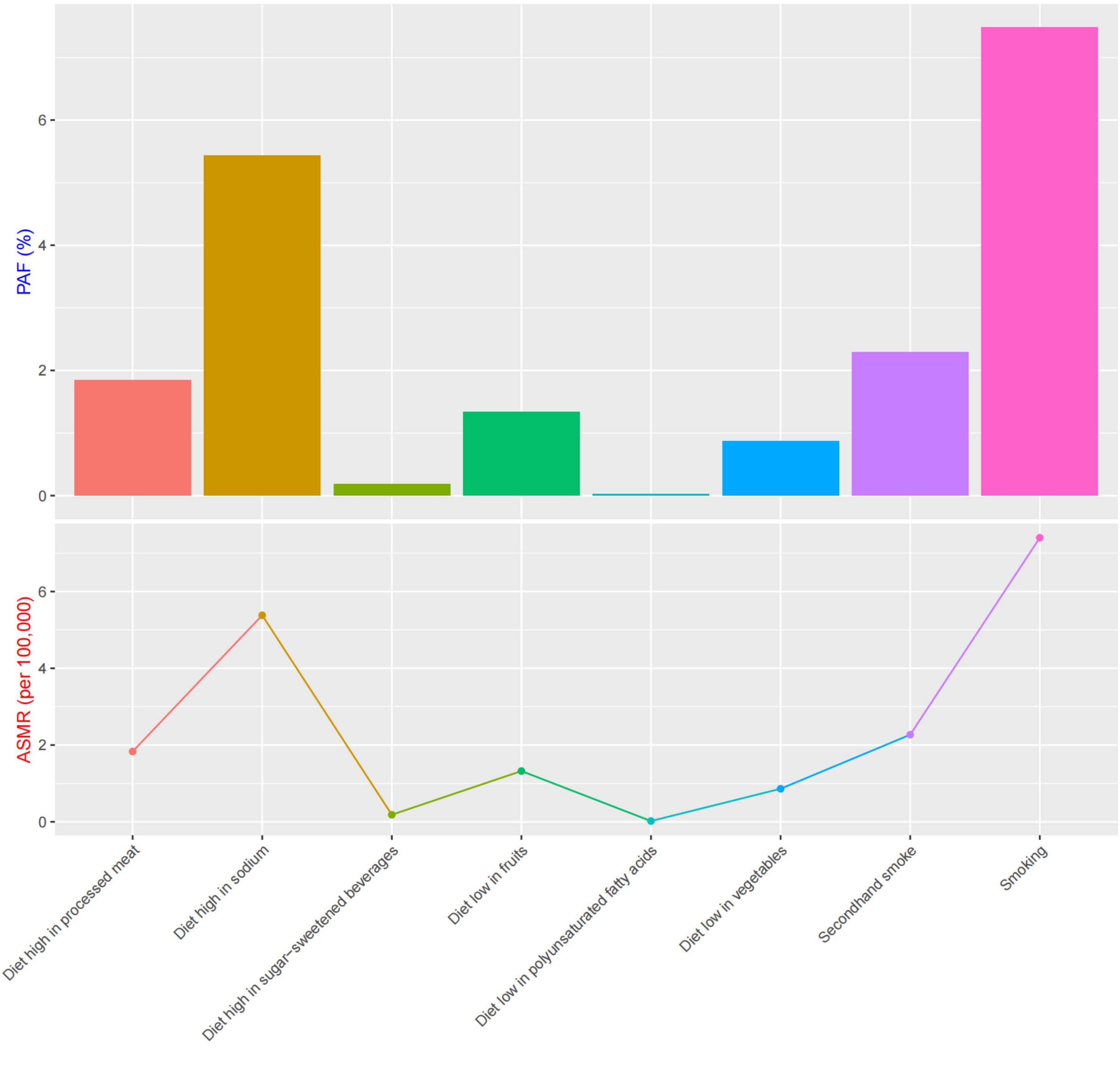

H

Dual X-axis plot illustrating the burden of ischemic stroke death attributable to eight behavioral risk factors in Ukraine

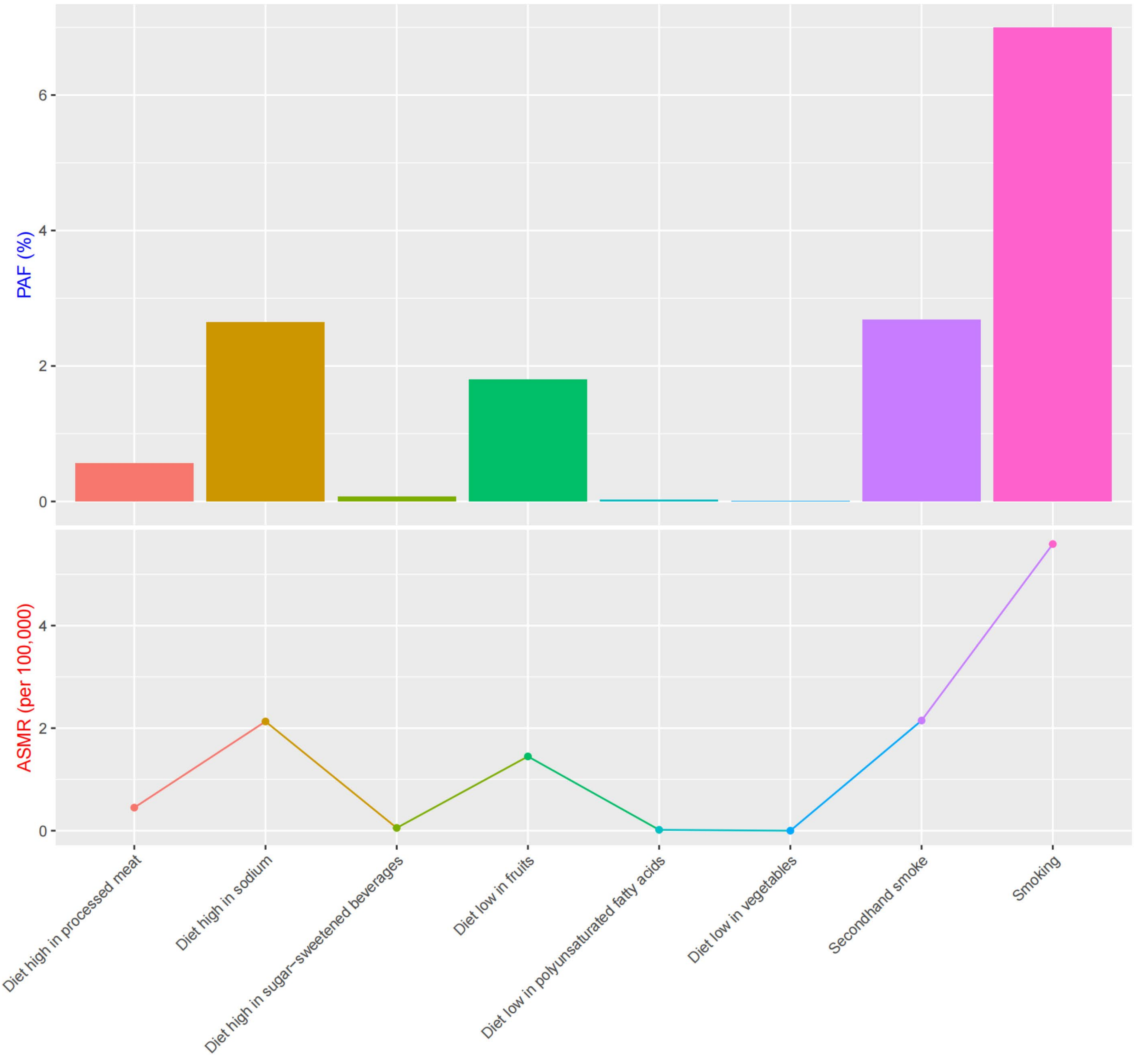

Supplementary Figure 6. Dual X-axis plot illustrating the burden of ischemic stroke death attributable to eight behavioral risk factors in Eastern European Countries: A. Dual X-axis plot illustrating the burden of ischemic stroke death attributable to eight behavioral risk factors in Eastern Europe; B. Dual X-axis plot illustrating the burden of ischemic stroke death attributable to eight behavioral risk factors in Belarus; C. Dual X-axis plot illustrating the burden of ischemic stroke death attributable to eight behavioral risk factors in Estonia; D. Dual X-axis plot illustrating the burden of ischemic stroke death attributable to eight behavioral risk factors in Latvia; E. Dual X-axis plot illustrating the burden of ischemic stroke death attributable to eight behavioral risk factors in Lithuania; F. Dual X-axis plot illustrating the burden of ischemic stroke death attributable to eight behavioral risk factors in Republic of Moldova; G. Dual X-axis plot illustrating the burden of ischemic stroke death attributable to eight behavioral risk factors in Russian Federation; H. Dual X-axis plot illustrating the burden of ischemic stroke death attributable to eight behavioral risk factors in Ukraine. Eight behavioral risk factors: smoking, secondhand smoke, diet high in sodium, diet low in vegetables and fruits, diet high in processed meat, diet high in sugar-sweetened beverages, and diet low in polyunsaturated fatty acids.
