# Supplementary material for: The burden of ischemic stroke in Eastern Europe from 1990 to 2021
Source: BMC Neurol. 2025 Feb 22;25:74. doi: 10.1186/s12883-025-04081-z (PMC11846382; doi:10.1186/s12883-025-04081-z)
Supplement: Supplementary file 2 — Supplementary Material 2 [file 12883_2025_4081_MOESM2_ESM.zip › Supplementary Table 1-10/Supplementary Table 1.docx]

Supplementary Table 1. Geographical Socio-Demographic Index Categories (2021 Values).

| **Country name** | **SDI quintile** |
| --- | --- |
| Afghanistan | Low SDI |
| Albania | Middle SDI |
| Algeria | Middle SDI |
| American Samoa | High-middle SDI |
| Andorra | High SDI |
| Angola | Low-middle SDI |
| Antigua and Barbuda | High-middle SDI |
| Argentina | High-middle SDI |
| Armenia | Middle SDI |
| Australia | High SDI |
| Austria | High SDI |
| Azerbaijan | Middle SDI |
| Bahamas | High-middle SDI |
| Bahrain | High-middle SDI |
| Bangladesh | Low-middle SDI |
| Barbados | High-middle SDI |
| Belarus | High-middle SDI |
| Belgium | High SDI |
| Belize | Low-middle SDI |
| Benin | Low SDI |
| Bermuda | High SDI |
| Bhutan | Low-middle SDI |
| Bolivia (Plurinational State of) | Low-middle SDI |
| Bosnia and Herzegovina | High-middle SDI |
| Botswana | Middle SDI |
| Brazil | Middle SDI |
| Brunei Darussalam | High-middle SDI |
| Bulgaria | High-middle SDI |
| Burkina Faso | Low SDI |
| Burundi | Low SDI |
| Cabo Verde | Low-middle SDI |
| Cambodia | Low-middle SDI |
| Cameroon | Low-middle SDI |
| Canada | High SDI |
| Central African Republic | Low SDI |
| Chad | Low SDI |
| Chile | High-middle SDI |
| China | High-middle SDI |
| Colombia | Middle SDI |
| Comoros | Low-middle SDI |
| Congo | Low-middle SDI |
| Cook Islands | High-middle SDI |
| Costa Rica | Middle SDI |
| Croatia | High-middle SDI |
| Cuba | Middle SDI |
| Cyprus | High SDI |
| Czechia | High SDI |
| Côte d'Ivoire | Low SDI |
| Democratic People's Republic of Korea | Low-middle SDI |
| Democratic Republic of the Congo | Low SDI |
| Denmark | High SDI |
| Djibouti | Low-middle SDI |
| Dominica | High-middle SDI |
| Dominican Republic | Middle SDI |
| Ecuador | Middle SDI |
| Egypt | Low-middle SDI |
| El Salvador | Low-middle SDI |
| Equatorial Guinea | Middle SDI |
| Eritrea | Low SDI |
| Estonia | High SDI |
| Eswatini | Low-middle SDI |
| Ethiopia | Low SDI |
| Fiji | Middle SDI |
| Finland | High SDI |
| France | High SDI |
| Gabon | Middle SDI |
| Gambia | Low SDI |
| Georgia | High-middle SDI |
| Germany | High SDI |
| Ghana | Low-middle SDI |
| Greece | High-middle SDI |
| Greenland | High SDI |
| Grenada | Middle SDI |
| Guam | High-middle SDI |
| Guatemala | Low-middle SDI |
| Guinea | Low SDI |
| Guinea-Bissau | Low SDI |
| Guyana | Middle SDI |
| Haiti | Low SDI |
| Honduras | Low-middle SDI |
| Hungary | High-middle SDI |
| Iceland | High SDI |
| India | Low-middle SDI |
| Indonesia | Middle SDI |
| Iran (Islamic Republic of) | Middle SDI |
| Iraq | Middle SDI |
| Ireland | High SDI |
| Israel | High-middle SDI |
| Italy | High-middle SDI |
| Jamaica | Middle SDI |
| Japan | High SDI |
| Jordan | High-middle SDI |
| Kazakhstan | High-middle SDI |
| Kenya | Low-middle SDI |
| Kiribati | Low-middle SDI |
| Kuwait | High SDI |
| Kyrgyzstan | Low-middle SDI |
| Lao People's Democratic Republic | Low-middle SDI |
| Latvia | High SDI |
| Lebanon | High-middle SDI |
| Lesotho | Low-middle SDI |
| Liberia | Low SDI |
| Libya | High-middle SDI |
| Lithuania | High SDI |
| Luxembourg | High SDI |
| Madagascar | Low SDI |
| Malawi | Low SDI |
| Malaysia | High-middle SDI |
| Maldives | Middle SDI |
| Mali | Low SDI |
| Malta | High-middle SDI |
| Marshall Islands | Low-middle SDI |
| Mauritania | Low-middle SDI |
| Mauritius | High-middle SDI |
| Mexico | Middle SDI |
| Micronesia (Federated States of) | Low-middle SDI |
| Monaco | High SDI |
| Mongolia | Low-middle SDI |
| Montenegro | High-middle SDI |
| Morocco | Low-middle SDI |
| Mozambique | Low SDI |
| Myanmar | Low-middle SDI |
| Namibia | Low-middle SDI |
| Nauru | Middle SDI |
| Nepal | Low SDI |
| Netherlands | High SDI |
| New Zealand | High SDI |
| Nicaragua | Low-middle SDI |
| Niger | Low SDI |
| Nigeria | Low-middle SDI |
| Niue | High-middle SDI |
| North Macedonia | High-middle SDI |
| Northern Mariana Islands | High-middle SDI |
| Norway | High SDI |
| Oman | High-middle SDI |
| Pakistan | Low-middle SDI |
| Palau | High-middle SDI |
| Palestine | Middle SDI |
| Panama | Middle SDI |
| Papua New Guinea | Low SDI |
| Paraguay | Middle SDI |
| Peru | Middle SDI |
| Philippines | Middle SDI |
| Poland | High SDI |
| Portugal | High-middle SDI |
| Puerto Rico | High SDI |
| Qatar | High SDI |
| Republic of Korea | High SDI |
| Republic of Moldova | High-middle SDI |
| Romania | High-middle SDI |
| Russian Federation | High-middle SDI |
| Rwanda | Low SDI |
| Saint Kitts and Nevis | High-middle SDI |
| Saint Lucia | Middle SDI |
| Saint Vincent and the Grenadines | Middle SDI |
| Samoa | Low-middle SDI |
| San Marino | High SDI |
| Sao Tome and Principe | Low-middle SDI |
| Saudi Arabia | High SDI |
| Senegal | Low SDI |
| Serbia | High-middle SDI |
| Seychelles | High-middle SDI |
| Sierra Leone | Low SDI |
| Singapore | High SDI |
| Slovakia | High-middle SDI |
| Slovenia | High SDI |
| Solomon Islands | Low SDI |
| Somalia | Low SDI |
| South Africa | Middle SDI |
| South Sudan | Low SDI |
| Spain | High-middle SDI |
| Sri Lanka | Middle SDI |
| Sudan | Low-middle SDI |
| Suriname | Middle SDI |
| Sweden | High SDI |
| Switzerland | High SDI |
| Syrian Arab Republic | Middle SDI |
| Taiwan (Province of China) | High SDI |
| Tajikistan | Low-middle SDI |
| Thailand | Middle SDI |
| Timor-Leste | Low SDI |
| Togo | Low SDI |
| Tokelau | Middle SDI |
| Tonga | Middle SDI |
| Trinidad and Tobago | High-middle SDI |
| Tunisia | Middle SDI |
| Turkey | High-middle SDI |
| Turkmenistan | Middle SDI |
| Tuvalu | Low-middle SDI |
| Uganda | Low SDI |
| Ukraine | High-middle SDI |
| United Arab Emirates | High SDI |
| United Kingdom | High SDI |
| United Republic of Tanzania | Low SDI |
| United States Virgin Islands | High SDI |
| United States of America | High SDI |
| Uruguay | High-middle SDI |
| Uzbekistan | Middle SDI |
| Vanuatu | Low-middle SDI |
| Venezuela (Bolivarian Republic of) | Low-middle SDI |
| Viet Nam | Middle SDI |
| Yemen | Low SDI |
| Zambia | Low-middle SDI |
| Zimbabwe | Low-middle SDI |
