# Supplementary material for: The burden of ischemic stroke in Eastern Europe from 1990 to 2021
Source: BMC Neurol. 2025 Feb 22;25:74. doi: 10.1186/s12883-025-04081-z (PMC11846382; doi:10.1186/s12883-025-04081-z)
Supplement: Supplementary file 2 — Supplementary Material 2 [file 12883_2025_4081_MOESM2_ESM.zip › Supplementary Table 1-10/Supplementary Table 10.docx]

Supplementary Table 10. Country-specific burden of ischemic stroke attributable to environmental and behavioral risk factors in Eastern Europe: Population attributable fraction and age-standardized mortality rates.

| **Risk Factor** | **Location** | **PAF (%)** | **ASMR (95% UI)** |
| --- | --- | --- | --- |
| **Environmental risks** |  |  |  |
|  | Eastern Europe | 19.81 (15.13 to 24.53) | 18.03 (13.65 to 22.65) |
|  | Belarus | 22.93 (17.70 to 27.94) | 17.06 (12.39 to 21.60) |
|  | Estonia | 14.93 (11.03 to 19.46) | 3.94 (2.75 to 5.29) |
|  | Latvia | 20.77 (16.23 to 26.03) | 18.23 (13.83 to 22.55) |
|  | Lithuania | 18.52 (14.47 to 22.42) | 9.98 (7.59 to 12.39) |
|  | Republic of Moldova | 25.43 (18.92 to 32.32) | 13.09 (9.75 to 16.92) |
|  | Russian Federation | 18.88 (14.30 to 23.58) | 18.72 (13.86 to 23.81) |
|  | Ukraine | 22.46 (16.63 to 28.57) | 17.98 (12.44 to 25.00) |
| Particulate matter pollution |  |  |  |
|  | Eastern Europe | 8.82 (5.56 to 13.20) | 8.03 (5.02 to 12.17) |
|  | Belarus | 11.26 (8.11 to 15.29) | 8.38 (5.80 to 11.66) |
|  | Estonia | 3.19 (0.99 to 7.19) | 0.84 (0.27 to 1.81) |
|  | Latvia | 8.93 (5.82 to 14.69) | 7.83 (5.01 to 12.27) |
|  | Lithuania | 6.36 (3.98 to 9.42) | 3.43 (2.12 to 5.14) |
|  | Republic of Moldova | 14.86 (9.73 to 21.68) | 7.65 (4.93 to 11.16) |
|  | Russian Federation | 7.81 (4.71 to 12.05) | 7.74 (4.65 to 12.19) |
|  | Ukraine | 12.07 (7.05 to 18.47) | 9.65 (5.54 to 15.65) |
| Ambient particulate matter pollution |  |  |  |
|  | Eastern Europe | 8.47 (5.47 to 12.72) | 7.71 (4.89 to 11.64) |
|  | Belarus | 11.19 (8.07 to 15.09) | 8.32 (5.80 to 11.55) |
|  | Estonia | 2.97 (0.99 to 5.31) | 0.78 (0.27 to 1.39) |
|  | Latvia | 8.42 (5.63 to 11.58) | 7.39 (4.88 to 10.35) |
|  | Lithuania | 6.27 (3.93 to 9.05) | 3.38 (2.08 to 5.06) |
|  | Republic of Moldova | 9.01 (4.21 to 15.06) | 4.63 (2.17 to 7.86) |
|  | Russian Federation | 7.69 (4.68 to 11.79) | 7.63 (4.62 to 11.76) |
|  | Ukraine | 11.02 (6.49 to 17.29) | 8.81 (5.13 to 14.66) |
| Household air pollution from solid fuels |  |  |  |
|  | Eastern Europe | 0.35 (0.05 to 1.63) | 0.32 (0.04 to 1.49) |
|  | Belarus | 0.07 (0.00 to 0.59) | 0.05 (0.00 to 0.45) |
|  | Estonia | 0.21 (0.00 to 2.33) | 0.06 (0.00 to 0.60) |
|  | Latvia | 0.50 (0.00 to 5.36) | 0.44 (0.00 to 4.73) |
|  | Lithuania | 0.09 (0.00 to 0.86) | 0.05 (0.00 to 0.47) |
|  | Republic of Moldova | 5.85 (2.04 to 11.08) | 3.01 (1.02 to 5.66) |
|  | Russian Federation | 0.11 (0.00 to 0.94) | 0.11 (0.00 to 0.93) |
|  | Ukraine | 1.04 (0.12 to 4.07) | 0.84 (0.09 to 3.17) |
| Low temperature |  |  |  |
|  | Eastern Europe | 9.56 (7.88 to 11.10) | 8.70 (6.91 to 10.27) |
|  | Belarus | 10.24 (7.99 to 11.64) | 7.62 (5.65 to 9.45) |
|  | Estonia | 9.83 (8.52 to 11.07) | 2.60 (2.11 to 3.07) |
|  | Latvia | 10.68 (9.05 to 11.91) | 9.37 (7.65 to 10.81) |
|  | Lithuania | 10.65 (8.69 to 11.91) | 5.74 (4.58 to 6.69) |
|  | Republic of Moldova | 8.83 (7.63 to 10.65) | 4.54 (3.82 to 5.72) |
|  | Russian Federation | 9.47 (7.68 to 11.19) | 9.38 (7.45 to 11.37) |
|  | Ukraine | 9.64 (8.01 to 11.12) | 7.73 (5.85 to 10.05) |
| **Behavioral risks** |  |  |  |
|  | Eastern Europe | 25.11 (12.84 to 37.43) | 22.85 (12.17 to 34.26) |
|  | Belarus | 27.67 (14.90 to 41.59) | 20.59 (10.73 to 31.71) |
|  | Estonia | 25.02 (13.04 to 39.27) | 6.61 (3.26 to 10.24) |
|  | Latvia | 26.20 (12.90 to 40.55) | 22.99 (11.06 to 36.16) |
|  | Lithuania | 28.21 (13.82 to 43.32) | 15.20 (7.47 to 23.27) |
|  | Republic of Moldova | 27.62 (15.69 to 41.51) | 14.21 (8.06 to 21.76) |
|  | Russian Federation | 25.45 (12.91 to 37.97) | 25.21 (13.47 to 37.73) |
|  | Ukraine | 22.67 (10.82 to 35.07) | 18.17 (8.32 to 28.81) |
| Smoking |  |  |  |
|  | Eastern Europe | 7.49 (6.20 to 9.03) | 6.81 (5.57 to 8.21) |
|  | Belarus | 9.91 (8.24 to 11.83) | 7.38 (5.59 to 9.36) |
|  | Estonia | 7.61 (6.24 to 9.40) | 2.01 (1.59 to 2.53) |
|  | Latvia | 5.83 (4.68 to 7.08) | 5.11 (3.97 to 6.44) |
|  | Lithuania | 6.83 (5.48 to 8.38) | 3.68 (2.92 to 4.62) |
|  | Republic of Moldova | 8.95 (7.40 to 10.96) | 4.60 (3.69 to 5.71) |
|  | Russian Federation | 7.48 (6.28 to 9.07) | 7.40 (6.08 to 8.89) |
|  | Ukraine | 6.99 (4.93 to 9.28) | 5.59 (3.72 to 7.72) |
| Secondhand smoke |  |  |  |
|  | Eastern Europe | 2.39 (1.57 to 3.27) | 2.18 (1.43 to 3.02) |
|  | Belarus | 2.72 (1.80 to 3.71) | 2.02 (1.26 to 2.83) |
|  | Estonia | 2.03 (1.29 to 2.83) | 0.53 (0.34 to 0.77) |
|  | Latvia | 2.85 (1.80 to 3.98) | 2.50 (1.58 to 3.49) |
|  | Lithuania | 2.14 (1.39 to 2.99) | 1.15 (0.74 to 1.61) |
|  | Republic of Moldova | 2.25 (1.50 to 3.09) | 1.16 (0.77 to 1.63) |
|  | Russian Federation | 2.29 (1.50 to 3.16) | 2.27 (1.49 to 3.13) |
|  | Ukraine | 2.68 (1.76 to 3.71) | 2.15 (1.35 to 3.19) |
| Diet high in sodium |  |  |  |
|  | Eastern Europe | 4.73 (0.18 to 14.87) | 4.30 (0.16 to 13.27) |
|  | Belarus | 2.69 (0.00 to 10.82) | 2.00 (0.00 to 8.54) |
|  | Estonia | 1.83 (0.00 to 8.62) | 0.49 (0.00 to 2.31) |
|  | Latvia | 3.65 (0.05 to 12.50) | 3.21 (0.04 to 11.26) |
|  | Lithuania | 4.18 (0.01 to 14.52) | 2.25 (0.00 to 7.84) |
|  | Republic of Moldova | 2.85 (0.00 to 11.63) | 1.46 (0.00 to 5.93) |
|  | Russian Federation | 5.43 (0.23 to 15.99) | 5.38 (0.23 to 15.95) |
|  | Ukraine | 2.64 (0.00 to 10.83) | 2.13 (0.00 to 9.05) |
| Diet low in vegetables |  |  |  |
|  | Eastern Europe | 0.74 (0.01 to 1.43) | 0.68 (0.01 to 1.32) |
|  | Belarus | NA | NA |
|  | Estonia | NA | NA |
|  | Latvia | NA | NA |
|  | Lithuania | NA | NA |
|  | Republic of Moldova | 1.08 (0.27 to 1.94) | 0.56 (0.13 to 1.00) |
|  | Russian Federation | 0.87 (0.04 to 1.67) | 0.86 (0.04 to 1.65) |
|  | Ukraine | NA | NA |
| Diet low in fruits |  |  |  |
|  | Eastern Europe | 1.43 (0.06 to 2.83) | 1.30 (0.06 to 2.57) |
|  | Belarus | 1.28 (0.16 to 2.37) | 0.96 (0.12 to 1.87) |
|  | Estonia | 1.17 (0.03 to 2.38) | 0.31 (0.01 to 0.63) |
|  | Latvia | NA | NA |
|  | Lithuania | 1.17 (0.04 to 2.32) | 0.63 (0.02 to 1.27) |
|  | Republic of Moldova | 1.53 (0.48 to 2.63) | 0.79 (0.23 to 1.36) |
|  | Russian Federation | 1.33 (0.04 to 2.68) | 1.32 (0.04 to 2.67) |
|  | Ukraine | 1.80 (0.12 to 3.56) | 1.45 (0.08 to 2.94) |
| Diet high in processed meat |  |  |  |
|  | Eastern Europe | 1.61 (0.37 to 2.87) | 1.46 (0.33 to 2.64) |
|  | Belarus | 0.91 (0.21 to 1.65) | 0.68 (0.15 to 1.24) |
|  | Estonia | 3.22 (0.76 to 5.94) | 0.85 (0.21 to 1.57) |
|  | Latvia | 3.59 (0.74 to 6.86) | 3.16 (0.65 to 6.10) |
|  | Lithuania | 4.21 (0.94 to 7.71) | 2.27 (0.47 to 4.32) |
|  | Republic of Moldova | 1.25 (0.29 to 2.23) | 0.64 (0.16 to 1.12) |
|  | Russian Federation | 1.85 (0.42 to 3.33) | 1.83 (0.41 to 3.29) |
|  | Ukraine | 0.56 (0.14 to 1.03) | 0.45 (0.11 to 0.85) |
| Diet high in sugar-sweetened beverages |  |  |  |
|  | Eastern Europe | 0.16 (0.08 to 0.26) | 0.14 (0.07 to 0.23) |
|  | Belarus | 0.07 (0.03 to 0.13) | 0.05 (0.02 to 0.09) |
|  | Estonia | 0.24 (0.11 to 0.40) | 0.06 (0.03 to 0.11) |
|  | Latvia | 0.15 (0.07 to 0.27) | 0.13 (0.06 to 0.24) |
|  | Lithuania | 0.27 (0.12 to 0.46) | 0.15 (0.06 to 0.26) |
|  | Republic of Moldova | 0.07 (0.03 to 0.12) | 0.04 (0.02 to 0.06) |
|  | Russian Federation | 0.19 (0.09 to 0.31) | 0.18 (0.09 to 0.30) |
|  | Ukraine | 0.07 (0.03 to 0.11) | 0.05 (0.03 to 0.09) |
| Diet low in polyunsaturated fatty acids |  |  |  |
|  | Eastern Europe | 0.02 (0.01 to 0.04) | 0.02 (0.00 to 0.04) |
|  | Belarus | 0.02 (0.01 to 0.04) | 0.02 (0.00 to 0.03) |
|  | Estonia | 0.02 (0.01 to 0.04) | 0.00 (0.00 to 0.01) |
|  | Latvia | 0.02 (0.00 to 0.03) | 0.01 (0.00 to 0.03) |
|  | Lithuania | 0.02 (0.00 to 0.03) | 0.01 (0.00 to 0.02) |
|  | Republic of Moldova | 0.02 (0.01 to 0.04) | 0.01 (0.00 to 0.02) |
|  | Russian Federation | 0.02 (0.01 to 0.04) | 0.02 (0.01 to 0.04) |
|  | Ukraine | 0.02 (0.01 to 0.04) | 0.02 (0.00 to 0.03) |

PAF, Population attributable fraction; ASMR, age-standardized mortality rate; 95% UI: 95% uncertainty interval.
