# Supplementary material for: The burden of ischemic stroke in Eastern Europe from 1990 to 2021
Source: BMC Neurol. 2025 Feb 22;25:74. doi: 10.1186/s12883-025-04081-z (PMC11846382; doi:10.1186/s12883-025-04081-z)
Supplement: Supplementary file 2 — Supplementary Material 2 [file 12883_2025_4081_MOESM2_ESM.zip › Supplementary Table 1-10/Supplementary Table 2.docx]

Supplementary Table 2. Global, regional, and national trends in ischemic stroke deaths from 1990 to 2021: Detailed comparisons of death counts, age-standardized mortality rates, percentage changes, and estimated annual percentage change.

| **Location** | **1990** | | **2021** | | **1990-2021** | |
| --- | --- | --- | --- | --- | --- | --- |
|  | **Deaths Cases**  **(95% UI)** | **ASMR**  **(95% UI)** | **Deaths Cases**  **(95% UI)** | **ASMR**  **(95% UI)** | **Cases**  **change**  **(%,95% UI)** | **EAPC**  **(95% CI)** |
| **Global** | 2317112 (2131460 to 2475546) | 73.15 (66.36 to 77.94) | 3591499 (3213281 to 3888327) | 44.18 (39.29 to 47.81) | 55.00 (43.20 to 66.76) | -1.83 (-1.92 to -1.74) |
| **Regions** |  |  |  |  |  |  |
| High SDI | 595528 (535847 to 623760) | 53.85 (48.15 to 56.57) | 507950 (426777 to 553062) | 19.42 (16.54 to 21.03) | -14.71 (-22.05 to -10.53) | -3.58 (-3.71 to -3.44) |
| High-middle SDI | 887525 (831447 to 925960) | 112.05 (103.37 to 116.96) | 1151655 (1025972 to 1263429) | 59.75 (52.99 to 65.45) | 29.76 (18.62 to 42.59) | -2.4 (-2.6 to -2.2) |
| Middle SDI | 484059 (438723 to 543563) | 66.56 (59.51 to 74.59) | 1171548 (1037070 to 1296280) | 51.64 (45.40 to 57.09) | 142.03 (108.63 to 178.27) | -0.86 (-0.95 to -0.76) |
| Low-middle SDI | 258771 (227017 to 300182) | 58.98 (51.59 to 67.65) | 581649 (517711 to 657998) | 50.90 (45.37 to 57.00) | 124.77 (101.68 to 153.09) | -0.49 (-0.53 to -0.44) |
| Low SDI | 87275 (72465 to 110946) | 57.07 (47.48 to 72.01) | 174655 (149333 to 216891) | 49.38 (42.13 to 60.35) | 100.12 (76.13 to 127.97) | -0.48 (-0.54 to -0.42) |
| Eastern Europe | 405262 (383950 to 415284) | 168.09 (157.84 to 173) | 329291 (299911 to 356035) | 90.99 (82.79 to 98.48) | -18.75 (-24.17 to -13.28) | -2.78 (-3.24 to -2.32) |
| **Country** |  |  |  |  |  |  |
| Belarus | 12842 (11796 to 13644) | 104.04 (95.76 to 110.78) | 12332 (10422 to 14334) | 74.42 (63.09 to 86.47) | -3.97 (-17.87 to 11.99) | -1.74 (-2.12 to -1.36) |
| Estonia | 2703 (2524 to 2846) | 136.94 (127.64 to 144.38) | 886 (754 to 994) | 26.43 (22.60 to 29.58) | -67.23 (-71.21 to -63.40) | -6.83 (-7.44 to -6.22) |
| Latvia | 5182 (4873 to 5470) | 148.41 (139.01 to 156.85) | 4304 (3770 to 4748) | 87.79 (77.02 to 96.90) | -16.95 (-25.46 to -7.76) | -2.16 (-2.42 to -1.89) |
| Lithuania | 3411 (3182 to 3612) | 76.26 (71.02 to 80.79) | 3783 (3338 to 4184) | 53.88 (47.72 to 59.62) | 10.90 (-1.87 to 22.32) | -1.39 (-1.7 to -1.07) |
| Republic of Moldova | 2749 (2491 to 2984) | 87.03 (78.62 to 94.10) | 3133 (2830 to 3459) | 51.45 (46.56 to 56.79) | 13.96 (1.17 to 31.13) | -1.47 (-1.9 to -1.04) |
| Russian Federation | 280039 (265350 to 286551) | 185.67 (174.06 to 191.00) | 240445 (218161 to 258644) | 99.09 (89.81 to 106.59) | -14.14 (-19.82 to -8.68) | -2.88 (-3.41 to -2.35) |
| Ukraine | 98337 (93099 to 102439) | 154.39 (144.51 to 161.04) | 64409 (50727 to 79552) | 80.12 (63.20 to 99.30) | -34.50 (-48.12 to -18.76) | -2.86 (-3.18 to -2.54) |

ASMR, age-standardized mortality rate; EAPC, estimated annual percentage change; SDI, Socio-Demographic Index. 95% UI: 95% uncertainty interval. 95% CI: 95% confidence interval.
