# Supplementary material for: The burden of ischemic stroke in Eastern Europe from 1990 to 2021
Source: BMC Neurol. 2025 Feb 22;25:74. doi: 10.1186/s12883-025-04081-z (PMC11846382; doi:10.1186/s12883-025-04081-z)
Supplement: Supplementary file 2 — Supplementary Material 2 [file 12883_2025_4081_MOESM2_ESM.zip › Supplementary Table 1-10/Supplementary Table 3.docx]

Supplementary Table 3. Global, regional, and national trends in the burden of ischemic stroke from 1990 to 2021: Disability-adjusted life years, age-standardized DALYs rates, percentage changes, and estimated annual percentage change.

| **Location** | **1990** | | **2021** | | **1990-2021** | |
| --- | --- | --- | --- | --- | --- | --- |
|  | **DALYs Cases**  **(95% UI)** | **ASDR**  **(95% UI)** | **DALYs Cases**  **(95% UI)** | **ASDR**  **(95% UI)** | **Cases change (%,95% UI)** | **EAPC**  **(95% CI)** |
| **Global** | 46176240 (42961948 to 49414586) | 1286.31 (1195.19 to 1376.06) | 70357912 (64329576 to 76007063) | 837.36 (763.73 to 904.98) | 52.37 (41.05 to 64.32) | -1.59 (-1.68 to -1.5) |
| **Regions** |  |  |  |  |  |  |
| High SDI | 10239252 (9461539 to 10819068) | 916.44 (845.06 to 969.25) | 8975176 (7913835 to 9812024) | 395.56 (352.17 to 434.79) | -12.35 (-16.97 to -8.13) | -2.98 (-3.11 to -2.85) |
| High-middle SDI | 16876355 (15868635 to 17770724) | 1885.84 (1768.07 to 1980.92) | 21054343 (19044778 to 23026603) | 1076.54 (973.38 to 1176.25) | 24.76 (14.23 to 36.75) | -2.19 (-2.39 to -1.99) |
| Middle SDI | 10950854 (9944440 to 12298162) | 1221.85 (1103.17 to 1364.82) | 23896857 (21522553 to 26137545) | 960.71 (863.98 to 1047.82) | 118.22 (90.97 to 148.07) | -0.83 (-0.89 to -0.76) |
| Low-middle SDI | 5912050 (5266484 to 6751650) | 1094.89 (975.43 to 1254.57) | 12300862 (11013179 to 13998937) | 942.27 (846.11 to 1065.86) | 108.06 (86.51 to 132.35) | -0.52 (-0.55 to -0.48) |
| Low SDI | 2126089 (1791117 to 2645820) | 1074.89 (910.35 to 1345.18) | 4059455 (3492447 to 4963612) | 914.28 (789.76 to 1116.62) | 90.94 (67.92 to 115.12) | -0.58 (-0.63 to -0.53) |
| Eastern Europe | 7422390 (7152142 to 7643098) | 2825.98 (2711.24 to 2913.4) | 5713718 (5294961 to 6142848) | 1601.2 (1483.51 to 1723.12) | -23.02 (-27.81 to -17.93) | -2.61 (-3.06 to -2.16) |
| **Country** |  |  |  |  |  |  |
| Belarus | 253688 (233403 to 270306) | 1987.30 (1825.11 to 2119.29) | 226406 (192635 to 263057) | 1386.51 (1178.06 to 1609.28) | -10.75 (-23.55 to 3.94) | -1.88 (-2.31 to -1.45) |
| Estonia | 48300 (45570 to 50921) | 2372.94 (2233.67 to 2496.56) | 14986 (13200 to 16732) | 514.34 (453.80 to 575.43) | -68.97 (-72.53 to -65.82) | -6.3 (-6.84 to -5.77) |
| Latvia | 89466 (84421 to 94601) | 2504.41 (2362.79 to 2647.56) | 65466 (58444 to 72264) | 1458.59 (1298.88 to 1616.34) | -26.83 (-34.18 to -19.11) | -2.21 (-2.46 to -1.97) |
| Lithuania | 65985 (61468 to 70244) | 1466.44 (1364.60 to 1561.54) | 60850 (54067 to 67264) | 969.78 (863.90 to 1072.11) | -7.78 (-17.69 to 1.64) | -1.25 (-1.56 to -0.93) |
| Republic of Moldova | 52664 (47423 to 57435) | 1418.74 (1286.09 to 1541.33) | 63140 (57401 to 69442) | 1045.92 (950.27 to 1148.48) | 19.89 (6.63 to 37.40) | -0.75 (-1.17 to -0.34) |
| Russian Federation | 5134684 (4942960 to 5276175) | 3081.00 (2948.28 to 3170.61) | 4128008 (3829112 to 4445427) | 1713.22 (1588.50 to 1846.08) | -19.61 (-24.47 to -14.52) | -2.75 (-3.27 to -2.22) |
| Ukraine | 1777604 (1682460 to 1859726) | 2593.14 (2442.04 to 2713.40) | 1154862 (915982 to 1417130) | 1462.06 (1160.17 to 1796.03) | -35.03 (-47.85 to -20.37) | -2.5 (-2.78 to -2.22) |

DALYs, Disability-adjusted life years; ASDR, age-standardized DALYs rate; EAPC, estimated annual percentage change; SDI, Socio-Demographic Index. 95% UI: 95% uncertainty interval. 95% CI: 95% confidence interval.
