# Supplementary material for: The burden of ischemic stroke in Eastern Europe from 1990 to 2021
Source: BMC Neurol. 2025 Feb 22;25:74. doi: 10.1186/s12883-025-04081-z (PMC11846382; doi:10.1186/s12883-025-04081-z)
Supplement: Supplementary file 2 — Supplementary Material 2 [file 12883_2025_4081_MOESM2_ESM.zip › Supplementary Table 1-10/Supplementary Table 4.docx]

Supplementary Table 4. Trends in ischemic stroke incidence by year, age group, and gender in Eastern European countries (1990-2021): Case counts and incidence rates (per 100,000 population) for different demographic groups.

| **Location** | **Age** | **Year** | **Incidence cases**  **(95% UI)** | | | **Incidence rate (1/100000)**  **(95% UI)** | | |
| --- | --- | --- | --- | --- | --- | --- | --- | --- |
|  |  |  | **Both** | **Male** | **Female** | **Both** | **Male** | **Female** |
| Belarus |  |  |  |  |  |  |  |  |
|  | 0-14 years |  |  |  |  |  |  |  |
|  |  | 1990 year | 162 (82 to 282) | 60 (25 to 113) | 101 (52 to 174) | 6.72 (3.40 to 11.71) | 4.92 (2.07 to 9.17) | 8.60 (4.42 to 14.80) |
|  |  | 2000 year | 115 (55 to 197) | 37 (15 to 65) | 78 (39 to 134) | 5.94 (2.87 to 10.23) | 3.71 (1.51 to 6.53) | 8.28 (4.17 to 14.21) |
|  |  | 2010 year | 84 (42 to 143) | 26 (11 to 46) | 57 (29 to 96) | 5.80 (2.90 to 9.90) | 3.55 (1.52 to 6.18) | 8.18 (4.20 to 13.64) |
|  |  | 2021 year | 91 (42 to 167) | 33 (13 to 62) | 59 (29 to 103) | 5.79 (2.68 to 10.58) | 4.01 (1.61 to 7.68) | 7.67 (3.74 to 13.48) |
|  |  | Rate of change（%） | -43.50 (-51.93 to -34.58) | -45.95 (-60.75 to -28.68) | -42.03 (-51.12 to -32.54) | NA | NA | NA |
|  | 15-49 years |  |  |  |  |  |  |  |
|  |  | 1990 year | 2005 (1588 to 2554) | 1083 (865 to 1385) | 922 (706 to 1220) | 39.77 (31.50 to 50.65) | 43.19 (34.49 to 55.20) | 36.39 (27.85 to 48.14) |
|  |  | 2000 year | 2208 (1790 to 2723) | 1313 (1054 to 1613) | 894 (701 to 1158) | 41.20 (33.40 to 50.82) | 49.31 (39.57 to 60.54) | 33.19 (26.00 to 42.96) |
|  |  | 2010 year | 1989 (1628 to 2419) | 1178 (941 to 1449) | 811 (630 to 1039) | 39.95 (32.71 to 48.59) | 47.57 (38.00 to 58.51) | 32.41 (25.20 to 41.55) |
|  |  | 2021 year | 1567 (1253 to 2015) | 871 (681 to 1126) | 697 (536 to 921) | 36.84 (29.44 to 47.37) | 40.81 (31.93 to 52.74) | 32.84 (25.29 to 43.43) |
|  |  | Rate of change（%） | -21.84 (-29.57 to -12.47) | -19.61 (-31.58 to -4.87) | -24.46 (-33.82 to -13.57) | NA | NA | NA |
|  | 50-74 years |  |  |  |  |  |  |  |
|  |  | 1990 year | 13443 (11045 to 16460) | 6149 (4848 to 7625) | 7294 (5795 to 8988) | 532.96 (437.90 to 652.56) | 593.54 (467.96 to 736.01) | 490.73 (389.88 to 604.68) |
|  |  | 2000 year | 15525 (12887 to 18301) | 7895 (6617 to 9336) | 7630 (6091 to 9256) | 627.39 (520.77 to 739.55) | 764.91 (641.09 to 904.45) | 528.97 (422.27 to 641.70) |
|  |  | 2010 year | 13357 (11243 to 15414) | 7497 (6155 to 8923) | 5860 (4796 to 7014) | 508.04 (427.62 to 586.26) | 672.73 (552.28 to 800.70) | 386.88 (316.59 to 463.03) |
|  |  | 2021 year | 12168 (10004 to 14653) | 6546 (5331 to 8095) | 5622 (4514 to 6921) | 414.44 (340.74 to 499.08) | 523.16 (426.04 to 646.97) | 333.70 (267.90 to 410.78) |
|  |  | Rate of change（%） | -9.49 (-20.41 to 1.51) | 6.45 (-10.74 to 28.23) | -22.92 (-34.70 to -10.98) | NA | NA | NA |
|  | 75+ years |  |  |  |  |  |  |  |
|  |  | 1990 year | 7655 (6216 to 9209) | 2002 (1600 to 2437) | 5654 (4498 to 6941) | 1608.67 (1306.29 to 1935.04) | 1543.80 (1234.18 to 1879.07) | 1632.96 (1299.19 to 2004.73) |
|  |  | 2000 year | 7279 (6160 to 8532) | 1913 (1565 to 2291) | 5366 (4512 to 6544) | 1608.13 (1360.90 to 1884.97) | 1653.76 (1353.00 to 1980.89) | 1592.47 (1338.92 to 1941.83) |
|  |  | 2010 year | 9507 (7743 to 11060) | 2715 (2196 to 3203) | 6793 (5459 to 8134) | 1573.22 (1281.35 to 1830.14) | 1660.20 (1343.00 to 1958.66) | 1540.96 (1238.44 to 1845.34) |
|  |  | 2021 year | 8360 (6877 to 9905) | 2014 (1588 to 2428) | 6346 (5150 to 7809) | 1504.24 (1237.44 to 1782.17) | 1398.20 (1101.97 to 1685.52) | 1541.35 (1250.88 to 1896.79) |
|  |  | Rate of change（%） | 9.20 (-4.73 to 27.45) | 0.63 (-14.48 to 18.75) | 12.24 (-5.05 to 35.89) | NA | NA | NA |
| Estonia |  |  |  |  |  |  |  |  |
|  | 0-14 years |  |  |  |  |  |  |  |
|  |  | 1990 year | 18 (9 to 31) | 8 (4 to 14) | 10 (5 to 17) | 5.18 (2.51 to 8.85) | 4.64 (2.18 to 7.76) | 5.74 (2.75 to 9.93) |
|  |  | 2000 year | 9 (5 to 14) | 4 (2 to 6) | 5 (3 to 9) | 3.65 (1.97 to 5.73) | 3.10 (1.68 to 4.85) | 4.24 (2.21 to 7.24) |
|  |  | 2010 year | 8 (4 to 14) | 3 (1 to 5) | 5 (2 to 9) | 3.92 (1.88 to 6.91) | 2.88 (1.29 to 5.00) | 5.03 (2.30 to 9.02) |
|  |  | 2021 year | 10 (5 to 18) | 4 (1 to 7) | 6 (3 to 11) | 4.59 (2.09 to 8.31) | 3.40 (1.28 to 6.26) | 5.84 (2.53 to 10.68) |
|  |  | Rate of change（%） | -45.12 (-56.76 to -36.67) | -54.27 (-72.04 to -41.96) | -37.44 (-50.54 to -25.43) | NA | NA | NA |
|  | 15-49 years |  |  |  |  |  |  |  |
|  |  | 1990 year | 299 (250 to 359) | 179 (150 to 215) | 120 (95 to 152) | 39.37 (32.86 to 47.26) | 47.25 (39.70 to 56.91) | 31.54 (24.96 to 39.81) |
|  |  | 2000 year | 245 (208 to 289) | 150 (127 to 177) | 95 (77 to 118) | 35.57 (30.20 to 41.97) | 43.93 (37.08 to 51.77) | 27.32 (22.24 to 34.14) |
|  |  | 2010 year | 173 (137 to 220) | 92 (72 to 118) | 80 (61 to 108) | 26.88 (21.37 to 34.20) | 28.42 (22.15 to 36.26) | 25.31 (19.09 to 34.10) |
|  |  | 2021 year | 143 (107 to 195) | 69 (51 to 93) | 75 (55 to 104) | 24.98 (18.69 to 33.99) | 23.18 (17.16 to 31.50) | 26.90 (19.81 to 37.52) |
|  |  | Rate of change（%） | -52.03 (-61.24 to -39.24) | -61.66 (-69.96 to -51.17) | -37.70 (-50.60 to -19.75) | NA | NA | NA |
|  | 50-74 years |  |  |  |  |  |  |  |
|  |  | 1990 year | 1729 (1537 to 1969) | 869 (769 to 1000) | 860 (723 to 1019) | 454.90 (404.31 to 517.94) | 556.09 (491.90 to 639.72) | 384.24 (322.86 to 455.19) |
|  |  | 2000 year | 1571 (1395 to 1742) | 844 (755 to 949) | 728 (622 to 838) | 414.74 (368.33 to 459.81) | 535.38 (478.89 to 602.14) | 328.83 (281.33 to 378.55) |
|  |  | 2010 year | 1037 (871 to 1237) | 571 (454 to 690) | 466 (374 to 566) | 269.45 (226.40 to 321.47) | 349.95 (278.29 to 422.44) | 210.12 (168.59 to 255.49) |
|  |  | 2021 year | 818 (651 to 1023) | 460 (357 to 584) | 358 (278 to 453) | 206.94 (164.57 to 258.78) | 261.56 (202.95 to 332.16) | 163.19 (126.75 to 206.25) |
|  |  | Rate of change（%） | -52.67 (-59.64 to -44.70) | -47.06 (-56.82 to -35.04) | -58.34 (-65.39 to -49.96) | NA | NA | NA |
|  | 75+ years |  |  |  |  |  |  |  |
|  |  | 1990 year | 1337 (1133 to 1558) | 323 (271 to 384) | 1014 (843 to 1190) | 1672.61 (1417.34 to 1948.75) | 1535.22 (1287.71 to 1822.23) | 1721.71 (1431.64 to 2020.50) |
|  |  | 2000 year | 1046 (917 to 1191) | 252 (215 to 289) | 794 (691 to 912) | 1385.95 (1215.00 to 1577.15) | 1332.36 (1137.78 to 1529.48) | 1403.87 (1220.85 to 1611.07) |
|  |  | 2010 year | 953 (770 to 1134) | 255 (206 to 310) | 699 (554 to 852) | 923.07 (745.55 to 1098.16) | 896.70 (724.40 to 1090.78) | 933.06 (739.11 to 1137.35) |
|  |  | 2021 year | 917 (739 to 1106) | 247 (195 to 304) | 669 (522 to 819) | 732.02 (590.26 to 882.70) | 683.69 (539.73 to 840.40) | 751.65 (586.50 to 919.02) |
|  |  | Rate of change（%） | -31.45 (-40.98 to -19.23) | -23.45 (-38.64 to -4.62) | -34.00 (-44.19 to -19.98) | NA | NA | NA |
| Latvia |  |  |  |  |  |  |  |  |
|  | 0-14 years |  |  |  |  |  |  |  |
|  |  | 1990 year | 29 (13 to 52) | 11 (5 to 21) | 17 (8 to 31) | 5.03 (2.34 to 9.21) | 3.92 (1.67 to 7.28) | 6.18 (2.96 to 10.99) |
|  |  | 2000 year | 21 (9 to 38) | 8 (3 to 16) | 12 (5 to 23) | 4.79 (2.01 to 8.90) | 3.70 (1.35 to 7.13) | 5.92 (2.46 to 11.14) |
|  |  | 2010 year | 14 (6 to 25) | 5 (2 to 10) | 9 (4 to 15) | 4.71 (2.12 to 8.47) | 3.52 (1.33 to 6.52) | 5.95 (2.83 to 10.41) |
|  |  | 2021 year | 13 (6 to 24) | 5 (2 to 9) | 8 (4 to 15) | 4.44 (1.99 to 7.93) | 3.32 (1.24 to 6.08) | 5.62 (2.59 to 10.17) |
|  |  | Rate of change（%） | -53.92 (-61.56 to -46.62) | -55.22 (-67.92 to -36.73) | -53.07 (-60.90 to -45.88) | NA | NA | NA |
|  | 15-49 years |  |  |  |  |  |  |  |
|  |  | 1990 year | 521 (417 to 666) | 276 (218 to 348) | 245 (190 to 317) | 40.53 (32.47 to 51.85) | 43.34 (34.15 to 54.64) | 37.77 (29.25 to 48.95) |
|  |  | 2000 year | 392 (325 to 470) | 214 (178 to 259) | 177 (140 to 223) | 33.45 (27.76 to 40.17) | 37.04 (30.75 to 44.78) | 29.94 (23.71 to 37.58) |
|  |  | 2010 year | 325 (269 to 401) | 183 (153 to 220) | 142 (111 to 183) | 31.41 (25.93 to 38.65) | 35.48 (29.59 to 42.60) | 27.36 (21.33 to 35.13) |
|  |  | 2021 year | 230 (185 to 286) | 128 (103 to 159) | 103 (79 to 132) | 29.22 (23.44 to 36.25) | 32.02 (25.87 to 39.97) | 26.36 (20.28 to 33.85) |
|  |  | Rate of change（%） | -55.79 (-60.43 to -49.92) | -53.79 (-60.32 to -45.52) | -58.04 (-63.29 to -52.62) | NA | NA | NA |
|  | 50-74 years |  |  |  |  |  |  |  |
|  |  | 1990 year | 3561 (2943 to 4424) | 1692 (1389 to 2075) | 1869 (1488 to 2340) | 536.30 (443.11 to 666.22) | 628.06 (515.51 to 770.14) | 473.66 (377.12 to 592.84) |
|  |  | 2000 year | 3438 (2962 to 3976) | 1770 (1530 to 2071) | 1669 (1378 to 1972) | 525.59 (452.72 to 607.73) | 660.46 (571.10 to 772.75) | 432.02 (356.82 to 510.58) |
|  |  | 2010 year | 3069 (2668 to 3449) | 1686 (1465 to 1928) | 1383 (1140 to 1643) | 494.38 (429.84 to 555.65) | 655.09 (569.16 to 749.12) | 380.52 (313.77 to 452.22) |
|  |  | 2021 year | 2349 (1939 to 2758) | 1288 (1066 to 1535) | 1061 (852 to 1293) | 394.02 (325.29 to 462.74) | 500.42 (414.41 to 596.34) | 313.18 (251.56 to 381.58) |
|  |  | Rate of change（%） | -34.05 (-41.00 to -24.72) | -23.88 (-35.11 to -10.18) | -43.25 (-51.32 to -32.66) | NA | NA | NA |
|  | 75+ years |  |  |  |  |  |  |  |
|  |  | 1990 year | 3112 (2563 to 3745) | 861 (684 to 1069) | 2251 (1850 to 2801) | 2226.94 (1834.43 to 2680.21) | 2204.85 (1751.81 to 2737.94) | 2235.49 (1836.55 to 2781.36) |
|  |  | 2000 year | 2462 (2163 to 2776) | 612 (524 to 703) | 1850 (1603 to 2112) | 1916.36 (1683.76 to 2161.20) | 1939.35 (1663.17 to 2229.88) | 1908.88 (1653.39 to 2179.24) |
|  |  | 2010 year | 2862 (2495 to 3192) | 815 (702 to 945) | 2046 (1769 to 2321) | 1730.99 (1509.35 to 1931.16) | 1857.06 (1598.74 to 2152.04) | 1685.41 (1456.69 to 1911.42) |
|  |  | 2021 year | 3087 (2626 to 3557) | 827 (671 to 1006) | 2260 (1922 to 2668) | 1633.99 (1390.16 to 1882.89) | 1580.14 (1280.71 to 1920.79) | 1654.64 (1407.23 to 1953.84) |
|  |  | Rate of change（%） | -0.80 (-11.06 to 14.41) | -3.87 (-18.22 to 16.12) | 0.38 (-12.84 to 17.42) | NA | NA | NA |
| Lithuania |  |  |  |  |  |  |  |  |
|  | 0-14 years |  |  |  |  |  |  |  |
|  |  | 1990 year | 56 (28 to 100) | 21 (9 to 38) | 35 (17 to 63) | 6.71 (3.33 to 12.09) | 4.96 (2.18 to 9.09) | 8.53 (4.24 to 15.49) |
|  |  | 2000 year | 41 (19 to 72) | 16 (6 to 28) | 26 (12 to 46) | 5.84 (2.74 to 10.25) | 4.31 (1.78 to 7.67) | 7.45 (3.58 to 13.31) |
|  |  | 2010 year | 27 (14 to 49) | 10 (4 to 18) | 17 (9 to 31) | 5.82 (2.90 to 10.31) | 4.13 (1.76 to 7.61) | 7.59 (3.88 to 13.29) |
|  |  | 2021 year | 22 (10 to 40) | 8 (3 to 15) | 14 (7 to 25) | 5.44 (2.55 to 9.72) | 3.87 (1.60 to 7.25) | 7.09 (3.44 to 12.39) |
|  |  | Rate of change（%） | -60.24 (-66.39 to -54.64) | -61.43 (-72.61 to -52.03) | -59.52 (-66.34 to -52.09) | NA | NA | NA |
|  | 15-49 years |  |  |  |  |  |  |  |
|  |  | 1990 year | 866 (718 to 1063) | 438 (353 to 553) | 428 (345 to 530) | 47.33 (39.21 to 58.05) | 48.22 (38.80 to 60.80) | 46.45 (37.42 to 57.51) |
|  |  | 2000 year | 732 (646 to 836) | 365 (321 to 416) | 368 (313 to 431) | 41.45 (36.55 to 47.31) | 41.85 (36.82 to 47.71) | 41.06 (34.97 to 48.14) |
|  |  | 2010 year | 685 (601 to 781) | 357 (318 to 405) | 328 (274 to 388) | 45.38 (39.78 to 51.72) | 47.66 (42.38 to 54.05) | 43.12 (36.09 to 51.02) |
|  |  | 2021 year | 428 (345 to 536) | 225 (180 to 284) | 204 (161 to 263) | 37.06 (29.86 to 46.37) | 38.56 (30.90 to 48.85) | 35.53 (28.15 to 45.94) |
|  |  | Rate of change（%） | -50.59 (-56.35 to -45.00) | -48.76 (-56.02 to -40.17) | -52.46 (-59.30 to -44.40) | NA | NA | NA |
|  | 50-74 years |  |  |  |  |  |  |  |
|  |  | 1990 year | 5258 (4359 to 6144) | 2573 (2097 to 3005) | 2685 (2170 to 3207) | 627.05 (519.85 to 732.69) | 737.60 (601.13 to 861.34) | 548.28 (443.11 to 654.94) |
|  |  | 2000 year | 6147 (5652 to 6590) | 3036 (2791 to 3295) | 3111 (2763 to 3458) | 702.26 (645.69 to 752.91) | 832.90 (765.85 to 904.18) | 609.05 (540.93 to 676.90) |
|  |  | 2010 year | 5907 (5461 to 6339) | 3158 (2909 to 3401) | 2749 (2389 to 3069) | 682.15 (630.66 to 732.08) | 864.42 (796.26 to 930.83) | 549.12 (477.36 to 613.13) |
|  |  | 2021 year | 3988 (3231 to 4796) | 2171 (1739 to 2680) | 1817 (1426 to 2254) | 447.40 (362.50 to 538.05) | 562.29 (450.46 to 694.30) | 359.62 (282.30 to 446.02) |
|  |  | Rate of change（%） | -24.16 (-35.23 to -14.54) | -15.64 (-29.65 to 0.15) | -32.32 (-44.27 to -21.04) | NA | NA | NA |
|  | 75+ years |  |  |  |  |  |  |  |
|  |  | 1990 year | 3280 (2704 to 3833) | 1085 (835 to 1321) | 2195 (1782 to 2601) | 1881.42 (1550.98 to 2198.22) | 1915.31 (1473.81 to 2332.72) | 1865.12 (1513.96 to 2209.44) |
|  |  | 2000 year | 3711 (3396 to 4041) | 1157 (1044 to 1270) | 2555 (2278 to 2833) | 2143.26 (1961.28 to 2333.51) | 2312.17 (2086.49 to 2538.06) | 2074.62 (1850.06 to 2300.37) |
|  |  | 2010 year | 5219 (4679 to 5709) | 1789 (1616 to 1966) | 3430 (2990 to 3839) | 2136.99 (1915.63 to 2337.55) | 2504.28 (2262.42 to 2752.77) | 1985.16 (1730.14 to 2221.58) |
|  |  | 2021 year | 4431 (3646 to 5287) | 1236 (985 to 1532) | 3195 (2627 to 3805) | 1617.88 (1331.30 to 1930.51) | 1553.91 (1238.07 to 1925.88) | 1644.06 (1351.59 to 1957.59) |
|  |  | Rate of change（%） | 35.09 (17.90 to 52.43) | 13.90 (-5.98 to 41.16) | 45.56 (20.02 to 70.78) | NA | NA | NA |
| Republic of Moldova | | |  |  |  |  |  |  |
|  | 0-14 years |  |  |  |  |  |  |  |
|  |  | 1990 year | 61 (27 to 110) | 24 (10 to 45) | 37 (17 to 67) | 4.91 (2.22 to 8.92) | 3.80 (1.64 to 7.08) | 6.06 (2.78 to 10.99) |
|  |  | 2000 year | 46 (20 to 83) | 17 (7 to 31) | 28 (13 to 52) | 4.96 (2.21 to 9.01) | 3.70 (1.52 to 6.60) | 6.28 (2.84 to 11.63) |
|  |  | 2010 year | 30 (14 to 53) | 11 (5 to 20) | 19 (9 to 33) | 5.03 (2.45 to 9.00) | 3.64 (1.50 to 6.61) | 6.49 (3.23 to 11.51) |
|  |  | 2021 year | 26 (11 to 47) | 10 (4 to 19) | 16 (7 to 29) | 4.93 (2.17 to 9.04) | 3.66 (1.43 to 6.99) | 6.27 (2.82 to 11.52) |
|  |  | Rate of change（%） | -57.58 (-62.29 to -51.89) | -58.85 (-68.05 to -48.72) | -56.76 (-61.63 to -51.15) | NA | NA | NA |
|  | 15-49 years |  |  |  |  |  |  |  |
|  |  | 1990 year | 595 (472 to 761) | 290 (226 to 370) | 306 (237 to 393) | 27.10 (21.50 to 34.63) | 27.03 (21.14 to 34.55) | 27.16 (21.10 to 34.94) |
|  |  | 2000 year | 686 (550 to 876) | 362 (292 to 462) | 325 (249 to 424) | 30.32 (24.31 to 38.71) | 32.40 (26.13 to 41.32) | 28.29 (21.73 to 37.01) |
|  |  | 2010 year | 681 (565 to 863) | 375 (304 to 468) | 306 (248 to 395) | 32.77 (27.19 to 41.50) | 36.09 (29.20 to 45.03) | 29.45 (23.83 to 38.07) |
|  |  | 2021 year | 566 (449 to 712) | 309 (242 to 394) | 257 (199 to 330) | 31.61 (25.09 to 39.79) | 34.06 (26.65 to 43.39) | 29.09 (22.58 to 37.38) |
|  |  | Rate of change（%） | -4.96 (-16.36 to 8.49) | 6.74 (-12.05 to 28.59) | -16.05 (-25.39 to -3.48) | NA | NA | NA |
|  | 50-74 years |  |  |  |  |  |  |  |
|  |  | 1990 year | 2868 (2322 to 3509) | 1390 (1119 to 1721) | 1478 (1160 to 1844) | 323.71 (262.05 to 395.98) | 367.79 (295.98 to 455.41) | 290.92 (228.26 to 363.02) |
|  |  | 2000 year | 3124 (2511 to 3813) | 1600 (1264 to 1989) | 1524 (1206 to 1867) | 359.97 (289.28 to 439.37) | 428.02 (338.01 to 531.90) | 308.47 (244.05 to 377.94) |
|  |  | 2010 year | 3481 (2929 to 4179) | 1923 (1608 to 2331) | 1558 (1279 to 1893) | 351.28 (295.52 to 421.65) | 444.06 (371.16 to 538.08) | 279.26 (229.28 to 339.34) |
|  |  | 2021 year | 3577 (2852 to 4268) | 1967 (1585 to 2384) | 1610 (1257 to 2014) | 333.66 (266.02 to 398.08) | 423.31 (341.00 to 513.02) | 265.07 (206.96 to 331.56) |
|  |  | Rate of change（%） | 24.72 (8.87 to 48.48) | 41.51 (18.71 to 71.73) | 8.92 (-7.41 to 35.14) | NA | NA | NA |
|  | 75+ years |  |  |  |  |  |  |  |
|  |  | 1990 year | 1631 (1327 to 1972) | 579 (455 to 723) | 1051 (840 to 1272) | 1271.02 (1034.27 to 1536.86) | 1308.66 (1026.94 to 1634.16) | 1251.20 (999.44 to 1513.21) |
|  |  | 2000 year | 1499 (1233 to 1788) | 538 (425 to 654) | 961 (782 to 1190) | 1047.32 (861.07 to 1248.86) | 1155.55 (911.65 to 1403.57) | 995.10 (810.00 to 1232.50) |
|  |  | 2010 year | 2001 (1615 to 2341) | 734 (559 to 893) | 1267 (1011 to 1548) | 1071.34 (864.58 to 1253.31) | 1157.21 (881.27 to 1408.86) | 1027.20 (819.63 to 1254.97) |
|  |  | 2021 year | 2236 (1830 to 2630) | 774 (629 to 934) | 1462 (1184 to 1724) | 1069.33 (875.23 to 1257.90) | 1112.80 (905.16 to 1342.65) | 1047.67 (848.43 to 1235.41) |
|  |  | Rate of change（%） | 37.12 (17.14 to 58.82) | 33.57 (10.13 to 67.32) | 39.08 (16.59 to 65.88) | NA | NA | NA |
| Russian Federation | | |  |  |  |  |  |  |
|  | 0-14 years |  |  |  |  |  |  |  |
|  |  | 1990 year | 2109 (1024 to 3714) | 769 (329 to 1367) | 1340 (678 to 2337) | 6.08 (2.95 to 10.70) | 4.36 (1.86 to 7.74) | 7.86 (3.98 to 13.71) |
|  |  | 2000 year | 1542 (764 to 2678) | 562 (254 to 984) | 980 (503 to 1695) | 5.77 (2.86 to 10.02) | 4.12 (1.86 to 7.20) | 7.50 (3.85 to 12.97) |
|  |  | 2010 year | 1181 (582 to 2031) | 388 (175 to 688) | 793 (408 to 1382) | 5.26 (2.59 to 9.05) | 3.38 (1.52 to 5.98) | 7.25 (3.73 to 12.63) |
|  |  | 2021 year | 1352 (630 to 2432) | 448 (183 to 823) | 905 (441 to 1602) | 5.19 (2.41 to 9.33) | 3.35 (1.37 to 6.15) | 7.12 (3.48 to 12.61) |
|  |  | Rate of change（%） | -35.86 (-41.60 to -30.82) | -41.77 (-55.16 to -30.42) | -32.47 (-37.55 to -28.91) | NA | NA | NA |
|  | 15-49 years |  |  |  |  |  |  |  |
|  |  | 1990 year | 29040 (22404 to 37365) | 14523 (11104 to 18772) | 14518 (11110 to 18925) | 39.13 (30.19 to 50.34) | 39.04 (29.85 to 50.47) | 39.21 (30.01 to 51.11) |
|  |  | 2000 year | 36729 (28547 to 46677) | 19983 (15223 to 25566) | 16746 (12946 to 21509) | 45.52 (35.38 to 57.84) | 49.92 (38.03 to 63.87) | 41.18 (31.83 to 52.89) |
|  |  | 2010 year | 29676 (23562 to 37399) | 16093 (12571 to 20180) | 13582 (10785 to 17256) | 39.41 (31.29 to 49.67) | 43.19 (33.74 to 54.16) | 35.71 (28.35 to 45.37) |
|  |  | 2021 year | 26353 (20642 to 33373) | 13926 (10842 to 17756) | 12427 (9720 to 15871) | 39.08 (30.61 to 49.49) | 41.52 (32.32 to 52.93) | 36.67 (28.68 to 46.82) |
|  |  | Rate of change（%） | -9.25 (-16.31 to -1.29) | -4.11 (-12.33 to 5.73) | -14.40 (-20.68 to -8.23) | NA | NA | NA |
|  | 50-74 years |  |  |  |  |  |  |  |
|  |  | 1990 year | 173290 (131789 to 226576) | 77047 (57888 to 102219) | 96243 (72666 to 126573) | 483.38 (367.61 to 632.02) | 531.28 (399.17 to 704.86) | 450.84 (340.40 to 592.91) |
|  |  | 2000 year | 174225 (134592 to 223167) | 84389 (65372 to 109463) | 89836 (69275 to 116183) | 486.15 (375.56 to 622.72) | 571.26 (442.53 to 741.01) | 426.47 (328.86 to 551.54) |
|  |  | 2010 year | 142049 (112251 to 177698) | 71763 (56842 to 91106) | 70286 (55384 to 89751) | 356.92 (282.05 to 446.49) | 433.48 (343.35 to 550.33) | 302.39 (238.28 to 386.13) |
|  |  | 2021 year | 164903 (128529 to 211956) | 83809 (65375 to 108702) | 81094 (62741 to 105315) | 379.59 (295.86 to 487.91) | 455.99 (355.69 to 591.43) | 323.57 (250.34 to 420.21) |
|  |  | Rate of change（%） | -4.84 (-13.51 to 3.92) | 8.78 (-1.80 to 19.68) | -15.74 (-23.12 to -7.09) | NA | NA | NA |
|  | 75+ years |  |  |  |  |  |  |  |
|  |  | 1990 year | 121497 (89986 to 157329) | 26855 (18542 to 36292) | 94642 (71147 to 122320) | 1954.55 (1447.63 to 2530.98) | 1946.29 (1343.80 to 2630.21) | 1956.90 (1471.10 to 2529.19) |
|  |  | 2000 year | 109246 (85752 to 134890) | 21830 (16268 to 28139) | 87416 (69429 to 108264) | 1835.09 (1440.44 to 2265.85) | 1661.62 (1238.30 to 2141.83) | 1884.22 (1496.51 to 2333.58) |
|  |  | 2010 year | 118450 (92886 to 148028) | 28294 (21214 to 36127) | 90155 (71391 to 111091) | 1515.91 (1188.75 to 1894.45) | 1352.08 (1013.74 to 1726.36) | 1575.84 (1247.86 to 1941.77) |
|  |  | 2021 year | 145101 (116604 to 179755) | 33681 (26129 to 42289) | 111420 (89336 to 138177) | 1837.45 (1476.59 to 2276.29) | 1569.10 (1217.30 to 1970.15) | 1937.63 (1553.58 to 2402.95) |
|  |  | Rate of change（%） | 19.43 (4.81 to 36.60) | 25.42 (11.14 to 45.74) | 17.73 (3.28 to 34.82) | NA | NA | NA |
| Ukraine |  |  |  |  |  |  |  |  |
|  | 0-14 years |  |  |  |  |  |  |  |
|  |  | 1990 year | 860 (420 to 1496) | 324 (143 to 597) | 536 (259 to 920) | 7.56 (3.69 to 13.16) | 5.59 (2.47 to 10.30) | 9.61 (4.65 to 16.48) |
|  |  | 2000 year | 560 (262 to 1012) | 203 (82 to 374) | 358 (174 to 641) | 6.48 (3.03 to 11.71) | 4.58 (1.85 to 8.46) | 8.49 (4.13 to 15.22) |
|  |  | 2010 year | 413 (211 to 735) | 139 (62 to 254) | 273 (142 to 486) | 6.27 (3.20 to 11.16) | 4.12 (1.83 to 7.49) | 8.54 (4.45 to 15.18) |
|  |  | 2021 year | 406 (198 to 731) | 141 (57 to 263) | 265 (130 to 469) | 6.40 (3.12 to 11.52) | 4.32 (1.75 to 8.05) | 8.62 (4.24 to 15.23) |
|  |  | Rate of change（%） | -52.75 (-58.53 to -46.53) | -56.41 (-66.79 to -42.68) | -50.55 (-57.27 to -43.38) | NA | NA | NA |
|  | 15-49 years |  |  |  |  |  |  |  |
|  |  | 1990 year | 11185 (8478 to 14468) | 5820 (4365 to 7615) | 5365 (4036 to 6978) | 44.83 (33.98 to 57.99) | 47.35 (35.51 to 61.95) | 42.38 (31.88 to 55.13) |
|  |  | 2000 year | 13061 (10061 to 16781) | 7492 (5717 to 9677) | 5570 (4174 to 7314) | 51.34 (39.54 to 65.96) | 59.76 (45.61 to 77.20) | 43.15 (32.34 to 56.67) |
|  |  | 2010 year | 10837 (8539 to 13728) | 6314 (4903 to 8137) | 4523 (3457 to 5917) | 46.21 (36.41 to 58.54) | 54.37 (42.21 to 70.06) | 38.21 (29.20 to 49.98) |
|  |  | 2021 year | 10132 (8029 to 12977) | 6099 (4726 to 7832) | 4033 (3054 to 5241) | 50.10 (39.70 to 64.16) | 60.26 (46.70 to 77.38) | 39.91 (30.22 to 51.87) |
|  |  | Rate of change（%） | -9.41 (-19.41 to 1.21) | 4.79 (-9.91 to 21.64) | -24.82 (-33.62 to -15.81) | NA | NA | NA |
|  | 50-74 years |  |  |  |  |  |  |  |
|  |  | 1990 year | 74167 (55289 to 98772) | 34093 (25349 to 46638) | 40074 (30049 to 53739) | 537.76 (400.88 to 716.17) | 604.21 (449.23 to 826.54) | 491.75 (368.74 to 659.44) |
|  |  | 2000 year | 75463 (57472 to 97165) | 37959 (28782 to 49469) | 37503 (28350 to 49738) | 573.35 (436.66 to 738.24) | 692.91 (525.39 to 903.01) | 488.10 (368.97 to 647.34) |
|  |  | 2010 year | 57987 (44948 to 75278) | 29620 (23155 to 39392) | 28367 (21697 to 36739) | 437.99 (339.50 to 568.59) | 540.30 (422.38 to 718.56) | 365.68 (279.69 to 473.61) |
|  |  | 2021 year | 54963 (43038 to 70903) | 28882 (22185 to 37647) | 26081 (20105 to 34010) | 404.46 (316.71 to 521.76) | 505.65 (388.39 to 659.10) | 331.08 (255.22 to 431.75) |
|  |  | Rate of change（%） | -25.89 (-33.85 to -17.03) | -15.29 (-26.66 to -2.15) | -34.92 (-43.15 to -27.16) | NA | NA | NA |
|  | 75+ years |  |  |  |  |  |  |  |
|  |  | 1990 year | 55211 (39743 to 72249) | 14700 (10585 to 19612) | 40511 (29463 to 52360) | 2127.78 (1531.67 to 2784.44) | 2172.46 (1564.40 to 2898.38) | 2112.01 (1536.02 to 2729.78) |
|  |  | 2000 year | 45133 (33618 to 58159) | 11807 (8331 to 16170) | 33326 (25212 to 42196) | 1900.83 (1415.88 to 2449.45) | 1972.70 (1391.89 to 2701.59) | 1876.61 (1419.69 to 2376.10) |
|  |  | 2010 year | 43431 (32657 to 54694) | 12535 (8875 to 16408) | 30895 (23672 to 39626) | 1550.07 (1165.57 to 1952.07) | 1520.73 (1076.66 to 1990.57) | 1562.31 (1197.04 to 2003.80) |
|  |  | 2021 year | 41958 (32648 to 53180) | 11617 (8911 to 14830) | 30341 (23773 to 38616) | 1438.32 (1119.16 to 1823.01) | 1356.57 (1040.65 to 1731.80) | 1472.28 (1153.58 to 1873.83) |
|  |  | Rate of change（%） | -24.00 (-32.06 to -14.14) | -20.97 (-31.63 to -6.54) | -25.10 (-34.91 to -13.76) | NA | NA | NA |

95% UI: 95% uncertainty interval.
