# Supplementary material for: The burden of ischemic stroke in Eastern Europe from 1990 to 2021
Source: BMC Neurol. 2025 Feb 22;25:74. doi: 10.1186/s12883-025-04081-z (PMC11846382; doi:10.1186/s12883-025-04081-z)
Supplement: Supplementary file 2 — Supplementary Material 2 [file 12883_2025_4081_MOESM2_ESM.zip › Supplementary Table 1-10/Supplementary Table 5.docx]

Supplementary Table 5. Trends in ischemic stroke mortality by year, age group, and gender in Eastern European countries (1990-2021): Death counts and Death rates (per 100,000 population) across demographic groups.

| **Location** | **Age** | **Year** | **Death cases**  **(95% UI)** | | | **Death rate (1/100000)**  **(95% UI)** | | |
| --- | --- | --- | --- | --- | --- | --- | --- | --- |
|  |  |  | **Both** | **Male** | **Female** | **Both** | **Male** | **Female** |
| Belarus |  |  |  |  |  |  |  |  |
|  | 0-14 years |  |  |  |  |  |  |  |
|  |  | 1990 year | 1 (1 to 2) | 1 (0 to 1) | 1 (0 to 1) | 0.05 (0.04 to 0.07) | 0.05 (0.03 to 0.07) | 0.05 (0.03 to 0.08) |
|  |  | 2000 year | 1 (0 to 1) | 0 (0 to 0) | 0 (0 to 0) | 0.03 (0.03 to 0.04) | 0.04 (0.02 to 0.05) | 0.03 (0.02 to 0.05) |
|  |  | 2010 year | 0 (0 to 0) | 0 (0 to 0) | 0 (0 to 0) | 0.01 (0.01 to 0.02) | 0.02 (0.01 to 0.02) | 0.01 (0.01 to 0.02) |
|  |  | 2021 year | 0 (0 to 0) | 0 (0 to 0) | 0 (0 to 0) | 0.00 (0.00 to 0.00) | 0.00 (0.00 to 0.00) | 0.00 (0.00 to 0.00) |
|  |  | Rate of change（%） | -94.89 (-96.70 to -92.67) | -95.17 (-97.05 to -92.06) | -94.61 (-96.75 to -91.59) | NA | NA | NA |
|  | 15-49 years |  |  |  |  |  |  |  |
|  |  | 1990 year | 146 (112 to 167) | 97 (65 to 115) | 49 (37 to 58) | 2.89 (2.23 to 3.32) | 3.87 (2.59 to 4.58) | 1.91 (1.44 to 2.29) |
|  |  | 2000 year | 216 (173 to 247) | 154 (105 to 181) | 62 (46 to 75) | 4.02 (3.22 to 4.61) | 5.78 (3.94 to 6.78) | 2.29 (1.69 to 2.80) |
|  |  | 2010 year | 173 (147 to 195) | 130 (106 to 151) | 43 (35 to 52) | 3.47 (2.95 to 3.93) | 5.24 (4.29 to 6.10) | 1.71 (1.39 to 2.06) |
|  |  | 2021 year | 89 (70 to 110) | 66 (52 to 83) | 23 (17 to 29) | 2.09 (1.65 to 2.59) | 3.10 (2.45 to 3.89) | 1.07 (0.81 to 1.35) |
|  |  | Rate of change（%） | -38.93 (-53.70 to -12.91) | -31.80 (-49.78 to 11.46) | -53.19 (-66.23 to -33.15) | NA | NA | NA |
|  | 50-74 years |  |  |  |  |  |  |  |
|  |  | 1990 year | 4693 (4291 to 5036) | 2149 (1798 to 2350) | 2544 (2298 to 2751) | 186.05 (170.14 to 199.67) | 207.40 (173.51 to 226.80) | 171.17 (154.60 to 185.06) |
|  |  | 2000 year | 7292 (6681 to 7808) | 3680 (3206 to 4012) | 3612 (3267 to 3909) | 294.68 (269.98 to 315.55) | 356.54 (310.62 to 388.72) | 250.41 (226.48 to 271.04) |
|  |  | 2010 year | 5165 (4818 to 5508) | 2935 (2670 to 3171) | 2229 (2067 to 2416) | 196.44 (183.26 to 209.50) | 263.40 (239.62 to 284.55) | 147.18 (136.44 to 159.47) |
|  |  | 2021 year | 4071 (3279 to 4867) | 2426 (1955 to 2972) | 1645 (1336 to 1945) | 138.65 (111.67 to 165.77) | 193.90 (156.23 to 237.57) | 97.61 (79.27 to 115.46) |
|  |  | Rate of change（%） | -13.26 (-28.79 to 6.25) | 12.91 (-8.91 to 45.49) | -35.36 (-47.59 to -21.75) | NA | NA | NA |
|  | 75+ years |  |  |  |  |  |  |  |
|  |  | 1990 year | 8003 (7291 to 8523) | 2108 (1922 to 2262) | 5895 (5274 to 6339) | 1681.65 (1532.01 to 1790.99) | 1625.61 (1481.95 to 1744.28) | 1702.64 (1523.31 to 1831.01) |
|  |  | 2000 year | 8039 (7330 to 8578) | 1950 (1799 to 2083) | 6088 (5478 to 6563) | 1775.93 (1619.43 to 1895.03) | 1686.08 (1555.23 to 1800.70) | 1806.77 (1625.54 to 1947.55) |
|  |  | 2010 year | 9684 (8856 to 10232) | 2642 (2436 to 2818) | 7042 (6345 to 7520) | 1602.44 (1465.52 to 1693.19) | 1615.48 (1489.85 to 1723.14) | 1597.60 (1439.34 to 1705.97) |
|  |  | 2021 year | 8173 (6971 to 9433) | 2148 (1865 to 2430) | 6025 (5097 to 6994) | 1470.51 (1254.26 to 1697.38) | 1490.58 (1294.50 to 1686.35) | 1463.48 (1238.10 to 1698.77) |
|  |  | Rate of change（%） | 2.12 (-11.81 to 17.34) | 1.88 (-12.41 to 18.01) | 2.21 (-12.45 to 18.86) | NA | NA | NA |
| Estonia |  |  |  |  |  |  |  |  |
|  | 0-14 years |  |  |  |  |  |  |  |
|  |  | 1990 year | 0 (0 to 0) | 0 (0 to 0) | 0 (0 to 0) | 0.03 (0.03 to 0.03) | 0.03 (0.02 to 0.04) | 0.03 (0.03 to 0.03) |
|  |  | 2000 year | 0 (0 to 0) | 0 (0 to 0) | 0 (0 to 0) | 0.01 (0.01 to 0.01) | 0.01 (0.01 to 0.01) | 0.01 (0.01 to 0.01) |
|  |  | 2010 year | 0 (0 to 0) | 0 (0 to 0) | 0 (0 to 0) | 0.00 (0.00 to 0.00) | 0.00 (0.00 to 0.00) | 0.00 (0.00 to 0.01) |
|  |  | 2021 year | 0 (0 to 0) | 0 (0 to 0) | 0 (0 to 0) | 0.00 (0.00 to 0.00) | 0.00 (0.00 to 0.00) | 0.00 (0.00 to 0.00) |
|  |  | Rate of change（%） | -98.59 (-98.95 to -98.15) | -98.76 (-99.14 to -98.20) | -98.41 (-98.84 to -97.93) | NA | NA | NA |
|  | 15-49 years |  |  |  |  |  |  |  |
|  |  | 1990 year | 21 (18 to 25) | 14 (11 to 17) | 7 (6 to 8) | 2.80 (2.35 to 3.29) | 3.75 (3.04 to 4.54) | 1.86 (1.49 to 2.21) |
|  |  | 2000 year | 20 (17 to 22) | 14 (12 to 16) | 6 (5 to 6) | 2.84 (2.51 to 3.26) | 4.07 (3.51 to 4.81) | 1.62 (1.38 to 1.86) |
|  |  | 2010 year | 6 (5 to 7) | 4 (4 to 5) | 2 (1 to 2) | 0.90 (0.79 to 1.03) | 1.29 (1.10 to 1.52) | 0.51 (0.44 to 0.59) |
|  |  | 2021 year | 3 (2 to 4) | 2 (2 to 3) | 1 (1 to 1) | 0.52 (0.43 to 0.62) | 0.81 (0.66 to 0.98) | 0.22 (0.18 to 0.27) |
|  |  | Rate of change（%） | -85.84 (-88.94 to -81.32) | -83.15 (-87.23 to -76.95) | -91.23 (-93.37 to -88.84) | NA | NA | NA |
|  | 50-74 years |  |  |  |  |  |  |  |
|  |  | 1990 year | 848 (798 to 898) | 418 (387 to 448) | 430 (394 to 463) | 223.13 (210.08 to 236.15) | 267.45 (247.63 to 286.46) | 192.19 (176.08 to 206.93) |
|  |  | 2000 year | 803 (759 to 849) | 434 (404 to 463) | 369 (339 to 401) | 212.04 (200.37 to 224.06) | 275.54 (256.39 to 293.70) | 166.82 (153.08 to 181.29) |
|  |  | 2010 year | 280 (259 to 304) | 170 (156 to 185) | 111 (98 to 123) | 72.85 (67.41 to 78.91) | 103.83 (95.64 to 113.58) | 50.02 (44.19 to 55.54) |
|  |  | 2021 year | 176 (152 to 200) | 123 (105 to 141) | 53 (45 to 61) | 44.50 (38.45 to 50.60) | 69.73 (59.78 to 80.17) | 24.30 (20.63 to 27.98) |
|  |  | Rate of change（%） | -79.25 (-82.35 to -76.46) | -70.66 (-74.97 to -66.12) | -87.60 (-89.66 to -85.45) | NA | NA | NA |
|  | 75+ years |  |  |  |  |  |  |  |
|  |  | 1990 year | 1833 (1687 to 1941) | 475 (442 to 502) | 1359 (1247 to 1448) | 2292.64 (2109.45 to 2427.25) | 2254.80 (2101.93 to 2386.13) | 2306.16 (2116.01 to 2457.85) |
|  |  | 2000 year | 1451 (1311 to 1540) | 355 (330 to 375) | 1097 (986 to 1177) | 1921.93 (1736.02 to 2039.74) | 1873.94 (1746.85 to 1983.20) | 1937.98 (1743.21 to 2079.96) |
|  |  | 2010 year | 749 (658 to 810) | 230 (211 to 249) | 519 (446 to 571) | 725.42 (637.07 to 784.54) | 810.94 (742.87 to 877.22) | 693.00 (595.34 to 762.23) |
|  |  | 2021 year | 707 (598 to 792) | 239 (208 to 269) | 468 (389 to 526) | 564.14 (477.49 to 632.48) | 660.36 (573.66 to 743.18) | 525.04 (437.11 to 591.03) |
|  |  | Rate of change（%） | -61.46 (-66.29 to -57.01) | -49.66 (-56.00 to -43.06) | -65.58 (-70.17 to -61.47) | NA | NA | NA |
| Latvia |  |  |  |  |  |  |  |  |
|  | 0-14 years |  |  |  |  |  |  |  |
|  |  | 1990 year | 0 (0 to 0) | 0 (0 to 0) | 0 (0 to 0) | 0.04 (0.03 to 0.04) | 0.04 (0.03 to 0.05) | 0.04 (0.03 to 0.04) |
|  |  | 2000 year | 0 (0 to 0) | 0 (0 to 0) | 0 (0 to 0) | 0.01 (0.01 to 0.02) | 0.01 (0.01 to 0.01) | 0.02 (0.01 to 0.02) |
|  |  | 2010 year | 0 (0 to 0) | 0 (0 to 0) | 0 (0 to 0) | 0.01 (0.01 to 0.01) | 0.01 (0.00 to 0.01) | 0.01 (0.01 to 0.01) |
|  |  | 2021 year | 0 (0 to 0) | 0 (0 to 0) | 0 (0 to 0) | 0.00 (0.00 to 0.00) | 0.00 (0.00 to 0.00) | 0.00 (0.00 to 0.00) |
|  |  | Rate of change（%） | -98.04 (-98.60 to -97.39) | -98.74 (-99.16 to -98.21) | -97.31 (-98.09 to -96.37) | NA | NA | NA |
|  | 15-49 years |  |  |  |  |  |  |  |
|  |  | 1990 year | 42 (36 to 48) | 28 (23 to 33) | 14 (12 to 16) | 3.27 (2.84 to 3.70) | 4.40 (3.64 to 5.23) | 2.15 (1.79 to 2.50) |
|  |  | 2000 year | 33 (30 to 37) | 21 (18 to 25) | 12 (11 to 14) | 2.85 (2.58 to 3.17) | 3.67 (3.18 to 4.29) | 2.05 (1.80 to 2.33) |
|  |  | 2010 year | 26 (23 to 29) | 18 (16 to 21) | 8 (7 to 9) | 2.52 (2.27 to 2.81) | 3.47 (3.01 to 4.04) | 1.58 (1.39 to 1.79) |
|  |  | 2021 year | 12 (10 to 14) | 9 (7 to 11) | 3 (2 to 3) | 1.53 (1.27 to 1.80) | 2.29 (1.85 to 2.77) | 0.74 (0.59 to 0.89) |
|  |  | Rate of change（%） | -71.37 (-77.20 to -64.40) | -67.46 (-75.37 to -57.24) | -79.24 (-84.24 to -73.32) | NA | NA | NA |
|  | 50-74 years |  |  |  |  |  |  |  |
|  |  | 1990 year | 1425 (1342 to 1517) | 682 (623 to 735) | 743 (682 to 811) | 214.58 (202.10 to 228.51) | 253.16 (231.21 to 272.97) | 188.23 (172.76 to 205.41) |
|  |  | 2000 year | 1520 (1434 to 1598) | 799 (740 to 856) | 721 (671 to 772) | 232.33 (219.18 to 244.21) | 298.01 (276.27 to 319.41) | 186.77 (173.82 to 199.99) |
|  |  | 2010 year | 1126 (1060 to 1194) | 649 (603 to 694) | 478 (440 to 519) | 181.46 (170.83 to 192.30) | 251.97 (234.43 to 269.46) | 131.50 (121.16 to 142.77) |
|  |  | 2021 year | 794 (703 to 889) | 464 (400 to 532) | 331 (286 to 373) | 133.28 (117.99 to 149.18) | 180.18 (155.42 to 206.72) | 97.64 (84.49 to 110.07) |
|  |  | Rate of change（%） | -44.25 (-51.64 to -36.73) | -32.01 (-42.48 to -20.04) | -55.48 (-62.09 to -47.54) | NA | NA | NA |
|  | 75+ years |  |  |  |  |  |  |  |
|  |  | 1990 year | 3715 (3431 to 3931) | 1035 (967 to 1093) | 2680 (2461 to 2860) | 2658.49 (2455.10 to 2812.98) | 2652.01 (2478.62 to 2799.50) | 2661.00 (2444.07 to 2839.65) |
|  |  | 2000 year | 3195 (2911 to 3384) | 794 (738 to 843) | 2401 (2169 to 2557) | 2487.50 (2266.22 to 2634.04) | 2518.44 (2341.61 to 2673.47) | 2477.44 (2238.18 to 2637.90) |
|  |  | 2010 year | 2888 (2590 to 3127) | 747 (688 to 799) | 2141 (1895 to 2319) | 1746.98 (1566.93 to 1891.77) | 1702.30 (1568.21 to 1821.42) | 1763.13 (1560.89 to 1910.18) |
|  |  | 2021 year | 3497 (3018 to 3874) | 912 (810 to 1012) | 2585 (2189 to 2867) | 1851.17 (1597.52 to 2050.33) | 1742.21 (1547.46 to 1932.31) | 1892.94 (1602.83 to 2099.32) |
|  |  | Rate of change（%） | -5.85 (-15.59 to 4.14) | -11.88 (-21.00 to -1.16) | -3.53 (-13.96 to 7.11) | NA | NA | NA |
| Lithuania |  |  |  |  |  |  |  |  |
|  | 0-14 years |  |  |  |  |  |  |  |
|  |  | 1990 year | 0 (0 to 0) | 0 (0 to 0) | 0 (0 to 0) | 0.02 (0.02 to 0.03) | 0.02 (0.02 to 0.02) | 0.03 (0.02 to 0.03) |
|  |  | 2000 year | 0 (0 to 0) | 0 (0 to 0) | 0 (0 to 0) | 0.01 (0.01 to 0.01) | 0.01 (0.01 to 0.01) | 0.01 (0.01 to 0.01) |
|  |  | 2010 year | 0 (0 to 0) | 0 (0 to 0) | 0 (0 to 0) | 0.00 (0.00 to 0.01) | 0.00 (0.00 to 0.00) | 0.01 (0.00 to 0.01) |
|  |  | 2021 year | 0 (0 to 0) | 0 (0 to 0) | 0 (0 to 0) | 0.00 (0.00 to 0.00) | 0.00 (0.00 to 0.00) | 0.00 (0.00 to 0.00) |
|  |  | Rate of change（%） | -97.51 (-98.13 to -96.79) | -97.31 (-98.05 to -96.28) | -97.66 (-98.23 to -96.98) | NA | NA | NA |
|  | 15-49 years |  |  |  |  |  |  |  |
|  |  | 1990 year | 48 (41 to 57) | 31 (26 to 37) | 18 (15 to 21) | 2.65 (2.23 to 3.11) | 3.38 (2.84 to 4.02) | 1.92 (1.59 to 2.29) |
|  |  | 2000 year | 37 (32 to 42) | 25 (21 to 28) | 12 (10 to 14) | 2.08 (1.81 to 2.39) | 2.84 (2.43 to 3.27) | 1.33 (1.16 to 1.54) |
|  |  | 2010 year | 31 (27 to 35) | 22 (19 to 26) | 8 (7 to 10) | 2.03 (1.77 to 2.30) | 2.96 (2.53 to 3.42) | 1.10 (0.94 to 1.30) |
|  |  | 2021 year | 17 (14 to 20) | 14 (11 to 16) | 3 (3 to 4) | 1.47 (1.20 to 1.76) | 2.35 (1.93 to 2.83) | 0.57 (0.45 to 0.71) |
|  |  | Rate of change（%） | -64.93 (-73.63 to -55.22) | -55.41 (-66.61 to -42.79) | -81.43 (-86.27 to -75.47) | NA | NA | NA |
|  | 50-74 years |  |  |  |  |  |  |  |
|  |  | 1990 year | 1142 (1075 to 1213) | 571 (534 to 609) | 571 (527 to 615) | 136.22 (128.25 to 144.64) | 163.78 (152.99 to 174.65) | 116.58 (107.55 to 125.54) |
|  |  | 2000 year | 1320 (1237 to 1396) | 671 (626 to 714) | 648 (600 to 694) | 150.76 (141.34 to 159.53) | 184.19 (171.84 to 195.89) | 126.91 (117.53 to 135.88) |
|  |  | 2010 year | 1148 (1074 to 1224) | 666 (621 to 711) | 483 (446 to 524) | 132.62 (123.99 to 141.35) | 182.23 (169.85 to 194.58) | 96.41 (89.12 to 104.59) |
|  |  | 2021 year | 750 (651 to 835) | 487 (426 to 543) | 263 (222 to 300) | 84.16 (73.07 to 93.70) | 126.16 (110.28 to 140.73) | 52.07 (44.03 to 59.30) |
|  |  | Rate of change（%） | -34.33 (-44.08 to -26.25) | -14.76 (-27.31 to -2.26) | -53.91 (-61.00 to -46.51) | NA | NA | NA |
|  | 75+ years |  |  |  |  |  |  |  |
|  |  | 1990 year | 2220 (2039 to 2369) | 702 (650 to 750) | 1518 (1385 to 1627) | 1273.36 (1169.76 to 1358.80) | 1239.83 (1147.63 to 1324.36) | 1289.50 (1176.55 to 1382.40) |
|  |  | 2000 year | 2515 (2274 to 2674) | 716 (662 to 758) | 1800 (1617 to 1928) | 1452.47 (1313.04 to 1544.01) | 1430.27 (1322.85 to 1515.83) | 1461.49 (1313.00 to 1565.97) |
|  |  | 2010 year | 3156 (2844 to 3362) | 913 (843 to 977) | 2242 (1990 to 2407) | 1292.11 (1164.56 to 1376.52) | 1278.51 (1179.78 to 1368.24) | 1297.73 (1151.39 to 1393.09) |
|  |  | 2021 year | 3016 (2615 to 3342) | 879 (779 to 973) | 2137 (1830 to 2379) | 1101.05 (954.92 to 1220.26) | 1105.45 (978.95 to 1223.43) | 1099.25 (941.73 to 1223.94) |
|  |  | Rate of change（%） | 35.84 (21.15 to 49.74) | 25.18 (11.69 to 38.85) | 40.77 (25.13 to 56.06) | NA | NA | NA |
| Republic of Moldova | | |  |  |  |  |  |  |
|  | 0-14 years |  |  |  |  |  |  |  |
|  |  | 1990 year | 1 (0 to 1) | 0 (0 to 0) | 0 (0 to 0) | 0.05 (0.04 to 0.06) | 0.04 (0.03 to 0.06) | 0.05 (0.04 to 0.06) |
|  |  | 2000 year | 0 (0 to 0) | 0 (0 to 0) | 0 (0 to 0) | 0.03 (0.02 to 0.03) | 0.02 (0.02 to 0.03) | 0.03 (0.03 to 0.03) |
|  |  | 2010 year | 0 (0 to 0) | 0 (0 to 0) | 0 (0 to 0) | 0.02 (0.01 to 0.02) | 0.01 (0.01 to 0.02) | 0.02 (0.01 to 0.02) |
|  |  | 2021 year | 0 (0 to 0) | 0 (0 to 0) | 0 (0 to 0) | 0.01 (0.00 to 0.01) | 0.00 (0.00 to 0.01) | 0.01 (0.00 to 0.01) |
|  |  | Rate of change（%） | -95.41 (-96.69 to -93.63) | -95.55 (-96.98 to -93.32) | -95.27 (-96.75 to -93.23) | NA | NA | NA |
|  | 15-49 years |  |  |  |  |  |  |  |
|  |  | 1990 year | 31 (25 to 37) | 17 (13 to 22) | 13 (10 to 16) | 1.39 (1.12 to 1.67) | 1.63 (1.24 to 2.03) | 1.17 (0.89 to 1.45) |
|  |  | 2000 year | 40 (35 to 44) | 25 (22 to 29) | 15 (13 to 16) | 1.75 (1.56 to 1.96) | 2.25 (1.93 to 2.62) | 1.27 (1.13 to 1.41) |
|  |  | 2010 year | 47 (42 to 53) | 32 (28 to 38) | 15 (13 to 17) | 2.26 (2.00 to 2.57) | 3.09 (2.65 to 3.61) | 1.43 (1.26 to 1.61) |
|  |  | 2021 year | 22 (18 to 26) | 16 (13 to 19) | 6 (5 to 7) | 1.23 (1.02 to 1.45) | 1.74 (1.42 to 2.12) | 0.70 (0.58 to 0.83) |
|  |  | Rate of change（%） | -28.06 (-43.82 to -4.15) | -9.41 (-33.22 to 27.14) | -52.83 (-63.94 to -36.72) | NA | NA | NA |
|  | 50-74 years |  |  |  |  |  |  |  |
|  |  | 1990 year | 897 (783 to 1006) | 421 (343 to 482) | 477 (398 to 547) | 101.24 (88.36 to 113.55) | 111.25 (90.68 to 127.61) | 93.80 (78.34 to 107.58) |
|  |  | 2000 year | 1244 (1167 to 1324) | 620 (567 to 679) | 624 (583 to 667) | 143.37 (134.44 to 152.60) | 165.87 (151.66 to 181.55) | 126.33 (117.98 to 134.98) |
|  |  | 2010 year | 1603 (1507 to 1696) | 858 (795 to 927) | 744 (694 to 794) | 161.72 (152.08 to 171.10) | 198.18 (183.49 to 214.08) | 133.42 (124.35 to 142.23) |
|  |  | 2021 year | 1354 (1211 to 1514) | 768 (678 to 868) | 586 (526 to 656) | 126.31 (112.94 to 141.23) | 165.28 (145.94 to 186.75) | 96.50 (86.65 to 107.95) |
|  |  | Rate of change（%） | 50.96 (30.10 to 81.12) | 82.66 (50.75 to 129.09) | 22.99 (3.89 to 49.82) | NA | NA | NA |
|  | 75+ years |  |  |  |  |  |  |  |
|  |  | 1990 year | 1821 (1653 to 1964) | 612 (542 to 667) | 1209 (1071 to 1331) | 1419.14 (1288.74 to 1530.73) | 1382.30 (1224.37 to 1505.86) | 1438.55 (1274.59 to 1583.51) |
|  |  | 2000 year | 1499 (1383 to 1574) | 546 (514 to 577) | 953 (865 to 1012) | 1047.07 (966.11 to 1099.32) | 1172.51 (1103.73 to 1238.77) | 986.54 (895.30 to 1047.66) |
|  |  | 2010 year | 2101 (1932 to 2224) | 790 (727 to 844) | 1311 (1180 to 1398) | 1124.79 (1034.06 to 1190.38) | 1245.49 (1147.06 to 1330.85) | 1062.74 (956.58 to 1132.71) |
|  |  | 2021 year | 1756 (1556 to 1953) | 616 (547 to 683) | 1141 (983 to 1281) | 839.93 (744.18 to 933.87) | 885.35 (787.01 to 982.06) | 817.31 (704.03 to 917.40) |
|  |  | Rate of change（%） | -3.53 (-14.20 to 9.98) | 0.61 (-12.17 to 17.97) | -5.63 (-18.02 to 7.87) | NA | NA | NA |
| Russian Federation | | |  |  |  |  |  |  |
|  | 0-14 years |  |  |  |  |  |  |  |
|  |  | 1990 year | 13 (13 to 14) | 7 (6 to 7) | 7 (7 to 7) | 0.04 (0.04 to 0.04) | 0.04 (0.04 to 0.04) | 0.04 (0.04 to 0.04) |
|  |  | 2000 year | 8 (8 to 8) | 4 (4 to 4) | 4 (4 to 4) | 0.03 (0.03 to 0.03) | 0.03 (0.03 to 0.03) | 0.03 (0.03 to 0.03) |
|  |  | 2010 year | 4 (4 to 4) | 2 (2 to 2) | 2 (2 to 2) | 0.02 (0.02 to 0.02) | 0.02 (0.02 to 0.02) | 0.02 (0.02 to 0.02) |
|  |  | 2021 year | 2 (2 to 2) | 1 (1 to 1) | 1 (1 to 1) | 0.01 (0.01 to 0.01) | 0.01 (0.01 to 0.01) | 0.01 (0.01 to 0.01) |
|  |  | Rate of change（%） | -84.75 (-85.86 to -83.78) | -84.93 (-86.55 to -83.54) | -84.58 (-85.67 to -83.43) | NA | NA | NA |
|  | 15-49 years |  |  |  |  |  |  |  |
|  |  | 1990 year | 2824 (2748 to 2886) | 1837 (1789 to 1885) | 987 (952 to 1016) | 3.80 (3.70 to 3.89) | 4.94 (4.81 to 5.07) | 2.67 (2.57 to 2.74) |
|  |  | 2000 year | 4893 (4794 to 4989) | 3397 (3320 to 3477) | 1496 (1460 to 1531) | 6.06 (5.94 to 6.18) | 8.49 (8.29 to 8.69) | 3.68 (3.59 to 3.77) |
|  |  | 2010 year | 3537 (3474 to 3606) | 2477 (2420 to 2537) | 1060 (1037 to 1084) | 4.70 (4.61 to 4.79) | 6.65 (6.49 to 6.81) | 2.79 (2.73 to 2.85) |
|  |  | 2021 year | 2327 (2132 to 2531) | 1651 (1475 to 1818) | 676 (585 to 763) | 3.45 (3.16 to 3.75) | 4.92 (4.40 to 5.42) | 1.99 (1.73 to 2.25) |
|  |  | Rate of change（%） | -17.58 (-24.55 to -10.15) | -10.11 (-20.40 to -0.21) | -31.48 (-40.22 to -22.67) | NA | NA | NA |
|  | 50-74 years |  |  |  |  |  |  |  |
|  |  | 1990 year | 91734 (89809 to 93128) | 41797 (41098 to 42380) | 49937 (48568 to 50869) | 255.89 (250.52 to 259.77) | 288.22 (283.40 to 292.24) | 233.92 (227.51 to 238.29) |
|  |  | 2000 year | 154619 (151612 to 156622) | 78739 (77677 to 79736) | 75880 (73791 to 77119) | 431.45 (423.05 to 437.04) | 533.02 (525.83 to 539.77) | 360.22 (350.30 to 366.10) |
|  |  | 2010 year | 100051 (98034 to 101455) | 54643 (53758 to 55453) | 45408 (44029 to 46251) | 251.39 (246.33 to 254.92) | 330.07 (324.72 to 334.96) | 195.36 (189.42 to 198.98) |
|  |  | 2021 year | 71038 (65519 to 77074) | 40649 (35689 to 45021) | 30389 (27034 to 33564) | 163.52 (150.82 to 177.42) | 221.16 (194.18 to 244.95) | 121.25 (107.87 to 133.92) |
|  |  | Rate of change（%） | -22.56 (-28.77 to -16.00) | -2.75 (-13.97 to 7.81) | -39.15 (-45.64 to -32.87) | NA | NA | NA |
|  | 75+ years |  |  |  |  |  |  |  |
|  |  | 1990 year | 185468 (172761 to 191001) | 42628 (40837 to 43556) | 142840 (132073 to 147643) | 2983.65 (2779.23 to 3072.66) | 3089.35 (2959.56 to 3156.65) | 2953.49 (2730.87 to 3052.80) |
|  |  | 2000 year | 216249 (201948 to 222716) | 47265 (45274 to 48184) | 168985 (156694 to 174586) | 3632.51 (3392.28 to 3741.14) | 3597.62 (3446.08 to 3667.63) | 3642.39 (3377.48 to 3763.13) |
|  |  | 2010 year | 193615 (179411 to 200187) | 51037 (48644 to 52312) | 142578 (130675 to 147898) | 2477.87 (2296.08 to 2561.98) | 2438.87 (2324.50 to 2499.80) | 2492.13 (2284.08 to 2585.12) |
|  |  | 2021 year | 167078 (148007 to 179917) | 41802 (37588 to 45575) | 125276 (109481 to 135741) | 2115.76 (1874.25 to 2278.34) | 1947.43 (1751.13 to 2123.20) | 2178.59 (1903.90 to 2360.59) |
|  |  | Rate of change（%） | -9.92 (-15.84 to -4.52) | -1.94 (-10.53 to 5.62) | -12.30 (-19.30 to -6.12) | NA | NA | NA |
| Ukraine |  |  |  |  |  |  |  |  |
|  | 0-14 years |  |  |  |  |  |  |  |
|  |  | 1990 year | 1 (1 to 2) | 1 (1 to 2) | 0 (0 to 0) | 0.01 (0.01 to 0.02) | 0.02 (0.01 to 0.03) | 0.00 (0.00 to 0.01) |
|  |  | 2000 year | 1 (1 to 1) | 1 (0 to 1) | 0 (0 to 0) | 0.01 (0.01 to 0.01) | 0.01 (0.01 to 0.02) | 0.00 (0.00 to 0.01) |
|  |  | 2010 year | 0 (0 to 0) | 0 (0 to 0) | 0 (0 to 0) | 0.01 (0.00 to 0.01) | 0.01 (0.01 to 0.01) | 0.00 (0.00 to 0.00) |
|  |  | 2021 year | 0 (0 to 0) | 0 (0 to 0) | 0 (0 to 0) | 0.00 (0.00 to 0.00) | 0.00 (0.00 to 0.01) | 0.00 (0.00 to 0.00) |
|  |  | Rate of change（%） | -85.96 (-91.51 to -78.87) | -86.84 (-91.74 to -79.35) | -82.04 (-90.61 to -71.91) | NA | NA | NA |
|  | 15-49 years |  |  |  |  |  |  |  |
|  |  | 1990 year | 711 (589 to 835) | 468 (362 to 582) | 243 (195 to 293) | 2.85 (2.36 to 3.35) | 3.81 (2.94 to 4.73) | 1.92 (1.54 to 2.31) |
|  |  | 2000 year | 1170 (1007 to 1311) | 846 (697 to 978) | 324 (264 to 380) | 4.60 (3.96 to 5.15) | 6.75 (5.56 to 7.81) | 2.51 (2.04 to 2.95) |
|  |  | 2010 year | 804 (731 to 887) | 580 (513 to 650) | 223 (191 to 260) | 3.43 (3.12 to 3.78) | 5.00 (4.41 to 5.59) | 1.89 (1.62 to 2.20) |
|  |  | 2021 year | 634 (440 to 854) | 481 (312 to 688) | 152 (82 to 240) | 3.13 (2.17 to 4.22) | 4.76 (3.08 to 6.79) | 1.51 (0.81 to 2.37) |
|  |  | Rate of change（%） | -10.91 (-41.00 to 30.20) | 2.87 (-36.61 to 60.40) | -37.41 (-66.21 to 3.20) | NA | NA | NA |
|  | 50-74 years |  |  |  |  |  |  |  |
|  |  | 1990 year | 29375 (27419 to 31242) | 13322 (12161 to 14458) | 16053 (14952 to 17200) | 212.99 (198.81 to 226.53) | 236.10 (215.53 to 256.23) | 196.99 (183.48 to 211.07) |
|  |  | 2000 year | 37757 (35657 to 39946) | 19521 (18241 to 20845) | 18236 (16857 to 19594) | 286.87 (270.91 to 303.50) | 356.34 (332.97 to 380.51) | 237.34 (219.39 to 255.02) |
|  |  | 2010 year | 25848 (24485 to 27228) | 13517 (12667 to 14432) | 12331 (11380 to 13219) | 195.24 (184.94 to 205.66) | 246.57 (231.05 to 263.26) | 158.96 (146.70 to 170.41) |
|  |  | 2021 year | 19479 (14659 to 25096) | 10780 (7168 to 15215) | 8699 (5805 to 12374) | 143.34 (107.87 to 184.67) | 188.73 (125.49 to 266.37) | 110.43 (73.70 to 157.09) |
|  |  | Rate of change（%） | -33.69 (-50.63 to -14.31) | -19.08 (-46.81 to 14.86) | -45.81 (-63.51 to -21.41) | NA | NA | NA |
|  | 75+ years |  |  |  |  |  |  |  |
|  |  | 1990 year | 68249 (63755 to 71026) | 18039 (16859 to 19088) | 50210 (46395 to 52837) | 2630.27 (2457.06 to 2737.31) | 2666.01 (2491.58 to 2820.95) | 2617.66 (2418.79 to 2754.61) |
|  |  | 2000 year | 59047 (55285 to 61766) | 14590 (13723 to 15492) | 44458 (41235 to 46700) | 2486.84 (2328.39 to 2601.36) | 2437.61 (2292.76 to 2588.41) | 2503.44 (2321.96 to 2629.72) |
|  |  | 2010 year | 52335 (48777 to 54771) | 15602 (14722 to 16478) | 36733 (33779 to 38915) | 1867.87 (1740.89 to 1954.82) | 1892.73 (1785.99 to 1999.09) | 1857.50 (1708.12 to 1967.85) |
|  |  | 2021 year | 44297 (34669 to 55074) | 13379 (10309 to 17076) | 30918 (22318 to 40838) | 1518.48 (1188.47 to 1887.95) | 1562.38 (1203.86 to 1994.09) | 1500.24 (1082.97 to 1981.63) |
|  |  | Rate of change（%） | -35.10 (-47.67 to -20.02) | -25.83 (-43.90 to -4.85) | -38.42 (-53.63 to -18.64) | NA | NA | NA |

95% UI: 95% uncertainty interval.
