# Supplementary material for: The burden of ischemic stroke in Eastern Europe from 1990 to 2021
Source: BMC Neurol. 2025 Feb 22;25:74. doi: 10.1186/s12883-025-04081-z (PMC11846382; doi:10.1186/s12883-025-04081-z)
Supplement: Supplementary file 2 — Supplementary Material 2 [file 12883_2025_4081_MOESM2_ESM.zip › Supplementary Table 1-10/Supplementary Table 6.docx]

Supplementary Table 6. Trends in ischemic stroke disability-adjusted life years by year, age group, and gender in Eastern European countries (1990-2021): DALYs counts and DALYs rates (per 100,000 population) for different demographic groups.

| **Location** | **Age** | **Year** | **DALYs cases**  **(95% UI)** | | | **DALYs rate (1/100000)**  **(95% UI)** | | |
| --- | --- | --- | --- | --- | --- | --- | --- | --- |
|  |  |  | **Both** | **Male** | **Female** | **Both** | **Male** | **Female** |
| Belarus |  |  |  |  |  |  |  |  |
|  | 0-14 years |  |  |  |  |  |  |  |
|  |  | 1990 year | 288 (208 to 387) | 110 (86 to 140) | 177 (118 to 257) | 11.97 (8.65 to 16.09) | 9.00 (7.01 to 11.38) | 15.06 (10.02 to 21.84) |
|  |  | 2000 year | 227 (159 to 304) | 83 (66 to 101) | 144 (91 to 210) | 11.74 (8.22 to 15.77) | 8.36 (6.62 to 10.25) | 15.30 (9.69 to 22.32) |
|  |  | 2010 year | 124 (88 to 170) | 44 (34 to 56) | 80 (50 to 118) | 8.57 (6.07 to 11.78) | 5.92 (4.52 to 7.52) | 11.38 (7.18 to 16.86) |
|  |  | 2021 year | 127 (84 to 176) | 42 (30 to 56) | 85 (53 to 128) | 8.06 (5.30 to 11.17) | 5.21 (3.64 to 6.95) | 11.09 (6.93 to 16.65) |
|  |  | rate of change（%） | -55.77 (-67.34 to -41.06) | -61.70 (-70.02 to -53.84) | -52.08 (-68.54 to -28.10) | NA | NA | NA |
|  | 15-49 years |  |  |  |  |  |  |  |
|  |  | 1990 year | 10238 (8330 to 11807) | 5883 (4363 to 6886) | 4355 (3614 to 5105) | 203.04 (165.21 to 234.16) | 234.51 (173.92 to 274.48) | 171.88 (142.62 to 201.50) |
|  |  | 2000 year | 13911 (11780 to 15669) | 8688 (6516 to 10011) | 5223 (4214 to 6162) | 259.63 (219.86 to 292.42) | 326.20 (244.63 to 375.87) | 193.82 (156.37 to 228.66) |
|  |  | 2010 year | 11477 (9934 to 12976) | 7377 (6169 to 8498) | 4101 (3346 to 4858) | 230.57 (199.57 to 260.69) | 297.84 (249.07 to 343.10) | 163.96 (133.78 to 194.23) |
|  |  | 2021 year | 7207 (5889 to 8651) | 4290 (3489 to 5174) | 2917 (2302 to 3592) | 169.39 (138.41 to 203.32) | 201.03 (163.48 to 242.46) | 137.55 (108.54 to 169.40) |
|  |  | rate of change（%） | -29.60 (-41.12 to -11.69) | -27.08 (-42.64 to 3.58) | -33.02 (-43.93 to -17.96) | NA | NA | NA |
|  | 50-74 years |  |  |  |  |  |  |  |
|  |  | 1990 year | 138421 (126356 to 148726) | 65531 (54881 to 71886) | 72889 (65534 to 78859) | 5487.75 (5009.46 to 5896.32) | 6325.55 (5297.46 to 6938.94) | 4903.82 (4408.96 to 5305.46) |
|  |  | 2000 year | 197748 (181725 to 211771) | 102410 (90023 to 111524) | 95338 (85856 to 102887) | 7991.24 (7343.73 to 8557.93) | 9921.46 (8721.40 to 10804.46) | 6609.90 (5952.49 to 7133.29) |
|  |  | 2010 year | 144966 (134670 to 155711) | 83489 (76429 to 90494) | 61477 (56482 to 66652) | 5513.71 (5122.09 to 5922.37) | 7491.82 (6858.29 to 8120.39) | 4058.45 (3728.70 to 4400.05) |
|  |  | 2021 year | 119151 (99257 to 140380) | 70398 (57555 to 85308) | 48753 (41464 to 56291) | 4058.26 (3380.67 to 4781.31) | 5626.52 (4600.08 to 6818.28) | 2893.66 (2461.04 to 3341.01) |
|  |  | rate of change（%） | -13.92 (-27.24 to 2.67) | 7.43 (-11.67 to 35.92) | -33.11 (-43.31 to -20.73) | NA | NA | NA |
|  | 75+ years |  |  |  |  |  |  |  |
|  |  | 1990 year | 104741 (96125 to 111338) | 28869 (26372 to 31296) | 75873 (69044 to 81552) | 22009.61 (20199.13 to 23395.77) | 22263.44 (20337.82 to 24135.13) | 21914.54 (19942.08 to 23554.87) |
|  |  | 2000 year | 107553 (98918 to 114698) | 28033 (25854 to 30097) | 79520 (72349 to 85774) | 23761.08 (21853.40 to 25339.73) | 24236.32 (22352.54 to 26020.78) | 23597.96 (21470.05 to 25453.80) |
|  |  | 2010 year | 129279 (120050 to 136622) | 38261 (35496 to 40702) | 91018 (83296 to 97202) | 21392.65 (19865.43 to 22607.68) | 23398.57 (21708.06 to 24891.52) | 20648.53 (18896.65 to 22051.38) |
|  |  | 2021 year | 99921 (86448 to 115311) | 28298 (24816 to 31992) | 71623 (61420 to 83076) | 17979.08 (15554.79 to 20748.30) | 19641.31 (17224.32 to 22205.03) | 17397.37 (14919.08 to 20179.35) |
|  |  | rate of change（%） | -4.60 (-16.96 to 9.33) | -1.98 (-14.90 to 12.85) | -5.60 (-18.39 to 9.19) | NA | NA | NA |
| Estonia |  |  |  |  |  |  |  |  |
|  | 0-14 years |  |  |  |  |  |  |  |
|  |  | 1990 year | 29 (22 to 37) | 12 (9 to 15) | 17 (13 to 23) | 8.34 (6.41 to 10.66) | 6.70 (5.25 to 8.26) | 10.04 (7.42 to 13.41) |
|  |  | 2000 year | 18 (13 to 24) | 7 (5 to 9) | 11 (8 to 16) | 7.13 (5.07 to 9.58) | 5.14 (3.66 to 6.73) | 9.26 (6.23 to 12.90) |
|  |  | 2010 year | 11 (8 to 15) | 4 (3 to 5) | 7 (5 to 10) | 5.56 (3.96 to 7.42) | 3.71 (2.62 to 4.91) | 7.51 (5.17 to 10.34) |
|  |  | 2021 year | 13 (9 to 18) | 4 (3 to 6) | 9 (6 to 12) | 6.00 (4.09 to 8.27) | 4.02 (2.78 to 5.47) | 8.09 (5.33 to 11.44) |
|  |  | rate of change（%） | -55.46 (-63.55 to -45.40) | -62.61 (-68.96 to -56.26) | -50.50 (-63.04 to -34.81) | NA | NA | NA |
|  | 15-49 years |  |  |  |  |  |  |  |
|  |  | 1990 year | 1454 (1236 to 1688) | 877 (734 to 1032) | 578 (477 to 678) | 191.53 (162.81 to 222.24) | 231.76 (193.96 to 272.84) | 151.59 (125.26 to 178.05) |
|  |  | 2000 year | 1313 (1140 to 1482) | 827 (711 to 950) | 485 (407 to 569) | 190.60 (165.52 to 215.16) | 241.84 (207.69 to 277.67) | 140.01 (117.60 to 164.34) |
|  |  | 2010 year | 617 (510 to 724) | 338 (284 to 394) | 279 (214 to 346) | 96.04 (79.36 to 112.60) | 104.12 (87.32 to 121.19) | 87.79 (67.43 to 108.88) |
|  |  | 2021 year | 451 (356 to 550) | 234 (193 to 282) | 217 (157 to 278) | 78.51 (62.07 to 95.89) | 79.06 (65.36 to 95.49) | 77.92 (56.31 to 99.89) |
|  |  | rate of change（%） | -69.01 (-74.19 to -62.23) | -73.34 (-78.39 to -66.51) | -62.45 (-70.56 to -53.03) | NA | NA | NA |
|  | 50-74 years |  |  |  |  |  |  |  |
|  |  | 1990 year | 23245 (21915 to 24558) | 11806 (10965 to 12616) | 11439 (10580 to 12293) | 6115.89 (5765.80 to 6461.17) | 7554.94 (7016.45 to 8073.29) | 5111.08 (4727.11 to 5492.84) |
|  |  | 2000 year | 21464 (20236 to 22749) | 11808 (11006 to 12614) | 9656 (8944 to 10431) | 5666.22 (5341.95 to 6005.39) | 7494.55 (6985.61 to 8006.14) | 4364.26 (4042.34 to 4714.41) |
|  |  | 2010 year | 8370 (7709 to 9078) | 4890 (4532 to 5322) | 3479 (3098 to 3845) | 2174.86 (2003.22 to 2358.89) | 2994.96 (2775.39 to 3259.22) | 1570.42 (1398.54 to 1735.43) |
|  |  | 2021 year | 5805 (5056 to 6556) | 3749 (3253 to 4235) | 2056 (1763 to 2368) | 1467.93 (1278.74 to 1657.90) | 2131.26 (1849.35 to 2407.61) | 936.50 (803.16 to 1078.82) |
|  |  | rate of change（%） | -75.03 (-78.06 to -71.82) | -68.25 (-72.56 to -63.73) | -82.03 (-84.51 to -79.36) | NA | NA | NA |
|  | 75+ years |  |  |  |  |  |  |  |
|  |  | 1990 year | 23571 (21888 to 24924) | 6420 (6002 to 6816) | 17152 (15862 to 18254) | 29477.74 (27372.40 to 31169.03) | 30495.13 (28511.02 to 32376.61) | 29114.18 (26924.45 to 30986.07) |
|  |  | 2000 year | 18183 (16661 to 19299) | 4709 (4402 to 4988) | 13474 (12237 to 14429) | 24083.80 (22067.75 to 25562.28) | 24894.47 (23269.07 to 26365.19) | 23812.76 (21627.36 to 25501.38) |
|  |  | 2010 year | 9619 (8610 to 10415) | 3162 (2912 to 3418) | 6457 (5660 to 7059) | 9313.41 (8336.57 to 10083.49) | 11138.06 (10255.87 to 12037.66) | 8621.70 (7557.45 to 9425.46) |
|  |  | 2021 year | 8718 (7523 to 9746) | 3184 (2819 to 3587) | 5534 (4724 to 6204) | 6960.66 (6006.78 to 7781.37) | 8800.01 (7790.79 to 9914.56) | 6213.40 (5303.57 to 6965.19) |
|  |  | rate of change（%） | -63.01 (-67.24 to -59.10) | -50.40 (-56.17 to -44.44) | -67.74 (-71.92 to -63.99) | NA | NA | NA |
| Latvia |  |  |  |  |  |  |  |  |
|  | 0-14 years |  |  |  |  |  |  |  |
|  |  | 1990 year | 55 (42 to 69) | 22 (17 to 27) | 33 (24 to 44) | 9.67 (7.35 to 12.20) | 7.61 (5.99 to 9.41) | 11.81 (8.59 to 15.62) |
|  |  | 2000 year | 37 (27 to 49) | 14 (10 to 18) | 24 (16 to 33) | 8.68 (6.20 to 11.46) | 6.17 (4.46 to 8.03) | 11.30 (7.72 to 15.55) |
|  |  | 2010 year | 19 (13 to 26) | 7 (5 to 9) | 12 (8 to 17) | 6.55 (4.59 to 8.69) | 4.50 (3.21 to 5.91) | 8.71 (5.92 to 11.68) |
|  |  | 2021 year | 18 (13 to 26) | 6 (4 to 9) | 12 (8 to 17) | 6.17 (4.25 to 8.58) | 4.14 (2.88 to 5.57) | 8.34 (5.53 to 11.88) |
|  |  | rate of change（%） | -66.67 (-72.75 to -58.71) | -71.28 (-76.99 to -66.46) | -63.58 (-72.23 to -51.97) | NA | NA | NA |
|  | 15-49 years |  |  |  |  |  |  |  |
|  |  | 1990 year | 2790 (2436 to 3170) | 1639 (1401 to 1916) | 1151 (958 to 1345) | 217.04 (189.51 to 246.63) | 257.14 (219.92 to 300.70) | 177.61 (147.82 to 207.48) |
|  |  | 2000 year | 2281 (2014 to 2582) | 1284 (1116 to 1468) | 997 (848 to 1147) | 194.83 (172.06 to 220.53) | 222.14 (193.07 to 253.89) | 168.18 (143.17 to 193.66) |
|  |  | 2010 year | 1788 (1591 to 2008) | 1065 (933 to 1224) | 723 (611 to 852) | 172.58 (153.50 to 193.77) | 206.18 (180.63 to 237.06) | 139.18 (117.54 to 163.88) |
|  |  | 2021 year | 1007 (842 to 1170) | 609 (505 to 712) | 398 (315 to 487) | 127.70 (106.85 to 148.44) | 152.72 (126.77 to 178.54) | 102.12 (80.84 to 125.06) |
|  |  | rate of change（%） | -63.92 (-68.93 to -58.54) | -62.85 (-69.87 to -54.10) | -65.43 (-71.10 to -58.84) | NA | NA | NA |
|  | 50-74 years |  |  |  |  |  |  |  |
|  |  | 1990 year | 39654 (37237 to 42245) | 19421 (17867 to 21019) | 20233 (18624 to 21955) | 5971.45 (5607.46 to 6361.55) | 7208.90 (6632.09 to 7802.02) | 5126.74 (4719.02 to 5563.07) |
|  |  | 2000 year | 40611 (38262 to 42961) | 21630 (19988 to 23198) | 18980 (17775 to 20232) | 6207.52 (5848.49 to 6566.72) | 8072.08 (7459.11 to 8657.13) | 4913.99 (4601.79 to 5238.00) |
|  |  | 2010 year | 30644 (28521 to 32586) | 17640 (16439 to 18740) | 13003 (11958 to 14064) | 4936.47 (4594.55 to 5249.39) | 6852.95 (6386.05 to 7280.18) | 3578.74 (3290.99 to 3870.75) |
|  |  | 2021 year | 22436 (20049 to 25089) | 13125 (11532 to 14886) | 9311 (8209 to 10464) | 3763.88 (3363.43 to 4208.92) | 5099.80 (4480.97 to 5784.17) | 2748.88 (2423.42 to 3089.07) |
|  |  | rate of change（%） | -43.42 (-49.74 to -36.40) | -32.42 (-41.50 to -22.14) | -53.98 (-59.57 to -47.11) | NA | NA | NA |
|  | 75+ years |  |  |  |  |  |  |  |
|  |  | 1990 year | 46968 (43729 to 49635) | 13636 (12793 to 14431) | 33332 (30824 to 35429) | 33610.92 (31293.21 to 35519.70) | 34934.35 (32775.02 to 36971.56) | 33097.97 (30607.77 to 35180.23) |
|  |  | 2000 year | 39445 (36416 to 41610) | 10289 (9603 to 10916) | 29156 (26743 to 30990) | 30706.64 (28348.58 to 32391.73) | 32628.16 (30452.04 to 34614.52) | 30081.45 (27592.49 to 31973.51) |
|  |  | 2010 year | 36529 (33149 to 39127) | 10312 (9515 to 11052) | 26217 (23421 to 28370) | 22096.74 (20052.54 to 23668.48) | 23492.72 (21676.08 to 25178.30) | 21592.07 (19289.89 to 23365.37) |
|  |  | 2021 year | 42005 (36821 to 46171) | 12157 (10836 to 13515) | 29848 (25580 to 33032) | 22232.85 (19489.26 to 24437.76) | 23219.50 (20696.35 to 25812.96) | 21854.60 (18729.82 to 24185.78) |
|  |  | rate of change（%） | -10.57 (-19.64 to -1.36) | -10.84 (-20.05 to 0.06) | -10.45 (-20.00 to -1.06) | NA | NA | NA |
| Lithuania |  |  |  |  |  |  |  |  |
|  | 0-14 years |  |  |  |  |  |  |  |
|  |  | 1990 year | 86 (58 to 121) | 31 (22 to 42) | 55 (34 to 81) | 10.34 (6.99 to 14.58) | 7.30 (5.14 to 9.97) | 13.48 (8.30 to 19.82) |
|  |  | 2000 year | 68 (43 to 105) | 24 (17 to 32) | 44 (24 to 74) | 9.68 (6.11 to 14.85) | 6.64 (4.58 to 8.99) | 12.86 (7.07 to 21.51) |
|  |  | 2010 year | 39 (27 to 54) | 13 (9 to 17) | 26 (17 to 37) | 8.24 (5.64 to 11.43) | 5.46 (3.87 to 7.19) | 11.17 (7.32 to 15.97) |
|  |  | 2021 year | 31 (21 to 44) | 10 (7 to 15) | 21 (13 to 30) | 7.65 (5.04 to 10.77) | 4.98 (3.32 to 6.97) | 10.46 (6.67 to 14.92) |
|  |  | rate of change（%） | -63.67 (-71.06 to -54.43) | -66.30 (-70.46 to -61.97) | -62.20 (-72.31 to -46.39) | NA | NA | NA |
|  | 15-49 years |  |  |  |  |  |  |  |
|  |  | 1990 year | 3555 (2974 to 4129) | 1940 (1658 to 2264) | 1614 (1326 to 1910) | 194.17 (162.47 to 225.53) | 213.50 (182.39 to 249.12) | 175.13 (143.83 to 207.18) |
|  |  | 2000 year | 2956 (2516 to 3371) | 1647 (1403 to 1889) | 1309 (1066 to 1562) | 167.34 (142.41 to 190.80) | 189.03 (160.96 to 216.83) | 146.23 (119.01 to 174.43) |
|  |  | 2010 year | 2516 (2162 to 2899) | 1471 (1290 to 1670) | 1045 (834 to 1255) | 166.59 (143.19 to 191.96) | 196.15 (172.04 to 222.68) | 137.45 (109.69 to 165.09) |
|  |  | 2021 year | 1555 (1259 to 1856) | 940 (777 to 1099) | 616 (463 to 779) | 134.64 (108.95 to 160.66) | 161.40 (133.45 to 188.68) | 107.44 (80.78 to 135.97) |
|  |  | rate of change（%） | -56.24 (-62.86 to -48.70) | -51.56 (-60.81 to -41.47) | -61.87 (-68.16 to -54.25) | NA | NA | NA |
|  | 50-74 years |  |  |  |  |  |  |  |
|  |  | 1990 year | 33649 (31291 to 35903) | 16857 (15788 to 17967) | 16792 (15410 to 18129) | 4012.96 (3731.68 to 4281.77) | 4832.02 (4525.47 to 5150.28) | 3429.40 (3147.15 to 3702.38) |
|  |  | 2000 year | 37134 (34740 to 39528) | 18933 (17626 to 20278) | 18201 (16742 to 19649) | 4242.27 (3968.74 to 4515.82) | 5194.83 (4836.08 to 5563.94) | 3562.70 (3277.14 to 3846.12) |
|  |  | 2010 year | 35879 (32971 to 39037) | 20514 (19000 to 22097) | 15365 (13582 to 17185) | 4143.61 (3807.84 to 4508.37) | 5615.15 (5200.85 to 6048.45) | 3069.61 (2713.40 to 3433.22) |
|  |  | 2021 year | 23003 (20132 to 25740) | 14392 (12689 to 16005) | 8611 (7387 to 9771) | 2580.74 (2258.60 to 2887.79) | 3727.95 (3286.77 to 4145.81) | 1704.23 (1461.95 to 1933.74) |
|  |  | rate of change（%） | -31.64 (-40.10 to -23.54) | -14.62 (-25.87 to -3.41) | -48.72 (-55.94 to -41.86) | NA | NA | NA |
|  | 75+ years |  |  |  |  |  |  |  |
|  |  | 1990 year | 28695 (26395 to 30527) | 9349 (8691 to 9986) | 19346 (17630 to 20814) | 16458.38 (15139.47 to 17509.16) | 16504.29 (15342.93 to 17629.15) | 16436.29 (14978.80 to 17683.67) |
|  |  | 2000 year | 32055 (29365 to 33976) | 9499 (8820 to 10124) | 22556 (20481 to 24100) | 18510.63 (16957.54 to 19619.81) | 18984.23 (17625.98 to 20233.03) | 18318.18 (16633.29 to 19572.36) |
|  |  | 2010 year | 45529 (41237 to 49346) | 14667 (13575 to 15749) | 30862 (27191 to 33981) | 18641.88 (16884.48 to 20204.79) | 20531.93 (19003.00 to 22046.75) | 17860.52 (15736.14 to 19665.17) |
|  |  | 2021 year | 36260 (31921 to 40065) | 11382 (10129 to 12677) | 24878 (21568 to 27695) | 13239.00 (11654.77 to 14628.18) | 14311.95 (12736.42 to 15940.21) | 12799.99 (11096.63 to 14249.45) |
|  |  | rate of change（%） | 26.37 (13.15 to 39.32) | 21.75 (8.23 to 34.82) | 28.60 (13.66 to 42.57) | NA | NA | NA |
| Republic of Moldova | | |  |  |  |  |  |  |
|  | 0-14 years |  |  |  |  |  |  |  |
|  |  | 1990 year | 126 (98 to 157) | 51 (40 to 62) | 75 (57 to 97) | 10.19 (7.89 to 12.68) | 8.06 (6.42 to 9.79) | 12.38 (9.40 to 15.93) |
|  |  | 2000 year | 87 (65 to 111) | 32 (25 to 40) | 54 (39 to 72) | 9.41 (7.06 to 12.08) | 6.84 (5.23 to 8.41) | 12.08 (8.74 to 16.06) |
|  |  | 2010 year | 46 (34 to 59) | 16 (12 to 21) | 29 (21 to 39) | 7.77 (5.69 to 10.04) | 5.42 (4.11 to 6.87) | 10.25 (7.15 to 13.74) |
|  |  | 2021 year | 36 (25 to 50) | 13 (9 to 17) | 24 (16 to 33) | 6.96 (4.84 to 9.55) | 4.70 (3.29 to 6.24) | 9.36 (6.19 to 13.15) |
|  |  | rate of change（%） | -71.11 (-77.13 to -64.06) | -75.08 (-80.28 to -70.23) | -68.43 (-76.18 to -58.71) | NA | NA | NA |
|  | 15-49 years |  |  |  |  |  |  |  |
|  |  | 1990 year | 2653 (2190 to 3134) | 1298 (1028 to 1559) | 1355 (1088 to 1644) | 120.73 (99.67 to 142.65) | 121.08 (95.92 to 145.49) | 120.40 (96.70 to 146.07) |
|  |  | 2000 year | 3194 (2732 to 3642) | 1710 (1442 to 1967) | 1484 (1230 to 1747) | 141.06 (120.67 to 160.84) | 153.03 (129.07 to 176.07) | 129.41 (107.20 to 152.36) |
|  |  | 2010 year | 3375 (2980 to 3840) | 1980 (1724 to 2267) | 1395 (1190 to 1643) | 162.31 (143.34 to 184.70) | 190.33 (165.71 to 217.96) | 134.26 (114.52 to 158.21) |
|  |  | 2021 year | 2141 (1769 to 2549) | 1209 (1012 to 1429) | 932 (744 to 1146) | 119.62 (98.83 to 142.40) | 133.18 (111.50 to 157.45) | 105.67 (84.30 to 129.96) |
|  |  | rate of change（%） | -19.28 (-30.62 to -5.89) | -6.82 (-24.76 to 15.70) | -31.21 (-42.10 to -18.60) | NA | NA | NA |
|  | 50-74 years |  |  |  |  |  |  |  |
|  |  | 1990 year | 25838 (22699 to 28725) | 12203 (10126 to 13948) | 13635 (11553 to 15447) | 2916.04 (2561.83 to 3241.94) | 3228.57 (2679.09 to 3690.20) | 2683.54 (2273.85 to 3040.16) |
|  |  | 2000 year | 34054 (31918 to 36333) | 17134 (15717 to 18681) | 16920 (15816 to 18017) | 3923.78 (3677.69 to 4186.37) | 4582.99 (4204.13 to 4996.88) | 3424.94 (3201.29 to 3646.90) |
|  |  | 2010 year | 43965 (41500 to 46781) | 23820 (22162 to 25658) | 20145 (18896 to 21453) | 4436.12 (4187.40 to 4720.26) | 5499.13 (5116.44 to 5923.49) | 3610.83 (3386.81 to 3845.13) |
|  |  | 2021 year | 37939 (34439 to 42119) | 21424 (19081 to 23907) | 16515 (14847 to 18435) | 3538.72 (3212.25 to 3928.58) | 4609.99 (4105.91 to 5144.19) | 2719.06 (2444.33 to 3035.17) |
|  |  | rate of change（%） | 46.84 (28.73 to 72.24) | 75.56 (48.86 to 114.53) | 21.13 (4.78 to 44.56) | NA | NA | NA |
|  | 75+ years |  |  |  |  |  |  |  |
|  |  | 1990 year | 24047 (21939 to 25977) | 8374 (7404 to 9234) | 15674 (13988 to 17210) | 18743.66 (17100.51 to 20247.72) | 18918.41 (16727.06 to 20861.14) | 18651.62 (16645.44 to 20479.64) |
|  |  | 2000 year | 20811 (19408 to 21875) | 7725 (7262 to 8147) | 13086 (11965 to 13932) | 14536.72 (13556.30 to 15279.82) | 16580.56 (15587.90 to 17486.03) | 13550.71 (12389.37 to 14426.48) |
|  |  | 2010 year | 28932 (26885 to 30387) | 11290 (10493 to 12113) | 17642 (16141 to 18785) | 15488.51 (14392.70 to 16267.43) | 17802.30 (16545.37 to 19100.41) | 14299.19 (13082.56 to 15225.44) |
|  |  | 2021 year | 23024 (20658 to 25412) | 8581 (7730 to 9414) | 14443 (12663 to 16061) | 11010.38 (9878.98 to 12152.66) | 12341.40 (11117.78 to 13540.25) | 10347.38 (9072.01 to 11506.66) |
|  |  | rate of change（%） | -4.26 (-13.56 to 8.60) | 2.47 (-9.77 to 19.82) | -7.85 (-19.29 to 4.62) | NA | NA | NA |
| Russian Federation | | |  |  |  |  |  |  |
|  | 0-14 years |  |  |  |  |  |  |  |
|  |  | 1990 year | 3727 (2826 to 4708) | 1399 (1097 to 1735) | 2327 (1716 to 3004) | 10.74 (8.14 to 13.57) | 7.93 (6.22 to 9.83) | 13.65 (10.07 to 17.62) |
|  |  | 2000 year | 2913 (2149 to 3841) | 1032 (796 to 1298) | 1881 (1352 to 2539) | 10.90 (8.04 to 14.37) | 7.56 (5.83 to 9.50) | 14.39 (10.34 to 19.42) |
|  |  | 2010 year | 1783 (1306 to 2329) | 577 (435 to 730) | 1205 (868 to 1591) | 7.94 (5.82 to 10.38) | 5.02 (3.78 to 6.35) | 11.02 (7.94 to 14.54) |
|  |  | 2021 year | 1942 (1356 to 2658) | 597 (419 to 801) | 1345 (912 to 1865) | 7.45 (5.20 to 10.19) | 4.47 (3.13 to 5.99) | 10.59 (7.18 to 14.69) |
|  |  | rate of change（%） | -47.88 (-53.11 to -42.40) | -57.31 (-62.35 to -53.66) | -42.21 (-49.26 to -34.38) | NA | NA | NA |
|  | 15-49 years |  |  |  |  |  |  |  |
|  |  | 1990 year | 187896 (170436 to 206054) | 107933 (101036 to 115361) | 79962 (69607 to 91211) | 253.16 (229.63 to 277.62) | 290.18 (271.64 to 310.15) | 215.97 (188.00 to 246.35) |
|  |  | 2000 year | 289772 (272481 to 309616) | 182343 (174770 to 190615) | 107429 (96608 to 119217) | 359.09 (337.66 to 383.68) | 455.55 (436.63 to 476.21) | 264.16 (237.55 to 293.14) |
|  |  | 2010 year | 220156 (204664 to 235777) | 137055 (130065 to 144015) | 83101 (73387 to 92398) | 292.39 (271.81 to 313.13) | 367.84 (349.08 to 386.52) | 218.47 (192.94 to 242.92) |
|  |  | 2021 year | 159348 (144382 to 177738) | 96892 (87758 to 106287) | 62456 (53430 to 72891) | 236.29 (214.10 to 263.56) | 288.85 (261.62 to 316.86) | 184.27 (157.64 to 215.06) |
|  |  | rate of change（%） | -15.19 (-20.33 to -9.64) | -10.23 (-18.39 to -2.61) | -21.89 (-27.87 to -16.29) | NA | NA | NA |
|  | 50-74 years |  |  |  |  |  |  |  |
|  |  | 1990 year | 2544746 (2466831 to 2619890) | 1190376 (1160053 to 1220747) | 1354370 (1303765 to 1401465) | 7098.39 (6881.05 to 7308.00) | 8208.39 (7999.29 to 8417.82) | 6344.34 (6107.29 to 6564.95) |
|  |  | 2000 year | 3913612 (3835467 to 3993751) | 2030097 (1996729 to 2065835) | 1883514 (1830694 to 1935781) | 10920.46 (10702.41 to 11144.08) | 13742.64 (13516.75 to 13984.56) | 8941.37 (8690.62 to 9189.49) |
|  |  | 2010 year | 2642143 (2574910 to 2712283) | 1461872 (1430931 to 1492798) | 1180271 (1137578 to 1223177) | 6638.81 (6469.88 to 6815.05) | 8830.44 (8643.54 to 9017.25) | 5077.85 (4894.18 to 5262.45) |
|  |  | 2021 year | 1987864 (1828771 to 2151498) | 1128234 (1000091 to 1251906) | 859630 (766704 to 943769) | 4575.90 (4209.68 to 4952.57) | 6138.50 (5441.30 to 6811.38) | 3429.96 (3059.18 to 3765.68) |
|  |  | rate of change（%） | -21.88 (-27.53 to -15.67) | -5.22 (-15.47 to 4.10) | -36.53 (-42.43 to -31.01) | NA | NA | NA |
|  | 75+ years |  |  |  |  |  |  |  |
|  |  | 1990 year | 2398315 (2254612 to 2465329) | 579722 (558593 to 591741) | 1818593 (1695431 to 1875042) | 38582.12 (36270.34 to 39660.18) | 42014.27 (40482.93 to 42885.28) | 37602.91 (35056.30 to 38770.09) |
|  |  | 2000 year | 2695092 (2539329 to 2770513) | 624557 (600040 to 636570) | 2070535 (1937098 to 2133498) | 45271.62 (42655.14 to 46538.52) | 47539.21 (45673.02 to 48453.59) | 44629.48 (41753.31 to 45986.62) |
|  |  | 2010 year | 2461558 (2304730 to 2536850) | 693857 (665383 to 709284) | 1767700 (1636156 to 1828105) | 31502.84 (29495.76 to 32466.42) | 33156.90 (31796.23 to 33894.10) | 30897.82 (28598.54 to 31953.65) |
|  |  | 2021 year | 1978854 (1785280 to 2120138) | 528715 (480232 to 575031) | 1450139 (1281167 to 1564069) | 25058.82 (22607.55 to 26847.95) | 24631.40 (22372.71 to 26789.16) | 25218.38 (22279.90 to 27199.65) |
|  |  | rate of change（%） | -17.49 (-22.63 to -12.78) | -8.80 (-16.83 to -1.75) | -20.26 (-26.13 to -14.84) | NA | NA | NA |
| Ukraine |  |  |  |  |  |  |  |  |
|  | 0-14 years |  |  |  |  |  |  |  |
|  |  | 1990 year | 1131 (717 to 1629) | 415 (301 to 551) | 716 (392 to 1113) | 9.94 (6.30 to 14.32) | 7.17 (5.20 to 9.51) | 12.82 (7.03 to 19.94) |
|  |  | 2000 year | 900 (561 to 1327) | 307 (222 to 411) | 593 (324 to 977) | 10.42 (6.50 to 15.36) | 6.94 (5.02 to 9.27) | 14.07 (7.70 to 23.19) |
|  |  | 2010 year | 545 (346 to 786) | 176 (126 to 231) | 369 (211 to 558) | 8.28 (5.25 to 11.94) | 5.20 (3.71 to 6.82) | 11.53 (6.60 to 17.45) |
|  |  | 2021 year | 578 (351 to 862) | 176 (120 to 240) | 401 (230 to 643) | 9.11 (5.54 to 13.59) | 5.40 (3.67 to 7.35) | 13.04 (7.47 to 20.90) |
|  |  | rate of change（%） | -48.90 (-65.34 to -26.45) | -57.50 (-63.85 to -52.45) | -43.91 (-68.24 to -3.48) | NA | NA | NA |
|  | 15-49 years |  |  |  |  |  |  |  |
|  |  | 1990 year | 53360 (44548 to 62949) | 30413 (24843 to 36904) | 22947 (18496 to 27709) | 213.85 (178.54 to 252.29) | 247.40 (202.09 to 300.20) | 181.28 (146.11 to 218.90) |
|  |  | 2000 year | 75676 (65559 to 84970) | 48507 (41079 to 54818) | 27168 (22744 to 31623) | 297.45 (257.68 to 333.98) | 386.96 (327.69 to 437.30) | 210.51 (176.23 to 245.02) |
|  |  | 2010 year | 56757 (50141 to 63130) | 35626 (31549 to 39718) | 21131 (17267 to 24757) | 242.02 (213.80 to 269.19) | 306.75 (271.65 to 341.98) | 178.50 (145.86 to 209.14) |
|  |  | 2021 year | 47699 (36498 to 58650) | 30758 (21822 to 40304) | 16942 (12120 to 22087) | 235.83 (180.45 to 289.97) | 303.90 (215.61 to 398.22) | 167.65 (119.94 to 218.57) |
|  |  | rate of change（%） | -10.61 (-30.14 to 14.90) | 1.13 (-28.57 to 43.75) | -26.17 (-42.85 to -6.44) | NA | NA | NA |
|  | 50-74 years |  |  |  |  |  |  |  |
|  |  | 1990 year | 834697 (775793 to 886370) | 389064 (355005 to 424275) | 445633 (411283 to 478262) | 6052.11 (5625.02 to 6426.77) | 6895.09 (6291.48 to 7519.12) | 5468.41 (5046.91 to 5868.81) |
|  |  | 2000 year | 1013197 (948240 to 1076238) | 532281 (494792 to 571119) | 480916 (445726 to 514996) | 7698.05 (7204.52 to 8177.02) | 9716.33 (9032.00 to 10425.29) | 6259.06 (5801.06 to 6702.60) |
|  |  | 2010 year | 705545 (667050 to 745349) | 375086 (350476 to 398686) | 330458 (309324 to 353540) | 5329.13 (5038.37 to 5629.78) | 6842.00 (6393.08 to 7272.48) | 4259.98 (3987.53 to 4557.53) |
|  |  | 2021 year | 561562 (436114 to 700431) | 310685 (218433 to 425931) | 250876 (181031 to 344679) | 4132.39 (3209.26 to 5154.30) | 5439.26 (3824.16 to 7456.90) | 3184.78 (2298.12 to 4375.57) |
|  |  | rate of change（%） | -32.72 (-47.81 to -15.28) | -20.15 (-44.60 to 9.65) | -43.70 (-59.32 to -22.37) | NA | NA | NA |
|  | 75+ years |  |  |  |  |  |  |  |
|  |  | 1990 year | 888416 (837859 to 926322) | 244420 (228208 to 259337) | 643996 (601351 to 677556) | 34238.91 (32290.46 to 35699.76) | 36122.64 (33726.61 to 38327.10) | 33574.40 (31351.13 to 35324.04) |
|  |  | 2000 year | 746159 (702309 to 780603) | 196445 (183670 to 208852) | 549714 (511920 to 578292) | 31425.36 (29578.56 to 32876.00) | 32821.50 (30687.14 to 34894.46) | 30954.82 (28826.58 to 32564.07) |
|  |  | 2010 year | 684340 (642678 to 717956) | 217104 (204672 to 230217) | 467236 (432951 to 495355) | 24424.53 (22937.58 to 25624.32) | 26337.98 (24829.71 to 27928.73) | 23626.95 (21893.26 to 25048.90) |
|  |  | 2021 year | 545023 (434543 to 669042) | 170675 (133250 to 217446) | 374348 (270807 to 487040) | 18683.40 (14896.12 to 22934.76) | 19931.34 (15560.85 to 25393.22) | 18164.85 (13140.65 to 23633.13) |
|  |  | rate of change（%） | -38.65 (-50.19 to -25.10) | -30.17 (-46.83 to -10.47) | -41.87 (-55.97 to -23.94) | NA | NA | NA |

DALYs, disability-adjusted life years; 95% UI: 95% uncertainty interval.
