# Supplementary material for: The burden of ischemic stroke in Eastern Europe from 1990 to 2021
Source: BMC Neurol. 2025 Feb 22;25:74. doi: 10.1186/s12883-025-04081-z (PMC11846382; doi:10.1186/s12883-025-04081-z)
Supplement: Supplementary file 2 — Supplementary Material 2 [file 12883_2025_4081_MOESM2_ESM.zip › Supplementary Table 1-10/Supplementary Table 7.docx]

Supplementary Table 7. Annual percentage change in age-standardized incidence rates of ischemic stroke across Eastern European countries from 1990 to 2021.

| **location** | **sex** | **Segment Start** | **Segment End** | **APC**  **(95% UI)** | **P-Value** |
| --- | --- | --- | --- | --- | --- |
| Eastern Europe | Both | 1990 | 1994 | -1.54 (-2.02 to -1.06) | <0.001 |
| Eastern Europe | Both | 1994 | 2003 | -0.30 (-0.45 to -0.15) | 0.001 |
| Eastern Europe | Both | 2003 | 2010 | -2.47 (-2.69 to -2.24) | <0.001 |
| Eastern Europe | Both | 2010 | 2014 | -1.15 (-1.79 to -0.50) | 0.002 |
| Eastern Europe | Both | 2014 | 2019 | 0.52 (0.10 to 0.95) | 0.018 |
| Eastern Europe | Both | 2019 | 2021 | -2.26 (-3.61 to -0.89) | 0.003 |
| Eastern Europe | Female | 1990 | 1994 | -1.92 (-2.34 to -1.49) | <0.001 |
| Eastern Europe | Female | 1994 | 2002 | -0.50 (-0.67 to -0.33) | <0.001 |
| Eastern Europe | Female | 2002 | 2010 | -2.36 (-2.52 to -2.20) | <0.001 |
| Eastern Europe | Female | 2010 | 2014 | -0.89 (-1.49 to -0.29) | 0.007 |
| Eastern Europe | Female | 2014 | 2019 | 0.63 (0.23 to 1.02) | 0.004 |
| Eastern Europe | Female | 2019 | 2021 | -2.12 (-3.36 to -0.88) | 0.003 |
| Eastern Europe | Male | 1990 | 1994 | -1.49 (-2.12 to -0.86) | <0.001 |
| Eastern Europe | Male | 1994 | 2003 | -0.07 (-0.28 to 0.13) | 0.453 |
| Eastern Europe | Male | 2003 | 2013 | -2.27 (-2.43 to -2.11) | <0.001 |
| Eastern Europe | Male | 2013 | 2019 | 0.23 (-0.15 to 0.61) | 0.225 |
| Eastern Europe | Male | 2019 | 2021 | -2.39 (-4.16 to -0.58) | 0.013 |
| Belarus | Both | 1990 | 2005 | 0.07 (0.05 to 0.09) | <0.001 |
| Belarus | Both | 2005 | 2010 | -1.39 (-1.54 to -1.23) | <0.001 |
| Belarus | Both | 2010 | 2014 | -3.41 (-3.67 to -3.15) | <0.001 |
| Belarus | Both | 2014 | 2019 | -0.24 (-0.44 to -0.05) | 0.016 |
| Belarus | Both | 2019 | 2021 | -2.77 (-3.39 to -2.15) | <0.001 |
| Belarus | Female | 1990 | 1993 | -1.22 (-1.42 to -1.02) | <0.001 |
| Belarus | Female | 1993 | 2005 | -0.55 (-0.57 to -0.53) | <0.001 |
| Belarus | Female | 2005 | 2010 | -1.80 (-1.90 to -1.69) | <0.001 |
| Belarus | Female | 2010 | 2014 | -2.25 (-2.43 to -2.07) | <0.001 |
| Belarus | Female | 2014 | 2019 | -0.12 (-0.25 to 0.02) | 0.082 |
| Belarus | Female | 2019 | 2021 | -2.03 (-2.43 to -1.63) | <0.001 |
| Belarus | Male | 1990 | 2006 | 0.78 (0.75 to 0.81) | <0.001 |
| Belarus | Male | 2006 | 2011 | -1.69 (-1.92 to -1.46) | <0.001 |
| Belarus | Male | 2011 | 2014 | -5.44 (-6.14 to -4.74) | <0.001 |
| Belarus | Male | 2014 | 2019 | -0.38 (-0.64 to -0.11) | 0.008 |
| Belarus | Male | 2019 | 2021 | -3.66 (-4.51 to -2.81) | <0.001 |
| Estonia | Both | 1990 | 1995 | -1.74 (-1.84 to -1.65) | <0.001 |
| Estonia | Both | 1995 | 2003 | -2.18 (-2.23 to -2.13) | <0.001 |
| Estonia | Both | 2003 | 2006 | -2.84 (-3.33 to -2.34) | <0.001 |
| Estonia | Both | 2006 | 2014 | -5.72 (-5.79 to -5.65) | <0.001 |
| Estonia | Both | 2014 | 2019 | 0.00 (-0.20 to 0.19) | 0.969 |
| Estonia | Both | 2019 | 2021 | -2.61 (-3.31 to -1.92) | <0.001 |
| Estonia | Female | 1990 | 2001 | -2.43 (-2.49 to -2.37) | <0.001 |
| Estonia | Female | 2001 | 2005 | -1.45 (-1.91 to -0.99) | <0.001 |
| Estonia | Female | 2005 | 2014 | -5.44 (-5.55 to -5.32) | <0.001 |
| Estonia | Female | 2014 | 2019 | 0.04 (-0.34 to 0.42) | 0.822 |
| Estonia | Female | 2019 | 2021 | -1.47 (-2.77 to -0.16) | 0.03 |
| Estonia | Male | 1990 | 1996 | -1.17 (-1.22 to -1.12) | <0.001 |
| Estonia | Male | 1996 | 2002 | -2.00 (-2.06 to -1.93) | <0.001 |
| Estonia | Male | 2002 | 2006 | -2.96 (-3.15 to -2.78) | <0.001 |
| Estonia | Male | 2006 | 2014 | -5.88 (-5.94 to -5.82) | <0.001 |
| Estonia | Male | 2014 | 2019 | -0.24 (-0.41 to -0.07) | 0.01 |
| Estonia | Male | 2019 | 2021 | -3.83 (-4.37 to -3.27) | <0.001 |
| Latvia | Both | 1990 | 1996 | -1.45 (-1.63 to -1.26) | <0.001 |
| Latvia | Both | 1996 | 1999 | -2.14 (-2.98 to -1.30) | <0.001 |
| Latvia | Both | 1999 | 2010 | -0.75 (-0.81 to -0.68) | <0.001 |
| Latvia | Both | 2010 | 2014 | -1.91 (-2.30 to -1.52) | <0.001 |
| Latvia | Both | 2014 | 2021 | -1.39 (-1.52 to -1.26) | <0.001 |
| Latvia | Female | 1990 | 1995 | -1.82 (-1.95 to -1.70) | <0.001 |
| Latvia | Female | 1995 | 2000 | -2.23 (-2.39 to -2.08) | <0.001 |
| Latvia | Female | 2000 | 2009 | -1.18 (-1.23 to -1.13) | <0.001 |
| Latvia | Female | 2009 | 2015 | -1.77 (-1.88 to -1.66) | <0.001 |
| Latvia | Female | 2015 | 2018 | -0.25 (-0.75 to 0.25) | 0.298 |
| Latvia | Female | 2018 | 2021 | -0.99 (-1.28 to -0.69) | <0.001 |
| Latvia | Male | 1990 | 2000 | -1.21 (-1.34 to -1.08) | <0.001 |
| Latvia | Male | 2000 | 2010 | -0.16 (-0.28 to -0.04) | 0.013 |
| Latvia | Male | 2010 | 2021 | -2.20 (-2.31 to -2.10) | <0.001 |
| Lithuania | Both | 1990 | 1995 | 0.80 (0.21 to 1.39) | 0.011 |
| Lithuania | Both | 1995 | 2000 | -0.42 (-0.94 to 0.10) | 0.103 |
| Lithuania | Both | 2000 | 2005 | 0.69 (0.24 to 1.13) | 0.005 |
| Lithuania | Both | 2005 | 2010 | -0.64 (-1.08 to -0.20) | 0.008 |
| Lithuania | Both | 2010 | 2014 | -2.21 (-3.13 to -1.28) | <0.001 |
| Lithuania | Both | 2014 | 2021 | -4.17 (-4.58 to -3.77) | <0.001 |
| Lithuania | Female | 1990 | 1994 | 0.51 (0.27 to 0.75) | <0.001 |
| Lithuania | Female | 1994 | 2006 | -0.12 (-0.15 to -0.09) | <0.001 |
| Lithuania | Female | 2006 | 2010 | -1.40 (-1.64 to -1.15) | <0.001 |
| Lithuania | Female | 2010 | 2013 | -2.70 (-3.27 to -2.12) | <0.001 |
| Lithuania | Female | 2013 | 2019 | -3.56 (-3.73 to -3.39) | <0.001 |
| Lithuania | Female | 2019 | 2021 | -1.01 (-1.95 to -0.07) | 0.037 |
| Lithuania | Male | 1990 | 1994 | 1.93 (0.85 to 3.03) | 0.002 |
| Lithuania | Male | 1994 | 2000 | -0.56 (-1.04 to -0.08) | 0.025 |
| Lithuania | Male | 2000 | 2005 | 1.47 (0.96 to 1.98) | <0.001 |
| Lithuania | Male | 2005 | 2011 | -0.06 (-0.42 to 0.30) | 0.714 |
| Lithuania | Male | 2011 | 2015 | -2.34 (-3.44 to -1.23) | <0.001 |
| Lithuania | Male | 2015 | 2021 | -5.99 (-6.61 to -5.37) | <0.001 |
| Republic of Moldova | Both | 1990 | 1996 | -1.51 (-1.72 to -1.30) | <0.001 |
| Republic of Moldova | Both | 1996 | 2010 | 0.34 (0.27 to 0.40) | <0.001 |
| Republic of Moldova | Both | 2010 | 2014 | -1.78 (-2.39 to -1.16) | <0.001 |
| Republic of Moldova | Both | 2014 | 2019 | -0.10 (-0.49 to 0.30) | 0.607 |
| Republic of Moldova | Both | 2019 | 2021 | -2.24 (-3.53 to -0.94) | 0.002 |
| Republic of Moldova | Female | 1990 | 1994 | -2.10 (-2.26 to -1.93) | <0.001 |
| Republic of Moldova | Female | 1994 | 1999 | -1.06 (-1.22 to -0.89) | <0.001 |
| Republic of Moldova | Female | 1999 | 2010 | -0.06 (-0.11 to -0.02) | 0.006 |
| Republic of Moldova | Female | 2010 | 2014 | -1.50 (-1.75 to -1.25) | <0.001 |
| Republic of Moldova | Female | 2014 | 2019 | -0.01 (-0.17 to 0.15) | 0.924 |
| Republic of Moldova | Female | 2019 | 2021 | -2.15 (-2.70 to -1.60) | <0.001 |
| Republic of Moldova | Male | 1990 | 1996 | -0.94 (-1.24 to -0.63) | <0.001 |
| Republic of Moldova | Male | 1996 | 2006 | 1.02 (0.86 to 1.18) | <0.001 |
| Republic of Moldova | Male | 2006 | 2010 | 0.31 (-0.57 to 1.18) | 0.468 |
| Republic of Moldova | Male | 2010 | 2015 | -1.84 (-2.39 to -1.28) | <0.001 |
| Republic of Moldova | Male | 2015 | 2018 | 0.34 (-1.43 to 2.15) | 0.691 |
| Republic of Moldova | Male | 2018 | 2021 | -1.66 (-2.62 to -0.69) | 0.002 |
| Russian Federation | Both | 1990 | 1994 | -2.27 (-2.85 to -1.68) | <0.001 |
| Russian Federation | Both | 1994 | 2003 | -0.05 (-0.24 to 0.14) | 0.582 |
| Russian Federation | Both | 2003 | 2010 | -2.70 (-2.99 to -2.42) | <0.001 |
| Russian Federation | Both | 2010 | 2014 | -0.50 (-1.33 to 0.33) | 0.217 |
| Russian Federation | Both | 2014 | 2019 | 1.07 (0.53 to 1.60) | 0.001 |
| Russian Federation | Both | 2019 | 2021 | -2.85 (-4.53 to -1.13) | 0.003 |
| Russian Federation | Female | 1990 | 1994 | -2.39 (-2.88 to -1.89) | <0.001 |
| Russian Federation | Female | 1994 | 2003 | -0.35 (-0.51 to -0.19) | <0.001 |
| Russian Federation | Female | 2003 | 2010 | -2.69 (-2.92 to -2.45) | <0.001 |
| Russian Federation | Female | 2010 | 2014 | -0.17 (-0.86 to 0.54) | 0.621 |
| Russian Federation | Female | 2014 | 2019 | 1.13 (0.68 to 1.59) | <0.001 |
| Russian Federation | Female | 2019 | 2021 | -2.69 (-4.13 to -1.23) | 0.001 |
| Russian Federation | Male | 1990 | 1994 | -2.46 (-3.08 to -1.83) | <0.001 |
| Russian Federation | Male | 1994 | 2004 | -0.08 (-0.25 to 0.09) | 0.337 |
| Russian Federation | Male | 2004 | 2010 | -3.12 (-3.51 to -2.72) | <0.001 |
| Russian Federation | Male | 2010 | 2014 | -0.68 (-1.55 to 0.19) | 0.115 |
| Russian Federation | Male | 2014 | 2019 | 0.93 (0.36 to 1.49) | 0.003 |
| Russian Federation | Male | 2019 | 2021 | -3.05 (-4.82 to -1.25) | 0.003 |
| Ukraine | Both | 1990 | 1998 | -0.41 (-0.43 to -0.38) | <0.001 |
| Ukraine | Both | 1998 | 2001 | -0.77 (-1.00 to -0.54) | <0.001 |
| Ukraine | Both | 2001 | 2006 | -2.13 (-2.20 to -2.06) | <0.001 |
| Ukraine | Both | 2006 | 2009 | -2.66 (-2.88 to -2.44) | <0.001 |
| Ukraine | Both | 2009 | 2014 | -2.20 (-2.26 to -2.13) | <0.001 |
| Ukraine | Both | 2014 | 2021 | -0.32 (-0.35 to -0.29) | <0.001 |
| Ukraine | Female | 1990 | 1999 | -0.86 (-0.89 to -0.83) | <0.001 |
| Ukraine | Female | 1999 | 2002 | -1.60 (-1.95 to -1.25) | <0.001 |
| Ukraine | Female | 2002 | 2010 | -2.45 (-2.50 to -2.41) | <0.001 |
| Ukraine | Female | 2010 | 2014 | -2.01 (-2.17 to -1.85) | <0.001 |
| Ukraine | Female | 2014 | 2021 | -0.45 (-0.50 to -0.41) | <0.001 |
| Ukraine | Male | 1990 | 2000 | -0.03 (-0.08 to 0.02) | 0.235 |
| Ukraine | Male | 2000 | 2003 | -1.46 (-2.04 to -0.88) | <0.001 |
| Ukraine | Male | 2003 | 2006 | -2.15 (-2.71 to -1.58) | <0.001 |
| Ukraine | Male | 2006 | 2010 | -2.77 (-3.05 to -2.48) | <0.001 |
| Ukraine | Male | 2010 | 2014 | -2.38 (-2.64 to -2.11) | <0.001 |
| Ukraine | Male | 2014 | 2021 | -0.21 (-0.28 to -0.14) | <0.001 |

APC, Annual percentage change (Positive APC values indicate an increasing trend in ASIR, while negative APC values indicate a decreasing trend.); ASIR, age-standardized incidence rate; P-value: Statistical significance level. P-values < 0.05 indicate a statistically significant trend; 95% UI: 95% uncertainty interval.
