# Supplementary material for: The burden of ischemic stroke in Eastern Europe from 1990 to 2021
Source: BMC Neurol. 2025 Feb 22;25:74. doi: 10.1186/s12883-025-04081-z (PMC11846382; doi:10.1186/s12883-025-04081-z)
Supplement: Supplementary file 2 — Supplementary Material 2 [file 12883_2025_4081_MOESM2_ESM.zip › Supplementary Table 1-10/Supplementary Table 8.docx]

Supplementary Table 8. Annual percentage change in age-standardized mortality rates of ischemic stroke across Eastern European countries from 1990 to 2021.

| **location** | **sex** | **Segment Start** | **Segment End** | **APC**  **(95% UI)** | **P-Value** |
| --- | --- | --- | --- | --- | --- |
| Eastern Europe | Both | 1990 | 1994 | 6.27 (4.58 to 7.98) | <0.001 |
| Eastern Europe | Both | 1994 | 1997 | -2.98 (-7.45 to 1.71) | 0.195 |
| Eastern Europe | Both | 1997 | 2003 | 0.59 (-0.50 to 1.69) | 0.274 |
| Eastern Europe | Both | 2003 | 2012 | -5.84 (-6.37 to -5.31) | <0.001 |
| Eastern Europe | Both | 2012 | 2021 | -2.84 (-3.47 to -2.20) | <0.001 |
| Eastern Europe | Female | 1990 | 1994 | 5.67 (4.08 to 7.28) | <0.001 |
| Eastern Europe | Female | 1994 | 1997 | -2.76 (-7.07 to 1.75) | 0.211 |
| Eastern Europe | Female | 1997 | 2003 | 0.35 (-0.71 to 1.42) | 0.498 |
| Eastern Europe | Female | 2003 | 2012 | -6.09 (-6.62 to -5.56) | <0.001 |
| Eastern Europe | Female | 2012 | 2021 | -2.87 (-3.53 to -2.20) | <0.001 |
| Eastern Europe | Male | 1990 | 1994 | 6.88 (4.98 to 8.82) | <0.001 |
| Eastern Europe | Male | 1994 | 1997 | -3.45 (-7.99 to 1.32) | 0.144 |
| Eastern Europe | Male | 1997 | 2003 | 0.46 (-0.63 to 1.56) | 0.389 |
| Eastern Europe | Male | 2003 | 2012 | -5.49 (-6.02 to -4.97) | <0.001 |
| Eastern Europe | Male | 2012 | 2021 | -2.82 (-3.59 to -2.04) | <0.001 |
| Belarus | Both | 1990 | 1993 | 5.08 (1.14 to 9.18) | 0.014 |
| Belarus | Both | 1993 | 2005 | 0.04 (-0.43 to 0.52) | 0.851 |
| Belarus | Both | 2005 | 2015 | -4.38 (-4.98 to -3.77) | <0.001 |
| Belarus | Both | 2015 | 2021 | -0.75 (-2.45 to 0.98) | 0.375 |
| Belarus | Female | 1990 | 1993 | 4.49 (0.94 to 8.17) | 0.015 |
| Belarus | Female | 1993 | 2005 | -0.46 (-0.91 to -0.01) | 0.046 |
| Belarus | Female | 2005 | 2015 | -4.68 (-5.27 to -4.09) | <0.001 |
| Belarus | Female | 2015 | 2021 | -0.70 (-2.26 to 0.89) | 0.371 |
| Belarus | Male | 1990 | 1993 | 5.39 (0.76 to 10.23) | 0.024 |
| Belarus | Male | 1993 | 2004 | 0.90 (0.21 to 1.60) | 0.013 |
| Belarus | Male | 2004 | 2016 | -3.35 (-3.84 to -2.87) | <0.001 |
| Belarus | Male | 2016 | 2021 | -0.46 (-2.71 to 1.84) | 0.679 |
| Estonia | Both | 1990 | 1993 | 2.75 (-0.98 to 6.62) | 0.141 |
| Estonia | Both | 1993 | 2005 | -4.16 (-4.68 to -3.64) | <0.001 |
| Estonia | Both | 2005 | 2009 | -14.86 (-18.95 to -10.56) | <0.001 |
| Estonia | Both | 2009 | 2015 | -9.69 (-12.09 to -7.23) | <0.001 |
| Estonia | Both | 2015 | 2021 | 0.57 (-1.74 to 2.93) | 0.616 |
| Estonia | Female | 1990 | 1993 | 2.21 (-2.11 to 6.72) | 0.302 |
| Estonia | Female | 1993 | 2005 | -4.42 (-5.03 to -3.81) | <0.001 |
| Estonia | Female | 2005 | 2009 | -15.99 (-20.94 to -10.72) | <0.001 |
| Estonia | Female | 2009 | 2015 | -10.40 (-13.29 to -7.43) | <0.001 |
| Estonia | Female | 2015 | 2021 | -0.25 (-2.97 to 2.55) | 0.852 |
| Estonia | Male | 1990 | 1993 | 3.40 (-0.11 to 7.03) | 0.057 |
| Estonia | Male | 1993 | 2005 | -4.01 (-4.47 to -3.55) | <0.001 |
| Estonia | Male | 2005 | 2009 | -12.41 (-15.99 to -8.67) | <0.001 |
| Estonia | Male | 2009 | 2015 | -8.68 (-10.66 to -6.65) | <0.001 |
| Estonia | Male | 2015 | 2021 | 1.29 (-0.83 to 3.44) | 0.219 |
| Latvia | Both | 1990 | 1994 | 3.76 (1.21 to 6.37) | 0.006 |
| Latvia | Both | 1994 | 1999 | -4.13 (-6.47 to -1.73) | 0.002 |
| Latvia | Both | 1999 | 2002 | 2.15 (-6.19 to 11.24) | 0.605 |
| Latvia | Both | 2002 | 2011 | -4.59 (-5.57 to -3.59) | <0.001 |
| Latvia | Both | 2011 | 2021 | -0.17 (-1.10 to 0.78) | 0.71 |
| Latvia | Female | 1990 | 1994 | 2.65 (0.22 to 5.14) | 0.034 |
| Latvia | Female | 1994 | 1999 | -3.45 (-5.69 to -1.16) | 0.006 |
| Latvia | Female | 1999 | 2003 | 0.58 (-3.52 to 4.84) | 0.775 |
| Latvia | Female | 2003 | 2010 | -5.66 (-7.08 to -4.22) | <0.001 |
| Latvia | Female | 2010 | 2021 | -0.43 (-1.19 to 0.34) | 0.257 |
| Latvia | Male | 1990 | 1994 | 5.43 (2.37 to 8.59) | 0.002 |
| Latvia | Male | 1994 | 1999 | -5.13 (-7.71 to -2.48) | 0.001 |
| Latvia | Male | 1999 | 2002 | 2.48 (-6.95 to 12.87) | 0.596 |
| Latvia | Male | 2002 | 2012 | -4.25 (-5.15 to -3.33) | <0.001 |
| Latvia | Male | 2012 | 2017 | 1.92 (-1.75 to 5.72) | 0.287 |
| Latvia | Male | 2017 | 2021 | -3.96 (-8.44 to 0.74) | 0.092 |
| Lithuania | Both | 1990 | 1994 | 5.65 (3.54 to 7.81) | <0.001 |
| Lithuania | Both | 1994 | 2000 | -2.35 (-3.78 to -0.89) | 0.003 |
| Lithuania | Both | 2000 | 2007 | 0.44 (-0.68 to 1.56) | 0.425 |
| Lithuania | Both | 2007 | 2021 | -3.10 (-3.47 to -2.73) | <0.001 |
| Lithuania | Female | 1990 | 1994 | 5.40 (3.21 to 7.64) | <0.001 |
| Lithuania | Female | 1994 | 2003 | -2.05 (-2.78 to -1.32) | <0.001 |
| Lithuania | Female | 2003 | 2006 | 2.23 (-4.65 to 9.60) | 0.518 |
| Lithuania | Female | 2006 | 2021 | -3.44 (-3.78 to -3.09) | <0.001 |
| Lithuania | Male | 1990 | 1994 | 6.80 (4.48 to 9.16) | <0.001 |
| Lithuania | Male | 1994 | 1997 | -5.09 (-11.09 to 1.32) | 0.111 |
| Lithuania | Male | 1997 | 2007 | 0.69 (0.07 to 1.31) | 0.031 |
| Lithuania | Male | 2007 | 2021 | -2.54 (-2.92 to -2.16) | <0.001 |
| Republic of Moldova | Both | 1990 | 1994 | 3.15 (-0.12 to 6.54) | 0.058 |
| Republic of Moldova | Both | 1994 | 1998 | -11.91 (-14.88 to -8.84) | <0.001 |
| Republic of Moldova | Both | 1998 | 2005 | 3.97 (2.69 to 5.27) | <0.001 |
| Republic of Moldova | Both | 2005 | 2015 | -1.75 (-2.42 to -1.08) | <0.001 |
| Republic of Moldova | Both | 2015 | 2021 | -5.26 (-6.89 to -3.61) | <0.001 |
| Republic of Moldova | Female | 1990 | 1994 | 2.54 (-0.81 to 6.00) | 0.128 |
| Republic of Moldova | Female | 1994 | 1998 | -13.06 (-16.11 to -9.90) | <0.001 |
| Republic of Moldova | Female | 1998 | 2006 | 3.53 (2.53 to 4.54) | <0.001 |
| Republic of Moldova | Female | 2006 | 2009 | -4.08 (-10.74 to 3.09) | 0.237 |
| Republic of Moldova | Female | 2009 | 2015 | -1.16 (-2.83 to 0.54) | 0.166 |
| Republic of Moldova | Female | 2015 | 2021 | -5.77 (-7.39 to -4.12) | <0.001 |
| Republic of Moldova | Male | 1990 | 1994 | 4.29 (0.91 to 7.77) | 0.016 |
| Republic of Moldova | Male | 1994 | 1998 | -9.85 (-12.84 to -6.77) | <0.001 |
| Republic of Moldova | Male | 1998 | 2005 | 4.04 (2.85 to 5.23) | <0.001 |
| Republic of Moldova | Male | 2005 | 2015 | -1.79 (-2.40 to -1.19) | <0.001 |
| Republic of Moldova | Male | 2015 | 2018 | -7.29 (-13.93 to -0.15) | 0.046 |
| Republic of Moldova | Male | 2018 | 2021 | -0.71 (-6.25 to 5.16) | 0.795 |
| Russian Federation | Both | 1990 | 1994 | 7.42 (5.10 to 9.80) | <0.001 |
| Russian Federation | Both | 1994 | 1997 | -2.89 (-8.83 to 3.45) | 0.343 |
| Russian Federation | Both | 1997 | 2003 | 0.99 (-0.50 to 2.50) | 0.18 |
| Russian Federation | Both | 2003 | 2012 | -6.39 (-7.11 to -5.67) | <0.001 |
| Russian Federation | Both | 2012 | 2021 | -2.97 (-3.82 to -2.12) | <0.001 |
| Russian Federation | Female | 1990 | 1994 | 6.77 (4.60 to 8.98) | <0.001 |
| Russian Federation | Female | 1994 | 1997 | -2.51 (-8.25 to 3.59) | 0.391 |
| Russian Federation | Female | 1997 | 2003 | 0.79 (-0.63 to 2.23) | 0.26 |
| Russian Federation | Female | 2003 | 2012 | -6.52 (-7.23 to -5.80) | <0.001 |
| Russian Federation | Female | 2012 | 2021 | -3.01 (-3.87 to -2.13) | <0.001 |
| Russian Federation | Male | 1990 | 1994 | 7.85 (5.29 to 10.47) | <0.001 |
| Russian Federation | Male | 1994 | 1997 | -3.37 (-9.71 to 3.42) | 0.303 |
| Russian Federation | Male | 1997 | 2003 | 0.47 (-1.13 to 2.11) | 0.546 |
| Russian Federation | Male | 2003 | 2012 | -6.41 (-7.18 to -5.63) | <0.001 |
| Russian Federation | Male | 2012 | 2021 | -2.83 (-3.82 to -1.83) | <0.001 |
| Ukraine | Both | 1990 | 1994 | 3.29 (1.64 to 4.97) | 0.001 |
| Ukraine | Both | 1994 | 1998 | -3.16 (-5.51 to -0.76) | 0.013 |
| Ukraine | Both | 1998 | 2002 | 0.71 (-1.89 to 3.38) | 0.575 |
| Ukraine | Both | 2002 | 2016 | -4.67 (-4.93 to -4.41) | <0.001 |
| Ukraine | Both | 2016 | 2021 | -0.13 (-3.19 to 3.02) | 0.93 |
| Ukraine | Female | 1990 | 1993 | 4.32 (2.31 to 6.38) | <0.001 |
| Ukraine | Female | 1993 | 1998 | -2.47 (-3.64 to -1.29) | <0.001 |
| Ukraine | Female | 1998 | 2003 | 0.04 (-1.18 to 1.27) | 0.95 |
| Ukraine | Female | 2003 | 2006 | -7.38 (-11.15 to -3.45) | 0.001 |
| Ukraine | Female | 2006 | 2016 | -4.54 (-4.90 to -4.18) | <0.001 |
| Ukraine | Female | 2016 | 2021 | -0.47 (-3.14 to 2.27) | 0.715 |
| Ukraine | Male | 1990 | 1995 | 2.78 (1.45 to 4.13) | <0.001 |
| Ukraine | Male | 1995 | 1998 | -4.55 (-9.59 to 0.78) | 0.088 |
| Ukraine | Male | 1998 | 2002 | 1.34 (-1.41 to 4.17) | 0.322 |
| Ukraine | Male | 2002 | 2016 | -4.26 (-4.52 to -4.00) | <0.001 |
| Ukraine | Male | 2016 | 2021 | -0.49 (-4.51 to 3.69) | 0.804 |

APC, Annual percentage change (Positive APC values indicate an increasing trend in ASMR, while negative APC values indicate a decreasing trend.); ASMR, age-standardized mortality rate; P-value: Statistical significance level. P-values < 0.05 indicate a statistically significant trend; 95% UI: 95% uncertainty interval.
