# Supplementary material for: The burden of ischemic stroke in Eastern Europe from 1990 to 2021
Source: BMC Neurol. 2025 Feb 22;25:74. doi: 10.1186/s12883-025-04081-z (PMC11846382; doi:10.1186/s12883-025-04081-z)
Supplement: Supplementary file 2 — Supplementary Material 2 [file 12883_2025_4081_MOESM2_ESM.zip › Supplementary Table 1-10/Supplementary Table 9.docx]

Supplementary Table 9. Annual percentage change in age-standardized disability-adjusted life years rates of ischemic stroke across Eastern European countries from 1990 to 2021.

| **location** | **sex** | **Segment Start** | **Segment End** | **APC**  **(95% UI)** | **P-Value** |
| --- | --- | --- | --- | --- | --- |
| Eastern Europe | Both | 1990 | 1994 | 6.76 (4.91 to 8.64) | <0.001 |
| Eastern Europe | Both | 1994 | 1998 | -2.73 (-5.20 to -0.19) | 0.037 |
| Eastern Europe | Both | 1998 | 2003 | 0.99 (-0.65 to 2.65) | 0.221 |
| Eastern Europe | Both | 2003 | 2013 | -5.30 (-5.79 to -4.82) | <0.001 |
| Eastern Europe | Both | 2013 | 2021 | -2.64 (-3.53 to -1.74) | <0.001 |
| Eastern Europe | Female | 1990 | 1994 | 5.72 (4.02 to 7.45) | <0.001 |
| Eastern Europe | Female | 1994 | 1998 | -2.58 (-4.87 to -0.24) | 0.033 |
| Eastern Europe | Female | 1998 | 2003 | 0.38 (-1.17 to 1.95) | 0.615 |
| Eastern Europe | Female | 2003 | 2013 | -5.58 (-6.05 to -5.10) | <0.001 |
| Eastern Europe | Female | 2013 | 2021 | -2.71 (-3.59 to -1.83) | <0.001 |
| Eastern Europe | Male | 1990 | 1994 | 7.66 (5.51 to 9.85) | <0.001 |
| Eastern Europe | Male | 1994 | 1998 | -3.08 (-5.71 to -0.36) | 0.029 |
| Eastern Europe | Male | 1998 | 2003 | 1.38 (-0.28 to 3.06) | 0.097 |
| Eastern Europe | Male | 2003 | 2013 | -4.96 (-5.45 to -4.47) | <0.001 |
| Eastern Europe | Male | 2013 | 2021 | -2.65 (-3.74 to -1.54) | <0.001 |
| Belarus | Both | 1990 | 1995 | 3.55 (1.77 to 5.36) | <0.001 |
| Belarus | Both | 1995 | 2005 | -0.16 (-0.82 to 0.51) | 0.632 |
| Belarus | Both | 2005 | 2015 | -4.73 (-5.35 to -4.10) | <0.001 |
| Belarus | Both | 2015 | 2021 | -0.92 (-2.53 to 0.72) | 0.254 |
| Belarus | Female | 1990 | 1995 | 2.82 (1.28 to 4.39) | 0.001 |
| Belarus | Female | 1995 | 2005 | -0.90 (-1.51 to -0.28) | 0.006 |
| Belarus | Female | 2005 | 2015 | -4.89 (-5.44 to -4.32) | <0.001 |
| Belarus | Female | 2015 | 2021 | -0.89 (-2.31 to 0.55) | 0.213 |
| Belarus | Male | 1990 | 1993 | 6.12 (2.38 to 10.00) | 0.003 |
| Belarus | Male | 1993 | 2003 | 1.23 (0.61 to 1.86) | 0.001 |
| Belarus | Male | 2003 | 2011 | -2.87 (-3.63 to -2.11) | <0.001 |
| Belarus | Male | 2011 | 2014 | -6.84 (-11.39 to -2.05) | 0.008 |
| Belarus | Male | 2014 | 2021 | -1.09 (-1.96 to -0.22) | 0.017 |
| Estonia | Both | 1990 | 1994 | 1.47 (-0.65 to 3.63) | 0.165 |
| Estonia | Both | 1994 | 2005 | -4.54 (-5.07 to -4.02) | <0.001 |
| Estonia | Both | 2005 | 2009 | -13.49 (-17.12 to -9.71) | <0.001 |
| Estonia | Both | 2009 | 2015 | -8.11 (-10.20 to -5.98) | <0.001 |
| Estonia | Both | 2015 | 2021 | 0.27 (-1.77 to 2.36) | 0.783 |
| Estonia | Female | 1990 | 1993 | 2.00 (-2.25 to 6.42) | 0.337 |
| Estonia | Female | 1993 | 2002 | -4.27 (-5.11 to -3.43) | <0.001 |
| Estonia | Female | 2002 | 2006 | -7.86 (-12.09 to -3.43) | 0.002 |
| Estonia | Female | 2006 | 2009 | -14.68 (-23.70 to -4.60) | 0.008 |
| Estonia | Female | 2009 | 2015 | -8.18 (-10.83 to -5.45) | <0.001 |
| Estonia | Female | 2015 | 2021 | -0.80 (-3.28 to 1.75) | 0.511 |
| Estonia | Male | 1990 | 1994 | 2.14 (0.09 to 4.23) | 0.042 |
| Estonia | Male | 1994 | 2005 | -4.23 (-4.71 to -3.75) | <0.001 |
| Estonia | Male | 2005 | 2010 | -11.66 (-13.70 to -9.57) | <0.001 |
| Estonia | Male | 2010 | 2015 | -7.36 (-9.86 to -4.80) | <0.001 |
| Estonia | Male | 2015 | 2021 | 0.94 (-0.93 to 2.85) | 0.307 |
| Latvia | Both | 1990 | 1994 | 4.33 (1.70 to 7.02) | 0.003 |
| Latvia | Both | 1994 | 1999 | -4.34 (-6.70 to -1.92) | 0.002 |
| Latvia | Both | 1999 | 2003 | 0.70 (-3.50 to 5.08) | 0.735 |
| Latvia | Both | 2003 | 2011 | -4.52 (-5.70 to -3.33) | <0.001 |
| Latvia | Both | 2011 | 2021 | -0.71 (-1.62 to 0.22) | 0.125 |
| Latvia | Female | 1990 | 1994 | 2.79 (0.42 to 5.22) | 0.023 |
| Latvia | Female | 1994 | 1999 | -3.71 (-5.77 to -1.60) | 0.002 |
| Latvia | Female | 1999 | 2003 | 0.18 (-3.45 to 3.94) | 0.92 |
| Latvia | Female | 2003 | 2011 | -5.01 (-6.09 to -3.93) | <0.001 |
| Latvia | Female | 2011 | 2021 | -0.75 (-1.56 to 0.07) | 0.071 |
| Latvia | Male | 1990 | 1994 | 6.34 (2.96 to 9.84) | 0.001 |
| Latvia | Male | 1994 | 1999 | -5.35 (-8.01 to -2.61) | 0.001 |
| Latvia | Male | 1999 | 2002 | 2.55 (-7.56 to 13.77) | 0.613 |
| Latvia | Male | 2002 | 2012 | -3.81 (-4.71 to -2.91) | <0.001 |
| Latvia | Male | 2012 | 2017 | 0.94 (-2.68 to 4.70) | 0.593 |
| Latvia | Male | 2017 | 2021 | -3.92 (-8.34 to 0.73) | 0.091 |
| Lithuania | Both | 1990 | 1994 | 4.68 (2.83 to 6.56) | <0.001 |
| Lithuania | Both | 1994 | 2000 | -2.54 (-3.74 to -1.33) | <0.001 |
| Lithuania | Both | 2000 | 2004 | -0.27 (-2.98 to 2.51) | 0.835 |
| Lithuania | Both | 2004 | 2007 | 3.07 (-2.25 to 8.69) | 0.242 |
| Lithuania | Both | 2007 | 2016 | -2.71 (-3.38 to -2.04) | <0.001 |
| Lithuania | Both | 2016 | 2021 | -4.93 (-6.65 to -3.18) | <0.001 |
| Lithuania | Female | 1990 | 1994 | 4.22 (2.34 to 6.14) | <0.001 |
| Lithuania | Female | 1994 | 2004 | -2.11 (-2.61 to -1.61) | <0.001 |
| Lithuania | Female | 2004 | 2007 | 3.05 (-2.65 to 9.07) | 0.282 |
| Lithuania | Female | 2007 | 2016 | -3.16 (-3.89 to -2.43) | <0.001 |
| Lithuania | Female | 2016 | 2021 | -6.04 (-7.77 to -4.28) | <0.001 |
| Lithuania | Male | 1990 | 1994 | 5.80 (3.26 to 8.40) | <0.001 |
| Lithuania | Male | 1994 | 1997 | -5.08 (-12.00 to 2.37) | 0.164 |
| Lithuania | Male | 1997 | 2004 | 0.00 (-1.24 to 1.26) | 0.998 |
| Lithuania | Male | 2004 | 2007 | 4.67 (-2.91 to 12.84) | 0.219 |
| Lithuania | Male | 2007 | 2021 | -2.58 (-3.03 to -2.12) | <0.001 |
| Republic of Moldova | Both | 1990 | 1994 | 3.81 (0.40 to 7.33) | 0.03 |
| Republic of Moldova | Both | 1994 | 1998 | -9.01 (-12.02 to -5.90) | <0.001 |
| Republic of Moldova | Both | 1998 | 2006 | 4.15 (3.14 to 5.16) | <0.001 |
| Republic of Moldova | Both | 2006 | 2015 | -2.03 (-2.85 to -1.20) | <0.001 |
| Republic of Moldova | Both | 2015 | 2021 | -4.50 (-6.10 to -2.87) | <0.001 |
| Republic of Moldova | Female | 1990 | 1994 | 3.09 (0.06 to 6.21) | 0.046 |
| Republic of Moldova | Female | 1994 | 1998 | -10.06 (-12.81 to -7.23) | <0.001 |
| Republic of Moldova | Female | 1998 | 2006 | 3.81 (2.94 to 4.68) | <0.001 |
| Republic of Moldova | Female | 2006 | 2016 | -2.46 (-3.06 to -1.86) | <0.001 |
| Republic of Moldova | Female | 2016 | 2019 | -6.78 (-13.58 to 0.55) | 0.067 |
| Republic of Moldova | Female | 2019 | 2021 | 0.28 (-8.89 to 10.36) | 0.952 |
| Republic of Moldova | Male | 1990 | 1994 | 4.80 (0.13 to 9.68) | 0.044 |
| Republic of Moldova | Male | 1994 | 1998 | -7.47 (-11.45 to -3.31) | 0.002 |
| Republic of Moldova | Male | 1998 | 2006 | 4.37 (3.11 to 5.64) | <0.001 |
| Republic of Moldova | Male | 2006 | 2015 | -1.78 (-2.71 to -0.84) | 0.001 |
| Republic of Moldova | Male | 2015 | 2021 | -4.24 (-6.04 to -2.41) | <0.001 |
| Russian Federation | Both | 1990 | 1994 | 7.93 (5.42 to 10.51) | <0.001 |
| Russian Federation | Both | 1994 | 1998 | -2.75 (-6.13 to 0.75) | 0.115 |
| Russian Federation | Both | 1998 | 2003 | 1.60 (-0.62 to 3.87) | 0.148 |
| Russian Federation | Both | 2003 | 2012 | -6.08 (-6.84 to -5.31) | <0.001 |
| Russian Federation | Both | 2012 | 2021 | -3.12 (-4.07 to -2.16) | <0.001 |
| Russian Federation | Female | 1990 | 1994 | 6.79 (4.52 to 9.12) | <0.001 |
| Russian Federation | Female | 1994 | 1998 | -2.38 (-5.49 to 0.84) | 0.136 |
| Russian Federation | Female | 1998 | 2003 | 0.87 (-1.16 to 2.94) | 0.384 |
| Russian Federation | Female | 2003 | 2013 | -6.13 (-6.76 to -5.49) | <0.001 |
| Russian Federation | Female | 2013 | 2021 | -2.83 (-3.96 to -1.68) | <0.001 |
| Russian Federation | Male | 1990 | 1994 | 8.60 (5.64 to 11.64) | <0.001 |
| Russian Federation | Male | 1994 | 1998 | -3.21 (-6.81 to 0.54) | 0.088 |
| Russian Federation | Male | 1998 | 2003 | 1.74 (-0.64 to 4.18) | 0.143 |
| Russian Federation | Male | 2003 | 2012 | -5.89 (-6.68 to -5.09) | <0.001 |
| Russian Federation | Male | 2012 | 2021 | -3.04 (-4.13 to -1.94) | <0.001 |
| Ukraine | Both | 1990 | 1995 | 2.96 (1.93 to 4.00) | <0.001 |
| Ukraine | Both | 1995 | 1998 | -4.46 (-8.56 to -0.16) | 0.043 |
| Ukraine | Both | 1998 | 2003 | -0.31 (-1.76 to 1.16) | 0.664 |
| Ukraine | Both | 2003 | 2016 | -4.00 (-4.27 to -3.73) | <0.001 |
| Ukraine | Both | 2016 | 2021 | -0.74 (-3.45 to 2.06) | 0.583 |
| Ukraine | Female | 1990 | 1994 | 3.16 (2.13 to 4.20) | <0.001 |
| Ukraine | Female | 1994 | 1998 | -3.31 (-4.76 to -1.83) | <0.001 |
| Ukraine | Female | 1998 | 2003 | -1.07 (-2.13 to -0.01) | 0.049 |
| Ukraine | Female | 2003 | 2016 | -4.28 (-4.48 to -4.08) | <0.001 |
| Ukraine | Female | 2016 | 2021 | -0.50 (-2.70 to 1.74) | 0.64 |
| Ukraine | Male | 1990 | 1995 | 3.71 (2.70 to 4.73) | <0.001 |
| Ukraine | Male | 1995 | 1998 | -4.74 (-8.47 to -0.86) | 0.02 |
| Ukraine | Male | 1998 | 2001 | 0.94 (-3.31 to 5.37) | 0.65 |
| Ukraine | Male | 2001 | 2008 | -2.15 (-2.84 to -1.45) | <0.001 |
| Ukraine | Male | 2008 | 2011 | -6.10 (-9.54 to -2.52) | 0.003 |
| Ukraine | Male | 2011 | 2021 | -2.65 (-3.25 to -2.05) | <0.001 |

APC, Annual percentage change (Positive APC values indicate an increasing trend in ASDR, while negative APC values indicate a decreasing trend.); ASDR, age-standardized DALYs rate; P-value: Statistical significance level. P-values < 0.05 indicate a statistically significant trend; 95% UI: 95% uncertainty interval.
